# iNitro-Tyr: Prediction of Nitrotyrosine Sites in Proteins with General Pseudo Amino Acid Composition

**DOI:** 10.1371/journal.pone.0105018

**Published:** 2014-08-14

**Authors:** Yan Xu, Xin Wen, Li-Shu Wen, Ling-Yun Wu, Nai-Yang Deng, Kuo-Chen Chou

**Affiliations:** 1 Department of Information and Computer Science, University of Science and Technology Beijing, Beijing, China; 2 College of Sciences, Liaoning Shiyou University, FuShun, China; 3 Institute of Applied Mathematics, Academy of Mathematics and Systems Science, Chinese Academy of Sciences, Beijing, China; 4 College of Science, China Agricultural University, Beijing, China; 5 Center of Excellence in Genomic Medicine Research (CEGMR), King Abdulaziz University, Jeddah, Saudi Arabia; 6 Gordon Life Science Institute, Boston, Massachusetts, United States of America; Institut Jacques Monod, France

## Abstract

Nitrotyrosine is one of the post-translational modifications (PTMs) in proteins that occurs when their tyrosine residue is nitrated. Compared with healthy people, a remarkably increased level of nitrotyrosine is detected in those suffering from rheumatoid arthritis, septic shock, and coeliac disease. Given an uncharacterized protein sequence that contains many tyrosine residues, which one of them can be nitrated and which one cannot? This is a challenging problem, not only directly related to in-depth understanding the PTM’s mechanism but also to the nitrotyrosine-based drug development. Particularly, with the avalanche of protein sequences generated in the postgenomic age, it is highly desired to develop a high throughput tool in this regard. Here, a new predictor called “iNitro-Tyr” was developed by incorporating the position-specific dipeptide propensity into the general pseudo amino acid composition for discriminating the nitrotyrosine sites from non-nitrotyrosine sites in proteins. It was demonstrated via the rigorous jackknife tests that the new predictor not only can yield higher success rate but also is much more stable and less noisy. A web-server for iNitro-Tyr is accessible to the public at http://app.aporc.org/iNitro-Tyr/. For the convenience of most experimental scientists, we have further provided a protocol of step-by-step guide, by which users can easily get their desired results without the need to follow the complicated mathematics that were presented in this paper just for the integrity of its development process. It has not escaped our notice that the approach presented here can be also used to deal with the other PTM sites in proteins.

## Introduction

**Figure 1:**
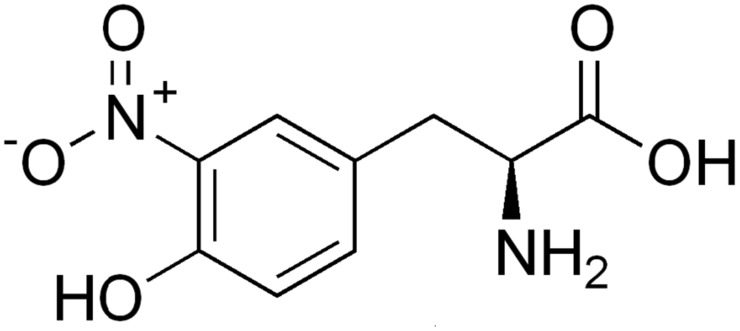
A schematic drawing to show protein nitrotyrosine.

As one of the post-translational modifications (PTMs) of proteins, nitrotyrosine is a product of tyrosine nitration mediated by reactive nitrogen species such as peroxynitrite anion and nitrogen dioxide (**Fig. 1**). Compared with the fluids from healthy people, a remarkably increased level of nitrotyrosine is detected in those suffering from rheumatoid arthritis, septic shock, and coeliac disease. Accordingly, knowledge of nitrotyrosine sites in proteins is very useful for both basic research and drug development. Although conventional experimental methods did provide useful insight into the biological roles of tyrosine nitration [1]–[3], it is time-consuming and expensive to determine the nitrotyrosine sites based on the experimental approach alone. Particularly, identification of endogenous 3-NTyr modifications remains largely elusive (see, e.g., [4]–[7]). With the avalanche of protein sequences generated in the postgenomic age, it is highly desired to develop computational methods for identifying the nitrotyrosine sites in proteins. The present study was initiated in an attempt to propose a new method for identifying the nitrotyrosine sites in proteins in hope that it can play a complementary role with the existing methods in this area.

As summarized in [8] and demonstrated in a series of recent publications [9]–[21], to establish a really useful statistical predictor for a biological system, we need to consider the following procedures: (i) construct or select a valid benchmark dataset to train and test the predictor; (ii) formulate the biological samples with an effective mathematical expression that can truly capture their essence and intrinsic correlation with the target to be predicted; (iii) introduce or develop a powerful algorithm (or engine) to operate the prediction; (iv) properly perform cross-validation tests to objectively evaluate the anticipated accuracy; (v) establish a user-friendly web-server that is accessible to the public. Below, let us describe how to deal with these steps one by one.

## Materials and Methods

### 1. Benchmark Dataset

To develop a statistical predictor, it is fundamentally important to establish a reliable and stringent benchmark dataset to train and test the predictor. If the benchmark dataset contains some errors, the predictor trained by it must be unreliable and the accuracy tested by it would be completely meaningless.

**Figure 2:**
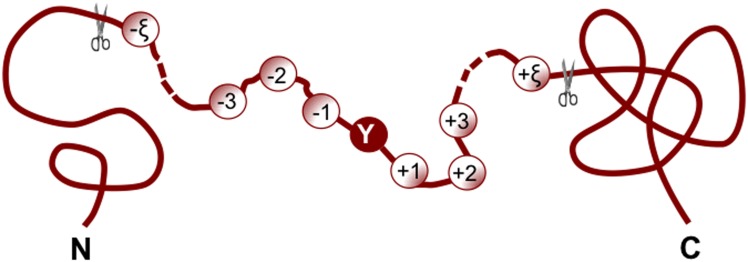
An illustration to show Chou’s scheme for a peptide of 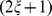 residues with tyrosine (Y) at the center. Adapted from Chou [55], [76] with permission.

For facilitating description later, let us adopt the Chou’s peptide formulation here that was used for studying HIV protease cleavage sites [22], [23], specificity of GalNAc-transferase [24], and signal peptide cleavage sites [25]. According to Chou’s scheme, a potential nitrotyrosine peptide, i.e., a peptide with Tyr (namely Y) located at its center (**Fig. 2**), can be expressed as
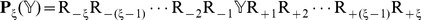
(1)where the subscript 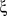 is an integer, 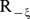 represents the 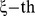 upstream amino acid residue from the center, 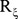 the 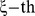 downstream amino acid residue, and so forth. A 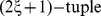 peptide 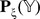 can be further classified into the following categories:
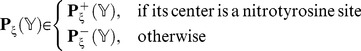
(2)where 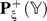 represents a true nitrotyrosine peptide, 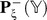 a false nitrotyrosine peptide, and 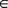 represents “a member of” in the set theory.

As pointed out by a comprehensive review [26], there is no need to separate a benchmark dataset into a training dataset and a testing dataset for examining the performance of a prediction method if it is tested by the jackknife test or subsampling (K-fold) cross-validation test. Thus, the benchmark dataset for the current study can be formulated as
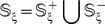
(3)where 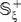 only contains the samples of 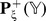, i.e., the nitrotyrosine peptides; 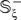 only contains the samples of 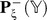, i.e., the non-nitrotyrosine peptide (cf. **Eq. 2**); and 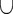 represents the symbol for “union” in the set theory.

Since the length of the peptide 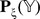 is 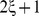
(**Eq. 1**), the benchmark dataset with different values of 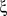 will contain peptides of different numbers of amino acid residues, as formulated by
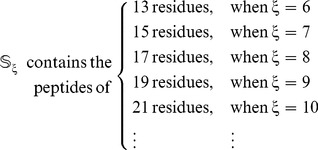
(4)


**Figure 3:**
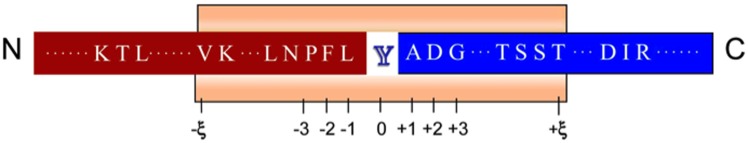
Illustration to show the peptide segment highlighted by sliding the scaled window 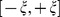
 **along a protein sequence.** During the sliding process, the scales on the window are aligned with different amino acids so as to define different peptide segments. When, and only when, the scale 0 is aligned with Y (tyrosine), is the 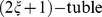 peptide segment seen within the window regarded as a potential nitrotyrosine peptide. Adapted from Chou [55], [77] with permission.

The detailed procedures to construct 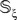 are as follows. (i) Its elements were derived based on the same 546 source proteins used in [27] that contain 1,044 nitrotyrosine sites (see columns 1 and 2 of Supporting Information S1). (ii) Slide a flexible window of 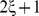 amino acids (**Fig. 3**) along each of the 546 protein sequences taken from the Uni-Prot database (version 2014_01). (iii) Collect only those peptide segments with Y (tyrosine) at the center. (iv) If the upstream or downstream in a protein was less than 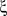, the lacking residue was filled with a dummy residue “X” [28]. (v) Those peptide samples thus obtained were put into the positive subset 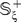 if their centers have been experimentally confirmed as the nitrotyrosine sites; otherwise, into the negative subset 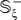.

By following the aforementioned procedures, five such benchmark datasets (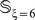,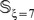,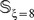,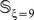, and 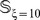) had been constructed. Each of these datasets contained 1,044 nitrotyrosine peptides and 7,669 non-nitrotyrosine peptides. Note that the sample numbers thus obtained have some minor difference with those in [27]. This is because some proteins originally used in [27] have been removed or replaced in the updated version of the Uni-Prot database.

**Figure 4:**
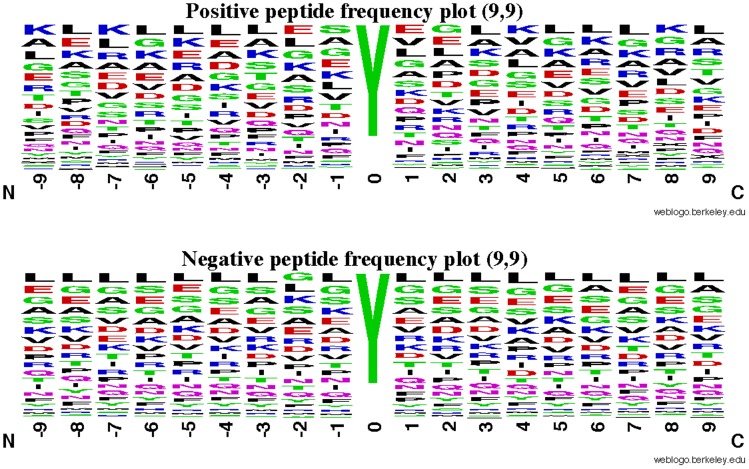
A sequence logo plot to show the difference between the positive and negative peptides. The window’s size is 19 when 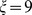. See Eq. 1 and the legend of Fig. 3 for further explanation.

**Figure 5:**
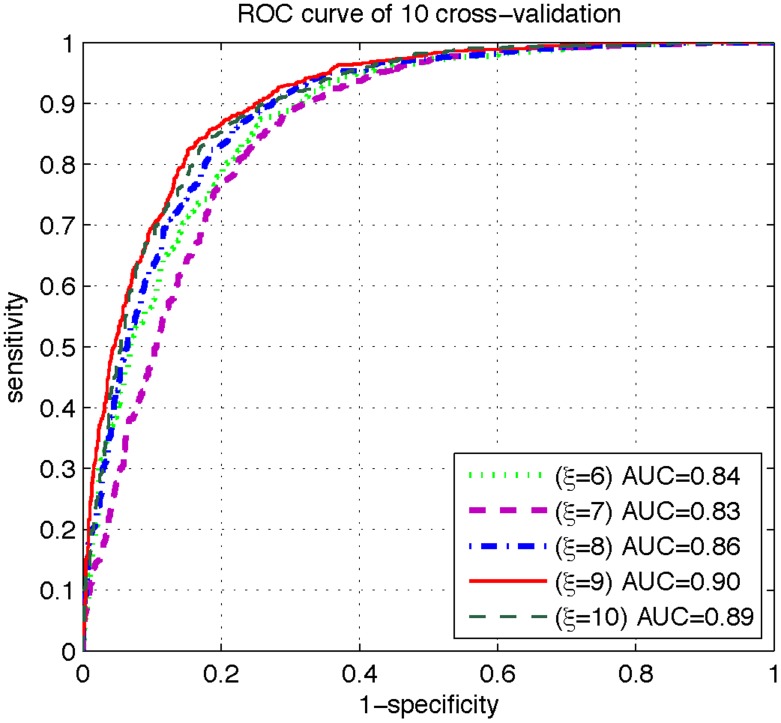
A plot to show the different ROC curves obtained by the 10-fold cross-validation under different 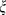 values. As we can see, when 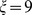, the corresponding AUC (i.e., the area under its curve) is the largest, meaning the most promising compared with the other values of 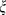.

However, it was observed via preliminary trials that when 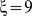, i.e., the peptide samples concerned were formed by 19 residues, the corresponding results were most promising (see **Fig. 4** and **Fig. 5**). Accordingly, we choose 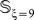 as the benchmark dataset for further investigation. Thus, **Eq. 3** can be reduced to
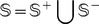
(5)where 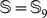, 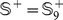 containing 1,044 nitrotyrosine peptide samples, and 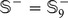 containing 7,669 non-nitrotyrosine peptide samples. The detailed 19-tuple peptide sequences and their positions in proteins are given in Supporting Information S1.

### 2. Feature Vector and Pseudo Amino Acid Composition

One of the most important but also most difficult problems in computational biology today is how to effectively formulate a biological sequence with a discrete model or a vector, yet still keep considerable sequence order information. This is because all the existing operation engines, such as correlation angle approach [29], covariance discriminant [30], neural network [31], support vector machine (SVM) [32], random forest [33], conditional random field [28], K-nearest neighbor (KNN) [34], OET-KNN [35], Fuzzy K-nearest neighbor [36], ML-KNN algorithm [37], and SLLE algorithm [30], can only handle vector but not sequence samples. However, a vector defined in a discrete model may totally miss the sequence-order information. To deal with such a dilemma, the approach of pseudo amino acid composition [38] or Chou’s PseAAC [39] was proposed. Ever since it was introduced in 2001 [38], the concept of PseAAC has been rapidly penetrated into almost all the areas of computational proteomics, such as in identifying bacterial virulent proteins [40], predicting anticancer peptides [41], predicting protein subcellular location [42], predicting membrane protein types [43], analyzing genetic sequence [44], predicting GABA(A) receptor proteins [45], identifying antibacterial peptides [46], predicting anticancer peptides [41], identifying allergenic proteins [47], predicting metalloproteinase family [48], identifying GPCRs and their types [49], identifying protein quaternary structural attributes [50], among many others (see a long list of references cited in a 2014 article [51]). Recently, the concept of PseAAC was further extended to represent the feature vectors of DNA and nucleotides [9], as well as other biological samples (see, e.g., [52]). Because it has been widely and increasingly used, recently three types of powerful open access soft-ware, called ‘PseAAC-Builder’ [53], ‘propy’ [54], and ‘PseAAC-General’ [51], were established: the former two are for generating various modes of Chou’s special PseAAC; while the 3^rd^ one for those of Chou’s general PseAAC.

According to a comprehensive review [8], PseAAC can be generally formulated as
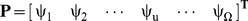
(6)where 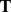 is the transpose operator, while 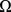 an integer to reflect the vector’s dimension. The value of 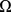 as well as the components 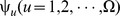 in **Eq. 6** will depend on how to extract the desired information from a protein/peptide sequence. Below, let us describe how to extract the useful information from the benchmark datasets to define the peptide samples via **Eq. 6**.

For convenience in formulation, let rewrite **Eq. 1** as follows
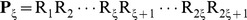
(7)where 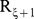, the residue at the center of the peptide, is tyrosine (Y), and all the other residues 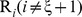 can be any of the 20 native amino acids or the dummy code X as defined above. Hereafter, let us use the numerical codes 1, 2, 3, …, 20 to represent the 20 native amino acids according to the alphabetic order of their single letter codes, and use 21 to represent the dummy amino acid X. Accordingly, the number of possible different dipeptides will be 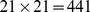, and the number of dipeptide subsite positions on the sequence of **Eq. 7** will be 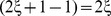.

Now, let us introduce a positive and a negative PSDP (position-specific dipeptide propensity) matrix, as given below
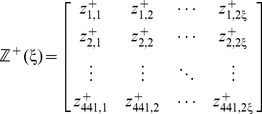
(8a)




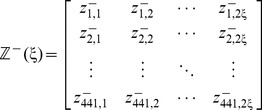
(8b)where the element
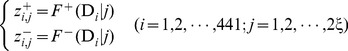
(9)and



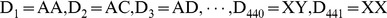
(10)In **Eq. 9**, 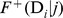 is the occurrence frequency of the 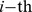 dipeptide (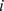 = 1,2,
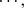
441) at the 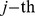 subsite on the sequence of **Eq. 7** (or the 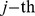 column in the positive subset dataset 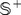) that can be easily derived using the method described in [55] from the sequences in the Supporting Information S1; while 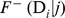 is the corresponding occurrence frequency but derived from the negative subset dataset 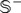. Thus, for the peptide sequence of **Eq. 7**, its attribute to the positive set 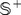 or negative set 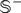 can be formulated by a 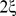-D (dimension) vector 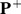 or 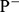, as defined by [23]

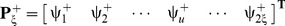
(11a)




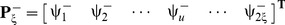
(11b)where



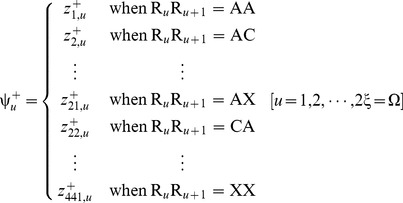
(12a)

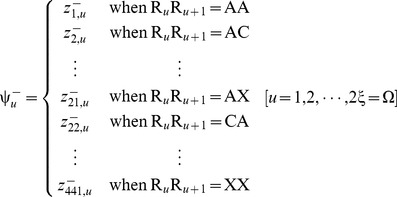
(12b)where 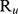 and 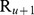 represent the residues in the 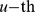 and 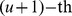 positions of the peptide concerned.

### 3. Discriminant Function Approach

Now in the 2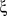-D space, let us define an ideal nitrotyrosine peptide 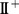 [22] and an ideal non-nitrotyrosine peptide 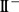 as expressed by
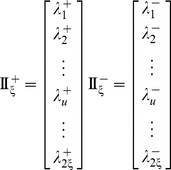
(13)where 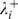 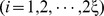 is the upper limit of the corresponding matrix element in **Eq. 12a**, and 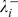 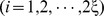 is the upper limit of the corresponding matrix element in **Eq. 12b**. Theoretically speaking, each of these hypothetical upper limits in **Eq. 13** should be 1 [23]. Thus, the similarity score of 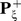 with 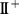 and that of 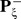 with 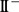 can be defined as



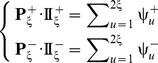
(14)Similar to the treatment in [23], let us define a discriminant function Δ given by
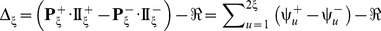
(15)where 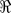 is the adjust parameter used to optimize the overall success rate when the positive and negative benchmark datasets are highly imbalanced in size. Now the peptide 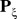 of **Eq. 7** can be identified according to the following rule



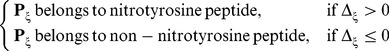
(16)The predictor obtained via the above procedures is called **iNitro-Tyr**. How to properly and objectively evaluate the anticipated accuracy of a new predictor and how to make it easily accessible and user-friendly are the two key issues that will have important impacts on its application value [56]. Below, let us address these problems.

## Results and Discussion

### 1. Metrics for Scoring Prediction Quality

In literature the following four metrics are often used to score the quality of a predictor at four different angles
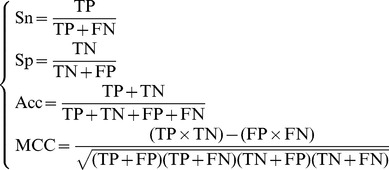
(17)where TP represents the number of the true positive; TN, the number of the true negative; FP, the number of the false positive; FN, the number of the false negative; Sn, the sensitivity; Sp, the specificity; Acc, the accuracy; MCC, the Mathew’s correlation coefficient. To most biologists, unfortunately, the four metrics as formulated in **Eq. 17** are not quite intuitive and easy-to-understand, particularly the equation for MCC. Here let us adopt the formulation proposed recently in [9], [11], [28] based on the symbols introduced by Chou [25], [55] in predicting signal peptides. According to the formulation, the same four metrics can be expressed as
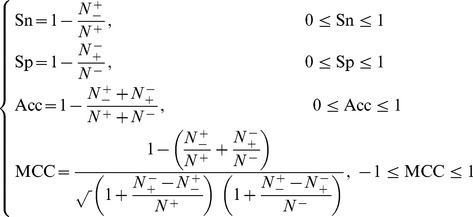
(18)where 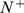 is the total number of the nitrotyrosine peptides investigated while 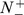 the number of the nitrotyrosine peptides incorrectly predicted as the non-nitrotyrosine peptides; 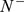 the total number of the non-nitrotyrosine peptides investigated while 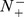 the number of the non-nitrotyrosine peptides incorrectly predicted as the nitrotyrosine peptides [57].

Now, it is crystal clear from **Eq. 18** that when 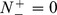 meaning none of the nitrotyrosine peptides was incorrectly predicted to be a non-nitrotyrosine peptide, we have the sensitivity 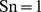. When 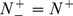 meaning that all the nitrotyrosine peptides were incorrectly predicted as the non-nitrotyrosine peptides, we have the sensitivity 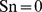. Likewise, when 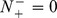 meaning none of the non-nitrotyrosine peptides was incorrectly predicted to be the nitrotyrosine peptide, we have the specificity 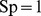; whereas 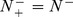 meaning all the non-nitrotyrosine peptides were incorrectly predicted as the nitrotyrosine peptides, we have the specificity 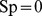. When 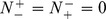 meaning that none of nitrotyrosine peptides in the positive dataset 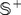 and none of the non- nitrotyrosine peptides in the negative dataset 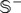 was incorrectly predicted, we have the overall accuracy 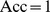 and 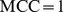; when 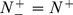 and 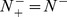 meaning that all the nitrotyrosine peptides in the positive dataset 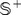 and all the non- nitrotyrosine peptides in the negative dataset 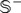 were incorrectly predicted, we have the overall accuracy 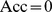 and 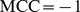; whereas when 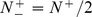 and 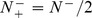 we have 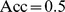 and 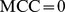 meaning no better than random prediction. As we can see from the above discussion based on **Eq. 18**, the meanings of sensitivity, specificity, overall accuracy, and Mathew’s correlation coefficient have become much more intuitive and easier-to-understand.

It is instructive to point out, however, the set of metrics in **Eqs. 17–18** is valid only for the single-label systems. For the multi-label systems, such as those for the subcellular localization of multiplex proteins (see, e.g., [58]–[62]) where a protein may have two or more locations, and those for the functional types of antimicrobial peptides (see, e.g., [63] where a peptide may possess two or more functional types, a completely different set of metrics is needed as elaborated in [37].

### 2. Jackknife Cross-Validation

With a set of clear and valid metrics as defined in **Eq. 18** to measure the quality of a predictor, the next thing we need to consider is how to objectively derive the values of these metrics for a predictor.

In statistical prediction, the following three cross-validation methods are often used to calculate the metrics of **Eq. 18** for evaluating the quality of a predictor: independent dataset test, subsampling test, and jackknife test [64]. However, of the three test methods, the jackknife test is deemed the least arbitrary that can always yield an unique result for a given benchmark dataset [65]. The reasons are as follows. (i) For the independent dataset test, although all the samples used to test the predictor are outside the training dataset used to train it so as to exclude the “memory” effect or bias, the way of how to select the independent samples to test the predictor could be quite arbitrary unless the number of independent samples is sufficiently large. This kind of arbitrariness might result in completely different conclusions. For instance, a predictor achieving a higher success rate than the other predictor for a given independent testing dataset might fail to keep so when tested by another independent testing dataset [64]. (ii) For the subsampling test, the concrete procedure usually used in literatures is the 5-fold, 7-fold or 10-fold cross-validation. The problem with this kind of subsampling test is that the number of possible selections in dividing a benchmark dataset is an astronomical figure even for a very simple dataset, as demonstrated by Eqs.28–30 in [8]. Therefore, in any actual subsampling cross-validation tests, only an extremely small fraction of the possible selections are taken into account. Since different selections will always lead to different results even for a same benchmark dataset and a same predictor, the subsampling test cannot avoid the arbitrariness either. A test method unable to yield an unique outcome cannot be deemed as a good one. (iii) In the jackknife test, all the samples in the benchmark dataset will be singled out one-by-one and tested by the predictor trained by the remaining samples. During the process of jackknifing, both the training dataset and testing dataset are actually open, and each sample will be in turn moved between the two. The jackknife test can exclude the “memory” effect. Also, the arbitrariness problem as mentioned above for the independent dataset test and subsampling test can be avoided because the outcome obtained by the jackknife cross-validation is always unique for a given benchmark dataset. Accordingly, the jackknife test has been increasingly used and widely recognized by investigators to examine the quality of various predictors (see, e.g., [33], [41], [43], [45]–[47], [66]–[72]).

Accordingly, in this study we also used the jackknife cross-validation method to calculate the metrics in **Eq. 18** although it would take more computational time.

### 3. Comparison with Other Methods

The jackknife test results by iNitro-Tyr on the benchmark dataset 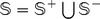 (cf. Supporting Information S1) for the four metrics defined in **Eq. 18** are listed in **Table 1**, where for facilitating comparison, the corresponding results by GPS-YNO2 [27] with different thresholds are also given.

**Table 1:** Comparison of the new iNitro-Tyr predictor with the existing predictors in identifying the nitrotyrosine sites; the rates listed below were derived by the jackknife cross-validation on the 546 source proteins used in [27].

Predictor	Threshold	Acc (%)	MCC	Sn (%)	Sp (%)
GPS-YNO2^a^	High	82.57	0.1884	28.89	90.02
	Medium	79.60	0.2171	40.53	85.02
	Low	76.51	0.2335	50.09	90.18
iNitro-Tyr^b^		84.52	0.4905	81.76	85.89

a As reported in [27], where 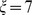, i.e., the length of the potential nitrotyrosine peptides considered is 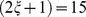.

b See Eqs. 15–16, where 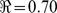 and 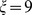, i.e., the length of the potential nitrotyrosine peptides considered is 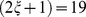.

From the table, we can see the following facts. (i) The overall accuracy by the current iNitro-Tyr predictor is 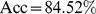, which is higher than the overall accuracy by GPS-YNO2 regardless what threshold is used for the latter. (ii) The Mathew’s correlation coefficient obtained by iNitro-Tyr is 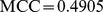, which is significantly higher than that by GPS-YNO2, indicating that the new predictor is more stable and less noisy. (iii) The sensitivity and specificity obtained by iNitro-Tyr are 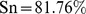 and 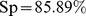, which are much more evenly distributed than those by the GPS-YNO2 predictor.

It is instructive to point out that, as shown by **Eqs. 12a** and **b**, the amino acid pairwise coupling effects [11] has been incorporated via the general form of PseAAC [8] to formulate the peptide samples. If, however, we just used the single amino acid specific position occurrence frequency to formulate the peptide samples, the corresponding prediction quality would drop down to 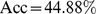 and 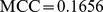, clearly indicating that consideration of the amino acid pairwise coupling effects could significantly enhance the prediction quality, fully consistent with the reports by previous investigators [73], [74], where it was observed that the prediction of protein secondary structural contents had been remarkably improved by taking into account the amino acid pairwise coupling effects.

Accordingly, compared with the best of existing predictors for identifying the nitrotyrosine sites in proteins, the new **iNitro-Tyr** predictor not only can yield higher or comparable accuracy, but is also much more stable and less noisy. It is anticipated that **iNitro-Tyr** may become a useful high throughput tool in this area, or at the very least play a complementary role to the existing predictors.

### 4. Web-Server and User Guide

For the convenience of most experimental scientists, we have established a web-server for the **iNitro-Tyr** predictor, with which users can easily get their desired results according to the steps below without the need to understand the mathematical equations in the method section.

#### Step 1

**Figure 6:**
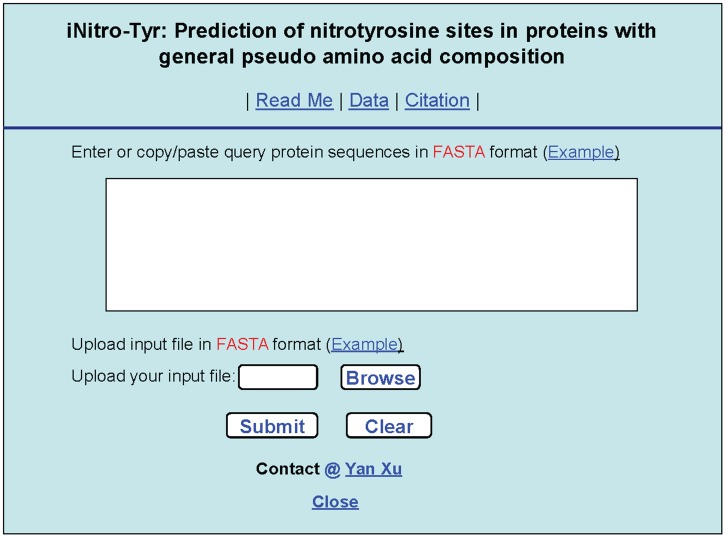
A semi-screenshot to show the top page of the iNitro-Tyr srver. Its website address is at http://app.aporc.org/iNitro-Tyr/.

Open the web server at http://app.aporc.org/iNitro-Tyr/ and you will see the top page of the predictor on your computer screen, as shown in **Fig. 6**. Click on the Read Me button to see a brief introduction about **iNitro-Tyr** predictor and the caveat when using it.

#### Step 2

Either type or copy/paste the sequences of query proteins into the input box shown at the center of **Fig. 6**. All the input sequences should be in the FASTA format. A sequence in FASTA format consists of a single initial line beginning with the symbol “>” in the first column, followed by lines of sequence data in which amino acids are represented using single-letter codes. Except for the mandatory symbol “>”, all the other characters in the single initial line are optional and only used for the purpose of identification and description. The sequence ends if another line starting with the symbol “>” appears; this indicates the start of another sequence. Example sequences in FASTA format can be seen by clicking on the Example button right above the open box. Note that if your input protein sequences should be formed by the 20 native amino acid codes (ACDEFGHIKLMNPQRSTVWY).

#### Step 3

Click on the Submit button to see the predicted results. For example, if you use the two query protein sequences in the Example window as the input, after clicking the Submit button, you will see the following on your screen. (i) The 1^st^ protein (P05181) contains 18 Y residues; of which only those located at the sequence position 71, 318, 349, 381, and 423 are of nitrotyrosine site, while all the others are of non-nitrotyrosine site. (ii) The 2nd protein (P03023) contains 8 Y residues; of which only those located at the sequence positions 7, 12, 17, and 47 belong to the nitrotyrosine site, while all the others belong to non-nitrotyrosine site. All these results are fully consistent with experimental observations except for one Y residue at the position 349 in the 1^st^ protein (P05181) that is actually non-nitrotyrosine site but was overpredicted as nitrotyrosine site.

#### Step 4

As shown on the lower panel of **Fig. 6**, you may also submit your query proteins in an input file (with FASTA format) via the “Browse” button. To see the sample of input file, click on the Example button right under the input box.

#### Step 5

Click on the Data button to download the benchmark dataset used to train and test the iNitro-Tyr predictor.

## Conclusions

As one of the important posttranslational modifications (PTMs), nitrotyrosine is a product occurring in proteins when their tyrosine (Tyr or Y) residue is nitrated. Since a remarkably increasing level of nitrotyrosine is detected for those patients who have suffered from rheumatoid arthritis, septic shock, and coeliac disease, knowledge of nitrotyrosine is very useful for developing drugs against these diseases.

A new predictor was developed for identifying the nitrotyrosine sites in proteins based on a set of 19-tuple peptides generated as follows. Sliding a window of 19 amino acids along each of the 546 protein sequences taken from a protein database, collected were only those peptide segments with Y (tyrosine) at the center, i.e., the potential nitrotyrosine-site-containing peptides. The benchmark dataset thus obtained contains 1,044 experiment-confirmed nitrotyrosine peptides and 7,669 non-nitrotyrosine peptides.

The new predictor is called iNitro-Tyr, in which each of the potential nitrotyrosine-site-containing peptides was formulated with a 18-D vector formed by incorporating the position-specific dipeptide propensity (PSDP) into the general form [8] of pseudo amino acid composition [38], [75] or Chou’s PseAAC [39], [51], [54].

It has been observed by the rigorous cross validations that the iNitro-Tyr not only yields higher success rates but also is more stable and less noisy as reflected by a set of four metrics generally used to measure the quality of a predictor from different angles.

For the convenience of most experimental scientists, the web-server of iNitro-Tyr has been established at http://app.aporc.org/iNitro-Tyr/. Furthermore, to maximize their convenience, a step-by-step guide has been provided, by which users can easily get their desired results without the need to follow the complicated mathematics that were presented in this paper just for the integrity of the predictor.

It has not escaped our notice that the current approach can also be used to develop various effective methods for identifying the sites of other PTM sites in proteins.

## Supporting Information

Supporting Information S1 **The benchmark dataset used in this study contains 8,713 peptides formed by 19 amino acid residues with Y (tyrosine) at the center.** Of these peptides, 1,044 are of nitrotyrosine and 7,669 of non-nitrotyrosine. Listed are also the codes of the source proteins from which these 19-tuple peptide sequences are derived as well as their corresponding sites in proteins. See the main text for further explanation.
(DOC)

## References

[1] Casoni F, Basso M, Massignan T, Gianazza E, Cheroni C, et al (2005) Protein nitration in a mouse model of familial amyotrophic lateral sclerosis: possible multifunctional role in the pathogenesis. J Biol Chem 280: 16295–16304. 15699043 10.1074/jbc.M413111200

[2] Ghesquiere B, Colaert N, Helsens K, Dejager L, Vanhaute C, et al (2009) In vitro and in vivo protein-bound tyrosine nitration characterized by diagonal chromatography. Mol Cell Proteomics 8: 2642–2652. 19741252 10.1074/mcp.M900259-MCP200 PMC2816017

[3] Zhan X, Du Y, Crabb JS, Gu X, Kern TS, et al (2008) Targets of tyrosine nitration in diabetic rat retina. Mol Cell Proteomics 7: 864–874. 18165258 10.1074/mcp.M700417-MCP200 PMC2401337

[4] Jarmula A, Rode W (2013) Computational study of the effects of protein tyrosine nitrations on the catalytic activity of human thymidylate synthase. J Comput Aided Mol Des 27: 45–66. 23239172 10.1007/s10822-012-9624-4

[5] Abello N, Kerstjens HA, Postma DS, Bischoff R (2009) Protein tyrosine nitration: selectivity, physicochemical and biological consequences, denitration, and proteomics methods for the identification of tyrosine-nitrated proteins. J Proteome Res 8: 3222–3238. 19415921 10.1021/pr900039c

[6] Feeney MB, Schoneich C (2013) Proteomic Approaches to Analyze Protein Tyrosine Nitration. Antioxid Redox Signal. 10.1089/ars.2012.5058 PMC3785798 23157221

[7] Dekker F, Abello N, Wisastra R, Bischoff R (2012) Enrichment and detection of tyrosine-nitrated proteins. Curr Protoc Protein Sci Chapter 14: Unit 14 13. 10.1002/0471140864.ps1413s69 22851496

[8] Chou KC (2011) Some remarks on protein attribute prediction and pseudo amino acid composition (50th Anniversary Year Review). Journal of Theoretical Biology 273: 236–247. 21168420 10.1016/j.jtbi.2010.12.024 PMC7125570

[9] Chen W, Feng PM, Lin H (2013) iRSpot-PseDNC: identify recombination spots with pseudo dinucleotide composition Nucleic Acids Research. 41: e69. 10.1093/nar/gks1450 PMC3616736 23303794

[10] Min JL, Xiao X (2013) iEzy-Drug: A web server for identifying the interaction between enzymes and drugs in cellular networking. BioMed Research International 2013: 701317. 24371828 10.1155/2013/701317 PMC3858977

[11] Xu Y, Shao XJ, Wu LY, Deng NY (2013) iSNO-AAPair: incorporating amino acid pairwise coupling into PseAAC for predicting cysteine S-nitrosylation sites in proteins. PeerJ 1: e171. 24109555 10.7717/peerj.171 PMC3792191

[12] Xiao X, Min JL, Wang P (2013) iCDI-PseFpt: Identify the channel-drug interaction in cellular networking with PseAAC and molecular fingerprints. Journal of Theoretical Biology 337C: 71–79. 10.1016/j.jtbi.2013.08.013 23988798

[13] Fan YN, Xiao X, Min JL (2014) iNR-Drug: Predicting the interaction of drugs with nuclear receptors in cellular networking. Intenational Journal of Molecular Sciences 15: 4915–4937. 10.3390/ijms15034915 PMC3975431 24651462

[14] Guo SH, Deng EZ, Xu LQ, Ding H, Lin H, et al (2014) iNuc-PseKNC: a sequence-based predictor for predicting nucleosome positioning in genomes with pseudo k-tuple nucleotide composition. Bioinformatics 30: 1522–1529. 24504871 10.1093/bioinformatics/btu083

[15] Liu B, Zhang D, Xu R, Xu J, Wang X, et al (2014) Combining evolutionary information extracted from frequency profiles with sequence-based kernels for protein remote homology detection. Bioinformatics 30: 472–479. 24318998 10.1093/bioinformatics/btt709 PMC7537947

[16] Qiu WR, Xiao X (2014) iRSpot-TNCPseAAC: Identify recombination spots with trinucleotide composition and pseudo amino acid components. Int J Mol Sci 15: 1746–1766. 24469313 10.3390/ijms15021746 PMC3958819

[17] Xu Y, Wen X, Shao XJ, Deng NY (2014) iHyd-PseAAC: Predicting hydroxyproline and hydroxylysine in proteins by incorporating dipeptide position-specific propensity into pseudo amino acid composition. International Journal of Molecular Sciences 15: 7594–7610. 24857907 10.3390/ijms15057594 PMC4057693

[18] Ding H, Deng EZ, Yuan LF, Liu L, Lin H, et al (2014) iCTX-Type: A sequence-based predictor for identifying the types of conotoxins in targeting ion channels. BioMed Research International 2014: 286419. 24991545 10.1155/2014/286419 PMC4058692

[19] Qiu WR, Xiao X, Lin WZ (2014) iMethyl-PseAAC: Identification of protein methylation sites via a pseudo amino acid composition approach. BioMed Research International 2014: ID 947416. 10.1155/2014/947416 PMC4054830 24977164

[20] Chen W, Feng PM, Lin H (2014) iSS-PseDNC: identifying splicing sites using pseudo dinucleotide composition. Biomed Research International 2014: 623149. 24967386 10.1155/2014/623149 PMC4055483

[21] Chen W, Feng PM, Deng EZ, Lin H (2014) iTIS-PseTNC: a sequence-based predictor for identifying translation initiation site in human genes using pseudo trinucleotide composition. Analytical Biochemistry 10.1016/j.ab.2014.1006.1022. 10.1016/j.ab.2014.06.022 25016190

[22] Chou KC (1993) A vectorized sequence-coupling model for predicting HIV protease cleavage sites in proteins. Journal of Biological Chemistry 268: 16938–16948. 8349584

[23] Chou KC (1996) Review: Prediction of human immunodeficiency virus protease cleavage sites in proteins. Analytical Biochemistry 233: 1–14. 8789141 10.1006/abio.1996.0001

[24] Chou KC (1995) A sequence-coupled vector-projection model for predicting the specificity of GalNAc-transferase. Protein Science 4: 1365–1383. 7670379 10.1002/pro.5560040712 PMC2143175

[25] Chou KC (2001) Prediction of signal peptides using scaled window. Peptides 22: 1973–1979. 11786179 10.1016/s0196-9781(01)00540-x

[26] Chou KC, Shen HB (2007) Review: Recent progresses in protein subcellular location prediction. Analytical Biochemistry 370: 1–16. 17698024 10.1016/j.ab.2007.07.006

[27] Liu Z, Cao J, Ma Q, Gao X, Ren J, et al (2011) GPS-YNO2: computational prediction of tyrosine nitration sites in proteins. Mol Biosyst 7: 1197–1204. 21258675 10.1039/c0mb00279h

[28] Xu Y, Ding J, Wu LY (2013) iSNO-PseAAC: Predict cysteine S-nitrosylation sites in proteins by incorporating position specific amino acid propensity into pseudo amino acid composition PLoS ONE. 8: e55844. 10.1371/journal.pone.0055844 PMC3567014 23409062

[29] Chou JJ (1993) A formulation for correlating properties of peptides and its application to predicting human immunodeficiency virus protease-cleavable sites in proteins. Biopolymers 33: 1405–1414. 8400033 10.1002/bip.360330910

[30] Wang M, Yang J, Xu ZJ (2005) SLLE for predicting membrane protein types. Journal of Theoretical Biology 232: 7–15. 15498588 10.1016/j.jtbi.2004.07.023

[31] Feng KY, Cai YD (2005) Boosting classifier for predicting protein domain structural class. Biochemical & Biophysical Research Communications 334: 213–217. 15993842 10.1016/j.bbrc.2005.06.075

[32] Feng PM, Chen W, Lin H (2013) iHSP-PseRAAAC: Identifying the heat shock protein families using pseudo reduced amino acid alphabet composition. Analytical Biochemistry 442: 118–125. 23756733 10.1016/j.ab.2013.05.024

[33] Kandaswamy KK, Martinetz T, Moller S, Suganthan PN, et al (2011) AFP-Pred: A random forest approach for predicting antifreeze proteins from sequence-derived properties. Journal of Theoretical Biology 270: 56–62. 21056045 10.1016/j.jtbi.2010.10.037

[34] Chou KC, Shen HB (2006) Predicting eukaryotic protein subcellular location by fusing optimized evidence-theoretic K-nearest neighbor classifiers. Journal of Proteome Research 5: 1888–1897. 16889410 10.1021/pr060167c

[35] Shen HB (2009) A top-down approach to enhance the power of predicting human protein subcellular localization: Hum-mPLoc 2.0. Analytical Biochemistry 394: 269–274. 19651102 10.1016/j.ab.2009.07.046

[36] Xiao X, Min JL, Wang P (2013) iGPCR-Drug: A web server for predicting interaction between GPCRs and drugs in cellular networking. PLoS ONE 8: e72234. 24015221 10.1371/journal.pone.0072234 PMC3754978

[37] Chou KC (2013) Some Remarks on Predicting Multi-Label Attributes in Molecular Biosystems. Molecular Biosystems 9: 1092–1100. 23536215 10.1039/c3mb25555g

[38] Chou KC (2001) Prediction of protein cellular attributes using pseudo amino acid composition. PROTEINS: Structure, Function, and Genetics (Erratum: ibid, 2001, Vol44, 60) 43: 246–255. 10.1002/prot.1035 11288174

[39] Lin SX, Lapointe J (2013) Theoretical and experimental biology in one. J Biomedical Science and Engineering (JBiSE) 6: 435–442.

[40] Nanni L, Lumini A, Gupta D, Garg A (2012) Identifying Bacterial Virulent Proteins by Fusing a Set of Classifiers Based on Variants of Chou’s Pseudo Amino Acid Composition and on Evolutionary Information. IEEE/ACM Trans Comput Biol Bioinform 9: 467–475. 21860064 10.1109/TCBB.2011.117

[41] Hajisharifi Z, Piryaiee M, Mohammad Beigi M, Behbahani M, Mohabatkar H (2014) Predicting anticancer peptides with Chou’s pseudo amino acid composition and investigating their mutagenicity via Ames test. Journal of Theoretical Biology 341: 34–40. 24035842 10.1016/j.jtbi.2013.08.037

[42] Mei S (2012) Predicting plant protein subcellular multi-localization by Chou’s PseAAC formulation based multi-label homolog knowledge transfer learning. Journal of Theoretical Biology 310: 80–87. 22750634 10.1016/j.jtbi.2012.06.028

[43] Chen YK, Li KB (2013) Predicting membrane protein types by incorporating protein topology, domains, signal peptides, and physicochemical properties into the general form of Chou’s pseudo amino acid composition. Journal of Theoretical Biology 318: 1–12. 23137835 10.1016/j.jtbi.2012.10.033

[44] Georgiou DN, Karakasidis TE, Megaritis AC (2013) A short survey on genetic sequences, Chou’s pseudo amino acid composition and its combination with fuzzy set theory. The Open Bioinformatics Journal 7: 41–48.

[45] Mohabatkar H, Mohammad Beigi M, Esmaeili A (2011) Prediction of GABA(A) receptor proteins using the concept of Chou’s pseudo-amino acid composition and support vector machine. Journal of Theoretical Biology 281: 18–23. 21536049 10.1016/j.jtbi.2011.04.017

[46] Khosravian M, Faramarzi FK, Beigi MM, Behbahani M, Mohabatkar H (2013) Predicting Antibacterial Peptides by the Concept of Chou’s Pseudo-amino Acid Composition and Machine Learning Methods. Protein & Peptide Letters 20: 180–186. 22894156 10.2174/092986613804725307

[47] Mohabatkar H, Beigi MM, Abdolahi K, Mohsenzadeh S (2013) Prediction of Allergenic Proteins by Means of the Concept of Chou’s Pseudo Amino Acid Composition and a Machine Learning Approach. Medicinal Chemistry 9: 133–137. 22931491 10.2174/157340613804488341

[48] Mohammad Beigi M, Behjati M, Mohabatkar H (2011) Prediction of metalloproteinase family based on the concept of Chou’s pseudo amino acid composition using a machine learning approach. Journal of Structural and Functional Genomics 12: 191–197. 22143437 10.1007/s10969-011-9120-4

[49] Zia Ur R, Khan A (2012) Identifying GPCRs and their Types with Chou’s Pseudo Amino Acid Composition: An Approach from Multi-scale Energy Representation and Position Specific Scoring Matrix. Protein & Peptide Letters 19: 890–903. 22316312 10.2174/092986612801619589

[50] Sun XY, Shi SP, Qiu JD, Suo SB, Huang SY, et al (2012) Identifying protein quaternary structural attributes by incorporating physicochemical properties into the general form of Chou’s PseAAC via discrete wavelet transform. Molecular BioSystems 8: 3178–3184. 22990717 10.1039/c2mb25280e

[51] Du P, Gu S, Jiao Y (2014) PseAAC-General: Fast building various modes of general form of Chou’s pseudo-amino acid composition for large-scale protein datasets. International Journal of Molecular Sciences 15: 3495–3506. 24577312 10.3390/ijms15033495 PMC3975349

[52] Jiang Y, Huang T, Chen L, Gao YF, Cai Y, et al (2013) Signal propagation in protein interaction network during colorectal cancer progression. Biomed Res Int 2013: 287019. 23586028 10.1155/2013/287019 PMC3615629

[53] Du P, Wang X, Xu C, Gao Y (2012) PseAAC-Builder: A cross-platform stand-alone program for generating various special Chou’s pseudo-amino acid compositions. Analytical Biochemistry 425: 117–119. 22459120 10.1016/j.ab.2012.03.015

[54] Cao DS, Xu QS, Liang YZ (2013) propy: a tool to generate various modes of Chou’s PseAAC. Bioinformatics 29: 960–962. 23426256 10.1093/bioinformatics/btt072

[55] Chou KC (2001) Using subsite coupling to predict signal peptides. Protein Engineering 14: 75–79. 11297664 10.1093/protein/14.2.75

[56] Chou KC, Shen HB (2009) Review: recent advances in developing web-servers for predicting protein attributes. Natural Science 2: 63–92.

[57] Chou KC (2001) Prediction of protein signal sequences and their cleavage sites. PROTEINS: Structure, Function, and Genetics 42: 136–139. 10.1002/1097-0134(20010101)42:1<136::aid-prot130>3.0.co;2-f 11093267

[58] Chou KC, Wu ZC, Xiao X (2012) iLoc-Hum: Using accumulation-label scale to predict subcellular locations of human proteins with both single and multiple sites. Molecular Biosystems 8: 629–641. 22134333 10.1039/c1mb05420a

[59] Xiao X, Wu ZC (2011) iLoc-Virus: A multi-label learning classifier for identifying the subcellular localization of virus proteins with both single and multiple sites. Journal of Theoretical Biology 284: 42–51. 21684290 10.1016/j.jtbi.2011.06.005

[60] Wu ZC, Xiao X (2011) iLoc-Plant: a multi-label classifier for predicting the subcellular localization of plant proteins with both single and multiple sites. Molecular BioSystems 7: 3287–3297. 21984117 10.1039/c1mb05232b

[61] Lin WZ, Fang JA, Xiao X (2013) iLoc-Animal: A multi-label learning classifier for predicting subcellular localization of animal proteins Molecular BioSystems. 9: 634–644. 10.1039/c3mb25466f 23370050

[62] Chou KC, Wu ZC, Xiao X (2011) iLoc-Euk: A Multi-Label Classifier for Predicting the Subcellular Localization of Singleplex and Multiplex Eukaryotic Proteins. PLoS One 6: e18258. 21483473 10.1371/journal.pone.0018258 PMC3068162

[63] Xiao X, Wang P, Lin WZ, Jia JH (2013) iAMP-2L: A two-level multi-label classifier for identifying antimicrobial peptides and their functional types. Analytical Biochemistry 436: 168–177. 23395824 10.1016/j.ab.2013.01.019

[64] Chou KC, Zhang CT (1995) Review: Prediction of protein structural classes. Critical Reviews in Biochemistry and Molecular Biology 30: 275–349. 7587280 10.3109/10409239509083488

[65] Chou KC, Shen HB (2008) Cell-PLoc: A package of Web servers for predicting subcellular localization of proteins in various organisms. Nature Protocols 3: 153–162. 18274516 10.1038/nprot.2007.494

[66] Shen HB, Yang J, Liu XJ (2005) Using supervised fuzzy clustering to predict protein structural classes. Biochem Biophys Res Commun 334: 577–581. 16023077 10.1016/j.bbrc.2005.06.128

[67] Mei S (2012) Multi-kernel transfer learning based on Chou’s PseAAC formulation for protein submitochondria localization. Journal of Theoretical Biology 293: 121–130. 22037046 10.1016/j.jtbi.2011.10.015

[68] Chen W, Lin H, Feng PM, Ding C, Zuo YC, et al (2012) iNuc-PhysChem: A Sequence-Based Predictor for Identifying Nucleosomes via Physicochemical Properties. PLoS ONE 7: e47843. 23144709 10.1371/journal.pone.0047843 PMC3483203

[69] Sahu SS, Panda G (2010) A novel feature representation method based on Chou’s pseudo amino acid composition for protein structural class prediction. Computational Biology and Chemistry 34: 320–327. 21106461 10.1016/j.compbiolchem.2010.09.002

[70] Huang C, Yuan JQ (2013) Predicting protein subchloroplast locations with both single and multiple sites via three different modes of Chou’s pseudo amino acid compositions. Journal of Theoretical Biology 335: 205–212. 23850480 10.1016/j.jtbi.2013.06.034

[71] Kong L, Zhang L, Lv J (2014) Accurate prediction of protein structural classes by incorporating predicted secondary structure information into the general form of Chou’s pseudo amino acid composition. J Theor Biol 344: 12–18. 24316044 10.1016/j.jtbi.2013.11.021

[72] Jia C, Lin X, Wang Z (2014) Prediction of Protein S-Nitrosylation Sites Based on Adapted Normal Distribution Bi-Profile Bayes and Chou’s Pseudo Amino Acid Composition. Int J Mol Sci 15: 10410–10423. 24918295 10.3390/ijms150610410 PMC4100159

[73] Liu W (1999) Protein secondary structural content prediction. Protein Engineering 12: 1041–1050. 10611397 10.1093/protein/12.12.1041

[74] Chou KC (1999) Using pair-coupled amino acid composition to predict protein secondary structure content. Journal of Protein Chemistry 18: 473–480. 10449044 10.1023/a:1020696810938

[75] Chou KC (2005) Using amphiphilic pseudo amino acid composition to predict enzyme subfamily classes. Bioinformatics 21: 10–19. 15308540 10.1093/bioinformatics/bth466

[76] Chou KC (2002) Review: Prediction of protein signal sequences. Current Protein and Peptide Science 3: 615–622. 12470215 10.2174/1389203023380468

[77] Chou KC, Shen HB (2007) Signal-CF: a subsite-coupled and window-fusing approach for predicting signal peptides. Biochem Biophys Res Comm 357: 633–640. 17434148 10.1016/j.bbrc.2007.03.162

