# Supplementary material for: iNitro-Tyr: Prediction of Nitrotyrosine Sites in Proteins with General Pseudo Amino Acid Composition
Source: PLoS One. 2014 Aug 14;9(8):e105018. doi: 10.1371/journal.pone.0105018 (PMC4133382; doi:10.1371/journal.pone.0105018)
Supplement: Supporting Information S1 — The benchmark dataset used in this study contains 8,713 peptides formed by 19 amino acid residues with Y (tyrosine) at the center. Of these peptides, 1,044 are of nitrotyrosine and 7,669 of non-nitrotyrosine. Listed are also the codes of the source proteins from which these 19-tuple peptide sequences are derived as well as their corresponding sites in proteins. See the main text for further explanation. (DOC) [file pone.0105018.s001.doc]

**Supporting Information S1**. The benchmark dataset used in this study contains 8,713 peptides formed by 19 amino acid residues with Y (tyrosine) at the center. Of these peptides, 1,044 are of nitrotyrosine and 7,669 of non-nitrotyrosine. Listed are also the codes of the source proteins from which these 19-tuple peptide sequences are derived as well as their corresponding sites in proteins. See the main text for further explanation.

**I. Positive dataset contains 1,044 samples**

| Uniprot ID | Site | Sequence |
| --- | --- | --- |
| Q9L422  P00781  P00781  P00781  P00781  P00781  P00781  P00781  P00781  P00781  P00781  P00781  P00781  P07999  P07999  P14779  P08877  P08877  P68735  P68735  P00257  P00366  P00366  P00366  P00366  P00366  P00442  P00730  P00730  P00766  P00766  P01180  P02663  P02666  P02754  P02769  P02769  P02769  P02769  P02769  P02769  P02769  P02769  P02769  P02769  P02769  P02769  P02769  P02769  P02769  P02769  P02769  P02769  P02769  P02769  P11151  P11151  P11151  P11151  P11151  P11151  P11151  P11151  P17694  P17694  P23709  P31039  P31039  P48305  P48305  P48305  P48305  P48305  P61625  P61823  P61823  P61823  Q02366  Q9N2I2  P60615  P60615  P00294  P00784  P00784  P00784  P00784  Q60468  Q60468  Q60468  Q60468  Q60468  Q60468  P69542  P69542  P69542  P03040  P03040  P13332  P13332  P13332  P13332  P13332  P13332  P13332  P13332  P13332  P13332  P13332  P13332  P13332  P07515  P00004  P00004  P00004  P00004  P01245  P01245  P01245  P01245  P01245  P01245  P01245  P03023  P03023  P03023  P03023  P06968  P0A6A8  P0A786  P0A881  P0A881  P0A9C5  P0A9C5  P0A9C5  P0A9C5  P0A9C5  P0A9C5  P0A9C5  P0A9C5  P0AGD3  P12758  P33341  P42914  P45848  P68699  P68699  P69924  P69924  P69924  P69924  Q8X1D8  P00698  P00698  P00698  P02701  P02945  P02945  P02945  P02945  P01050  P01050  P00338  P00338  P00338  P00390  P00390  P00558  P00558  P00558  P00709  P00709  P00734  P00747  P00747  P00749  P01009  P01009  P01009  P01009  P01009  P01009  P01024  P01024  P01031  P01112  P01112  P01112  P01112  P01112  P01857  P01857  P02462  P02462  P02647  P02647  P02647  P02647  P02647  P02675  P02675  P04114  P04114  P04114  P04114  P04114  P04114  P04114  P04114  P04114  P04114  P04114  P04114  P04114  P04114  P04114  P04179  P04179  P04179  P04637  P05141  P05181  P05181  P05181  P05181  P05230  P05230  P05230  P05230  P05230  P06576  P06576  P06576  P06576  P06576  P06576  P06576  P06576  P06748  P07195  P07195  P07311  P07339  P07355  P07737  P07737  P07900  P07900  P07900  P07900  P07910  P07954  P07954  P08133  P08572  P08572  P08590  P08590  P08670  P08670  P08670  P09211  P09237  P09429  P10145  P10153  P10276  P10276  P10276  P10768  P10768  P10809  P10809  P11142  P11142  P11142  P11142  P11142  P11142  P11413  P11586  P11586  P11678  P12004  P12724  P13010  P13073  P13639  P13639  P13639  P13796  P13929  P13929  P13929  P13929  P13929  P13929  P13929  P13929  P13929  P13929  P13929  P14618  P14618  P14625  P14649  P14902  P14902  P14902  P15311  P15880  P16284  P16401  P18669  P18669  P18669  P19338  P19338  P19652  P19652  P19652  P19652  P19652  P19652  P19652  P19652  P19784  P20618  P20700  P20700  P20700  P20813  P20813  P20813  P20813  P20813  P20813  P20813  P20813  P20813  P20813  P21333  P21333  P22102  P22234  P23193  P23193  P23246  P23528  P23528  P24071  P24666  P25205  P25205  P25705  P25705  P25705  P25963  P25963  P26038  P26599  P26599  P26639  P26639  P27361  P27797  P27816  P27824  P28072  P29372  P29474  P29474  P29474  P29474  P29474  P29474  P29474  P29474  P29474  P29474  P29474  P29474  P29474  P29474  P29474  P29474  P29474  P29474  P29474  P29692  P30086  P30086  P30101  P30101  P31948  P32969  P33993  P34913  P34913  P34932  P35080  P35228  P35228  P35228  P35228  P35247  P35247  P35247  P35250  P35520  P35606  P35637  P35998  P36578  P36578  P36776  P36959  P37802  P37837  P37840  P37840  P37840  P37840  P38159  P38646  P38646  P40926  P40926  P40926  P41252  P42704  P43243  P45452  P46459  P46779  P47813  P49207  P49321  P49368  P49368  P49458  P49588  P50395  P50395  P50990  P50990  P50991  P50991  P51149  P52272  P52566  P53041  P53396  P53396  P53396  P55072  P55769  P55884  P60468  P60842  P60842  P61088  P61158  P61221  P61221  P61247  P61604  P61927  P61978  P61978  P62081  P62136  P62158  P62158  P62195  P62241  P62241  P62249  P62263  P62273  P62280  P62701  P62750  P62750  P62805  P62805  P62805  P62826  P62910  P62917  P62993  P63220  P63244  P67775  P68104  P68104  P68871  P68871  P69905  P69905  P69905  P78344  P78371  P78417  P83731  P84103  Q00325  Q00610  Q00610  Q00610  Q00610  Q00839  Q01130  Q01518  Q02543  Q02790  Q02878  Q03405  Q03405  Q03405  Q03405  Q03405  Q03405  Q04206  Q04206  Q04837  Q06547  Q06830  Q07955  Q07955  Q07955  Q08170  Q08211  Q08J23  Q09028  Q12906  Q12996  Q12996  Q13148  Q13237  Q13257  Q13263  Q13263  Q13347  Q13451  Q13547  Q13573  Q13765  Q13813  Q13813  Q13813  Q13813  Q13813  Q13813  Q13936  Q13976  Q13976  Q14204  Q14204  Q14566  Q14683  Q14683  Q14694  Q14847  Q15019  Q15056  Q15056  Q15056  Q15181  Q15181  Q15365  Q15370  Q15393  Q15427  Q15428  Q15428  Q15459  Q15459  Q16595  Q16595  Q16595  Q16629  Q16647  Q16695  Q16891  Q3MHD2  Q52LJ0  Q58FF8  Q58FF8  Q58FF8  Q5JTV8  Q6P2Q9  Q71U36  Q71U36  Q71U36  Q71U36  Q71U36  Q71U36  Q71U36  Q71U36  Q71U36  Q71U36  Q71UM5  Q7KZF4  Q7L014  Q86VP6  Q8IWL2  Q8IWL2  Q8IWL2  Q8IWL2  Q8IXH7  Q8N684  Q8NHL6  Q8NHL6  Q8NHL6  Q8NHL6  Q8NHL6  Q8NHL6  Q8NHL6  Q92526  Q92900  Q92922  Q96AE4  Q96IX5  Q96PK6  Q96QR8  Q99614  Q99727  Q99727  Q99727  Q99727  Q99832  Q9BUJ2  Q9BWJ5  Q9BZK7  Q9H074  Q9H0D6  Q9H3K2  Q9H3K6  Q9NPD3  Q9NR30  Q9NR45  Q9NUV9  Q9NYF8  Q9NZ32  Q9P2J5  Q9UHB9  Q9UHD1  Q9UI10  Q9UKK9  Q9UKY7  Q9UL46  Q9UN86  Q9UNZ2  Q9Y230  Q9Y285  Q9Y2W1  Q9Y2W2  Q9Y2W2  Q9Y314  Q9Y3F4  Q9Y3U8  Q9Y6A4  O94892  O95470  O95470  P25787  P31321  P50502  P50502  P61513  Q13017  Q15027  Q15413  Q8IUC4  Q9UHA7  O00151  O00165  O00232  O00483  O14737  O14744  O14976  O14979  O43143  O43252  O43290  O43615  O43660  O43684  O43684  O43709  O43719  O43765  O43776  O43776  O43852  O60256  O60506  O60641  O60884  O75083  O75347  O75390  O75390  O75396  O75792  O75832  O75947  O75947  O94903  O95466  P01654  P01864  P04370  P04370  P05064  P05064  P05201  P05202  P05202  P05202  P07724  P07724  P07724  P08551  P08551  P08551  P08551  P08551  P08553  P10649  P10649  P10649  P10649  P10649  P10649  P10649  P10649  P10853  P10853  P10853  P14094  P14206  P15105  P15105  P15105  P16858  P16858  P16858  P17183  P17183  P17183  P18531  P18531  P18872  P19246  P19783  P21803  P30275  P30275  P35441  P35441  P39053  P39053  P39053  P40142  P41588  P50114  P50153  P50516  P56387  P58281  P60202  P60710  P60710  P60710  P60710  P60710  P60710  P60710  P60710  P60710  P60710  P62259  P62259  P62259  P62259  P62259  P62814  P62814  P63168  P63328  P67778  P70338  P85094  P97427  P97427  P98063  Q04447  Q04447  Q08122  Q11011  Q3ULF4  Q3UYC0  Q60865  Q61644  Q61656  Q6I6G8  Q6PIC6  Q6PIC6  Q6ZQ93  Q7TMM9  Q7TMM9  Q7TMM9  Q7TMM9  Q80VM8  Q80YV4  Q8BH59  Q8BN78  Q8BN78  Q8BUE4  Q8BVE3  Q8CJG0  Q8K0T0  Q8QZT1  Q8R5C5  Q91VD9  Q91YM2  Q920Q4  Q99JX7  Q99LC5  Q99LC5  Q9CQV8  Q9CQV8  Q9CQV8  Q9CZC8  Q9D0K2  Q9D0K2  Q9D6J6  Q9D855  Q9D974  Q9DB20  Q9DBG3  Q9JIS5  Q9NRM2  Q9NRM2  Q9QUH6  Q9QWH1  Q9QYG0  Q9Z188  B2RSH2  O08530  O08553  O08553  O08585  O35633  O35643  O35652  O35678  O88990  P60301  P60301  P60301  P60770  P60770  P01448  P01448  P01448  P01426  P00369  P01387  P01387  P00187  P00187  P00187  P00187  P00187  P00489  P00489  P00489  P00489  P00489  P00489  P00489  P00489  P00489  P00489  P00489  P00489  P00489  P00489  P00489  P00489  P00489  P00489  P00489  P00489  P00489  P00489  P00489  P00489  P00489  P00489  P00489  P00883  P00883  P00883  P00883  P00883  P00883  P01870  P02602  P02602  P02602  P46406  P46406  P01218  P01218  P01218  P01218  P01240  P01240  P01240  P01240  P01240  P05979  P05979  P05979  P16038  Q51918  Q51918  Q51918  P02244  P02244  P02244  P02185  P02185  P02185  P00183  P00183  P00183  P00183  P00183  P00564  P00564  P00564  P00773  P02564  P02564  P04177  P04177  P04177  P04692  P04764  P04764  P07825  P08011  P08011  P10066  P18163  P18163  P19357  P21807  P21807  P32198  P32198  P35570  P35570  P35570  P35570  P35570  P48548  P52504  P56574  P70623  P98158  Q62673  Q62886  Q63269  Q63544  Q64455  Q91YE5  Q923K9  Q9CR40  Q9JKL9  Q9QYP2  Q07008  Q07008  Q07008  Q9QXQ0  O15229  O54922  O88457  O88457  O88943  O24819  P00924  P00924  P04806  P04806  P04806  P04806  P32178  P35809  P00644  P00807  P0A0E3  P0A0E3  P01006  P01006  P15444  P00592  P00592  P01315  P01315  P01315  P01315  P02703  P02703  P02703  P09955  P09955  P09955  P09955  P09955  P09955  P09955  P16276  P16276  P16276  P61013  Q5SIY4  P69687  P06859  P06859  P06859  P11507  P11507  P11507  P11507  P11507  P11507  P11507  P11507 | 51  21  56  90  103  142  166  205  208  213  237  261  262  160  254  335  29  37  242  379  140  319  458  464  528  550  109  308  358  146  171  80  199  208  115  54  108  161  163  171  173  179  180  184  286  342  355  357  364  376  393  424  434  475  520  103  124  130  194  318  341  346  455  47  53  247  56  142  6  47  51  52  129  1064  99  102  141  123  298  45  75  83  137  194  236  249  128  162  163  218  225  226  26  41  56  26  51  64  74  226  255  271  305  456  461  494  533  536  570  591  37  49  68  75  98  54  61  68  135  167  184  200  7  12  17  47  92  72  241  7  30  101  115  180  288  297  327  335  398  35  169  734  139  70  10  73  3  63  123  290  398  38  41  71  57  39  77  144  146  3  63  10  83  239  150  158  76  161  196  37  122  553  416  425  44  62  162  184  211  268  321  686  730  700  4  40  96  137  157  161  202  348  534  42  53  190  216  260  322  452  103  144  276  413  666  720  2524  3139  3295  3489  3771  4088  4116  4141  4211  58  69  217  327  195  71  318  381  423  30  70  109  112  140  196  230  247  269  331  395  418  508  67  84  240  92  232  30  60  129  197  216  313  627  139  54  270  218  327  1081  73  85  11  30  276  50  167  78  40  60  208  277  362  202  211  243  503  41  115  134  149  183  371  401  52  258  488  60  60  295  38  434  443  671  598  44  57  131  189  200  236  252  257  280  287  407  105  148  677  86  15  345  353  424  266  713  74  50  92  142  351  525  45  55  68  96  109  133  145  175  13  158  82  121  482  111  190  203  235  244  268  348  354  380  484  373  1308  417  22  126  273  691  68  140  223  88  147  456  337  440  8  181  305  85  247  430  252  298  156  285  1001  70  59  162  81  163  210  331  357  373  410  524  534  556  609  657  793  939  940  962  973  1087  1155  26  64  106  67  100  354  180  21  383  466  336  99  299  336  446  698  248  326  334  322  223  794  468  4  184  277  473  318  192  206  39  125  133  136  304  196  652  56  80  161  409  1321  202  338  294  77  106  13  540  247  359  51  690  111  229  340  468  269  282  28  64  24  434  213  227  384  644  11  449  59  70  197  76  16  155  594  155  88  27  72  323  177  255  100  139  121  83  188  115  72  34  55  82  117  144  52  73  89  147  95  133  209  12  52  284  29  254  131  146  25  43  141  751  213  108  11  32  196  377  883  921  1237  525  3  419  110  202  240  79  109  114  171  217  258  66  152  73  126  194  37  170  189  132  200  646  154  578  13  159  155  354  199  242  517  308  218  87  448  120  72  942  976  1261  1517  2423  2217  345  549  627  3641  598  105  575  615  257  129  12  86  101  17  169  183  45  1029  16  54  155  44  456  143  175  205  14  430  42  33  8  25  56  198  260  203  1204  83  103  108  161  224  262  272  282  357  451  31  329  1020  723  181  184  186  240  56  408  58  99  100  122  252  378  406  250  946  66  438  10  273  254  188  114  188  190  195  222  729  18  13  386  764  308  7  12  434  71  290  322  4  336  364  292  147  36  95  187  29  167  430  292  710  124  173  28  114  5  184  41  356  366  229  21  185  213  37  550  485  2577  258  96  321  132  174  62  73  179  1284  295  323  537  390  201  77  169  207  272  327  195  45  489  275  52  373  237  54  398  94  185  345  33  172  138  86  115  69  90  90  292  146  199  5  174  71  67  96  316  60  365  425  18  139  178  266  369  148  7  23  28  33  62  79  138  161  38  41  43  205  139  336  349  371  92  253  316  44  252  270  51  52  69  358  44  656  116  313  1120  1126  125  265  354  275  159  18  44  601  4  808  207  53  69  91  169  188  198  218  240  294  362  9  49  85  131  214  68  504  50  224  249  256  61  290  316  616  39  269  40  465  505  262  152  437  97  208  45  532  2286  36  51  106  222  374  320  302  491  492  252  417  2  753  328  4  636  477  4  125  105  235  84  106  213  293  43  115  69  56  82  35  707  511  99  102  505  593  68  129  167  19  182  431  146  186  574  153  58  292  32  43  72  46  56  22  25  51  24  169  4  22  72  189  244  272  367  75  76  84  85  91  114  162  186  204  227  234  263  281  298  405  473  512  525  549  554  614  649  727  778  781  792  821  204  223  244  328  343  364  154  60  127  182  312  318  45  65  116  117  74  177  199  215  225  355  385  417  84  359  361  372  19  68  71  104  147  152  97  180  202  204  306  14  20  39  53  134  142  423  428  432  214  131  257  250  93  154  17  9  86  56  17  376  282  589  489  939  999  1010  1172  53  106  311  20  4407  481  289  352  39  1034  1882  83  322  214  465  1175  1205  1905  260  37  409  5  6  226  116  191  259  308  309  340  346  234  97  197  96  6  37  38  106  35  91  145  40  50  101  106  72  75  76  197  306  316  348  356  367  385  71  151  472  6  36  140  80  82  118  122  130  294  295  497  586  753  990 | QTERYRGSEYTVEFLQKLK  KADKVQAQGYKGANVKVGI  GASFVSGESYNTDGNGHGT  LGVAPNVSLYAIKVLNSSG  VLNSSGSGTYSAIVSGIEW  ALKQAVDKAYASGIVVVAA  SSGSQNTIGYPAKYDSVIA  EVMAPGVSVYSTYPSNTYT  APGVSVYSTYPSNTYTSLN  VYSTYPSNTYTSLNGTSMA  GAAALILSKYPTLSASQVR  TATNLGDSFYYGKGLINVE  ATNLGDSFYYGKGLINVEA  KIPWPLFVHYAASKGGMKL  LFADGGMTQYPSFQAGRGX  WPTAPAFSLYAKEDTVLGG  TVLVQTASKYDADVNLEYN  KYDADVNLEYNGKTVNLKS  GATVPLNISYEGGKYVLRD  VTQETANLIYENQPGALNE  EENDMLDLAYGLTDRSRLG  GNVGLHSMRYLHRFGAKCI  VSYGRLTFKYERDSNYHLL  TFKYERDSNYHLLMSVQES  RQIMRTAMKYNLGLDLRTA  NAIEKVFRVYNEAGVTFTX  DPLISLSGEYSIIGRTMVV  FKAFLSIHSYSQLLLYPYG  KYGSIITTIYQASGGSIDW  VTTGWGLTRYTNANTPDRL  LLSNTNCKKYWGTKIKDAM  EALRCQEENYLPSPCQSGQ  ALPQYLKTVYQHQKAMKPW  DMPIQAFLLYQEPVLGPVR  NKVLVLDTDYKKYLLFCME  GLVLIAFSQYLQQCPFDEH  CKVASLRETYGDMADCCEK  ADEKKFWGKYLYEIARRHP  EKKFWGKYLYEIARRHPYF  LYEIARRHPYFYAPELLYY  EIARRHPYFYAPELLYYAN  PYFYAPELLYYANKYNGVF  YFYAPELLYYANKYNGVFQ  PELLYYANKYNGVFQECCQ  ADDRADLAKYICDNQDTIS  AEDKDVCKNYQEAKDAFLG  KDAFLGSFLYEYSRRHPEY  AFLGSFLYEYSRRHPEYAV  YEYSRRHPEYAVSVLLRLA  SVLLRLAKEYEATLEECCA  CAKDDPHACYSTVFDKLKH  CDQFEKLGEYGFQNALIVR  GFQNALIVRYTRKVPQVST  SERMPCTEDYLSLILNRLC  FSALTPDETYVPKAFDEKL  WVPKLVAALYKREPDSNVI  DWLSRAQQHYPVSAGYTKL  QQHYPVSAGYTKLVGQDVA  LDPAGPNFEYAEAPSRLSP  RKNRCNNMGYEINKVRAKR  YLKTRSQMPYKVFHYQVKI  SQMPYKVFHYQVKIHFSGT  IFCSREKMSYLQKGKSPVI  QPDVEWAEQYGGAVMYPTK  AEQYGGAVMYPTKETAHWK  NSPWEAFPAYRQPPESLKL  KVSDAISAQYPVVDHEFDA  WLGDQDAIHYMTEQAPASV  XXXXMSFPKYEASRLSSLP  AIRSRLKREYQLQYYDPSR  RLKREYQLQYYDPSRRGVI  LKREYQLQYYDPSRRGVIE  KLDRTFNISYXXXXXXXXX  FNSSKHFHLYGRNASMAQV  ACKNGQTNCYQSYSTMSIT  NGQTNCYQSYSTMSITDCR  IIVACEGNPYVPVHFDASV  RPKDFLSKFYVGHDPXXXX  MPMKRRLELYLPKFSIEGS  TCPPGENLCYRKMWCDAFC  AATCPSKKPYEEVTCCSTD  LTEAGTYSFYCAPHQGAGM  NDGDVNIPEYVDWRQKGAV  ELLDCDRRSYGCNGGYPWS  YCRSREKGPYAAKTDGVRQ  TDGVRQVQPYNEGALLYSI  GAVVGGLGGYMLGSAMSRP  NMNRYPNQVYYRPVDQYNN  MNRYPNQVYYRPVDQYNNQ  VVEQMCVTQYQKESQAYYD  TQYQKESQAYYDGRRSSAV  QYQKESQAYYDGRRSSAVL  SGVSRQGKPYSLNEQLCYV  LCYVDLGNEYPVLVKITLD  ITLDEGQPAYAPGLYTVHL  TKTAKDLGVYQSAINKAIH  LTINADGSVYAEEVKPFPS  GQPTAETADYFMSAMNFLQ  FMSAMNFLQYGNDLRVVRA  YGIPGVVALYPGELGDKIE  KGASALLPIYPGGGTRAST  ASTAKAVFGYGPQTDSQYA  STKRGEKDIYDSNIYIDDF  YAAIDGNHKYQYDKYNDVN  GNHKYQYDKYNDVNRWVPL  SQTWMSPAGYNRGQILNVI  PVTGTGGDGYVLYGDKTAT  GTGGDGYVLYGDKTATSVP  KTNIGRSSKYRLFELNNAF  SSFRTETAQYLQGNKALGG  KFNSDINLEYKGKSVNLKS  KTGQAPGFTYTDANKNKGI  TWKEETLMEYLENPKKYIP  MEYLENPKKYIPGTKMIFA  KTEREDLIAYLKKATNEXX  HLHQLAADTYKEFERAYIP  DTYKEFERAYIPEGQRYSI  RAYIPEGQRYSIQNAQAAF  LVFGTSDRVYEKLRDLEEG  RAGQILKQTYDKFDTNLRS  RSDDALLKNYGLLSCFKKD  KKDLHKAETYLRVMKCRRF  XXXMKPVTLYDVAEYAGVS  PVTLYDVAEYAGVSYQTVS  DVAEYAGVSYQTVSRVVNQ  VEAAMAELNYIPNRVAQQL  NLVGLIDSDYQGQLMISVW  ITTVQAAIDYINGHQAXXX  QKERLDPSEYANVKAQFVL  XXXMAQQSPYSAAMAEQRH  RFVDLLKNAYQNDLHLPLL  ILEPGTLQGYDRDPRSIAK  RSIAKRAEDYLRSTGIADT  GHRPAVKGGYFPVPPVDSA  GVNLFAGDKYAGLSEQALY  YAGLSEQALYYIGGVIKHA  NSYKRLVPGYEAPVMLAYS  GYEAPVMLAYSARNRSASI  PGEAMDKNLYDLPPEEAKE  YHYGKHHQTYVTNLNNLIK  DTFYPGQERYDTYSGRVVR  RGNRKIAAPYRGAVVLVNF  VFKTRIKMFYRPAHLKGRV  RNQQIKHGFYRDLPRLWMQ  MENLNMDLLYMAAAVMMGL  PMIAVGLGLYVMFAVAXXX  XXXXXXXMAYTTFSQTKND  VDVSRDRIDYQALPEHEKH  FSETIHSRSYTHIIRNIVN  AQQEKDWADYLFRDGSMIG  PRLLNEVMCYPLFDGGNIG  AMKRHGLDNYRGYSLGNWV  RHGLDNYRGYSLGNWVCAA  NRNTDGSTDYGILQINSRW  NSRGEFTGTYITAVTATSN  TALMGLGTLYFLVKGMGVS  TMYLSMLLGYGLTMVPFGG  GLVGALTKVYSYRFVWWAI  VGALTKVYSYRFVWWAIST  XXXXXXXVVYTDCTESGQN  GDFEEIPEEYLQXXXXXXX  MATLKDQLIYNLLKEEQTP  TPKIVSGKDYNVTANSKLV  VHKQVVESAYEVIKLKGYT  RVIKEKRDAYVSRLNAIYQ  AYVSRLNAIYQNNLTKSHI  PDGVPMPDKYSLEPVAVEL  ASLSKLGDVYVNDAFGTAH  GFLMKKELNYFAKALESPE  SQLLKDIDGYGGIALPELI  KILDIKGIDYWLAHKALCT  ITDNMFCAGYKPDEGKRGD  HRHQKTPENYPNAGLTMNY  YPNAGLTMNYCRNPDADKG  NGGTCVSNKYFSNIHWCNC  NLAEFAFSLYRQLAHQSNS  KFLEDVKKLYHSEAFTVNF  EEAKKQINDYVEKGTQGKI  RDTVFALVNYIFFKGKWER  LSSWVLLMKYLGNATAIFF  LPKLSITGTYDLKSVLGQL  EKRMDKVGKYPKELRKCCE  KKVFLDCCNYITELRRQHA  KHSVVKKCCYDGACVNNDE  XXXXXXMTEYKLVVVGAGG  EYDPTIEDSYRKQVVIDGE  TKSFEDIHQYREQIKRVKD  RQAQDLARSYGIPYIETSA  TRQGVEDAFYTLVREIRQH  EDPEVKFNWYVDGVEVHNA  HQDWLNGKEYKCKVSNKAL  PLGEKGERGYPGTPGPRGE  GAKGEPGEFYFDLRLKGDK  DRVKDLATVYVDVLKDSGR  DVLKDSGRDYVSQFEGSAL  DALRTHLAPYSDELRQRLA  ENGGARLAEYHAKATEHLS  VSFLSALEEYTKKLNTQXX  KNYCGLPGEYWLGNDKISQ  NGRYYWGGQYTWDMAKHGT  TSQCTLKEVYGFNPEGKAL  IPEGKQVFLYPEKDEPTYI  FLPFSYKNKYGMVAQVTQT  NPLLIDVVTYLVALIPEPS  GNLIFDPNNYLPKESMLKT  FPDSVNKALYWVNGQVPDG  TLEDTRDRMYQMDIQQELQ  LTIPEMRLPYTIITTPPLK  LGSDVRVPSYTLILPSLEL  KLSLESLTSYFSIESSTKG  KLDFREIQIYKKLRTSSFA  GVLYDYVNKYHWEHTGLTL  NLQNNAEWVYQGAIRQIDD  KAASGTTGTYQEWKDKAQN  FQFPGKPGIYTREELCTMF  LHHSKHHAAYVNNLNVTEE  NNLNVTEEKYQEALAKGDV  INWENVTERYMACKKXXXX  PKKKPLDGEYFTLQIRGRE  IYRAAYFGIYDTAKGMLPD  QRFGPVFTLYVGSQRMVVM  RYGLLILMKYPEIEEKLHE  ATRDTIFRGYLIPKGTVVV  FLNENGKFKYSDYFKPFST  GNYKKPKLLYCSNGGHFLR  LSAESVGEVYIKSTETGQY  FLERLEENHYNTYISKKHA  RLEENHYNTYISKKHAEKN  SCKRGPRTHYGQKAILFLP  IKVVDLLAPYAKGGKIGLF  NNVAKAHGGYSVFAGVGER  ERTREGNDLYHEMIESGVI  DATSKVALVYGQMNEPPGA  LGRIPSAVGYQPTLATDMG  SRAIAELGIYPAVDPLDST  DPNIVGSEHYDVARGVQKI  YDHLPEQAFYMVGPIEEAV  HIVEAEAMNYEGSPIKVTL  TPKIVADKDYSVTANSKIV  VHKMVVESAYEVIKLKGYT  NEKVILKLDYSDFQIVKXX  LVDQNIFSFYLSRDPDAQP  PSAYGSVKAYTNFDAERDA  LVGKDRSSFYVNGLTLGGQ  HGGLINKKCYEMASHLRRS  LHLKEDQTEYLEERRIKEI  VKKHSQFIGYPITLFVEKE  ITNEEYGEFYKSLTNDWED  ALRDNSTMGYMAAKKHLEI  RDYYDRMYSYPARVPPPPP  ASQNSFRIEYDTFGELKVP  RIKAAMPRIYELAAGGTAV  QHLRLVFDEYLKTTGKPIE  QKGSRGLDGYQGPDGPRGP  KGAPGRAGLYGEIGATGDF  TPKCEMKITYGQCGDVLRA  CGDVLRALGYNPTQAEVLR  STRSVSSSSYRRMFGGPGT  ASRPSSSRSYVTTSTRTYS  AALRDVRQQYESVAAKNLQ  EGSLKASCLYGQLPKFQDG  FARGAHGDSYPFDGPGNTL  ARYEREMKTYIPPKGETKK  ELRCQCIKTYSKPFHPKFI  TNAMQVINNYQRRCKNQNT  FPALCQLGKYTTNNSSEQR  ILILRICTRYTPEQDTMTF  EPLLEALKVYVRKRRPSRP  GTDQSKWKAYDATHLVKSY  YDATHLVKSYPGSQLDILI  GQKCEFQDAYVLLSEKKIS  IMQSSSEVGYDAMAGDFVN  DQGNRTTPSYVAFTDTERL  EYKGETKSFYPEEVSSMVL  TKMKEIAEAYLGKTVTNAV  TNAVVTVPAYFNDSQRQAT  NEPTAAAIAYGLDKKVGAE  SINPDEAVAYGAAVQAAIL  IRVQPNEAVYTKMMTKKPG  VGNRDDSNLYINVKLKAAE  GRKVVGDVAYDEAKERASF  PFMFRLDSQYRASAPNSHV  TLRSEGFDTYRCDRNLAMG  TIAMRAINNYRWRCKNQNT  KEDIQKETVYCLNDDDETE  KSEDFSLPAYMDRRDHPLP  LKVRIMGPNYTPGKKEDLY  YTPGKKEDLYLKPIQRTIL  LTDITKGVQYLNEIKDSVV  MARKIGARVYALPEDLVEV  VPSGASTGIYEALELRDGD  ELRDGDKGRYLGKGVLKAV  GAAEKGVPLYRHIADLAGN  EAMRIGAEVYHHLKGVIKA  HLKGVIKAKYGKDATNVGD  LKTAIQAAGYPDKVVIGMD  GMDVAASEFYRNGKYDLDF  ASEFYRNGKYDLDFKSPDD  ITGEKLGELYKSFIKNYPV  ELYKSFIKNYPVVSIEDPF  PCRSERLAKYNQLMRIEEA  ESFASDPILYRPVAVALDT  TLKITLDNAYMEKCDENIL  QTGKDISTNYYASQKKTFE  DRVGDGKILYSQCGDVMRA  ENSWTISKEYHIDEEVGFA  VKALVSLRSYHLQIVTKYI  SYHLQIVTKYILIPASQQP  EQLAAELAEYTAKIALLEE  KETVFTKSPYQEFTDHLVK  LGKKDTETVYSEVRKAVPD  LKKALAAGGYDVEKNNSRI  GGQALRDAGYEFDICFTSV  RTWRLNERHYGGLTGLNKA  YSNISKDRRYADLTEDQLP  RIGMTRKFGYVDFESAEDL  QNQNGKSKGYAFIEFASFE  LDRITGKWFYIASAFRNEE  IASAFRNEEYNKSVQEIQA  VQEIQATFFYFTPNKTEDT  QNQCFYNSSYLNVQRENGT  QRENGTVSRYEGGREHVAH  DTKTLMFGSYLDDEKNWGL  DEKNWGLSFYADKPETTKE  LCIPRSDVMYTDWKKDKCE  PAAGSRARVYAEVNSLRSR  VYSFDPVGSYQRDSFKAGG  RELTGLKALYETELADARR  AEHDQLLLNYAKKESDLNG  RKIGDTSVSYKYTSRYVLK  AMVDPFFRGYGVIFANGNR  SIVFGKRFHYQDQEFLKML  EFLKMLNLFYQTFSLISSV  YFPGAHRQVYKNLQEINAY  YKNLQEINAYIGHSVEKHR  SAPKDLIDTYLLHMEKEKS  ELHDRAKMPYTEAVIYEIQ  KMPYTEAVIYEIQRFSDLL  VTQHTSFRGYIIPKDTEVF  CGVGKIPPTYQIRFLPRXX  HIAKSPFEVYVDKSQGDAS  NPSGNLTETYVQDRGDGMY  AAIKFEGAIYRKDVGFRAI  LYEGKTKEVYELLDSPGKV  KDETNARDTYVSSFPRAPS  GKCKKKNCTYTQVQTRSAD  GMGPGTPAGYGRGREEYEG  DVGQTVDDPYATFVKMLPD  IKHELQANCYEEVKDRCTL  VVTDSIHQDYTTQNLIRMA  TKEDFATFDYILCMDESNL  RPKVVRSVHYCPATKKTIE  SVLAAANPVYGRYDQYKTP  LRRPPGREAYPGDVFYLHS  GTMKLELAQYREVAAFAQF  XXMLSVRVAYAVVRALPRR  LHSILKATNYNGHTCLHLA  TEFTEDELPYDDCVFGGQR  LLFKFRAKFYPEDVSEELI  KLSLDGQNIYNACCTLRID  QEDQGLTKDYGNSPLHRFK  KVNTPTTTVYRCGPLIDLC  ADMETLQRIYGISFPDPKM  LYQILRGLKYIHSANVLHR  KPRQIDNPDYKGTWIHPEI  VQIVSKKVSYSHIQSKCGS  KAPVPTGEVYFADSFDRGT  ADSRTTTGSYIANRVTDKL  PGTLYVYIIYGMYFCMNIS  KNWEVGSITYDTLSAQAQQ  EAEVAATGTYQLRESELVF  CRSAQEMFTYICNHIKYAT  WFAALGLRWYALPAVSNML  FPAAPFSGWYMSTEIGTRN  TRNLCDPHRYNILEDVAVC  EINVAVLHSYQLAKVTIVD  AKRVKATILYGSETGRAQS  GSETGRAQSYAQQLGRLFR  DPRVLCMDEYDVVSLEHET  SPRPEQHKSYKIRFNSISC  CVFGLGSRAYPHFCAFARA  DTGGQEGLQYQPGDHIGVC  TQLPLLQPRYYSVSSAPST  QLPLLQPRYYSVSSAPSTH  IHLTVAVLAYRTQDGLGPL  TQDGLGPLHYGVCSTWLSQ  SREPDNPKTYVQDILRTEL  IGVLRDQQRYHEDIFGLTL  KYDDAERRFYEQMNGPVAG  WDGLDSGKLYTLVLTDPDA  ISSGTVLSDYVGSGPPKGT  GHCKRLAPEYEAAATRLKG  TCNKYGVSGYPTLKIFRDG  ILKEQERLAYINPDLALEE  DIRKFLDGIYVSEKGTVQQ  KVKKFLQEFYQDDELGKKQ  SIKANPVFDYQLYFQEPGV  SGFRGPLNWYRNMERNWKW  QTKLKKEDIYAVEIVGGAT  TKSQGGEPTYNVAVGRAGR  CIDLGWKPKYGRFDVVPLV  LEVAMEHPKYEWFRELELK  HSAAESFMKYMQNEYRSRG  KQHIQIPKLYTSNVTWDPH  QHLQAAFSQYKKVELFPNG  DSKTEGKFTYPTGESLVYS  TYPTGESLVYSNWAPGEPN  KLEFIKEIGYTHMKIAEGV  IPNSHILDQYRNASNPLAH  AESLADPTEYENLFPGLKE  PGGSHMGGNYGDDRRGGRG  XXXXXXMPDYLGADQRKTK  KAWNDIKKVYASQRMRAGK  RKAASLKSNYNLPMHKMIN  WLTSIPWGKYSNENLDLAR  LGGLRSTCTYVGAAKLKEL  GASQAGMTGYGMPRQILXX  HVANTDKKSYEPLEDPGVK  AGKTKEGVLYVGSKTKEGV  MPVDPDNEAYEMPSEEGYQ  AYEMPSEEGYQDYEPEAXX  MPSEEGYQDYEPEAXXXXX  PPTRGPPPSYGGSSRYDDY  KNAVITVPAYFNDSQRQAT  ASLKLFEMAYKKMASEREG  SPLVSRLTLYDIAHTPGVA  IETKAAVKGYLGPEQLPDC  AEVFKKHGVYNPNKIFGVT  CWRSDTPLIYKAVPSWFVR  PELNEKEEAYNSLMKSYVS  GPSLNPVLDYDHGSRSQES  ELPNRIDAAYEHPSHDLIF  VNGPEILNKYVGESEANIR  SGQRKPATSYVRTTINKNA  ADEARSLKAYGELPEHAKI  RLTYRRRLSYNTASNKTRL  RQETKEAQLYAAQAHLKLG  IVLLDSSLEYKKGESQTDI  LEIKKIGDEYFTFITDCKD  VTDDLVCLVYKTDQAQDVK  GLRAVFDETYPDPVRVVSI  FKVTEGSFVYKGGKIYKVP  GKSPYLYPLYGLGELPQGF  TALPRLTPPYLEEMGHCDS  KANEVISKLYAVHQEGNKN  MDNQIVVSDYAQMDRVLRE  DRVLREERAYILNLVKQIK  VGKTSLMNQYVNKKFSNQY  KRGGNRFEPYANPTKRYRA  DDELDSKLNYKPPPQKSLK  RSHEVKAEGYEVAHGGRCV  PLVVTKDGVYVLDLAAKVD  AAKVDATADYICKVKWGDI  IFVRRGGPNYQEGLRVMGE  RPGRLDQLIYIPLPDEKSR  TEADVNPKAYPLADAHLTK  RMTLDTLSIYETPSMGLLD  AGTGGMWRFYTEDSPGLKV  RAILPCIKGYDVIAQAQSG  LSRGFKDQIYDIFQKLNSN  PKVRFMTKIYHPNVDKLGR  ACVVDCGTGYTKLGYAGNT  YFRGSELQNYFTKILEDDL  DVEQKKSGNYFFLDDXXXX  RNNQIRKTSYAQHQQVRQI  TKVVLDDKDYFLFRDGDIL  LCRRCGSKAYHLQKSTCGK  KNIKALRTDYNASVSVPDS  PPRGGDLMAYDRRGRPGDR  HKVETFSGVYKKLTGKDVN  RAHQVVEDGYEFFAKRQLV  RVFDKDGNGYISAAELRHV  DIDGDGQVNYEEFVQMMTA  CRVALRNDSYTLHKILPNK  RKTRIIDVVYNASNNELVR  PGQCGRADGYVLEGKELEF  KEIKDILIQYDRTLLVADP  KADRDESSPYAAMLAAQDV  SNRHGLIRKYGLNMCRQCF  TPKEAIEGTYIDKKCPFTG  DGKVRTDITYPAGFMDVIS  QIKQAVKKLYDIDVAKVNT  KAYVRLAPDYDALDVANKI  GVKRISGLIYEETRGVLKV  ENVIRDAVTYTEHAKRKTV  KTVTAMDVVYALKRQGRTL  FHRKKNLQYYDISAKSNYN  EVLLMCNKSYCAEIAHNVS  GKLARASGNYATVISHNPE  GQTGMFPRNYVTPVNRNVX  NDAGEFVDLYVPRKCSASN  WKLTRDETNYGIPQRALRG  IMELDDTLKYSFLQFDPAP  KSTTTGHLIYKCGGIDKRT  PLRLPLQDVYKIGGIGTVP  EFTPPVQAAYQKVVAGVAN  GVANALAHKYHXXXXXXXX  GKVGAHAGEYGAEALERMF  FLSFPTTKTYFPHFDLSHG  SVSTVLTSKYRXXXXXXXX  KLDPSPQTIYKWIKDNISP  KLGGSLADSYLDEGFLLDK  GKKLLPDDPYEKACQKMIL  KVELCSFSGYKIYPGHGRR  KTELERAFGYYGPLRSVWV  KVRIQTQPGYANTLRDAAP  FNALFAQGNYSEAAKVAAN  ATHNALAKIYIDSNNNPER  RDPHLACVAYERGQCDLEL  ASTLVHLGEYQAAVDGARK  KHAAENPGKYNILGTNTIM  XXXXXXXMSYGRPPPDVEG  INKTDGCHAYLSKNSLDCE  TTAGAVTQCYRDMGARHRA  GEGENLDLPYGLERAIQRM  EIFDTEKEKYEITEQRKID  SEKTNRTLSYRTGLKITSL  QGNSGRAVTYSRSRYLECI  RAVTYSRSRYLECISCGSS  DDRHLRGCGYLPGCPGSNG  NLPQNGRQCYSCKGNSTHG  GTHEPKNQSYMVRGCATAS  THPTIKINGYTGPGTVRIS  VPIEEQRGDYDLNAVRLCF  MWRSGDSEVYQLGDVSQKT  QEVVELLIKYGADVHTQSK  KPDVQKSKEYFSKQKXXXX  RTKDIEDVFYKYGAIRDID  EFVRKEDMTYAVRKLDNTK  FRSHEGETAYIRVKVDGPR  YMRQAGEVTYADAHKGRKN  KEKIQGEYKYTQVGPDHNR  FFRKLSSETYSQAKDLAKG  PSSDVLVFDYTKHPSKPDP  AGSNKKVAKYYAALAALEK  DGATEQAAEYVPEKVKKAE  LKGVEAVGSYAENQRITAV  GFGFVRFTEYETQVKVMSQ  LIKTLQKGEYFGEKALISD  IHKVNSMVAYKIPVNDXXX  QLNAHKDHQYQFLEDAVRN  VFPGSTTEDYNLIVIERGA  SYSSGGEDGYVRIHYFDPQ  MQREEQCILYLGPRYGFGE  SIRPDNMSEYSKQMQRFNV  GGKDMAQSIYRPSKNLDKD  VYKSPASDTYIVFGEAKIE  KLQIASDENYKDPTNLQGK  EALMSDLSAYGSSIQALRE  TGKELVLALYDYQEKSPRE  KNQALNTDNYGHDLASVQA  ADQLIAAGHYAKGDISSRR  RALSSEGKPYVTKEELYQN  EEELQDSRVYVSSLXXXXX  YEDAEAKAKYEAEAAFFAN  DYWSLGILMYELLTGSPPF  SLHDKFKVQYPQSQACKMS  PLLVQDVESYDPVLNPVLN  ESEDFIVEQYKHLRQRDGS  RVIVGGSSEYKINNKVVQL  EPETFLPLDYLEVKPTDEK  FGGHIRSVVYQQSSKESAT  GDTGMLPANYVEAIXXXXX  SYIDEQFERYLHDESGLNR  DFDTYDDRAYSSFGGGRGS  DTDKFKGFCYVEFDEVDSL  VDSLKEALTYDGALLGDRS  ERAAPFSLEYRVFLKNEKG  NVDDPDAANYNDINDVKRL  PQGRVMTIPYQPMPASSPV  KRPPDEQRLYKDDQLLDDG  QLIIFADDTYPRWVTTASL  SERNQDATVYVGGLDEKVS  YFMKNHLGSYECKLCLTLH  MPRHRFMSAYEQRIEPPDR  PSKPVVGIIYPPPEVRNIV  REKQSDDEVYAPGLDIESS  VKLGGDLGTYVINKQTPNK  YDWTGKNWVYSHDGVSLHE  TKLDLSSLAYSGKDAXXXX  GRYGGETKVYVGNLGTGAG  YKDGKRLKNYNMPWGAGHN  TGGVKKPHRYRPGTVALRE  LRPLRPCRRYSTSGSSGLT  XXMAAPPGEYFSVGSQVSC  VLDTLEALGYKGPLLEEQA  ASDALDKIRYESLTDPSKL  KKTKKIKEKYIDQEELNKT  RMKEIQKSIYYITGESKEQ  RRPPLRYPRYEATSVQQKV  RILPKCRTSYEEFTHKDGV  VIDEVRTGTYRQLFHPEQL  TGKEDAANNYARGHYTIGK  AANNYARGHYTIGKEIIDL  LLMERLSVDYGKKSKLEFS  RNLDIERPTYTNLNRLIGQ  TEFQTNLVPYPRIHFPLAT  PRIHFPLATYAPVISAEKA  APVISAEKAYHEQLSVAEI  PTGFKVGINYQPPTVVPGG  GEGEEEGEEYXXXXXXXXX  KRLVQSPNSYFMDVKCPGC  ERRLRIWRDYVAPTANLDQ  EELIRLQNSYQPTNKGRYK  SFLTTLAKVYPSSLSKISG  EAIASFVKKYNTYAYVGLT  ASFVKKYNTYAYVGLTEGP  FVKKYNTYAYVGLTEGPSP  GQWNDRNCLYSRLTICEFX  CLHKFSTRDYIMEPSIFNT  CLHGIEAKSYSVGASGSSS  CQGGQETQEYRLYREKKTA  WEHAGRYRCYYGSDTAGRS  EHAGRYRCYYGSDTAGRSE  PLELVVTGAYIKPTLSAQP  TLQCGSDAGYNRFVLYKDG  LRSTYQSQKYQAEFPMGPV  CYGSQSSKPYLLTHPSDPL  KTEVNSGFFYKTAEEKEKL  GARFMTTAMYDAREAIIPG  VWLGKHYKKYVHADAPTNK  IRGTPQQIDYARQLIEEKI  MAGPESDAQYQFTGIKKYF  QPMTAQAASYRAQPSVSLG  LRVSEVKPSYRNAITVPFK  SKAIQLNPSYIRAILRRAE  KLEANSQKQYLLTGQVLSD  TDWLLERKLYGYQAQHYVC  WLLERKLYGYQAQHYVCMK  KLYGYQAQHYVCMKHVDGT  GVAFKKTFSYAGFEMQPKK  ARNPPGASTYNKNSNIPGS  SQLEHLQSKYIGTGHADTT  SSDEVNFLVYRYLQESGFS  WGRVHATSTYREATPENDP  FKDPQFAEDYIFKAVMLPG  IKRAEVSPMYGVQKYDPIN  XXXMELSAEYLREKLQRDL  GLELLSDQGYRVDGRRAGE  AVIGDVIRVYSGHQGRTII  FNRKALERPYTSKHSWGKT  KKLAEQEAHYAVRQQRART  DESRGRSSFYPDGGDQETA  XXXXXXMPLYEGLGSGGEK  TQKAARNMSYQGFTKDNGV  LKPDQKQRDYILEGEPGKV  WGVIDVKRSYVTMTATKIE  PSGVKRLPEYPQVDDLLLR  KWVKLEKTTYMDPTGKTRT  KELEQKEVDYSGLRVQAMQ  ASKETHVMDYRALVHERDE  YTLLNKAPEYLHRFYGRNS  GAAPEEESAYVAGEKRQHS  VQVDDIKRVYSLFLDESRS  AEALQLPMDYVQRVKRTHS  EPGYKAEGKYKDDPVDLRL  EQKRAQLSQYFDAVKNAQH  PSILKKTSAYGPPTRAVSI  KKKDTAASGYGTQNIRLSR  TVDFTQDSNYLLTGGQDKL  XXXXXMALRYPMAVGLNKG  DELPAEFKLYLPVQNKAKQ  LYRDVMLEIYSNLLSMGYQ  ISADTHKYGYAPKGSSLVL  APKGSSLVLYSDKKYRNYQ  RLTPTEVKDYLAAIAXXXX  DESLKGCELYVQLHGIQQV  EINPDSAQPYKWRGKAHRL  DLALACKLDYDEDASAMLK  KIEISQHAKYTCSFCGKTK  LLLKHIGFVYHPTKETCLS  LGNVIINQIYEARVEAMAV  AIAGALPPDYLDTRITATL  FQRAAGVLNYLKDTFTHTP  LKEKDIMDLYNQPEPVKSF  RERVTPPEGYEVVTVFPKX  QTLRDSMLKYPDSHQPRIF  ILQELQVETYGSMEKKERV  WNKLGPNDQYKFYSVNVDY  PEKTKAVENYLIQMARYGQ  NAPTTHTEEYSGEEKTWMW  HPDKAAGQPYEQHAKMIFM  PVKKLLESRYHQIGSGKCE  GRTHPVEIFYTPEPERDYL  PHPETGKDLYEPSHGAKVL  TVGPRLASEYLTPEEMVTF  DSVLGQTGPYRRPQRLRKR  KGPQNATDSYVHKQYPANQ  LVWDLRNMGYVQQRRESSL  SIEGRVAVEYLDPSPEVQK  GREVRPDTQYTGRKRKPRF  SFRDPEEADYCIQTLDGRW  LELDPDNETYKSNLKIAEL  VGKEPFPTIYVDSQKENER  FDSEEILAGYKREGIDPTP  AEAEARHLVYESDQNKDGK  GVEMGKVQVYQEPNRETRV  LERVKKLKDYAFIHFDERD  GQCKDALEIYKRFLTRMTR  DKFKEISFAYEVLSNPEKR  RYTSLMLRDYSGQGVVKLD  EKDLEEAEEYKEARLVLDS  NSESNFARAYAQGISRTKY  LNSGRVVPGYGHAVLRKTD  EQSGRDLQQYQSQAKQLFR  TVKAKADALYPVVSAASIC  GANPDAKDHYEATAMHRAA  LKVPVPEDKYTAQVDAEEK  SLSKARIVEYEKEMEKMKN  HGQRTFGENYVQELLEKAS  AAYIQKLKSYVDTGGVSRK  PMEEDDTAMYFCQQSKEVP  ATVLDSDGSYFMYSKLRVQ  QKRPSQRSKYLATASTMDH  KDSHTRTTHYGSLPQKSQH  XXXXXMPHPYPALTPEQKK  MENANVLARYASICQQNGI  ANDNSLNHEYLPILGLAEF  KKMNLGVGAYRDDNGKPYV  IAAKNLDKEYLPIGGLAEF  QLKILIRPLYSNPPLNGAR  FSQYLQKCSYDEHAKLVQE  YEYSRRHPDYSVSLLLRLA  CDLYEKLGEYGFQNAILVR  YFSTSYKRRYVETPRVHIS  SEPSRFRALYEQEIRDLRL  ETLRNLQARYEEEVLSRED  AALKDIRAQYEKLAAKNMQ  RSTKSEMARYLKEYQDLLN  ASHAQLGDAYDQEIRELRA  XXXMPMILGYWNVRGLTHP  THPIRMLLEYTDSSYDEKR  MLLEYTDSSYDEKRYTMGD  TDSSYDEKRYTMGDAPDFD  LGLDFPNLPYLIDGSHKIT  ITQSNAILRYLARKHHLDG  KTIPEKMKLYSEFLGKRPW  KVTYVDFLAYDILDQYRMF  KRKRSRKESYSVYVYKVLK  RSRKESYSVYVYKVLKQVH  RKESYSVYVYKVLKQVHPD  LETYPLMMKYNPNVLPVQC  DHQPLTEASYVNLPTIALC  RTVGQEKKGYFEDRRPSAN  RRPSANCDPYAVTEAIVRT  NETGDEPFQYKNXXXXXXX  IKWGEAGAEYVVESTGVFT  TCRLEKPAKYDDIKKVVKQ  KLISWYDNEYGYSNRVVDL  VPSGASTGIYEALELRDGD  GMDVAASEFYRDGKYDLDF  FKSPADPSRYITGDQLGAL  VTGYSITSGYYWNWIRQFP  TGYSITSGYYWNWIRQFPG  GFSGEDVKQYKPVVYSNTI  DRHQADIASYQDAIQQLDS  FPTYADRRDYPLPDVAHVT  LARDINNIDYYKKTTNGRL  GMVAGDEETYEVFAELFDP  EFMWNERLGYILTCPSNLG  RLSHRPKTGYIRVVMYEGK  KTGYIRVVMYEGKKIMADS  SPVPINLRVYSPHVLNLTL  RKFFLSHPSYRHLADRMGT  EGSGDQIDTYELSGGARIN  MAEQIIQEIYSQVQSKKKI  FRKLHCTRNYIHLNLFLSF  VALIDVFHQYSGREGDKHK  SQAASDLLAYCEAHVREDP  DGEAKIKADYAQLLEDMQN  XXXXXXMEGYQRPCDEVGF  LKVNDEHPAYLASDEITTV  GSLCADARMYGVLPWNAFP  MVGMGQKDSYVGDEAQSKR  SKRGILTLKYPIEHGIVTN  MEKIWHHTFYNELRVAPEE  THTVPIYEGYALPHAILRL  DLAGRDLTDYLMKILTERG  LMKILTERGYSFTTTAERE  VRDIKEKLCYVALDFEQEM  ASSSSLEKSYELPDGQVIT  CDVDIRKDLYANTVLSGGT  QQMWISKQEYDESGPSIVH  XMDDREDLVYQAKLAEQAE  EERNLLSVAYKNVIGARRA  EDKLKMIREYRQMVETELK  YKMKGDYHRYLAEFATGND  ELDTLSEESYKDSTLIMQL  ILDHVKFPRYAEIVHLTLP  IPQSTLSEFYPRDSAKHXX  HIKKEFDKKYNPTWHCIVG  LDRFKEPPAYGPMCDILWS  KLEAAEDIAYQLSRSRNIT  TRLLLGGGSYKCIKCSKVF  DVPILLTEQYPEGLGPTVP  ASLGTDGTHYWSKNWAKAA  LSPDPTTPDYLTSLLACGD  ITSPGWPKEYPPNKNCIWQ  MAKVLTPELYAELRAKCTP  IETLFKSKNYEFMWNPHLG  DEFQFLQAQYHSLKVEYDK  KDFKKGMNMYLTKFQQKNA  LKLTQPSSFYSQRLAELTP  LQIERERSAYNISGGCTAL  RLKTVLELQYVLDKLGDDD  GQLGLYPANYVEAIXXXXX  NCPKPVLNFYEANFPANVM  KGMFFNPDPYLKMSIQPGK  MSVEEVCRKYNTDCVQGLT  EMKEAFQNAYLELGGLGER  EPEEENGREYKFDVSSELL  EHGIDPTGSYHGDSDLQLE  LQLERINVYYNEAAGNKYV  AGNNWAKGHYTEGAELVDS  RTLKLTTPTYGDLNHLVSA  KMVSLGTLSYVLLDAEATK  AKLHGLDRVYFGGFFIRGH  APLAEGALPYNLAELQRQQ  YELIVDGRVYYICIVCKRS  ELIVDGRVYYICIVCKRSY  NGIKINSSAYRSAFESRLA  AVAAHDVGEYVRHYPRGKR  XXXXXXXXMYSGAGPVLAS  VKHQAQVDQYLGLVRTHIN  PIDFPLAPAYAVPKVLKYA  XXXXXXMESYDIIANQPVV  SEIAGITLPYDTLDQVRNR  LEESVYMDIYGKHQKQIID  XXXXXXMDCYTANWNPLGD  KNWFKITIPYGRKYDKTWL  ILETQKQFSYTHICAGASA  KSGENFKLLYDLADQLHAA  EKKQQMGKEYREKIEAELQ  NDVLELLDKYLILNATQAE  ELDTLNEESYKDSTLIMQL  QNRSSPCIHYFTGTPDPSR  VSTRHHTKFYTDPVEAVKD  KQIKRMISSYVGENAEFER  KRIEAIVKNYPEGHQAAAV  AIRRLPEDLYNDRMLRIKR  RFNQCRERIYTKLIKFTSW  KLVRPPVQVYGIEGRYATA  TGIGMAPGGYVAPKAVWLP  ENQIHRGGQYFNDKFIGLR  VKLVADFQRYILYWRKRFT  VADFQRYILYWRKRFTEQP  EYMRLIGQKYLKDAIGEFI  LLVGNLKKKYAQGFLPEKP  KPKRPAIFTYHDVGLNYKS  GSFGQVVKAYDHQTQELVA  DLDRIAQPNYIPTQQDVLR  KALRSSVSDYGNYDIIVRH  RFQLTDSQIYEVLSVIRDI  AKTHNSALEYNIFEGMECR  KAIKELEEWYARQDEQLQK  GEVVRVRDSYVAIANACCA  CYIGTLASVYHKPPNAFVE  FCKLDYFRRYGTRCSRCGR  HGAGEHCGRYDELAHMLKG  QENEKLMEEYEKLASELLE  KCNKLVPLFYKTCPAGKNL  TCPAGKNLCYKMFMVATPK  CPKSSLLVKYVCCNTDRCN  GCSGGETNCYKKRWRDHRG  KKRWRDHRGYRTERGCGCP  TCPAGKNLCYQMYMVSKST  AGKNLCYQMYMVSKSTIPV  CPKSSLLVKYVCCNTDRCN  KTCPGETNCYKKVWRDHRG  REIGYMFGAYRKAANRFEG  XXXXXXTKCYVTPDVKSET  TCPAGQDICYTETWCDAWC  VALARLSRRYGDVFQIRLG  MAAVGRFDPYSQLVVSAAR  PVDFFPILRYLPNRPLQRF  FLQKTVREHYEDFDRNSIQ  RLSDRPQLPYLEAFILELF  GRWIRTQQHYYEKDPKRIY  RWIRTQQHYYEKDPKRIYY  YYEKDPKRIYYLSLEFYMG  YEKDPKRIYYLSLEFYMGR  RIYYLSLEFYMGRTLQNTM  LENACDEATYQLGLDMEEL  LAAYGYGIRYEFGIFNQKI  MEEADDWLRYGNPWEKARP  PEFTLPVHFYGRVEHTSQG  DTQVVLAMPYDTPVPGYRN  MPYDTPVPGYRNNVVNTMR  NLKDFNVGGYIQAVLDRNL  LAENISRVLYPNDNFFEGK  GKELRLKQEYFVVAATLQD  LLPRHLQIIYEINQRFLNR  LKKTIFKDFYELEPHKFQN  IIAERIGEEYISDLDQLRK  LDQLRKLLSYVDDEAFIRD  QENKLKFAAYLEREYKVHI  KFAAYLEREYKVHINPNSL  MIGGKAAPGYHMAKMIIKL  RLRVIFLENYRVSLAEKVI  DVDRLDQRGYNAQEYYDRI  HDRFKVFADYEEYVKCQER  FKVFADYEEYVKCQERVSA  KCQERVSALYKNPREWTRM  FSSDRTIAQYAREIWGVEP  GDHDLKRCQYVTEKVLAAV  YKALSDHHIYLEGTLLKPN  TPGHACTQKYSHEEIAMAT  ENLKAAQEEYVKRALANSL  ANSLACQGKYTPSGQAGAA  ESLFISNHAYXXXXXXXXX  DDPEVQFTWYINNEQVRTA  DEFKEAFLLYDRTGDSKIT  ISNNKDQGTYEDFVEGLRV  QEDSNGCINYEAFVKHIMS  DHFVKLISWYDNEFGYSNR  ISWYDNEFGYSNRVVDLMV  PECKLKENKYFSKPDAPIY  CMGCCFSRAYPTPARSKKT  HTECHCSTCYYHKSXXXXX  TECHCSTCYYHKSXXXXXX  EMFNEFDKRYAQGKGFITM  IPGAKETEPYPVWSGLPSL  DEDARHSAFYNLLHCLRRD  RRDSSKIDTYLKLLNCRII  LKLLNCRIIYNNNCXXXXX  EEYVQQLSGYFLQLKFDPE  RIAMEFNQLYHWHPLMPDS  LFNTSMLVDYGVEALVDAF  EMVNRFDEQYGQGINSESK  TFEEATAKAYAYADLLAKE  EEATAKAYAYADLLAKENG  DLLAKENGEYTADLEDGGN  VWDPSFRTFYSIIDDEHKT  EQVLMQASQYQFYDEHKKE  LMQASQYQFYDEHKKEHET  ATKHKIPIKYLEFISEAII  LFRKDIAAKYKELGYQGXX  IAAKYKELGYQGXXXXXXX  FIPREAGEAYDFIPTSMDP  PEEDIPHLKYLTDQMTRPD  TFAEAKEALYDYLIPIIEQ  AEAKEALYDYLIPIIEQRR  ADGRILTSDYEFHGVQLKK  NTHNKFKLNYKPQEEYPDL  KLNYKPQEEYPDLSKHNNH  MAKVLTPDLYNKLRDKETP  LQYLSGGSWYHTCGGTLIR  VNPYKWLPVYNAQVVAAYR  VYNAQVVAAYRGKKRSEAP  DPDTAAVQPYQDQTYQPVY  AVQPYQDQTYQPVYFVSES  YQDQTYQPVYFVSESFNDA  KSLEAQAEKYSQKEDKYEE  GAVEKGVPLYRHIADLAGN  ASEFYRAGKYDLDFKSPDD  PGDAYGDAGYGQGPGGYGP  VPFLGIGLLYSLSGPDLST  AYRLLRSRLYLXXXXXXXX  EDRGFQGRCYECSSDCPNL  XMEVHELFRYFRMPELIDI  LEDDKLLLYYYDDVRTMYD  APQKVIEQSYNATWLGRQG  LRSSISSTSYRRTFGPPPS  EEMARHLREYQELLNVKMA  GNTIHAILLYRRTLDREEL  KDMGKFCLTYEASMTRLFR  LSRGGNGHRYIPGATMGTS  PREETGSEEYMNMDLGPGR  MQIGCPRQSYVDTSPVAPV  DTSPVAPVSYADMRTGIAA  AGGLEKSLNYIDLDLVKDV  PEGKKPRQRYMEKTGKCNV  KETKTGTCAYCGLQFKQQH  GGFVWACKNYDGDVQSDIL  LVSSENFDDYMKEVGVGFA  LPKCKCSSGYSGEYCEVGL  LMKKITLLNYFRNYMSEHL  QKLLIKEGQYKCSKTAPSP  RRNAGEKIKYRLVAVPHGN  AEKTKEGVMYVGTKTKGER  WRMVWEKNVYAIVMLTKCV  FFESRWEEFYQGKQANLXX  PLCEKIGKIYEMRMMMDFN  GLAPLNIPRYEFGICVLDQ  LRDSRKVNTYLEVIKYQIF  ICLREPCENYMRCVSVLRF  GFSCKCVAGYHGSNCSEEI  GTCIDLTNTYKCSCPRGTQ  APAVISDFIYQGASLHNQT  RPDEKAIMTYVSSFYHAFS  AKRNFQIDVYEAREDTRVA  NIKNDPDKEYNMPKDGTVH  XXXXXMEERYYPVIFPDER  XXXXMEERYYPVIFPDERN  TWKLLGSVVYAHSKELVTA  SDDDLEFMVYPWPDYFGKA  EALRIGSEVYHNLKSLTKK  SSEFFKDGKYDLDFKNPNS  QAFEKMTSGYYLGELLRLV  AFEKMTSGYYLGELLRLVL  QDLSKLKQPYIMDTSYPAR  KQPYIMDTSYPARIEDDPF  ITPEYLVKIYKEIVIPITK  YYIVESYGSYDPSSAASHK  QGLAKVAYVYKPNNTHEQH  HINKDDIVAYSPILEKYVG  XXXXMEQNSYVIIDETGIH  KFDSDIQLEYNGKKVNLKS  APGDAPSALYAPSALVLTV  GEDVMCPMVYDPVLLTVDG  ICCGEELGCYFGTSETLRC  SCKFLVDNPYTESYSYSCS  EHKNLDTKKYCXXXXXXXX  CGSHLVEALYLVCGERGFF  LVCGERGFFYTPKARREAE  QCCTSICSLYQLENYCNXX  ICSLYQLENYCNXXXXXXX  NSECSAFTLYGVYYKCPCE  CSAFTLYGVYYKCPCERGL  SAFTLYGVYYKCPCERGLT  WFVREAVLTYGYESHMTEF  IKAYLTIHSYSQMILYPYS  SQMILYPYSYDYKLPENNA  ATLYGTKYTYGPGATTIYP  TYGPGATTIYPAAGGSDDW  AAGGSDDWAYDQGIKYSFT  TFELRDKGRYGFILPESQI  PLTLSEKIVYGHLDDPANQ  RAKDINQEVYNFLATAGAK  GEKNTIVTSYNRNFTGRND  XXXXMDKVQYLTRSAIRRA  DEAEGLGLAYEVFPFGGAA  VELIRGTGSYNRSSFESSS  TGCNPKLGKYTYSWNNGDI  CNPKLGKYTYSWNNGDIVC  ICFRDNLDTYDRNKYWRYP  ENAIEALKEYEPEMGKVYR  EYEPEMGKVYRQDRKSVQR  GGSWIRGAIYYFKIAVALA  GSWIRGAIYYFKIAVALAV  SRDRKSMSVYCTPNKPSRT  LEDSANFIKYETNLTFVGC  AAVEEGRAIYNNMKQFIRY  ETLKFVARNYLEPGKECAQ |

**II. Negative dataset contains 7,669 samples**

| Uniprot ID | Site | Sequence |
| --- | --- | --- |
| Q9L422  P00781  P00781  P07999  P07999  P07999  P07999  P07999  P07999  P14779  P14779  P14779  P14779  P14779  P14779  P14779  P14779  P14779  P14779  P14779  P14779  P14779  P14779  P14779  P14779  P14779  P14779  P14779  P14779  P14779  P14779  P14779  P14779  P14779  P14779  P14779  P14779  P14779  P14779  P14779  P14779  P14779  P14779  P68735  P68735  P68735  P68735  P68735  P68735  P68735  P68735  P68735  P68735  P68735  P68735  P68735  P68735  P68735  P68735  P68735  P68735  P68735  P68735  P68735  P68735  P68735  P68735  P68735  P68735  P00366  P00366  P00366  P00366  P00366  P00366  P00366  P00366  P00366  P00366  P00366  P00366  P00366  P00366  P00366  P00366  P00730  P00730  P00730  P00730  P00730  P00730  P00730  P00730  P00730  P00730  P00730  P00730  P00730  P00730  P00730  P00730  P00730  P00730  P00730  P00766  P00766  P01180  P01180  P02663  P02663  P02663  P02663  P02663  P02663  P02663  P02663  P02663  P02663  P02663  P02666  P02666  P02666  P02754  P02754  P02754  P02769  P11151  P11151  P11151  P11151  P11151  P11151  P11151  P11151  P11151  P11151  P11151  P17694  P17694  P17694  P17694  P17694  P17694  P17694  P17694  P17694  P17694  P17694  P17694  P17694  P17694  P17694  P17694  P17694  P17694  P23709  P23709  P23709  P23709  P23709  P23709  P23709  P23709  P23709  P31039  P31039  P31039  P31039  P31039  P31039  P31039  P31039  P31039  P31039  P31039  P31039  P31039  P31039  P31039  P31039  P31039  P31039  P31039  P31039  P31039  P31039  P48305  P48305  P48305  P48305  P48305  P61625  P61625  P61625  P61625  P61625  P61625  P61625  P61625  P61625  P61625  P61625  P61625  P61625  P61625  P61625  P61625  P61625  P61625  P61625  P61625  P61625  P61625  P61625  P61625  P61625  P61625  P61625  P61625  P61625  P61625  P61625  P61625  P61625  P61625  P61625  P61823  P61823  P61823  Q02366  Q02366  Q9N2I2  Q9N2I2  Q9N2I2  Q9N2I2  Q9N2I2  Q9N2I2  Q9N2I2  Q9N2I2  Q9N2I2  P60615  P00294  P00294  P00784  P00784  P00784  P00784  P00784  P00784  P00784  P00784  P00784  P00784  P00784  P00784  P00784  P00784  P00784  P00784  P00784  P00784  P00784  P00784  P00784  P00784  P00784  P00784  Q60468  Q60468  Q60468  Q60468  Q60468  Q60468  Q60468  P69542  P69542  P03040  P13332  P13332  P13332  P13332  P13332  P13332  P13332  P13332  P13332  P13332  P13332  P13332  P13332  P13332  P13332  P13332  P13332  P13332  P03023  P03023  P03023  P03023  P06968  P0A786  P0A786  P0A786  P0A786  P0A786  P0A786  P0A786  P0A9C5  P0A9C5  P0A9C5  P0A9C5  P0A9C5  P0A9C5  P0A9C5  P0A9C5  P0A9C5  P0AGD3  P0AGD3  P0AGD3  P0AGD3  P0AGD3  P0AGD3  P0AGD3  P0AGD3  P12758  P12758  P12758  P33341  P33341  P33341  P33341  P33341  P33341  P33341  P33341  P33341  P33341  P33341  P33341  P33341  P33341  P33341  P33341  P33341  P33341  P33341  P33341  P33341  P33341  P33341  P33341  P33341  P33341  P33341  P33341  P33341  P33341  P33341  P33341  P33341  P33341  P33341  P33341  P33341  P33341  P33341  P33341  P33341  P33341  P33341  P33341  P33341  P33341  P33341  P42914  P42914  P42914  P42914  P42914  P42914  P42914  P42914  P45848  P45848  P45848  P45848  P45848  P45848  P45848  P45848  P45848  P45848  P45848  P45848  P45848  P45848  P45848  P45848  P45848  P45848  P45848  P45848  P45848  P45848  P45848  P45848  P45848  P45848  P45848  P69924  P69924  P69924  P69924  P69924  P69924  P69924  P69924  P69924  P69924  P69924  P69924  Q8X1D8  Q8X1D8  Q8X1D8  Q8X1D8  Q8X1D8  Q8X1D8  Q8X1D8  Q8X1D8  P02945  P02945  P02945  P02945  P02945  P02945  P02945  P00338  P00338  P00338  P00338  P00338  P00390  P00390  P00390  P00390  P00390  P00390  P00390  P00390  P00390  P00390  P00390  P00558  P00709  P00709  P00734  P00734  P00734  P00734  P00734  P00734  P00734  P00734  P00734  P00734  P00734  P00734  P00734  P00734  P00734  P00734  P00734  P00734  P00734  P00734  P00747  P00747  P00747  P00747  P00747  P00747  P00747  P00747  P00747  P00747  P00747  P00747  P00747  P00747  P00747  P00747  P00747  P00747  P00747  P00747  P00747  P00747  P00747  P00747  P00747  P00747  P00747  P00747  P00749  P00749  P00749  P00749  P00749  P00749  P00749  P00749  P00749  P00749  P00749  P00749  P00749  P00749  P00749  P00749  P00749  P01024  P01024  P01024  P01024  P01024  P01024  P01024  P01024  P01024  P01024  P01024  P01024  P01024  P01024  P01024  P01024  P01024  P01024  P01024  P01024  P01024  P01024  P01024  P01024  P01024  P01024  P01024  P01024  P01024  P01024  P01024  P01024  P01024  P01024  P01024  P01024  P01024  P01024  P01024  P01024  P01024  P01024  P01024  P01024  P01024  P01024  P01024  P01024  P01024  P01024  P01024  P01024  P01024  P01024  P01024  P01031  P01031  P01031  P01031  P01031  P01031  P01031  P01031  P01031  P01031  P01031  P01031  P01031  P01031  P01031  P01031  P01031  P01031  P01031  P01031  P01031  P01031  P01031  P01031  P01031  P01031  P01031  P01031  P01031  P01031  P01031  P01031  P01031  P01031  P01031  P01031  P01031  P01031  P01031  P01031  P01031  P01031  P01031  P01031  P01031  P01031  P01031  P01031  P01031  P01031  P01031  P01031  P01031  P01031  P01031  P01031  P01031  P01031  P01031  P01031  P01031  P01031  P01031  P01031  P01031  P01031  P01031  P01031  P01031  P01031  P01031  P01031  P01031  P01031  P01031  P01031  P01031  P01031  P01031  P01031  P01031  P01031  P01112  P01112  P01112  P01112  P01857  P01857  P01857  P01857  P01857  P01857  P01857  P01857  P01857  P01857  P02462  P02462  P02462  P02462  P02462  P02462  P02462  P02462  P02462  P02462  P02462  P02462  P02462  P02462  P02462  P02462  P02647  P02647  P02675  P02675  P02675  P02675  P02675  P02675  P02675  P02675  P02675  P02675  P02675  P02675  P02675  P02675  P02675  P02675  P02675  P02675  P02675  P04114  P04114  P04114  P04114  P04114  P04114  P04114  P04114  P04114  P04114  P04114  P04114  P04114  P04114  P04114  P04114  P04114  P04114  P04114  P04114  P04114  P04114  P04114  P04114  P04114  P04114  P04114  P04114  P04114  P04114  P04114  P04114  P04114  P04114  P04114  P04114  P04114  P04114  P04114  P04114  P04114  P04114  P04114  P04114  P04114  P04114  P04114  P04114  P04114  P04114  P04114  P04114  P04114  P04114  P04114  P04114  P04114  P04114  P04114  P04114  P04114  P04114  P04114  P04114  P04114  P04114  P04114  P04114  P04114  P04114  P04114  P04114  P04114  P04114  P04114  P04114  P04114  P04114  P04114  P04114  P04114  P04114  P04114  P04114  P04114  P04114  P04114  P04114  P04114  P04114  P04114  P04114  P04114  P04114  P04114  P04114  P04114  P04114  P04114  P04114  P04114  P04114  P04114  P04114  P04114  P04114  P04114  P04114  P04114  P04114  P04114  P04114  P04114  P04114  P04114  P04114  P04114  P04114  P04114  P04114  P04114  P04114  P04114  P04114  P04114  P04114  P04114  P04114  P04114  P04114  P04114  P04114  P04114  P04114  P04114  P04114  P04114  P04114  P04179  P04179  P04179  P04179  P04179  P04179  P04179  P04637  P04637  P04637  P04637  P04637  P04637  P04637  P04637  P05141  P05141  P05141  P05141  P05141  P05141  P05141  P05141  P05141  P05141  P05141  P05141  P05141  P05181  P05181  P05181  P05181  P05181  P05181  P05181  P05181  P05181  P05181  P05181  P05181  P05181  P05181  P05230  P05230  P05230  P06576  P06576  P06576  P06576  P06576  P06748  P06748  P06748  P07195  P07195  P07195  P07195  P07195  P07311  P07339  P07339  P07339  P07339  P07339  P07339  P07339  P07339  P07339  P07339  P07339  P07339  P07339  P07339  P07339  P07339  P07339  P07339  P07355  P07355  P07355  P07355  P07355  P07355  P07355  P07355  P07355  P07355  P07355  P07355  P07355  P07355  P07355  P07355  P07355  P07355  P07737  P07737  P07737  P07900  P07900  P07900  P07900  P07900  P07900  P07900  P07900  P07900  P07900  P07900  P07900  P07900  P07900  P07900  P07900  P07900  P07900  P07900  P07900  P07900  P07910  P07910  P07910  P07910  P07910  P07910  P07910  P07954  P07954  P07954  P07954  P07954  P07954  P07954  P07954  P08133  P08133  P08133  P08133  P08133  P08133  P08133  P08133  P08133  P08133  P08133  P08133  P08133  P08133  P08133  P08133  P08133  P08133  P08133  P08133  P08133  P08133  P08572  P08572  P08572  P08572  P08572  P08572  P08572  P08572  P08572  P08572  P08572  P08572  P08572  P08572  P08572  P08572  P08572  P08572  P08572  P08572  P08572  P08572  P08572  P08572  P08572  P08572  P08590  P08590  P08670  P08670  P08670  P08670  P08670  P08670  P08670  P08670  P08670  P08670  P09211  P09211  P09211  P09211  P09211  P09211  P09211  P09211  P09211  P09211  P09211  P09237  P09237  P09237  P09237  P09237  P09237  P09237  P09237  P09237  P09237  P09429  P09429  P09429  P09429  P09429  P09429  P10153  P10153  P10153  P10276  P10276  P10276  P10276  P10276  P10276  P10276  P10276  P10276  P10768  P10768  P10768  P10768  P10768  P10768  P10768  P10768  P10768  P10768  P10768  P10768  P10768  P10809  P10809  P10809  P10809  P10809  P11142  P11142  P11142  P11142  P11142  P11142  P11142  P11142  P11142  P11413  P11413  P11413  P11413  P11413  P11413  P11413  P11413  P11413  P11413  P11413  P11413  P11413  P11413  P11413  P11413  P11413  P11413  P11413  P11413  P11586  P11586  P11586  P11586  P11586  P11586  P11586  P11586  P11586  P11586  P11586  P11586  P11586  P11586  P11678  P11678  P11678  P11678  P11678  P11678  P11678  P11678  P11678  P11678  P11678  P11678  P11678  P11678  P11678  P12004  P12004  P12004  P12004  P12004  P12004  P12724  P12724  P12724  P13010  P13010  P13010  P13010  P13010  P13010  P13010  P13010  P13010  P13010  P13010  P13010  P13073  P13073  P13073  P13073  P13639  P13639  P13639  P13639  P13639  P13639  P13639  P13639  P13639  P13639  P13639  P13639  P13639  P13639  P13639  P13639  P13639  P13639  P13796  P13796  P13796  P13796  P13796  P13796  P13796  P13796  P13796  P13796  P13796  P13796  P13796  P13796  P13796  P13796  P14618  P14618  P14618  P14618  P14618  P14618  P14618  P14625  P14625  P14625  P14625  P14625  P14625  P14625  P14625  P14625  P14625  P14625  P14625  P14625  P14625  P14625  P14625  P14625  P14625  P14625  P14625  P14625  P14625  P14625  P14649  P14649  P14649  P14902  P14902  P14902  P14902  P14902  P14902  P14902  P14902  P14902  P15311  P15311  P15311  P15311  P15311  P15311  P15311  P15311  P15311  P15311  P15311  P15311  P15311  P15311  P15311  P15311  P15311  P15311  P15880  P15880  P15880  P15880  P15880  P15880  P16284  P16284  P16284  P16284  P16284  P16284  P16284  P16284  P16284  P16284  P16284  P16284  P16284  P16284  P16284  P16284  P16284  P16284  P16284  P16284  P16284  P18669  P18669  P18669  P18669  P18669  P19338  P19338  P19338  P19338  P19338  P19338  P19652  P19652  P19652  P19784  P19784  P19784  P19784  P19784  P19784  P19784  P19784  P19784  P19784  P19784  P19784  P19784  P19784  P19784  P19784  P19784  P19784  P19784  P19784  P19784  P19784  P20618  P20618  P20618  P20618  P20618  P20618  P20618  P20618  P20618  P20618  P20700  P20700  P20700  P20700  P20700  P20700  P20700  P20700  P20700  P20700  P20700  P20813  P20813  P20813  P20813  P20813  P20813  P21333  P21333  P21333  P21333  P21333  P21333  P21333  P21333  P21333  P21333  P21333  P21333  P21333  P21333  P21333  P21333  P21333  P21333  P21333  P21333  P21333  P21333  P21333  P21333  P21333  P21333  P21333  P21333  P21333  P21333  P21333  P21333  P21333  P21333  P21333  P21333  P21333  P21333  P21333  P21333  P21333  P21333  P21333  P21333  P21333  P21333  P21333  P21333  P21333  P21333  P21333  P21333  P21333  P21333  P21333  P21333  P21333  P21333  P21333  P21333  P21333  P21333  P21333  P21333  P21333  P21333  P21333  P21333  P21333  P21333  P21333  P21333  P21333  P21333  P21333  P21333  P21333  P21333  P21333  P21333  P21333  P21333  P21333  P22102  P22102  P22102  P22102  P22102  P22102  P22102  P22102  P22102  P22102  P22102  P22102  P22102  P22234  P22234  P22234  P22234  P22234  P22234  P23193  P23193  P23193  P23246  P23246  P23246  P23246  P23246  P23246  P23246  P23246  P23246  P23246  P23246  P23246  P23246  P23528  P23528  P23528  P23528  P24071  P24071  P24071  P24071  P24071  P24071  P24071  P24071  P24071  P24071  P24071  P24666  P24666  P24666  P24666  P24666  P24666  P25205  P25205  P25205  P25205  P25205  P25205  P25205  P25205  P25205  P25205  P25205  P25205  P25205  P25205  P25205  P25205  P25205  P25205  P25205  P25205  P25205  P25205  P25205  P25205  P25205  P25705  P25705  P25705  P25705  P25705  P25705  P25705  P25705  P25705  P25705  P25705  P25705  P25705  P25705  P25963  P25963  P25963  P25963  P25963  P25963  P26038  P26038  P26038  P26038  P26038  P26038  P26038  P26038  P26038  P26038  P26038  P26038  P26599  P26599  P26599  P26599  P26599  P26599  P26599  P26599  P26639  P26639  P26639  P26639  P26639  P26639  P26639  P26639  P26639  P26639  P26639  P26639  P26639  P26639  P26639  P26639  P26639  P26639  P26639  P26639  P26639  P26639  P26639  P26639  P27361  P27361  P27361  P27361  P27361  P27361  P27361  P27361  P27361  P27361  P27361  P27361  P27361  P27361  P27361  P27361  P27361  P27797  P27797  P27797  P27797  P27797  P27797  P27797  P27797  P27797  P27797  P27797  P27797  P27797  P27816  P27816  P27816  P27816  P27816  P27824  P27824  P27824  P27824  P27824  P27824  P27824  P27824  P27824  P27824  P27824  P27824  P27824  P27824  P28072  P28072  P28072  P28072  P28072  P28072  P28072  P28072  P29372  P29372  P29372  P29372  P29372  P29372  P29372  P29474  P29474  P29474  P29474  P29474  P29474  P29474  P29474  P29474  P29474  P29474  P29692  P29692  P29692  P30086  P30086  P30086  P30086  P30086  P30086  P30086  P30086  P30101  P30101  P30101  P30101  P30101  P30101  P30101  P30101  P30101  P30101  P30101  P30101  P30101  P30101  P30101  P30101  P30101  P31948  P31948  P31948  P31948  P31948  P31948  P31948  P31948  P31948  P31948  P31948  P31948  P31948  P31948  P31948  P31948  P31948  P31948  P31948  P31948  P31948  P31948  P31948  P31948  P31948  P31948  P31948  P31948  P32969  P32969  P32969  P33993  P33993  P33993  P33993  P33993  P33993  P33993  P33993  P33993  P33993  P33993  P33993  P33993  P33993  P33993  P33993  P33993  P33993  P33993  P33993  P33993  P33993  P33993  P33993  P34913  P34913  P34913  P34913  P34913  P34913  P34913  P34913  P34913  P34913  P34913  P34932  P34932  P34932  P34932  P34932  P34932  P34932  P34932  P34932  P34932  P34932  P34932  P34932  P34932  P34932  P34932  P34932  P34932  P34932  P34932  P34932  P34932  P35080  P35080  P35080  P35080  P35080  P35080  P35228  P35228  P35228  P35228  P35228  P35228  P35228  P35228  P35228  P35228  P35228  P35228  P35228  P35228  P35228  P35228  P35228  P35228  P35228  P35228  P35228  P35228  P35228  P35228  P35228  P35228  P35228  P35228  P35228  P35228  P35228  P35228  P35228  P35247  P35247  P35250  P35250  P35250  P35250  P35250  P35250  P35250  P35250  P35520  P35520  P35520  P35520  P35520  P35520  P35520  P35606  P35606  P35606  P35606  P35606  P35606  P35606  P35606  P35606  P35606  P35606  P35606  P35606  P35606  P35606  P35606  P35606  P35606  P35606  P35606  P35606  P35606  P35606  P35606  P35606  P35606  P35606  P35606  P35606  P35606  P35606  P35637  P35637  P35637  P35637  P35637  P35637  P35637  P35637  P35637  P35637  P35637  P35637  P35637  P35637  P35637  P35637  P35637  P35637  P35637  P35637  P35637  P35637  P35637  P35637  P35637  P35637  P35637  P35637  P35637  P35637  P35637  P35637  P35637  P35637  P35637  P35998  P35998  P35998  P35998  P35998  P35998  P35998  P35998  P35998  P36578  P36578  P36578  P36578  P36578  P36578  P36578  P36776  P36776  P36776  P36776  P36776  P36776  P36776  P36776  P36776  P36776  P36776  P36776  P36776  P36776  P36776  P36776  P36776  P36776  P36776  P36776  P36776  P36776  P36776  P36959  P36959  P36959  P36959  P36959  P36959  P36959  P37802  P37802  P37802  P37802  P37837  P37837  P37837  P37837  P37837  P37837  P37837  P37837  P38159  P38159  P38159  P38159  P38159  P38159  P38159  P38159  P38159  P38159  P38159  P38159  P38159  P38159  P38159  P38159  P38159  P38159  P38159  P38159  P38159  P38159  P38159  P38159  P38159  P38159  P38646  P38646  P38646  P38646  P38646  P38646  P38646  P38646  P38646  P40926  P40926  P41252  P41252  P41252  P41252  P41252  P41252  P41252  P41252  P41252  P41252  P41252  P41252  P41252  P41252  P41252  P41252  P41252  P41252  P41252  P41252  P41252  P41252  P41252  P41252  P41252  P41252  P41252  P41252  P41252  P41252  P41252  P41252  P41252  P41252  P41252  P41252  P41252  P41252  P41252  P41252  P41252  P41252  P41252  P41252  P41252  P41252  P41252  P41252  P41252  P41252  P41252  P42704  P42704  P42704  P42704  P42704  P42704  P42704  P42704  P42704  P42704  P42704  P42704  P42704  P42704  P42704  P42704  P42704  P42704  P42704  P42704  P42704  P42704  P42704  P42704  P42704  P42704  P42704  P42704  P42704  P42704  P42704  P42704  P42704  P42704  P42704  P42704  P42704  P42704  P42704  P42704  P42704  P42704  P42704  P42704  P42704  P42704  P42704  P43243  P43243  P43243  P43243  P43243  P43243  P43243  P43243  P43243  P43243  P43243  P43243  P43243  P43243  P43243  P43243  P43243  P43243  P43243  P43243  P43243  P43243  P43243  P43243  P43243  P43243  P45452  P45452  P45452  P45452  P45452  P45452  P45452  P45452  P45452  P45452  P45452  P45452  P45452  P45452  P45452  P45452  P45452  P45452  P45452  P45452  P46459  P46459  P46459  P46459  P46459  P46459  P46459  P46459  P46459  P46779  P46779  P46779  P47813  P47813  P47813  P49207  P49207  P49207  P49207  P49321  P49321  P49321  P49321  P49321  P49321  P49321  P49321  P49321  P49321  P49368  P49368  P49368  P49368  P49368  P49368  P49368  P49458  P49458  P49458  P49588  P49588  P49588  P49588  P49588  P49588  P49588  P49588  P49588  P49588  P49588  P49588  P49588  P49588  P49588  P49588  P49588  P49588  P49588  P49588  P49588  P49588  P49588  P49588  P49588  P50395  P50395  P50395  P50395  P50395  P50395  P50395  P50395  P50395  P50395  P50395  P50395  P50395  P50395  P50395  P50395  P50395  P50395  P50395  P50395  P50395  P50395  P50395  P50395  P50990  P50990  P50990  P50990  P50990  P50990  P50990  P50990  P50990  P50990  P50990  P50990  P50990  P50990  P50990  P50990  P50990  P50990  P50990  P50990  P50990  P50990  P50990  P50990  P50990  P50990  P50990  P50990  P50990  P50990  P50990  P50990  P50990  P50990  P50990  P50990  P50990  P50990  P50990  P50990  P50990  P50990  P50990  P50990  P50990  P50990  P50990  P50990  P50990  P50991  P50991  P50991  P50991  P51149  P51149  P51149  P51149  P51149  P52272  P52272  P52272  P52272  P52566  P52566  P52566  P52566  P52566  P52566  P52566  P53041  P53041  P53041  P53041  P53041  P53041  P53041  P53041  P53041  P53041  P53041  P53041  P53041  P53041  P53041  P53041  P53041  P53041  P53041  P53041  P53041  P53041  P53041  P53041  P53041  P53041  P53396  P53396  P53396  P53396  P53396  P53396  P53396  P53396  P53396  P53396  P53396  P53396  P53396  P53396  P53396  P53396  P53396  P53396  P53396  P53396  P53396  P53396  P53396  P53396  P53396  P53396  P53396  P53396  P53396  P53396  P53396  P53396  P53396  P53396  P53396  P53396  P53396  P55072  P55072  P55072  P55072  P55072  P55072  P55072  P55072  P55072  P55072  P55072  P55072  P55769  P55769  P55884  P55884  P55884  P55884  P55884  P55884  P55884  P55884  P55884  P55884  P55884  P55884  P55884  P55884  P55884  P55884  P55884  P55884  P60468  P60842  P60842  P60842  P60842  P60842  P60842  P60842  P60842  P60842  P60842  P61088  P61088  P61088  P61158  P61158  P61158  P61158  P61158  P61158  P61158  P61158  P61158  P61158  P61158  P61158  P61158  P61158  P61158  P61158  P61158  P61221  P61221  P61221  P61221  P61221  P61221  P61221  P61221  P61221  P61221  P61221  P61221  P61221  P61221  P61221  P61221  P61221  P61221  P61247  P61247  P61247  P61247  P61604  P61604  P61927  P61927  P61927  P61978  P61978  P61978  P61978  P61978  P61978  P61978  P61978  P61978  P61978  P61978  P61978  P61978  P61978  P61978  P62136  P62136  P62136  P62136  P62136  P62136  P62136  P62136  P62136  P62136  P62136  P62136  P62195  P62195  P62195  P62195  P62195  P62195  P62241  P62241  P62241  P62241  P62241  P62241  P62241  P62241  P62249  P62249  P62249  P62249  P62249  P62249  P62249  P62273  P62273  P62280  P62280  P62280  P62280  P62280  P62280  P62701  P62701  P62701  P62701  P62750  P62750  P62750  P62805  P62826  P62826  P62826  P62826  P62826  P62826  P62826  P62910  P62910  P62917  P62917  P62917  P62917  P62917  P62993  P62993  P62993  P62993  P62993  P62993  P63220  P63244  P63244  P63244  P63244  P63244  P67775  P67775  P67775  P67775  P67775  P67775  P67775  P67775  P67775  P67775  P67775  P67775  P67775  P67775  P67775  P68104  P68104  P68104  P68104  P68104  P68104  P68104  P68104  P68104  P68104  P68871  P78344  P78344  P78344  P78344  P78344  P78344  P78344  P78344  P78344  P78344  P78344  P78344  P78371  P78371  P78371  P78371  P78371  P78417  P78417  P78417  P78417  P78417  P78417  P78417  P78417  P83731  P83731  P83731  P84103  P84103  P84103  Q00325  Q00325  Q00325  Q00325  Q00325  Q00325  Q00325  Q00325  Q00325  Q00325  Q00325  Q00325  Q00325  Q00325  Q00325  Q00610  Q00610  Q00610  Q00610  Q00610  Q00610  Q00610  Q00610  Q00610  Q00610  Q00610  Q00610  Q00610  Q00610  Q00610  Q00610  Q00610  Q00610  Q00610  Q00610  Q00610  Q00610  Q00610  Q00610  Q00610  Q00610  Q00610  Q00610  Q00610  Q00610  Q00610  Q00610  Q00610  Q00610  Q00610  Q00610  Q00610  Q00610  Q00610  Q00610  Q00610  Q00610  Q00610  Q00610  Q00610  Q00610  Q00610  Q00610  Q00610  Q00610  Q00610  Q00610  Q00610  Q00610  Q00610  Q00610  Q00610  Q00610  Q00610  Q00610  Q00610  Q00610  Q00610  Q00610  Q00610  Q00839  Q00839  Q00839  Q00839  Q00839  Q00839  Q00839  Q00839  Q00839  Q00839  Q00839  Q00839  Q00839  Q00839  Q00839  Q00839  Q00839  Q00839  Q00839  Q00839  Q00839  Q00839  Q00839  Q00839  Q00839  Q00839  Q01130  Q01130  Q01130  Q01130  Q01130  Q01130  Q01130  Q01130  Q01518  Q01518  Q01518  Q01518  Q01518  Q01518  Q01518  Q01518  Q01518  Q01518  Q01518  Q02543  Q02543  Q02543  Q02543  Q02543  Q02543  Q02543  Q02790  Q02790  Q02790  Q02790  Q02790  Q02790  Q02790  Q02790  Q02790  Q02790  Q02790  Q02790  Q02790  Q02790  Q02790  Q02878  Q02878  Q02878  Q02878  Q02878  Q02878  Q02878  Q02878  Q02878  Q03405  Q04206  Q04206  Q04206  Q04206  Q04206  Q04206  Q04206  Q04206  Q04206  Q04206  Q04837  Q04837  Q04837  Q04837  Q04837  Q06547  Q06547  Q06547  Q06547  Q06830  Q06830  Q06830  Q06830  Q07955  Q07955  Q07955  Q07955  Q07955  Q07955  Q07955  Q07955  Q07955  Q07955  Q07955  Q07955  Q08170  Q08170  Q08170  Q08170  Q08170  Q08170  Q08170  Q08170  Q08170  Q08170  Q08170  Q08170  Q08170  Q08211  Q08211  Q08211  Q08211  Q08211  Q08211  Q08211  Q08211  Q08211  Q08211  Q08211  Q08211  Q08211  Q08211  Q08211  Q08211  Q08211  Q08211  Q08211  Q08211  Q08211  Q08211  Q08211  Q08211  Q08211  Q08211  Q08211  Q08211  Q08211  Q08211  Q08211  Q08211  Q08211  Q08211  Q08211  Q08211  Q08211  Q08211  Q08211  Q08211  Q08211  Q08211  Q08211  Q08211  Q08211  Q08211  Q08211  Q08J23  Q08J23  Q08J23  Q08J23  Q08J23  Q08J23  Q08J23  Q08J23  Q08J23  Q08J23  Q08J23  Q08J23  Q08J23  Q08J23  Q08J23  Q09028  Q09028  Q09028  Q09028  Q09028  Q09028  Q09028  Q12906  Q12906  Q12906  Q12906  Q12906  Q12906  Q12906  Q12906  Q12906  Q12906  Q12906  Q12906  Q12906  Q12906  Q12906  Q12906  Q12906  Q12906  Q12906  Q12906  Q12906  Q12906  Q12906  Q12906  Q12906  Q12906  Q12906  Q12906  Q12906  Q12906  Q12906  Q12906  Q12906  Q12906  Q12906  Q12906  Q12906  Q12906  Q12906  Q12906  Q12906  Q12906  Q12906  Q12906  Q12906  Q12906  Q12906  Q12906  Q12906  Q12906  Q12906  Q12906  Q12906  Q12906  Q12906  Q12906  Q12906  Q12906  Q12906  Q12906  Q12906  Q12906  Q12906  Q12906  Q12906  Q12906  Q12906  Q12906  Q12906  Q12906  Q12996  Q12996  Q12996  Q12996  Q12996  Q12996  Q12996  Q12996  Q12996  Q12996  Q12996  Q12996  Q12996  Q12996  Q12996  Q12996  Q12996  Q12996  Q12996  Q12996  Q12996  Q12996  Q12996  Q12996  Q12996  Q12996  Q12996  Q12996  Q12996  Q12996  Q12996  Q12996  Q12996  Q12996  Q12996  Q12996  Q12996  Q12996  Q12996  Q12996  Q13148  Q13148  Q13148  Q13148  Q13148  Q13148  Q13148  Q13237  Q13237  Q13237  Q13237  Q13237  Q13237  Q13237  Q13237  Q13237  Q13237  Q13237  Q13237  Q13237  Q13237  Q13237  Q13237  Q13237  Q13237  Q13237  Q13237  Q13237  Q13237  Q13237  Q13237  Q13257  Q13257  Q13257  Q13257  Q13257  Q13257  Q13263  Q13263  Q13263  Q13263  Q13263  Q13263  Q13263  Q13263  Q13347  Q13347  Q13347  Q13347  Q13347  Q13347  Q13347  Q13347  Q13347  Q13347  Q13451  Q13451  Q13451  Q13451  Q13451  Q13451  Q13451  Q13451  Q13451  Q13451  Q13451  Q13451  Q13451  Q13451  Q13451  Q13547  Q13547  Q13547  Q13547  Q13547  Q13547  Q13547  Q13547  Q13547  Q13547  Q13547  Q13547  Q13547  Q13547  Q13547  Q13547  Q13547  Q13547  Q13547  Q13547  Q13547  Q13547  Q13547  Q13547  Q13547  Q13547  Q13547  Q13573  Q13573  Q13573  Q13573  Q13573  Q13573  Q13573  Q13573  Q13573  Q13573  Q13573  Q13573  Q13573  Q13573  Q13765  Q13813  Q13813  Q13813  Q13813  Q13813  Q13813  Q13813  Q13813  Q13813  Q13813  Q13813  Q13813  Q13813  Q13813  Q13813  Q13813  Q13813  Q13813  Q13813  Q13813  Q13813  Q13813  Q13813  Q13813  Q13813  Q13813  Q13813  Q13813  Q13813  Q13813  Q13813  Q13813  Q13813  Q13813  Q13813  Q13936  Q13936  Q13936  Q13936  Q13936  Q13936  Q13936  Q13936  Q13936  Q13936  Q13936  Q13936  Q13936  Q13936  Q13936  Q13936  Q13936  Q13936  Q13936  Q13936  Q13936  Q13936  Q13936  Q13936  Q13936  Q13936  Q13936  Q13936  Q13936  Q13936  Q13936  Q13936  Q13936  Q13936  Q13936  Q13936  Q13936  Q13936  Q13936  Q13936  Q13936  Q13936  Q13936  Q13936  Q13936  Q13936  Q13936  Q13936  Q13936  Q13936  Q13936  Q13936  Q13936  Q13936  Q13936  Q13936  Q13936  Q13936  Q13976  Q13976  Q13976  Q13976  Q13976  Q13976  Q13976  Q13976  Q13976  Q13976  Q13976  Q13976  Q13976  Q13976  Q13976  Q13976  Q13976  Q13976  Q13976  Q14204  Q14204  Q14204  Q14204  Q14204  Q14204  Q14204  Q14204  Q14204  Q14204  Q14204  Q14204  Q14204  Q14204  Q14204  Q14204  Q14204  Q14204  Q14204  Q14204  Q14204  Q14204  Q14204  Q14204  Q14204  Q14204  Q14204  Q14204  Q14204  Q14204  Q14204  Q14204  Q14204  Q14204  Q14204  Q14204  Q14204  Q14204  Q14204  Q14204  Q14204  Q14204  Q14204  Q14204  Q14204  Q14204  Q14204  Q14204  Q14204  Q14204  Q14204  Q14204  Q14204  Q14204  Q14204  Q14204  Q14204  Q14204  Q14204  Q14204  Q14204  Q14204  Q14204  Q14204  Q14204  Q14204  Q14204  Q14204  Q14204  Q14204  Q14204  Q14204  Q14204  Q14204  Q14204  Q14204  Q14204  Q14204  Q14204  Q14204  Q14204  Q14204  Q14204  Q14204  Q14204  Q14204  Q14204  Q14204  Q14204  Q14204  Q14204  Q14204  Q14204  Q14204  Q14204  Q14204  Q14204  Q14204  Q14204  Q14204  Q14204  Q14204  Q14204  Q14204  Q14204  Q14204  Q14204  Q14204  Q14204  Q14204  Q14204  Q14204  Q14204  Q14204  Q14204  Q14204  Q14204  Q14204  Q14204  Q14204  Q14204  Q14204  Q14204  Q14204  Q14204  Q14204  Q14566  Q14566  Q14566  Q14566  Q14566  Q14566  Q14566  Q14566  Q14566  Q14566  Q14566  Q14566  Q14566  Q14566  Q14566  Q14566  Q14566  Q14566  Q14566  Q14566  Q14566  Q14683  Q14683  Q14683  Q14683  Q14683  Q14683  Q14683  Q14683  Q14683  Q14683  Q14683  Q14683  Q14683  Q14683  Q14683  Q14683  Q14683  Q14683  Q14683  Q14683  Q14683  Q14683  Q14683  Q14683  Q14683  Q14683  Q14683  Q14683  Q14694  Q14694  Q14694  Q14694  Q14694  Q14694  Q14694  Q14694  Q14694  Q14694  Q14694  Q14694  Q14694  Q14694  Q14694  Q14694  Q14694  Q14694  Q14694  Q14694  Q14694  Q14694  Q14694  Q14694  Q14694  Q14694  Q14694  Q14847  Q14847  Q14847  Q14847  Q14847  Q14847  Q14847  Q14847  Q14847  Q14847  Q14847  Q14847  Q14847  Q14847  Q14847  Q15019  Q15019  Q15019  Q15019  Q15019  Q15019  Q15019  Q15019  Q15056  Q15056  Q15056  Q15181  Q15181  Q15181  Q15181  Q15181  Q15181  Q15181  Q15181  Q15365  Q15365  Q15365  Q15365  Q15393  Q15393  Q15393  Q15393  Q15393  Q15393  Q15393  Q15393  Q15393  Q15393  Q15393  Q15393  Q15393  Q15393  Q15393  Q15393  Q15393  Q15393  Q15393  Q15393  Q15393  Q15393  Q15393  Q15393  Q15393  Q15393  Q15393  Q15393  Q15393  Q15393  Q15393  Q15393  Q15393  Q15393  Q15393  Q15393  Q15393  Q15427  Q15427  Q15427  Q15427  Q15427  Q15427  Q15427  Q15427  Q15427  Q15427  Q15428  Q15428  Q15428  Q15428  Q15428  Q15428  Q15459  Q15459  Q15459  Q15459  Q15459  Q15459  Q15459  Q15459  Q15459  Q15459  Q15459  Q15459  Q15459  Q15459  Q16595  Q16595  Q16595  Q16595  Q16595  Q16629  Q16629  Q16629  Q16629  Q16629  Q16629  Q16629  Q16629  Q16629  Q16629  Q16647  Q16647  Q16647  Q16647  Q16647  Q16647  Q16647  Q16647  Q16647  Q16647  Q16647  Q16647  Q16647  Q16647  Q16647  Q16647  Q16695  Q16695  Q16891  Q16891  Q16891  Q16891  Q16891  Q16891  Q16891  Q16891  Q16891  Q16891  Q16891  Q16891  Q16891  Q16891  Q3MHD2  Q3MHD2  Q3MHD2  Q3MHD2  Q52LJ0  Q52LJ0  Q52LJ0  Q52LJ0  Q52LJ0  Q52LJ0  Q52LJ0  Q58FF8  Q58FF8  Q58FF8  Q58FF8  Q58FF8  Q58FF8  Q58FF8  Q58FF8  Q58FF8  Q58FF8  Q58FF8  Q58FF8  Q5JTV8  Q5JTV8  Q5JTV8  Q5JTV8  Q5JTV8  Q5JTV8  Q5JTV8  Q5JTV8  Q5JTV8  Q5JTV8  Q5JTV8  Q5JTV8  Q5JTV8  Q6P2Q9  Q6P2Q9  Q6P2Q9  Q6P2Q9  Q6P2Q9  Q6P2Q9  Q6P2Q9  Q6P2Q9  Q6P2Q9  Q6P2Q9  Q6P2Q9  Q6P2Q9  Q6P2Q9  Q6P2Q9  Q6P2Q9  Q6P2Q9  Q6P2Q9  Q6P2Q9  Q6P2Q9  Q6P2Q9  Q6P2Q9  Q6P2Q9  Q6P2Q9  Q6P2Q9  Q6P2Q9  Q6P2Q9  Q6P2Q9  Q6P2Q9  Q6P2Q9  Q6P2Q9  Q6P2Q9  Q6P2Q9  Q6P2Q9  Q6P2Q9  Q6P2Q9  Q6P2Q9  Q6P2Q9  Q6P2Q9  Q6P2Q9  Q6P2Q9  Q6P2Q9  Q6P2Q9  Q6P2Q9  Q6P2Q9  Q6P2Q9  Q6P2Q9  Q6P2Q9  Q6P2Q9  Q6P2Q9  Q6P2Q9  Q6P2Q9  Q6P2Q9  Q6P2Q9  Q6P2Q9  Q6P2Q9  Q6P2Q9  Q6P2Q9  Q6P2Q9  Q6P2Q9  Q6P2Q9  Q6P2Q9  Q6P2Q9  Q6P2Q9  Q6P2Q9  Q6P2Q9  Q6P2Q9  Q6P2Q9  Q6P2Q9  Q6P2Q9  Q6P2Q9  Q6P2Q9  Q6P2Q9  Q6P2Q9  Q6P2Q9  Q6P2Q9  Q6P2Q9  Q6P2Q9  Q6P2Q9  Q6P2Q9  Q6P2Q9  Q6P2Q9  Q6P2Q9  Q6P2Q9  Q6P2Q9  Q6P2Q9  Q6P2Q9  Q6P2Q9  Q6P2Q9  Q6P2Q9  Q6P2Q9  Q6P2Q9  Q6P2Q9  Q6P2Q9  Q6P2Q9  Q6P2Q9  Q6P2Q9  Q6P2Q9  Q6P2Q9  Q6P2Q9  Q6P2Q9  Q6P2Q9  Q6P2Q9  Q6P2Q9  Q71U36  Q71U36  Q71U36  Q71U36  Q71U36  Q71U36  Q71U36  Q71U36  Q71U36  Q71UM5  Q7KZF4  Q7KZF4  Q7KZF4  Q7KZF4  Q7KZF4  Q7KZF4  Q7KZF4  Q7KZF4  Q7KZF4  Q7KZF4  Q7KZF4  Q7KZF4  Q7KZF4  Q7KZF4  Q7KZF4  Q7KZF4  Q7KZF4  Q7KZF4  Q7KZF4  Q7KZF4  Q7KZF4  Q7KZF4  Q7KZF4  Q7KZF4  Q7KZF4  Q7KZF4  Q7KZF4  Q7L014  Q7L014  Q7L014  Q7L014  Q7L014  Q7L014  Q7L014  Q7L014  Q7L014  Q7L014  Q7L014  Q7L014  Q7L014  Q7L014  Q7L014  Q7L014  Q7L014  Q7L014  Q7L014  Q7L014  Q7L014  Q7L014  Q7L014  Q7L014  Q86VP6  Q86VP6  Q86VP6  Q86VP6  Q86VP6  Q86VP6  Q86VP6  Q86VP6  Q86VP6  Q86VP6  Q86VP6  Q86VP6  Q86VP6  Q86VP6  Q86VP6  Q86VP6  Q86VP6  Q86VP6  Q86VP6  Q86VP6  Q86VP6  Q86VP6  Q86VP6  Q86VP6  Q86VP6  Q86VP6  Q86VP6  Q86VP6  Q86VP6  Q86VP6  Q8IWL2  Q8IWL2  Q8IWL2  Q8IWL2  Q8IXH7  Q8IXH7  Q8IXH7  Q8IXH7  Q8IXH7  Q8IXH7  Q8IXH7  Q8IXH7  Q8IXH7  Q8IXH7  Q8IXH7  Q8IXH7  Q8IXH7  Q8IXH7  Q8IXH7  Q8IXH7  Q8N684  Q8N684  Q8N684  Q8N684  Q8N684  Q8N684  Q8N684  Q8N684  Q8N684  Q8N684  Q8N684  Q8N684  Q8N684  Q8NHL6  Q8NHL6  Q8NHL6  Q8NHL6  Q8NHL6  Q8NHL6  Q8NHL6  Q8NHL6  Q8NHL6  Q8NHL6  Q8NHL6  Q8NHL6  Q8NHL6  Q8NHL6  Q8NHL6  Q8NHL6  Q8NHL6  Q8NHL6  Q92526  Q92526  Q92526  Q92526  Q92526  Q92526  Q92526  Q92526  Q92900  Q92900  Q92900  Q92900  Q92900  Q92900  Q92900  Q92900  Q92900  Q92900  Q92900  Q92900  Q92900  Q92900  Q92900  Q92900  Q92900  Q92900  Q92900  Q92900  Q92900  Q92900  Q92900  Q92900  Q92900  Q92900  Q92900  Q92900  Q92900  Q92900  Q92900  Q92900  Q92900  Q92900  Q92900  Q92900  Q92900  Q92922  Q92922  Q92922  Q92922  Q92922  Q92922  Q92922  Q92922  Q92922  Q92922  Q92922  Q92922  Q92922  Q92922  Q92922  Q92922  Q92922  Q92922  Q92922  Q92922  Q92922  Q92922  Q92922  Q92922  Q92922  Q92922  Q96AE4  Q96AE4  Q96AE4  Q96AE4  Q96AE4  Q96AE4  Q96AE4  Q96AE4  Q96AE4  Q96AE4  Q96AE4  Q96AE4  Q96AE4  Q96AE4  Q96AE4  Q96AE4  Q96AE4  Q96AE4  Q96AE4  Q96AE4  Q96AE4  Q96AE4  Q96IX5  Q96IX5  Q96IX5  Q96IX5  Q96PK6  Q96PK6  Q96PK6  Q96PK6  Q96PK6  Q96PK6  Q96PK6  Q96PK6  Q96PK6  Q96PK6  Q96PK6  Q96PK6  Q96PK6  Q96PK6  Q96PK6  Q96PK6  Q96PK6  Q96PK6  Q96PK6  Q96PK6  Q96PK6  Q96PK6  Q96PK6  Q96PK6  Q96PK6  Q96PK6  Q96PK6  Q96PK6  Q96PK6  Q96PK6  Q96PK6  Q96PK6  Q96PK6  Q96PK6  Q96PK6  Q96PK6  Q96PK6  Q96PK6  Q96PK6  Q96PK6  Q96PK6  Q96PK6  Q96PK6  Q96PK6  Q96PK6  Q96PK6  Q96QR8  Q96QR8  Q96QR8  Q96QR8  Q96QR8  Q96QR8  Q96QR8  Q96QR8  Q96QR8  Q99614  Q99614  Q99614  Q99614  Q99614  Q99614  Q99727  Q99727  Q99727  Q99727  Q99727  Q99727  Q99727  Q99832  Q99832  Q99832  Q99832  Q99832  Q99832  Q99832  Q99832  Q99832  Q99832  Q9BUJ2  Q9BUJ2  Q9BUJ2  Q9BUJ2  Q9BUJ2  Q9BUJ2  Q9BUJ2  Q9BUJ2  Q9BUJ2  Q9BUJ2  Q9BUJ2  Q9BUJ2  Q9BUJ2  Q9BUJ2  Q9BUJ2  Q9BUJ2  Q9BUJ2  Q9BUJ2  Q9BUJ2  Q9BUJ2  Q9BUJ2  Q9BUJ2  Q9BUJ2  Q9BUJ2  Q9BUJ2  Q9BUJ2  Q9BUJ2  Q9BUJ2  Q9BUJ2  Q9BUJ2  Q9BUJ2  Q9BUJ2  Q9BUJ2  Q9BUJ2  Q9BUJ2  Q9BUJ2  Q9BUJ2  Q9BUJ2  Q9BUJ2  Q9BUJ2  Q9BUJ2  Q9BUJ2  Q9BUJ2  Q9BUJ2  Q9BUJ2  Q9BUJ2  Q9BUJ2  Q9BWJ5  Q9BWJ5  Q9BWJ5  Q9BWJ5  Q9BZK7  Q9BZK7  Q9BZK7  Q9BZK7  Q9BZK7  Q9BZK7  Q9BZK7  Q9BZK7  Q9H074  Q9H074  Q9H074  Q9H074  Q9H074  Q9H074  Q9H074  Q9H074  Q9H074  Q9H074  Q9H074  Q9H074  Q9H074  Q9H074  Q9H074  Q9H074  Q9H074  Q9H074  Q9H0D6  Q9H0D6  Q9H0D6  Q9H0D6  Q9H0D6  Q9H0D6  Q9H0D6  Q9H0D6  Q9H0D6  Q9H0D6  Q9H0D6  Q9H0D6  Q9H0D6  Q9H0D6  Q9H0D6  Q9H0D6  Q9H0D6  Q9H0D6  Q9H0D6  Q9H0D6  Q9H0D6  Q9H0D6  Q9H0D6  Q9H0D6  Q9H0D6  Q9H0D6  Q9H0D6  Q9H0D6  Q9H0D6  Q9H0D6  Q9H0D6  Q9H0D6  Q9H0D6  Q9H3K2  Q9H3K2  Q9H3K2  Q9H3K2  Q9H3K2  Q9H3K2  Q9H3K2  Q9H3K2  Q9H3K2  Q9H3K2  Q9H3K2  Q9H3K2  Q9H3K2  Q9NPD3  Q9NPD3  Q9NPD3  Q9NPD3  Q9NPD3  Q9NR30  Q9NR30  Q9NR30  Q9NR30  Q9NR30  Q9NR30  Q9NR30  Q9NR30  Q9NR30  Q9NR30  Q9NR30  Q9NR45  Q9NR45  Q9NR45  Q9NR45  Q9NR45  Q9NR45  Q9NR45  Q9NR45  Q9NR45  Q9NUV9  Q9NUV9  Q9NUV9  Q9NUV9  Q9NUV9  Q9NUV9  Q9NUV9  Q9NUV9  Q9NYF8  Q9NYF8  Q9NYF8  Q9NYF8  Q9NYF8  Q9NYF8  Q9NYF8  Q9NYF8  Q9NYF8  Q9NYF8  Q9NYF8  Q9NYF8  Q9NYF8  Q9NYF8  Q9NYF8  Q9NYF8  Q9NYF8  Q9NYF8  Q9NYF8  Q9NYF8  Q9NYF8  Q9NYF8  Q9NYF8  Q9NYF8  Q9NYF8  Q9NYF8  Q9NYF8  Q9NYF8  Q9NYF8  Q9NYF8  Q9NYF8  Q9NZ32  Q9NZ32  Q9NZ32  Q9NZ32  Q9NZ32  Q9NZ32  Q9NZ32  Q9NZ32  Q9NZ32  Q9NZ32  Q9NZ32  Q9NZ32  Q9P2J5  Q9P2J5  Q9P2J5  Q9P2J5  Q9P2J5  Q9P2J5  Q9P2J5  Q9P2J5  Q9P2J5  Q9P2J5  Q9P2J5  Q9P2J5  Q9P2J5  Q9P2J5  Q9P2J5  Q9P2J5  Q9P2J5  Q9P2J5  Q9P2J5  Q9P2J5  Q9P2J5  Q9P2J5  Q9P2J5  Q9P2J5  Q9P2J5  Q9P2J5  Q9P2J5  Q9P2J5  Q9P2J5  Q9P2J5  Q9P2J5  Q9P2J5  Q9P2J5  Q9P2J5  Q9P2J5  Q9P2J5  Q9P2J5  Q9P2J5  Q9P2J5  Q9P2J5  Q9P2J5  Q9UHB9  Q9UHB9  Q9UHB9  Q9UHB9  Q9UHB9  Q9UHB9  Q9UHB9  Q9UHB9  Q9UHB9  Q9UHB9  Q9UHB9  Q9UHB9  Q9UHB9  Q9UHB9  Q9UHB9  Q9UHB9  Q9UHB9  Q9UHB9  Q9UHB9  Q9UHD1  Q9UHD1  Q9UHD1  Q9UHD1  Q9UHD1  Q9UHD1  Q9UI10  Q9UI10  Q9UI10  Q9UI10  Q9UI10  Q9UI10  Q9UI10  Q9UI10  Q9UI10  Q9UI10  Q9UI10  Q9UI10  Q9UKK9  Q9UKK9  Q9UKK9  Q9UKK9  Q9UKK9  Q9UKK9  Q9UKY7  Q9UKY7  Q9UKY7  Q9UKY7  Q9UL46  Q9UL46  Q9UL46  Q9UL46  Q9UL46  Q9UL46  Q9UL46  Q9UN86  Q9UN86  Q9UN86  Q9UN86  Q9UN86  Q9UN86  Q9UN86  Q9UN86  Q9UN86  Q9UN86  Q9UNZ2  Q9UNZ2  Q9UNZ2  Q9UNZ2  Q9Y230  Q9Y230  Q9Y230  Q9Y230  Q9Y230  Q9Y230  Q9Y230  Q9Y230  Q9Y285  Q9Y285  Q9Y285  Q9Y285  Q9Y285  Q9Y285  Q9Y285  Q9Y285  Q9Y285  Q9Y285  Q9Y285  Q9Y285  Q9Y2W1  Q9Y2W1  Q9Y2W1  Q9Y2W1  Q9Y2W1  Q9Y2W1  Q9Y2W1  Q9Y2W1  Q9Y2W1  Q9Y2W1  Q9Y2W1  Q9Y2W1  Q9Y2W1  Q9Y2W1  Q9Y2W1  Q9Y2W1  Q9Y2W1  Q9Y2W1  Q9Y2W1  Q9Y2W1  Q9Y2W1  Q9Y2W1  Q9Y2W1  Q9Y2W1  Q9Y2W1  Q9Y2W1  Q9Y2W1  Q9Y2W2  Q9Y2W2  Q9Y2W2  Q9Y2W2  Q9Y2W2  Q9Y2W2  Q9Y2W2  Q9Y314  Q9Y314  Q9Y314  Q9Y314  Q9Y314  Q9Y314  Q9Y314  Q9Y3F4  Q9Y3F4  Q9Y3F4  Q9Y3F4  Q9Y3F4  Q9Y3F4  Q9Y3F4  Q9Y3F4  Q9Y3F4  Q9Y3F4  Q9Y3F4  Q9Y3U8  Q9Y6A4  Q9Y6A4  Q9Y6A4  Q9Y6A4  Q9Y6A4  Q9Y6A4  Q9Y6A4  Q9Y6A4  O94892  O94892  O94892  O94892  O94892  O94892  O94892  O94892  O94892  O94892  O94892  O94892  O94892  O94892  O94892  O94892  O94892  O94892  O94892  O95470  O95470  O95470  O95470  O95470  O95470  O95470  O95470  O95470  O95470  O95470  O95470  O95470  O95470  O95470  O95470  O95470  O95470  O95470  O95470  O95470  P25787  P25787  P25787  P25787  P25787  P25787  P25787  P25787  P25787  P25787  P25787  P25787  P25787  P31321  P31321  P31321  P31321  P31321  P31321  P31321  P31321  P31321  P31321  P31321  P31321  P50502  P50502  P50502  P50502  P61513  P61513  P61513  Q13017  Q13017  Q13017  Q13017  Q13017  Q13017  Q13017  Q13017  Q13017  Q13017  Q13017  Q13017  Q13017  Q13017  Q13017  Q13017  Q13017  Q13017  Q13017  Q13017  Q13017  Q13017  Q13017  Q13017  Q13017  Q13017  Q13017  Q13017  Q13017  Q13017  Q13017  Q13017  Q13017  Q13017  Q13017  Q13017  Q13017  Q13017  Q13017  Q13017  Q13017  Q13017  Q13017  Q13017  Q13017  Q13017  Q13017  Q13017  Q13017  Q15027  Q15027  Q15027  Q15027  Q15027  Q15027  Q15027  Q15027  Q15413  Q15413  Q15413  Q15413  Q15413  Q15413  Q15413  Q15413  Q15413  Q15413  Q15413  Q15413  Q15413  Q15413  Q15413  Q15413  Q15413  Q15413  Q15413  Q15413  Q15413  Q15413  Q15413  Q15413  Q15413  Q15413  Q15413  Q15413  Q15413  Q15413  Q15413  Q15413  Q15413  Q15413  Q15413  Q15413  Q15413  Q15413  Q15413  Q15413  Q15413  Q15413  Q15413  Q15413  Q15413  Q15413  Q15413  Q15413  Q15413  Q15413  Q15413  Q15413  Q15413  Q15413  Q15413  Q15413  Q15413  Q15413  Q15413  Q15413  Q15413  Q15413  Q15413  Q15413  Q15413  Q15413  Q15413  Q15413  Q15413  Q15413  Q15413  Q15413  Q15413  Q15413  Q15413  Q15413  Q15413  Q15413  Q15413  Q15413  Q15413  Q15413  Q15413  Q15413  Q15413  Q15413  Q15413  Q15413  Q15413  Q15413  Q15413  Q15413  Q15413  Q15413  Q15413  Q15413  Q15413  Q15413  Q15413  Q15413  Q15413  Q15413  Q15413  Q15413  Q15413  Q15413  Q15413  Q15413  Q15413  Q15413  Q15413  Q15413  Q15413  Q15413  Q15413  Q15413  Q15413  Q15413  Q15413  Q15413  Q15413  Q15413  Q15413  Q15413  Q15413  Q15413  Q15413  Q15413  Q15413  Q15413  Q15413  Q15413  Q15413  Q15413  Q15413  Q15413  Q15413  Q15413  Q15413  Q15413  Q15413  Q15413  Q15413  Q15413  Q15413  Q15413  Q15413  Q15413  Q8IUC4  Q8IUC4  Q8IUC4  Q8IUC4  Q8IUC4  Q8IUC4  Q8IUC4  Q8IUC4  Q8IUC4  Q8IUC4  Q8IUC4  Q8IUC4  Q8IUC4  Q8IUC4  Q8IUC4  Q8IUC4  Q8IUC4  Q8IUC4  Q8IUC4  Q8IUC4  Q9UHA7  Q9UHA7  O00151  O00151  O00151  O00151  O00151  O00151  O00165  O00232  O00232  O00232  O00232  O00232  O00232  O00232  O00232  O00232  O00232  O00232  O00232  O00232  O00232  O00232  O00483  O00483  O00483  O14737  O14737  O14744  O14744  O14744  O14744  O14744  O14744  O14744  O14744  O14744  O14744  O14744  O14744  O14744  O14744  O14744  O14744  O14744  O14744  O14744  O14744  O14744  O14976  O14976  O14976  O14976  O14976  O14976  O14976  O14976  O14976  O14976  O14976  O14976  O14976  O14976  O14976  O14976  O14976  O14976  O14976  O14976  O14976  O14976  O14976  O14976  O14976  O14976  O14976  O14976  O14976  O14976  O14976  O14979  O14979  O14979  O14979  O14979  O14979  O14979  O14979  O14979  O14979  O14979  O14979  O14979  O14979  O14979  O14979  O14979  O14979  O14979  O14979  O14979  O14979  O14979  O43143  O43143  O43143  O43143  O43143  O43143  O43143  O43143  O43143  O43143  O43143  O43143  O43143  O43143  O43143  O43143  O43143  O43143  O43143  O43143  O43143  O43143  O43143  O43143  O43143  O43143  O43143  O43143  O43143  O43143  O43252  O43252  O43252  O43252  O43252  O43252  O43252  O43252  O43252  O43252  O43252  O43252  O43252  O43252  O43252  O43252  O43252  O43252  O43252  O43252  O43252  O43290  O43290  O43290  O43290  O43290  O43290  O43290  O43290  O43290  O43290  O43290  O43290  O43615  O43615  O43615  O43615  O43615  O43615  O43615  O43615  O43615  O43660  O43660  O43660  O43660  O43660  O43660  O43660  O43660  O43660  O43660  O43660  O43660  O43660  O43684  O43684  O43684  O43684  O43684  O43684  O43684  O43684  O43684  O43684  O43684  O43684  O43709  O43709  O43709  O43709  O43709  O43709  O43709  O43709  O43709  O43709  O43719  O43719  O43719  O43719  O43719  O43719  O43719  O43719  O43719  O43719  O43719  O43719  O43719  O43765  O43765  O43765  O43765  O43765  O43765  O43765  O43765  O43765  O43776  O43776  O43776  O43776  O43776  O43776  O43776  O43776  O43776  O43776  O43776  O43776  O43776  O43776  O43776  O43776  O43776  O43776  O43776  O43776  O43776  O43852  O43852  O43852  O43852  O43852  O43852  O43852  O43852  O43852  O43852  O43852  O43852  O60256  O60256  O60256  O60256  O60256  O60256  O60256  O60506  O60506  O60506  O60506  O60506  O60506  O60506  O60506  O60506  O60506  O60506  O60506  O60506  O60506  O60506  O60506  O60506  O60506  O60506  O60506  O60506  O60506  O60506  O60506  O60506  O60506  O60506  O60506  O60506  O60506  O60506  O60506  O60506  O60506  O60506  O60641  O60641  O60641  O60641  O60641  O60641  O60641  O60641  O60884  O60884  O60884  O60884  O60884  O60884  O60884  O60884  O60884  O60884  O60884  O75083  O75083  O75083  O75083  O75083  O75083  O75083  O75083  O75083  O75083  O75083  O75083  O75083  O75083  O75083  O75083  O75083  O75083  O75083  O75083  O75083  O75083  O75083  O75083  O75083  O75347  O75347  O75347  O75390  O75390  O75390  O75390  O75390  O75390  O75390  O75390  O75390  O75390  O75390  O75390  O75390  O75390  O75390  O75390  O75396  O75396  O75396  O75396  O75396  O75396  O75396  O75396  O75792  O75792  O75792  O75792  O75792  O75792  O75792  O75792  O75792  O75832  O75832  O75832  O75832  O75832  O75947  O75947  O75947  O75947  O94903  O94903  O95466  O95466  O95466  O95466  O95466  O95466  O95466  O95466  O95466  O95466  O95466  O95466  O95466  O95466  O95466  O95466  O95466  O95466  P01654  P01654  P01864  P01864  P01864  P01864  P01864  P01864  P01864  P05064  P05064  P05064  P05064  P05064  P05064  P05064  P05064  P05064  P05064  P05201  P05201  P05201  P05201  P05201  P05201  P05201  P05201  P05201  P05201  P05201  P05202  P05202  P05202  P05202  P05202  P05202  P05202  P05202  P05202  P07724  P07724  P07724  P07724  P07724  P07724  P07724  P07724  P07724  P07724  P07724  P07724  P07724  P07724  P07724  P07724  P07724  P07724  P07724  P08551  P08551  P08551  P08551  P08551  P08551  P08551  P08551  P08551  P08551  P08551  P08551  P08551  P08551  P08551  P08553  P08553  P08553  P08553  P08553  P08553  P08553  P08553  P08553  P08553  P08553  P08553  P08553  P08553  P08553  P10649  P10649  P10649  P10649  P10649  P10853  P10853  P14094  P14094  P14094  P14094  P14094  P14094  P14094  P14094  P14094  P14094  P14094  P14094  P14094  P14094  P14094  P14094  P14094  P14094  P14094  P14206  P14206  P14206  P14206  P14206  P14206  P15105  P15105  P15105  P15105  P15105  P15105  P15105  P15105  P15105  P15105  P15105  P15105  P16858  P16858  P16858  P16858  P16858  P16858  P16858  P16858  P17183  P17183  P17183  P17183  P17183  P17183  P17183  P17183  P17183  P17183  P18531  P18531  P18531  P18531  P18531  P18531  P18531  P18872  P18872  P18872  P18872  P18872  P18872  P18872  P18872  P18872  P18872  P18872  P18872  P19246  P19246  P19246  P19246  P19246  P19246  P19246  P19783  P19783  P19783  P19783  P19783  P19783  P21803  P21803  P21803  P21803  P21803  P21803  P21803  P21803  P21803  P21803  P21803  P21803  P21803  P21803  P21803  P21803  P21803  P21803  P21803  P21803  P21803  P21803  P21803  P21803  P21803  P21803  P21803  P21803  P21803  P21803  P21803  P21803  P30275  P30275  P30275  P30275  P30275  P30275  P30275  P30275  P30275  P35441  P35441  P35441  P35441  P35441  P35441  P35441  P35441  P35441  P35441  P35441  P35441  P35441  P35441  P35441  P35441  P35441  P35441  P35441  P35441  P35441  P35441  P35441  P35441  P35441  P35441  P35441  P35441  P35441  P39053  P39053  P39053  P39053  P39053  P39053  P39053  P39053  P39053  P39053  P39053  P39053  P39053  P39053  P39053  P39053  P40142  P40142  P40142  P40142  P40142  P40142  P40142  P40142  P40142  P40142  P40142  P40142  P40142  P40142  P40142  P40142  P41588  P41588  P41588  P41588  P41588  P41588  P41588  P41588  P41588  P41588  P41588  P41588  P41588  P41588  P41588  P41588  P50516  P50516  P50516  P50516  P50516  P50516  P50516  P50516  P50516  P50516  P50516  P50516  P50516  P50516  P50516  P50516  P50516  P50516  P50516  P50516  P50516  P50516  P50516  P50516  P56387  P56387  P56387  P56387  P58281  P58281  P58281  P58281  P58281  P58281  P58281  P58281  P58281  P58281  P58281  P58281  P58281  P58281  P58281  P58281  P58281  P58281  P58281  P58281  P58281  P58281  P58281  P60202  P60202  P60202  P60202  P60202  P60202  P60202  P60202  P60202  P60202  P60202  P60202  P60202  P60710  P60710  P60710  P60710  P60710  P62259  P62259  P62259  P62259  P62259  P62259  P62259  P62814  P62814  P62814  P62814  P62814  P62814  P62814  P62814  P62814  P62814  P62814  P62814  P62814  P62814  P62814  P62814  P62814  P63168  P63168  P63168  P63168  P63328  P63328  P63328  P63328  P63328  P63328  P63328  P63328  P63328  P63328  P63328  P63328  P63328  P63328  P63328  P63328  P63328  P67778  P67778  P67778  P67778  P70338  P70338  P70338  P70338  P70338  P70338  P70338  P70338  P70338  P70338  P70338  P85094  P97427  P97427  P97427  P97427  P97427  P97427  P97427  P97427  P97427  P97427  P97427  P97427  P97427  P97427  P97427  P97427  P98063  P98063  P98063  P98063  P98063  P98063  P98063  P98063  P98063  P98063  P98063  P98063  P98063  P98063  P98063  P98063  P98063  P98063  P98063  P98063  P98063  P98063  P98063  P98063  P98063  P98063  P98063  P98063  P98063  P98063  P98063  P98063  P98063  P98063  P98063  P98063  P98063  P98063  P98063  P98063  P98063  Q04447  Q04447  Q04447  Q04447  Q04447  Q04447  Q04447  Q08122  Q08122  Q08122  Q08122  Q08122  Q08122  Q08122  Q08122  Q08122  Q08122  Q08122  Q08122  Q08122  Q08122  Q08122  Q08122  Q08122  Q08122  Q08122  Q08122  Q08122  Q08122  Q11011  Q11011  Q11011  Q11011  Q11011  Q11011  Q11011  Q11011  Q11011  Q11011  Q11011  Q11011  Q11011  Q11011  Q11011  Q11011  Q11011  Q11011  Q11011  Q11011  Q11011  Q11011  Q11011  Q11011  Q11011  Q11011  Q11011  Q11011  Q3ULF4  Q3ULF4  Q3ULF4  Q3ULF4  Q3ULF4  Q3ULF4  Q3ULF4  Q3ULF4  Q3ULF4  Q3ULF4  Q3ULF4  Q3ULF4  Q3ULF4  Q3ULF4  Q3ULF4  Q3UYC0  Q3UYC0  Q3UYC0  Q3UYC0  Q3UYC0  Q3UYC0  Q3UYC0  Q3UYC0  Q3UYC0  Q3UYC0  Q3UYC0  Q3UYC0  Q3UYC0  Q60865  Q60865  Q60865  Q60865  Q60865  Q60865  Q60865  Q60865  Q60865  Q60865  Q60865  Q60865  Q60865  Q60865  Q60865  Q60865  Q60865  Q60865  Q60865  Q60865  Q60865  Q60865  Q60865  Q61644  Q61644  Q61644  Q61644  Q61644  Q61644  Q61644  Q61644  Q61644  Q61644  Q61644  Q61644  Q61644  Q61644  Q61644  Q61656  Q61656  Q61656  Q61656  Q61656  Q61656  Q61656  Q61656  Q61656  Q61656  Q61656  Q61656  Q61656  Q61656  Q61656  Q61656  Q61656  Q61656  Q61656  Q61656  Q61656  Q61656  Q61656  Q61656  Q6I6G8  Q6I6G8  Q6I6G8  Q6I6G8  Q6I6G8  Q6I6G8  Q6I6G8  Q6I6G8  Q6I6G8  Q6I6G8  Q6I6G8  Q6I6G8  Q6I6G8  Q6I6G8  Q6I6G8  Q6I6G8  Q6I6G8  Q6I6G8  Q6I6G8  Q6I6G8  Q6I6G8  Q6I6G8  Q6I6G8  Q6I6G8  Q6I6G8  Q6I6G8  Q6I6G8  Q6I6G8  Q6I6G8  Q6I6G8  Q6I6G8  Q6I6G8  Q6I6G8  Q6I6G8  Q6I6G8  Q6I6G8  Q6I6G8  Q6I6G8  Q6I6G8  Q6I6G8  Q6I6G8  Q6PIC6  Q6PIC6  Q6PIC6  Q6PIC6  Q6PIC6  Q6PIC6  Q6PIC6  Q6PIC6  Q6PIC6  Q6PIC6  Q6PIC6  Q6PIC6  Q6PIC6  Q6PIC6  Q6PIC6  Q6PIC6  Q6PIC6  Q6PIC6  Q6PIC6  Q6PIC6  Q6PIC6  Q6ZQ93  Q6ZQ93  Q6ZQ93  Q6ZQ93  Q6ZQ93  Q6ZQ93  Q6ZQ93  Q6ZQ93  Q6ZQ93  Q6ZQ93  Q6ZQ93  Q6ZQ93  Q6ZQ93  Q6ZQ93  Q6ZQ93  Q6ZQ93  Q6ZQ93  Q6ZQ93  Q6ZQ93  Q6ZQ93  Q6ZQ93  Q6ZQ93  Q6ZQ93  Q6ZQ93  Q6ZQ93  Q6ZQ93  Q6ZQ93  Q6ZQ93  Q6ZQ93  Q6ZQ93  Q6ZQ93  Q6ZQ93  Q6ZQ93  Q6ZQ93  Q6ZQ93  Q6ZQ93  Q6ZQ93  Q6ZQ93  Q6ZQ93  Q6ZQ93  Q6ZQ93  Q6ZQ93  Q6ZQ93  Q6ZQ93  Q6ZQ93  Q6ZQ93  Q6ZQ93  Q6ZQ93  Q6ZQ93  Q6ZQ93  Q6ZQ93  Q6ZQ93  Q6ZQ93  Q6ZQ93  Q6ZQ93  Q6ZQ93  Q6ZQ93  Q6ZQ93  Q6ZQ93  Q6ZQ93  Q6ZQ93  Q6ZQ93  Q6ZQ93  Q6ZQ93  Q6ZQ93  Q6ZQ93  Q6ZQ93  Q6ZQ93  Q6ZQ93  Q6ZQ93  Q6ZQ93  Q6ZQ93  Q6ZQ93  Q6ZQ93  Q6ZQ93  Q6ZQ93  Q6ZQ93  Q6ZQ93  Q6ZQ93  Q6ZQ93  Q6ZQ93  Q6ZQ93  Q6ZQ93  Q6ZQ93  Q6ZQ93  Q6ZQ93  Q6ZQ93  Q6ZQ93  Q6ZQ93  Q6ZQ93  Q6ZQ93  Q6ZQ93  Q6ZQ93  Q6ZQ93  Q6ZQ93  Q7TMM9  Q7TMM9  Q7TMM9  Q7TMM9  Q7TMM9  Q7TMM9  Q7TMM9  Q7TMM9  Q7TMM9  Q7TMM9  Q7TMM9  Q7TMM9  Q80VM8  Q80VM8  Q80VM8  Q80VM8  Q80VM8  Q80VM8  Q80YV4  Q80YV4  Q80YV4  Q80YV4  Q80YV4  Q80YV4  Q80YV4  Q80YV4  Q80YV4  Q80YV4  Q80YV4  Q80YV4  Q80YV4  Q80YV4  Q80YV4  Q80YV4  Q80YV4  Q80YV4  Q80YV4  Q80YV4  Q80YV4  Q80YV4  Q8BH59  Q8BH59  Q8BH59  Q8BH59  Q8BH59  Q8BH59  Q8BH59  Q8BH59  Q8BH59  Q8BH59  Q8BH59  Q8BH59  Q8BH59  Q8BH59  Q8BH59  Q8BH59  Q8BH59  Q8BH59  Q8BH59  Q8BH59  Q8BH59  Q8BH59  Q8BH59  Q8BH59  Q8BN78  Q8BN78  Q8BN78  Q8BN78  Q8BN78  Q8BN78  Q8BN78  Q8BN78  Q8BN78  Q8BN78  Q8BN78  Q8BN78  Q8BN78  Q8BN78  Q8BN78  Q8BN78  Q8BN78  Q8BN78  Q8BUE4  Q8BUE4  Q8BUE4  Q8BUE4  Q8BUE4  Q8BUE4  Q8BUE4  Q8BUE4  Q8BUE4  Q8BUE4  Q8BVE3  Q8BVE3  Q8BVE3  Q8BVE3  Q8BVE3  Q8BVE3  Q8BVE3  Q8BVE3  Q8BVE3  Q8BVE3  Q8BVE3  Q8BVE3  Q8BVE3  Q8BVE3  Q8BVE3  Q8BVE3  Q8BVE3  Q8CJG0  Q8CJG0  Q8CJG0  Q8CJG0  Q8CJG0  Q8CJG0  Q8CJG0  Q8CJG0  Q8CJG0  Q8CJG0  Q8CJG0  Q8CJG0  Q8CJG0  Q8CJG0  Q8CJG0  Q8CJG0  Q8CJG0  Q8CJG0  Q8CJG0  Q8CJG0  Q8CJG0  Q8CJG0  Q8CJG0  Q8CJG0  Q8CJG0  Q8CJG0  Q8CJG0  Q8K0T0  Q8K0T0  Q8K0T0  Q8K0T0  Q8K0T0  Q8K0T0  Q8K0T0  Q8K0T0  Q8K0T0  Q8K0T0  Q8K0T0  Q8K0T0  Q8K0T0  Q8K0T0  Q8K0T0  Q8K0T0  Q8K0T0  Q8QZT1  Q8QZT1  Q8QZT1  Q8QZT1  Q8QZT1  Q8QZT1  Q8QZT1  Q8QZT1  Q8QZT1  Q8R5C5  Q8R5C5  Q8R5C5  Q8R5C5  Q8R5C5  Q8R5C5  Q8R5C5  Q8R5C5  Q8R5C5  Q8R5C5  Q8R5C5  Q8R5C5  Q91VD9  Q91VD9  Q91VD9  Q91VD9  Q91VD9  Q91VD9  Q91VD9  Q91VD9  Q91VD9  Q91VD9  Q91VD9  Q91VD9  Q91VD9  Q91VD9  Q91VD9  Q91VD9  Q91YM2  Q91YM2  Q91YM2  Q91YM2  Q91YM2  Q91YM2  Q91YM2  Q91YM2  Q91YM2  Q91YM2  Q91YM2  Q91YM2  Q91YM2  Q91YM2  Q91YM2  Q91YM2  Q91YM2  Q91YM2  Q91YM2  Q91YM2  Q91YM2  Q91YM2  Q91YM2  Q91YM2  Q91YM2  Q91YM2  Q91YM2  Q91YM2  Q91YM2  Q91YM2  Q91YM2  Q91YM2  Q91YM2  Q91YM2  Q91YM2  Q91YM2  Q91YM2  Q91YM2  Q91YM2  Q91YM2  Q91YM2  Q91YM2  Q920Q4  Q920Q4  Q920Q4  Q920Q4  Q920Q4  Q920Q4  Q920Q4  Q920Q4  Q920Q4  Q920Q4  Q920Q4  Q920Q4  Q920Q4  Q920Q4  Q920Q4  Q920Q4  Q920Q4  Q920Q4  Q920Q4  Q920Q4  Q920Q4  Q920Q4  Q920Q4  Q920Q4  Q920Q4  Q920Q4  Q920Q4  Q920Q4  Q920Q4  Q99JX7  Q99JX7  Q99JX7  Q99JX7  Q99JX7  Q99JX7  Q99JX7  Q99JX7  Q99JX7  Q99JX7  Q99JX7  Q99JX7  Q99JX7  Q99JX7  Q99JX7  Q99JX7  Q99JX7  Q99JX7  Q99LC5  Q99LC5  Q99LC5  Q99LC5  Q9CQV8  Q9CQV8  Q9CQV8  Q9CQV8  Q9CQV8  Q9CQV8  Q9CQV8  Q9CQV8  Q9CZC8  Q9CZC8  Q9CZC8  Q9CZC8  Q9CZC8  Q9CZC8  Q9CZC8  Q9CZC8  Q9CZC8  Q9CZC8  Q9D0K2  Q9D0K2  Q9D0K2  Q9D0K2  Q9D0K2  Q9D0K2  Q9D0K2  Q9D0K2  Q9D0K2  Q9D6J6  Q9D6J6  Q9D6J6  Q9D6J6  Q9D6J6  Q9D6J6  Q9D6J6  Q9D855  Q9D855  Q9D855  Q9D855  Q9D855  Q9D974  Q9DB20  Q9DB20  Q9DB20  Q9DB20  Q9DBG3  Q9DBG3  Q9DBG3  Q9DBG3  Q9DBG3  Q9DBG3  Q9DBG3  Q9DBG3  Q9DBG3  Q9DBG3  Q9DBG3  Q9DBG3  Q9DBG3  Q9DBG3  Q9DBG3  Q9DBG3  Q9DBG3  Q9DBG3  Q9DBG3  Q9DBG3  Q9DBG3  Q9DBG3  Q9DBG3  Q9DBG3  Q9DBG3  Q9DBG3  Q9DBG3  Q9DBG3  Q9JIS5  Q9JIS5  Q9JIS5  Q9JIS5  Q9JIS5  Q9JIS5  Q9JIS5  Q9JIS5  Q9JIS5  Q9JIS5  Q9JIS5  Q9JIS5  Q9JIS5  Q9JIS5  Q9JIS5  Q9JIS5  Q9JIS5  Q9JIS5  Q9JIS5  Q9JIS5  Q9JIS5  Q9JIS5  Q9JIS5  Q9JIS5  Q9JIS5  Q9NRM2  Q9NRM2  Q9NRM2  Q9NRM2  Q9NRM2  Q9NRM2  Q9NRM2  Q9NRM2  Q9NRM2  Q9NRM2  Q9NRM2  Q9NRM2  Q9NRM2  Q9QUH6  Q9QUH6  Q9QUH6  Q9QUH6  Q9QUH6  Q9QUH6  Q9QUH6  Q9QUH6  Q9QUH6  Q9QUH6  Q9QUH6  Q9QUH6  Q9QUH6  Q9QUH6  Q9QUH6  Q9QUH6  Q9QUH6  Q9QUH6  Q9QUH6  Q9QUH6  Q9QUH6  Q9QUH6  Q9QUH6  Q9QUH6  Q9QUH6  Q9QUH6  Q9QUH6  Q9QUH6  Q9QUH6  Q9QUH6  Q9QUH6  Q9QWH1  Q9QWH1  Q9QWH1  Q9QWH1  Q9QWH1  Q9QWH1  Q9QWH1  Q9QWH1  Q9QWH1  Q9QWH1  Q9QWH1  Q9QYG0  Q9QYG0  Q9QYG0  Q9QYG0  Q9QYG0  Q9QYG0  Q9QYG0  Q9QYG0  Q9QYG0  Q9QYG0  Q9QYG0  Q9QYG0  Q9QYG0  Q9Z188  Q9Z188  Q9Z188  Q9Z188  Q9Z188  Q9Z188  Q9Z188  Q9Z188  Q9Z188  Q9Z188  Q9Z188  Q9Z188  Q9Z188  Q9Z188  Q9Z188  Q9Z188  Q9Z188  Q9Z188  Q9Z188  Q9Z188  Q9Z188  B2RSH2  B2RSH2  B2RSH2  B2RSH2  B2RSH2  B2RSH2  B2RSH2  B2RSH2  B2RSH2  B2RSH2  B2RSH2  B2RSH2  O08530  O08530  O08530  O08530  O08530  O08530  O08530  O08530  O08530  O08530  O08530  O08530  O08530  O08530  O08530  O08553  O08553  O08553  O08553  O08553  O08553  O08553  O08553  O08553  O08553  O08553  O08553  O08585  O08585  O08585  O08585  O08585  O35633  O35633  O35633  O35633  O35633  O35633  O35633  O35633  O35633  O35633  O35633  O35633  O35633  O35633  O35633  O35643  O35643  O35643  O35643  O35643  O35643  O35643  O35643  O35643  O35643  O35643  O35643  O35643  O35643  O35643  O35643  O35643  O35643  O35643  O35643  O35643  O35643  O35643  O35643  O35643  O35643  O35652  O35652  O35652  O35652  O35652  O35652  O35652  O35652  O35652  O35678  O35678  O35678  O35678  O35678  O35678  O35678  O35678  O35678  O88990  O88990  O88990  O88990  O88990  O88990  O88990  O88990  O88990  O88990  O88990  O88990  O88990  O88990  O88990  O88990  O88990  O88990  O88990  O88990  P60301  P60770  P00369  P00369  P00369  P00369  P00369  P00369  P00369  P00369  P00369  P00369  P00369  P00369  P00369  P00369  P00187  P00187  P00187  P00187  P00489  P00489  P00489  P00489  P00489  P00489  P00489  P00489  P00489  P00883  P00883  P00883  P00883  P00883  P00883  P01870  P01870  P01870  P01870  P01870  P01870  P01870  P01870  P46406  P46406  P46406  P46406  P46406  P46406  P46406  P01218  P01218  P01218  P01218  P01240  P01240  P05979  P05979  P05979  P05979  P05979  P05979  P05979  P05979  P05979  P05979  P05979  P05979  P05979  P05979  P05979  P05979  P05979  P05979  P05979  P05979  P05979  P05979  P05979  P05979  P16038  P16038  P16038  P16038  P16038  P16038  Q51918  Q51918  Q51918  Q51918  Q51918  Q51918  Q51918  Q51918  Q51918  Q51918  Q51918  Q51918  Q51918  Q51918  Q51918  Q51918  Q51918  Q51918  Q51918  Q51918  Q51918  Q51918  Q51918  Q51918  Q51918  Q51918  Q51918  Q51918  Q51918  Q51918  P02244  P02244  P00183  P00183  P00183  P00183  P00564  P00564  P00564  P00564  P00564  P00564  P00564  P00773  P00773  P00773  P00773  P00773  P00773  P00773  P00773  P00773  P00773  P00773  P00773  P02564  P02564  P02564  P02564  P02564  P02564  P02564  P02564  P02564  P02564  P02564  P02564  P02564  P02564  P02564  P02564  P02564  P02564  P02564  P02564  P02564  P02564  P02564  P02564  P02564  P02564  P02564  P02564  P02564  P02564  P02564  P02564  P02564  P02564  P02564  P02564  P04177  P04177  P04177  P04177  P04177  P04177  P04177  P04177  P04177  P04177  P04177  P04177  P04177  P04177  P04692  P04692  P04692  P04692  P04692  P04764  P04764  P04764  P04764  P04764  P04764  P04764  P04764  P04764  P04764  P07825  P07825  P07825  P07825  P07825  P07825  P07825  P07825  P07825  P07825  P07825  P07825  P07825  P07825  P07825  P08011  P08011  P08011  P08011  P08011  P10066  P10066  P10066  P10066  P10066  P10066  P10066  P10066  P10066  P10066  P10066  P10066  P10066  P10066  P18163  P18163  P18163  P18163  P18163  P18163  P18163  P18163  P18163  P18163  P18163  P18163  P18163  P18163  P18163  P18163  P18163  P18163  P18163  P18163  P19357  P19357  P19357  P19357  P19357  P19357  P19357  P19357  P19357  P19357  P19357  P19357  P19357  P21807  P21807  P21807  P21807  P21807  P21807  P21807  P32198  P32198  P32198  P32198  P32198  P32198  P32198  P32198  P32198  P32198  P32198  P32198  P32198  P32198  P32198  P32198  P32198  P32198  P32198  P32198  P32198  P32198  P32198  P32198  P32198  P32198  P35570  P35570  P35570  P35570  P35570  P35570  P35570  P35570  P35570  P35570  P35570  P35570  P35570  P35570  P35570  P35570  P35570  P35570  P35570  P35570  P35570  P35570  P35570  P35570  P35570  P35570  P35570  P35570  P35570  P48548  P48548  P48548  P48548  P48548  P48548  P48548  P48548  P48548  P48548  P52504  P52504  P56574  P56574  P56574  P56574  P56574  P56574  P56574  P56574  P56574  P56574  P56574  P56574  P56574  P70623  P98158  P98158  P98158  P98158  P98158  P98158  P98158  P98158  P98158  P98158  P98158  P98158  P98158  P98158  P98158  P98158  P98158  P98158  P98158  P98158  P98158  P98158  P98158  P98158  P98158  P98158  P98158  P98158  P98158  P98158  P98158  P98158  P98158  P98158  P98158  P98158  P98158  P98158  P98158  P98158  P98158  P98158  P98158  P98158  P98158  P98158  P98158  P98158  P98158  P98158  P98158  P98158  P98158  P98158  P98158  P98158  P98158  P98158  P98158  P98158  P98158  P98158  P98158  P98158  P98158  P98158  P98158  P98158  P98158  P98158  P98158  P98158  P98158  P98158  P98158  P98158  P98158  P98158  P98158  P98158  P98158  P98158  P98158  P98158  P98158  P98158  P98158  P98158  P98158  P98158  P98158  P98158  P98158  P98158  P98158  P98158  P98158  P98158  P98158  P98158  P98158  P98158  P98158  P98158  P98158  P98158  P98158  P98158  P98158  P98158  P98158  P98158  P98158  P98158  P98158  P98158  P98158  P98158  P98158  P98158  P98158  P98158  P98158  P98158  P98158  P98158  P98158  P98158  P98158  P98158  P98158  P98158  P98158  P98158  P98158  P98158  P98158  P98158  P98158  P98158  P98158  P98158  P98158  P98158  P98158  P98158  P98158  P98158  P98158  Q62673  Q62673  Q62673  Q62673  Q62673  Q62673  Q62673  Q62673  Q62673  Q62673  Q62673  Q62673  Q62673  Q62673  Q62673  Q62673  Q62673  Q62673  Q62673  Q62673  Q62673  Q62673  Q62886  Q62886  Q62886  Q62886  Q62886  Q62886  Q62886  Q62886  Q62886  Q62886  Q62886  Q62886  Q62886  Q62886  Q62886  Q62886  Q62886  Q62886  Q62886  Q63269  Q63269  Q63269  Q63269  Q63269  Q63269  Q63269  Q63269  Q63269  Q63269  Q63269  Q63269  Q63269  Q63269  Q63269  Q63269  Q63269  Q63269  Q63269  Q63269  Q63269  Q63269  Q63269  Q63269  Q63269  Q63269  Q63269  Q63269  Q63269  Q63269  Q63269  Q63269  Q63269  Q63269  Q63269  Q63269  Q63269  Q63269  Q63269  Q63269  Q63269  Q63269  Q63269  Q63269  Q63269  Q63269  Q63269  Q63269  Q63269  Q63269  Q63269  Q63269  Q63269  Q63269  Q63269  Q63269  Q63269  Q63269  Q63269  Q63269  Q63269  Q63269  Q63269  Q63269  Q63269  Q63269  Q63269  Q63269  Q63269  Q63269  Q63269  Q63269  Q63269  Q63269  Q63269  Q63269  Q63269  Q64455  Q64455  Q64455  Q64455  Q64455  Q64455  Q64455  Q64455  Q64455  Q64455  Q64455  Q64455  Q64455  Q64455  Q64455  Q64455  Q64455  Q64455  Q64455  Q64455  Q64455  Q64455  Q64455  Q64455  Q64455  Q64455  Q64455  Q64455  Q64455  Q64455  Q64455  Q64455  Q64455  Q64455  Q64455  Q64455  Q64455  Q64455  Q64455  Q64455  Q64455  Q64455  Q64455  Q64455  Q64455  Q64455  Q64455  Q64455  Q64455  Q64455  Q64455  Q91YE5  Q91YE5  Q91YE5  Q91YE5  Q91YE5  Q91YE5  Q91YE5  Q91YE5  Q91YE5  Q91YE5  Q91YE5  Q91YE5  Q91YE5  Q91YE5  Q91YE5  Q91YE5  Q91YE5  Q91YE5  Q91YE5  Q91YE5  Q91YE5  Q91YE5  Q91YE5  Q91YE5  Q91YE5  Q91YE5  Q923K9  Q923K9  Q923K9  Q923K9  Q923K9  Q923K9  Q923K9  Q923K9  Q923K9  Q923K9  Q923K9  Q923K9  Q923K9  Q923K9  Q923K9  Q923K9  Q923K9  Q923K9  Q923K9  Q923K9  Q923K9  Q923K9  Q923K9  Q923K9  Q923K9  Q923K9  Q923K9  Q923K9  Q923K9  Q923K9  Q9CR40  Q9CR40  Q9CR40  Q9CR40  Q9CR40  Q9CR40  Q9CR40  Q9CR40  Q9CR40  Q9CR40  Q9CR40  Q9CR40  Q9CR40  Q9CR40  Q9CR40  Q9CR40  Q9CR40  Q9CR40  Q9CR40  Q9CR40  Q9CR40  Q9CR40  Q9CR40  Q9CR40  Q9JKL9  Q9JKL9  Q9JKL9  Q9JKL9  Q9JKL9  Q9JKL9  Q9QYP2  Q9QYP2  Q9QYP2  Q9QYP2  Q9QYP2  Q9QYP2  Q9QYP2  Q9QYP2  Q9QYP2  Q9QYP2  Q9QYP2  Q9QYP2  Q9QYP2  Q9QYP2  Q9QYP2  Q9QYP2  Q9QYP2  Q9QYP2  Q9QYP2  Q9QYP2  Q9QYP2  Q9QYP2  Q9QYP2  Q9QYP2  Q9QYP2  Q9QYP2  Q9QYP2  Q9QYP2  Q9QYP2  Q9QYP2  Q9QYP2  Q9QYP2  Q9QYP2  Q9QYP2  Q9QYP2  Q9QYP2  Q9QYP2  Q9QYP2  Q9QYP2  Q9QYP2  Q9QYP2  Q9QYP2  Q9QYP2  Q9QYP2  Q9QYP2  Q9QYP2  Q9QYP2  Q9QYP2  Q9QYP2  Q9QYP2  Q9QYP2  Q9QYP2  Q9QYP2  Q9QYP2  Q9QYP2  Q9QYP2  Q07008  Q07008  Q07008  Q07008  Q07008  Q07008  Q07008  Q07008  Q07008  Q07008  Q07008  Q07008  Q07008  Q07008  Q07008  Q07008  Q07008  Q07008  Q07008  Q07008  Q07008  Q07008  Q07008  Q07008  Q07008  Q07008  Q07008  Q07008  Q07008  Q07008  Q07008  Q07008  Q07008  Q07008  Q07008  Q07008  Q07008  Q07008  Q07008  Q07008  Q07008  Q07008  Q07008  Q07008  Q07008  Q07008  Q07008  Q07008  Q07008  Q07008  Q07008  Q07008  Q07008  Q07008  Q07008  Q07008  Q07008  Q07008  Q07008  Q07008  Q07008  Q9QXQ0  Q9QXQ0  Q9QXQ0  Q9QXQ0  Q9QXQ0  Q9QXQ0  Q9QXQ0  Q9QXQ0  Q9QXQ0  Q9QXQ0  Q9QXQ0  Q9QXQ0  Q9QXQ0  Q9QXQ0  Q9QXQ0  Q9QXQ0  Q9QXQ0  Q9QXQ0  Q9QXQ0  Q9QXQ0  Q9QXQ0  Q9QXQ0  Q9QXQ0  Q9QXQ0  O15229  O15229  O15229  O15229  O15229  O15229  O15229  O15229  O15229  O15229  O15229  O15229  O15229  O15229  O15229  O15229  O54922  O54922  O54922  O54922  O54922  O54922  O54922  O54922  O54922  O54922  O54922  O54922  O54922  O54922  O54922  O54922  O54922  O54922  O54922  O54922  O54922  O54922  O88457  O88457  O88457  O88457  O88457  O88457  O88457  O88457  O88457  O88457  O88457  O88457  O88457  O88457  O88457  O88457  O88457  O88457  O88457  O88457  O88457  O88457  O88457  O88457  O88457  O88457  O88457  O88457  O88457  O88457  O88457  O88457  O88457  O88457  O88457  O88457  O88457  O88457  O88457  O88457  O88943  O88943  O88943  O88943  O88943  O88943  O88943  O88943  O88943  O88943  O88943  O88943  O88943  O88943  O88943  O88943  O88943  O88943  O88943  O88943  O88943  O88943  O88943  O88943  O88943  O88943  O88943  O24819  O24819  O24819  O24819  O24819  O24819  O24819  O24819  O24819  O24819  O24819  O24819  O24819  O24819  O24819  O24819  O24819  O24819  P00924  P00924  P00924  P00924  P00924  P00924  P00924  P04806  P04806  P04806  P04806  P04806  P04806  P04806  P04806  P04806  P04806  P04806  P04806  P32178  P32178  P32178  P32178  P32178  P32178  P32178  P32178  P32178  P35809  P35809  P35809  P35809  P35809  P35809  P35809  P35809  P35809  P35809  P35809  P35809  P35809  P35809  P35809  P35809  P00644  P00644  P00644  P00644  P00644  P00644  P00644  P00644  P00644  P00807  P00807  P00807  P00807  P00807  P00807  P00807  P00807  P00807  P00807  P00807  P00807  P0A0E3  P01006  P00592  P00592  P00592  P00592  P00592  P00592  P02703  P09955  P09955  P09955  P09955  P09955  P09955  P09955  P09955  P09955  P09955  P09955  P09955  P09955  P09955  P09955  P09955  P09955  P09955  P16276  P16276  P16276  P16276  P16276  P16276  P16276  P16276  P16276  P16276  P16276  P16276  P16276  P16276  P16276  P16276  P16276  P16276  P16276  Q5SIY4  Q5SIY4  Q5SIY4  Q5SIY4  P69687  P69687  P69687  P06859  P06859  P06859  P06859  P06859  P06859  P11507  P11507  P11507  P11507  P11507  P11507  P11507  P11507  P11507  P11507  P11507 | 46  6  170  2  38  131  178  219  239  52  116  161  167  199  257  279  306  314  346  430  487  537  565  579  614  628  695  708  753  790  808  829  830  858  861  868  934  944  946  975  998  1018  1042  90  95  120  183  187  193  247  263  297  303  305  307  315  328  332  344  396  425  431  434  445  460  475  476  482  504  2  4  53  135  152  170  193  240  247  289  367  429  439  451  512  539  86  119  122  129  152  158  200  275  279  314  316  318  344  348  350  369  375  387  419  94  228  21  166  35  67  93  104  110  113  115  186  194  218  221  75  129  195  36  58  118  17  91  151  161  236  292  332  360  370  392  397  417  126  129  135  141  151  214  226  245  286  308  311  327  330  385  389  397  410  420  66  74  132  148  162  168  193  209  214  125  157  170  206  212  216  220  260  264  302  331  366  409  418  437  544  581  605  607  630  642  665  23  70  77  102  103  24  45  69  87  91  121  127  144  188  198  208  236  279  329  400  403  424  456  482  483  494  513  549  635  670  728  812  918  933  950  959  1081  1087  1089  1108  51  118  123  36  40  49  149  171  198  220  239  240  255  308  20  70  80  20  33  59  67  78  89  108  116  123  181  200  211  215  219  221  227  256  277  299  303  319  330  336  341  6  38  49  149  150  157  169  34  61  10  101  110  120  151  160  217  244  279  310  322  436  447  458  518  602  604  628  637  126  204  273  282  22  6  99  166  186  198  227  286  165  239  241  298  320  369  443  466  467  10  26  28  77  163  164  167  174  163  172  195  21  56  61  70  116  141  155  158  159  163  164  167  177  213  231  285  322  326  340  363  365  374  383  384  421  425  443  448  463  471  476  477  511  521  525  534  542  574  605  637  643  683  689  700  706  767  823  35  55  107  167  172  173  192  219  22  31  89  128  130  134  163  193  211  219  264  287  300  384  393  412  476  479  488  490  495  504  510  524  533  556  614  29  34  80  157  158  167  195  210  274  308  311  357  32  49  178  226  361  382  422  429  56  70  92  96  160  163  198  127  145  172  247  281  65  67  129  191  241  371  400  408  435  443  451  324  55  69  67  87  116  136  170  221  275  285  290  320  359  410  434  448  453  477  497  583  600  603  25  66  99  111  151  165  173  175  193  219  233  273  283  323  346  378  385  448  489  530  544  552  554  633  691  732  772  793  71  78  104  121  134  197  207  229  235  238  265  304  326  328  350  351  409  26  129  139  147  209  210  227  243  244  246  261  325  353  363  385  446  455  489  490  492  534  535  635  837  852  854  898  917  1027  1053  1081  1180  1187  1194  1228  1235  1261  1262  1266  1282  1348  1392  1428  1432  1447  1466  1480  1482  1483  1493  1543  1545  1561  1602  1620  23  44  46  59  66  101  103  121  135  146  196  205  216  222  235  240  254  256  266  312  313  322  324  347  352  369  452  457  464  466  492  499  501  523  543  544  582  610  690  768  831  846  848  939  950  961  1020  1024  1059  1064  1066  1092  1111  1124  1143  1193  1225  1253  1266  1280  1285  1300  1317  1326  1378  1391  1394  1399  1408  1453  1496  1498  1509  1559  1561  1577  1585  1617  1629  1635  1637  1647  32  64  71  141  32  63  81  179  183  232  256  274  290  319  101  309  427  475  608  1107  1352  1468  1471  1475  1519  1521  1581  1624  1625  1629  124  139  71  147  149  172  182  222  255  266  269  299  315  356  368  375  408  434  446  447  475  52  54  56  129  152  183  247  272  443  452  469  484  486  562  637  640  740  776  829  975  981  992  993  1014  1020  1053  1090  1157  1169  1200  1207  1247  1287  1346  1363  1367  1373  1389  1402  1412  1422  1496  1501  1526  1537  1563  1566  1578  1602  1606  1714  1746  1774  1780  1791  1803  1831  1840  1851  1885  1928  1946  1992  1999  2060  2080  2111  2125  2149  2177  2184  2189  2216  2256  2348  2368  2389  2432  2480  2535  2543  2549  2568  2692  2759  2834  2889  2976  3074  3096  3098  3177  3223  3233  3251  3272  3339  3379  3462  3470  3509  3520  3560  3680  3710  3717  3800  3894  3908  3926  3958  3964  3990  4025  4026  4082  4084  4152  4232  4245  4269  4324  4328  4340  4343  4380  4388  4400  4401  4425  4451  4491  4497  4509  4510  4528  4534  4551  19  33  35  189  190  193  200  103  107  126  163  205  220  234  236  51  81  95  112  132  165  174  187  191  229  251  291  297  83  93  136  191  201  218  227  245  284  310  349  398  426  485  23  79  89  46  292  361  431  499  17  29  271  128  146  173  248  282  12  55  74  79  80  123  131  142  150  203  254  255  261  269  325  347  354  393  394  24  75  109  147  151  188  199  232  235  238  269  275  311  316  317  318  327  333  7  25  140  38  61  139  142  160  284  309  364  381  434  438  465  466  480  492  493  520  528  604  667  689  40  57  105  126  132  133  137  2  67  68  110  251  257  465  491  10  30  62  66  76  95  134  138  185  201  255  297  302  313  340  439  477  481  556  572  609  645  54  71  134  172  182  236  274  306  351  421  605  835  1004  1294  1490  1515  1519  1548  1556  1557  1565  1586  1623  1667  1668  1672  130  185  38  53  61  117  150  291  319  358  383  400  4  8  64  80  104  109  112  119  154  180  199  38  43  45  95  111  116  210  233  236  258  16  71  109  144  155  162  125  134  150  19  24  53  85  98  100  122  142  178  35  49  67  106  118  121  123  166  191  258  262  263  279  26  90  223  227  385  15  107  288  294  431  443  525  545  611  49  70  107  112  118  139  147  197  202  249  308  322  343  424  428  437  482  484  503  507  84  240  307  348  371  402  429  687  723  816  834  852  901  920  51  75  92  145  175  315  331  415  431  451  454  511  571  606  694  114  133  211  239  249  250  125  134  149  74  225  264  316  333  395  397  416  421  433  444  631  82  124  126  162  79  175  192  232  265  367  373  397  411  457  579  603  634  639  730  745  760  787  28  48  118  124  222  276  299  300  374  417  430  447  462  502  566  585  83  161  175  370  390  444  466  94  200  258  271  280  355  359  372  401  408  412  429  480  527  539  563  567  575  652  667  678  727  735  143  146  198  36  90  111  126  145  233  249  298  332  44  49  85  116  128  137  146  191  201  205  228  270  291  354  478  483  499  565  82  133  169  223  248  250  76  83  94  102  107  123  290  302  384  415  442  474  534  537  546  570  571  623  663  690  728  4  26  119  133  218  402  433  462  463  473  495  83  92  160  24  27  40  51  116  126  137  139  147  183  189  197  207  210  212  240  256  258  262  308  324  326  8  33  72  103  125  132  133  135  150  216  46  138  212  238  240  260  268  360  377  484  488  62  226  309  317  325  401  148  260  287  319  346  403  442  446  470  506  538  542  543  550  635  643  731  735  775  791  838  846  933  937  951  1041  1047  1126  1134  1208  1221  1229  1235  1261  1317  1321  1324  1335  1414  1417  1422  1428  1511  1519  1525  1604  1608  1616  1622  1632  1712  1720  1761  1763  1832  1846  1859  1862  1871  1924  1932  1938  2077  2106  2114  2198  2216  2297  2305  2379  2388  2400  2435  2483  2487  2495  2501  2505  2571  2614  2618  2626  2642  236  269  274  310  344  348  437  537  578  583  638  662  868  14  119  122  231  316  345  158  177  187  251  293  320  381  470  488  490  527  597  602  624  642  698  82  85  89  117  56  68  98  102  107  111  123  191  194  197  202  50  58  120  132  133  143  19  32  45  91  95  99  159  174  188  193  241  251  312  357  459  462  507  545  571  589  594  651  653  705  708  243  246  271  287  291  299  311  321  343  380  401  476  489  495  42  195  248  251  254  289  49  116  128  134  146  191  201  205  228  270  291  556  113  114  127  193  228  267  275  361  53  55  61  103  153  169  175  184  185  188  232  284  333  351  353  364  392  487  540  555  575  608  633  670  42  47  53  60  81  119  130  145  148  204  210  222  250  280  329  333  334  22  57  75  109  128  150  172  182  271  299  306  308  338  47  110  113  144  176  60  100  150  164  185  198  214  229  238  330  379  393  487  514  95  122  124  146  168  170  172  177  71  75  127  157  159  165  278  134  135  217  251  254  467  475  597  735  900  1057  18  182  238  29  81  120  125  158  169  176  181  95  115  182  196  222  264  265  269  278  356  364  402  416  445  454  467  479  27  41  48  54  58  75  95  134  152  236  248  261  269  275  296  303  310  316  323  376  383  397  404  431  444  451  468  476  90  96  122  6  33  49  73  88  102  126  137  182  192  221  297  333  345  393  403  492  495  539  562  580  585  600  701  166  241  272  276  286  296  308  343  348  386  450  14  30  89  105  148  181  184  208  252  400  435  445  446  454  597  624  626  636  660  671  709  723  7  25  30  79  129  134  16  46  150  151  179  233  267  270  347  373  389  451  483  489  490  491  575  621  631  709  767  812  830  868  908  930  1025  1045  1055  1080  1113  1123  1134  18  26  34  42  161  190  213  279  290  311  163  233  301  308  381  484  518  35  86  101  144  193  194  201  216  261  269  290  354  375  378  403  434  450  490  536  545  546  562  566  574  585  594  658  696  732  761  824  6  14  17  25  33  38  41  50  55  58  66  75  81  91  97  100  113  122  130  136  143  149  155  161  177  194  208  232  239  304  325  397  479  484  526  36  41  111  147  174  249  420  429  432  11  52  122  161  211  264  331  7  163  186  310  330  360  394  461  492  522  544  565  599  623  661  673  703  725  757  818  834  921  939  45  80  134  196  267  285  297  8  22  70  103  37  58  68  129  221  223  224  229  31  134  190  206  214  220  225  234  241  243  246  255  260  266  272  281  285  288  310  313  320  327  335  357  364  391  46  118  128  161  181  230  243  331  568  253  287  44  56  71  94  116  127  148  152  167  173  198  244  267  273  283  289  295  312  327  333  369  397  434  462  535  540  569  608  618  626  657  660  680  714  717  734  772  781  803  845  869  888  949  1012  1013  1021  1044  1082  1173  1212  1252  39  59  160  165  172  177  200  207  270  277  317  320  334  393  441  477  480  565  570  583  586  607  623  633  705  714  751  849  852  892  904  958  959  965  1016  1052  1070  1082  1119  1215  1220  1251  1328  1341  1359  1365  1382  128  158  171  213  214  219  243  250  289  326  413  453  454  474  508  520  526  572  597  773  779  784  794  802  810  827  42  46  47  104  120  125  176  195  214  244  246  266  360  366  405  416  435  440  442  452  45  83  112  259  499  502  593  611  724  25  40  102  35  84  95  7  32  34  78  77  91  148  558  575  588  594  596  600  635  126  198  232  274  303  437  506  4  18  31  25  93  109  134  137  192  222  258  266  279  295  322  334  415  418  468  487  489  497  535  543  547  572  580  667  5  38  39  53  93  99  117  146  172  192  197  203  212  219  224  226  245  249  286  333  349  404  421  442  5  29  56  65  78  87  97  104  106  125  128  147  160  177  188  199  204  217  222  231  234  241  257  280  290  291  292  297  313  328  350  355  360  361  362  376  392  396  401  409  458  463  470  478  486  489  516  518  521  24  179  439  449  37  78  144  151  183  71  100  213  681  48  107  125  130  146  153  172  38  44  51  64  65  72  78  80  95  99  100  119  136  176  198  210  248  266  293  313  323  328  422  451  461  489  16  31  120  124  131  192  196  199  247  252  288  304  307  317  319  364  517  531  542  579  588  652  659  682  692  704  725  801  825  854  885  993  1006  1051  1073  1084  1093  110  134  138  143  173  203  244  495  517  755  796  805  32  80  222  232  237  253  272  295  306  339  348  387  525  592  652  665  681  727  760  768  93  48  50  126  170  175  250  267  347  356  391  34  62  147  21  72  100  109  125  140  184  202  231  233  245  256  273  316  375  391  400  87  134  146  172  246  268  281  290  296  316  348  350  411  436  483  491  527  573  31  133  205  256  76  100  39  47  71  135  138  225  230  234  236  280  333  361  369  371  373  380  449  458  69  70  78  87  93  110  114  134  137  144  272  306  23  24  72  148  189  369  21  27  55  109  113  117  149  198  49  82  95  96  99  141  145  7  46  10  36  37  92  95  99  54  103  121  149  60  74  137  99  39  79  80  98  146  155  197  28  60  40  67  89  186  189  7  37  52  118  134  160  53  140  194  228  246  302  80  86  91  92  107  127  130  137  145  152  218  248  265  267  307  56  85  86  141  162  167  177  183  357  418  36  29  128  132  185  277  439  555  642  779  832  839  856  297  299  408  439  456  27  84  92  97  139  175  229  239  14  21  55  13  33  104  55  59  100  130  141  148  158  165  208  217  230  246  271  333  339  128  156  184  279  281  285  294  337  417  430  479  504  510  556  608  634  654  689  713  731  754  790  792  799  803  899  900  908  946  966  1043  1050  1096  1128  1137  1156  1168  1206  1211  1218  1289  1290  1295  1324  1365  1368  1371  1404  1405  1411  1438  1451  1477  1487  1513  1534  1540  1574  1598  1606  1634  1657  1659  1664  1673  257  260  266  293  327  350  379  381  396  421  466  473  613  654  669  730  751  753  765  771  782  795  802  820  824  825  23  37  44  50  92  110  115  144  31  43  58  153  164  172  186  196  249  352  354  10  28  46  63  84  94  97  57  111  113  161  177  178  220  225  245  280  286  293  302  383  411  57  63  69  73  115  116  216  270  282  302  20  36  100  227  257  288  306  349  360  496  99  101  109  116  119  48  51  364  378  34  38  44  116  19  39  72  77  79  82  112  149  153  202  226  237  5  11  25  37  53  79  86  88  96  104  123  150  185  9  21  43  45  68  132  148  149  188  198  218  251  259  274  370  471  534  554  564  568  573  616  643  664  681  727  748  875  902  1000  1005  1038  1155  1167  1173  1180  1185  1189  1194  1199  1210  1218  1234  1241  1256  1260  1270  38  51  52  83  98  121  222  262  318  537  561  609  612  661  698  21  32  98  132  181  284  409  16  30  41  44  60  77  99  123  129  133  137  138  153  154  185  205  206  208  213  218  220  262  268  271  280  299  302  318  321  325  326  366  397  402  405  429  445  453  458  471  483  488  491  494  496  501  507  508  513  515  569  576  580  581  583  584  595  598  604  615  616  660  668  670  682  684  699  731  826  885  30  54  71  81  104  107  118  126  140  146  172  190  193  213  223  255  280  295  300  328  343  346  349  356  363  378  381  412  420  421  440  446  450  453  516  528  534  546  581  708  4  43  73  77  123  214  374  49  134  185  189  194  238  277  306  307  312  400  404  499  517  525  527  566  574  589  616  641  659  740  744  33  38  49  64  77  156  133  165  186  208  369  449  458  755  18  38  48  118  139  173  241  300  313  318  54  57  111  113  159  223  243  278  284  291  302  327  333  355  409  13  14  15  22  23  24  45  48  54  67  72  152  166  172  188  201  204  221  222  226  237  303  314  330  333  336  358  44  46  72  98  113  116  176  179  260  292  407  430  433  459  112  26  44  154  361  433  463  473  539  601  644  695  706  766  895  918  978  1020  1073  1176  1411  1579  1686  1792  1872  1898  2004  2167  2227  2282  2357  2391  2430  2440  2448  2462  9  20  86  144  164  181  190  282  299  370  384  437  505  527  546  576  583  633  713  719  732  742  837  935  958  975  988  1076  1093  1096  1142  1156  1158  1169  1197  1212  1222  1230  1234  1240  1243  1261  1298  1326  1410  1422  1503  1508  1529  1552  1667  1675  1757  1758  1806  1832  1920  1944  71  120  124  141  173  212  242  247  336  425  433  435  464  474  482  497  524  541  567  101  109  146  164  221  286  332  372  406  427  527  553  604  661  775  788  827  832  869  893  970  980  983  1004  1010  1019  1037  1050  1057  1069  1109  1120  1172  1190  1210  1283  1380  1383  1393  1473  1480  1512  1513  1546  1618  1630  1738  1745  1845  1868  1872  1889  1990  2022  2055  2086  2170  2190  2193  2211  2265  2350  2426  2472  2489  2493  2496  2517  2537  2582  2638  2641  2674  2693  2735  2738  2748  2765  2777  2792  2794  2873  2881  2892  2901  2959  2967  3026  3051  3103  3120  3125  3130  3176  3183  3371  3377  3379  3402  3447  3451  3516  3519  3552  3586  3604  3801  3812  3825  3827  3836  3841  3901  3972  4049  4180  4196  4205  4252  4357  4447  4467  4520  4527  4610  4636  46  84  87  89  111  174  276  294  347  418  510  546  567  575  699  720  752  783  805  810  817  15  84  117  168  186  209  229  298  326  375  441  511  516  530  559  600  611  714  973  983  1085  1107  1113  1147  1173  1194  1204  1223  8  34  54  77  110  117  159  160  162  359  364  425  440  449  503  505  527  659  691  706  732  739  750  776  788  791  792  13  44  47  52  57  86  122  152  171  183  186  207  211  213  241  17  63  105  121  151  211  258  296  7  42  45  28  38  80  87  90  94  182  193  207  222  270  341  4  77  92  142  168  189  211  224  255  258  329  357  359  424  473  520  565  577  664  693  723  725  736  783  807  904  926  928  970  989  1014  1041  1095  1119  1166  1167  1215  56  69  80  117  144  166  176  385  394  396  45  69  117  138  168  176  90  93  94  193  207  215  245  271  314  393  410  759  772  773  74  95  118  123  166  4  7  33  34  107  115  117  122  143  189  42  73  83  99  115  141  169  177  181  259  398  406  421  446  481  496  55  100  64  81  95  358  543  578  626  636  656  658  662  693  696  744  34  62  103  146  95  147  218  253  258  315  324  33  114  133  138  223  227  242  248  261  288  296  369  18  45  67  104  105  200  256  283  379  433  493  495  549  7  24  45  88  94  120  140  178  208  219  225  239  254  256  312  318  320  332  351  394  431  447  453  454  471  501  515  520  564  580  592  594  596  613  614  742  756  783  794  800  810  832  850  871  902  909  921  925  928  953  985  1008  1019  1028  1043  1044  1091  1113  1128  1178  1273  1317  1369  1371  1389  1432  1445  1470  1494  1548  1620  1635  1660  1671  1679  1687  1695  1709  1715  1741  1754  1762  1768  1786  1920  1930  1991  2062  2091  2102  2104  2127  2129  2168  2228  2235  2251  2256  2281  2294  2305  2326  2334  24  172  185  210  312  319  399  408  432  41  109  113  178  216  217  304  365  394  398  421  465  476  533  543  639  640  661  682  697  728  746  763  766  796  878  895  908  8  231  294  310  328  336  392  480  525  616  644  656  694  700  703  718  720  730  910  932  961  972  987  1028  6  82  234  252  274  291  304  307  311  313  339  372  398  502  533  564  681  772  784  796  797  878  891  907  928  973  980  1053  1090  1117  200  208  212  228  14  15  69  87  160  184  238  289  356  362  415  440  508  515  532  545  11  31  72  74  85  108  127  224  263  310  317  364  451  61  96  195  198  200  206  258  291  295  298  323  373  395  398  533  562  614  644  109  184  239  351  353  425  457  498  6  113  114  125  136  184  296  300  302  316  344  377  398  451  453  488  518  606  713  728  760  775  807  813  817  829  879  902  903  958  970  1008  1069  1093  1112  1118  1129  31  63  126  128  171  207  209  236  240  285  352  454  460  487  490  498  505  537  606  638  670  683  755  949  999  1074  4  58  60  105  242  268  484  489  505  514  540  541  544  545  582  589  590  611  618  619  625  626  18  22  35  44  25  114  187  226  237  249  261  285  305  318  331  341  348  356  365  377  384  395  402  410  417  425  432  442  449  461  474  486  498  504  516  528  540  548  558  576  588  592  602  614  635  645  648  652  655  665  47  94  131  132  193  216  241  275  291  96  132  139  199  212  280  73  92  94  133  152  164  208  111  232  263  275  302  358  419  423  438  475  92  99  111  124  137  175  183  216  232  241  250  302  304  319  369  390  450  473  502  510  594  629  655  667  688  717  750  757  762  767  771  784  786  789  792  797  808  813  816  819  826  827  836  837  840  842  845  5  40  43  52  15  59  99  245  395  446  456  480  136  140  144  148  155  161  205  216  224  250  278  397  405  419  423  436  450  466  14  52  84  99  176  177  211  312  367  377  383  462  520  521  542  552  553  554  563  564  568  629  642  666  700  831  841  846  909  918  931  938  947  45  98  99  119  128  130  182  221  276  281  293  313  323  40  53  76  124  135  221  293  298  361  389  394  521  541  547  652  721  81  95  101  142  169  188  203  214  322  5  19  126  167  186  221  226  259  34  43  53  57  61  71  73  80  81  88  93  133  150  219  284  383  408  438  459  466  511  620  635  730  733  736  786  839  846  858  892  57  65  67  76  112  143  152  248  325  332  377  378  46  52  54  76  111  156  183  213  214  237  240  264  275  313  369  373  416  468  473  507  531  534  577  600  604  637  666  684  685  687  768  812  835  901  916  939  944  985  1026  1135  1173  83  86  123  137  189  192  218  233  246  249  378  382  385  428  464  473  486  492  618  6  26  166  178  191  246  173  186  214  240  259  307  324  336  385  393  441  489  16  74  90  119  198  200  169  190  244  257  33  42  167  199  213  217  239  19  20  34  40  56  125  133  175  176  327  42  95  155  201  172  215  340  362  387  401  442  446  173  210  305  310  312  337  350  409  412  422  469  488  54  68  80  84  85  94  99  104  107  114  118  228  293  328  344  348  412  476  526  657  704  790  795  848  873  880  881  96  104  114  215  236  258  630  14  16  63  65  73  88  221  28  30  127  191  202  248  250  252  261  280  300  53  15  58  86  108  144  148  166  172  33  49  104  133  170  174  233  289  317  345  359  373  401  429  457  485  513  555  597  15  22  29  37  57  112  133  150  164  221  251  308  331  354  371  374  387  416  461  526  550  6  24  57  76  83  97  98  101  121  145  155  167  178  105  113  122  175  185  207  231  246  293  312  323  373  151  238  278  348  14  18  71  13  49  50  74  115  132  208  258  262  286  303  306  338  414  463  465  475  502  528  587  623  630  643  690  737  836  851  855  919  924  949  975  978  991  1010  1019  1091  1104  1109  1153  1155  1167  1168  1259  1296  1348  1382  1387  1450  52  166  173  216  297  301  514  712  58  105  119  181  217  251  294  348  366  449  495  521  657  661  690  693  710  712  806  810  827  918  957  963  1005  1022  1047  1049  1080  1088  1137  1181  1213  1287  1329  1330  1352  1355  1388  1435  1559  1565  1578  1591  1597  1607  1629  1813  1833  1845  1897  1932  1990  2004  2054  2099  2117  2165  2182  2195  2263  2289  2373  2416  2450  2499  2511  2517  2518  2527  2554  2616  2621  2640  2668  2716  2743  2769  2818  2827  2870  2877  2891  2992  3027  3034  3043  3074  3124  3141  3144  3198  3263  3266  3272  3360  3397  3432  3497  3512  3522  3559  3577  3580  3616  3670  3773  3785  3787  3788  3819  3931  3954  3976  4024  4028  4045  4122  4169  4364  4398  4405  4411  4431  4472  4495  4496  4521  4549  4562  4566  4608  4624  4628  4637  4682  4684  4696  4716  4721  4742  4745  4800  4821  4827  4842  4847  4865  20  101  137  142  144  174  199  227  269  321  339  352  358  386  454  544  557  607  615  686  64  108  102  144  151  197  284  306  207  20  75  111  137  199  226  227  240  247  251  273  302  324  369  370  32  65  71  80  125  116  198  280  283  286  297  304  307  324  334  337  342  389  468  481  502  535  554  560  613  633  55  66  153  201  204  226  235  254  276  285  338  358  367  371  403  412  450  453  460  486  537  555  558  602  615  645  686  697  764  1149  1267  126  167  252  281  314  353  354  358  361  365  371  374  377  379  382  384  387  389  393  396  403  417  420  13  127  128  142  177  219  235  254  303  331  389  447  485  490  499  545  581  631  645  651  685  686  697  710  736  746  765  766  792  795  30  75  84  135  174  192  232  238  287  330  365  388  396  444  497  518  565  574  575  617  618  102  249  313  316  337  569  673  685  691  704  712  783  36  51  104  242  265  344  348  421  435  52  82  92  100  148  197  260  277  284  292  385  445  489  42  53  65  104  141  180  194  219  235  275  297  299  18  26  48  56  70  77  143  164  191  201  22  50  71  75  136  173  188  209  224  236  353  634  650  9  114  127  135  141  158  162  181  182  7  123  155  168  182  265  266  289  303  321  335  393  413  448  499  500  502  508  516  531  539  47  106  129  134  136  149  185  187  213  218  263  294  94  110  113  172  180  291  360  47  102  133  144  153  205  223  276  293  428  431  432  433  434  454  456  460  461  463  466  467  469  470  472  474  477  481  485  486  488  523  570  604  606  614  15  44  96  118  127  130  138  211  10  28  35  66  69  128  271  298  320  330  391  3  34  54  66  72  96  98  160  214  216  238  300  303  336  338  348  390  420  446  472  497  522  529  557  606  26  48  75  69  80  194  199  217  221  246  258  286  331  357  381  411  412  419  420  63  87  110  126  180  186  193  209  47  51  111  127  149  206  210  271  287  15  112  160  161  199  56  57  150  152  58  252  59  83  153  163  240  312  348  358  377  739  760  847  857  882  891  973  997  1057  32  53  9  32  62  184  237  279  295  59  85  138  204  214  223  302  328  343  364  41  124  135  159  161  226  243  264  373  381  400  75  179  181  182  246  277  284  401  420  35  54  108  162  172  174  180  181  185  287  343  356  358  377  394  419  435  476  521  6  9  14  33  40  43  57  240  373  390  413  425  433  443  444  3  14  52  63  92  113  119  259  294  318  357  382  399  422  804  116  155  167  197  203  84  122  39  43  68  98  101  112  167  169  199  230  236  237  243  244  246  247  255  280  286  37  39  47  156  202  204  17  30  78  104  137  161  162  171  180  185  283  288  40  43  47  138  274  312  318  328  25  57  131  189  200  236  257  280  287  407  8  45  69  72  78  112  113  74  95  147  155  156  168  231  291  297  303  320  354  26  109  144  227  319  383  400  33  38  82  124  126  162  42  50  91  105  155  207  229  237  244  281  308  328  340  375  381  466  561  566  575  586  588  608  616  657  680  704  733  769  779  783  805  812  48  54  73  134  154  159  207  208  393  168  258  325  565  579  615  665  669  674  682  711  729  758  763  765  779  815  817  876  1000  1022  1029  1036  1047  1058  1108  1139  1143  1165  80  231  276  314  390  449  485  541  553  563  597  600  626  669  706  732  4  58  83  137  141  147  150  173  202  309  321  363  447  481  563  564  10  82  110  122  129  133  183  210  217  225  243  322  340  354  374  402  20  40  67  117  153  178  185  266  274  328  336  342  374  377  386  454  457  464  465  525  528  535  548  579  35  62  64  76  41  80  82  102  113  132  146  206  229  268  278  282  425  498  518  582  637  746  777  841  861  862  863  50  55  58  60  70  73  82  92  104  157  175  177  263  133  143  166  306  337  20  121  122  128  152  181  182  39  47  165  222  287  303  326  328  335  371  378  389  423  426  451  468  474  32  65  75  77  113  119  124  132  140  159  170  175  258  260  262  288  291  311  315  324  341  28  107  114  259  15  25  116  123  140  169  201  212  223  341  345  33  32  36  75  135  145  167  170  174  182  251  336  395  431  479  499  504  35  37  50  180  185  193  252  269  277  295  322  343  347  375  382  384  436  456  460  495  497  514  517  575  583  632  651  683  730  772  807  847  879  889  905  924  926  933  943  953  978  68  82  100  125  173  174  279  2  47  61  64  65  69  220  238  367  389  410  448  502  534  580  667  694  710  731  755  768  772  64  108  123  161  165  174  225  245  254  270  293  296  301  327  384  394  405  439  452  508  533  590  594  650  673  749  843  909  43  100  138  195  212  245  256  270  327  406  547  615  659  711  740  41  80  145  281  316  349  359  373  404  418  423  466  502  76  97  188  205  228  243  281  292  368  440  447  539  543  571  602  603  623  634  637  649  660  663  668  5  22  53  74  118  157  206  220  261  265  347  353  391  393  433  4  59  76  148  190  202  244  297  416  418  425  442  497  514  518  535  540  567  571  577  593  595  606  612  21  66  68  95  130  142  143  249  300  314  418  477  808  831  869  947  973  1079  1087  1155  1166  1195  1207  1215  1242  1253  1264  1283  1284  1289  1295  1328  1339  1354  1411  1436  1473  1477  1514  1555  1562  105  121  138  139  305  478  493  548  549  768  814  844  859  892  898  960  971  984  991  1012  1013  37  53  172  175  212  231  271  275  321  424  462  864  975  1000  1001  1015  1062  1086  1124  1157  1161  1162  1221  1266  1282  1329  1476  1499  1503  1580  1640  1642  1665  1715  1758  1771  1882  1896  1914  1918  1941  1950  1964  1983  1992  2001  2066  2079  2097  2128  2155  2183  2185  2202  2203  2215  2221  2250  2269  2273  2316  2429  2479  2484  2510  2546  2613  2663  2674  2714  2723  2765  2778  2792  2798  2812  2843  2870  2891  2892  2929  2969  3019  3048  3078  3117  3129  3171  3178  3182  3212  3253  3271  3306  3452  50  59  159  183  200  208  281  310  340  398  422  425  33  53  163  218  266  270  50  51  79  100  128  169  189  258  310  338  358  377  383  431  447  484  498  523  603  662  781  815  23  33  43  47  74  147  218  236  260  272  278  328  345  369  382  389  481  498  502  557  598  606  639  656  14  56  84  86  432  440  482  501  520  524  534  548  560  582  595  597  615  671  69  132  165  220  223  277  281  296  321  345  36  92  114  125  163  164  197  214  241  263  305  326  355  388  421  450  466  22  56  58  102  175  226  280  312  323  339  394  421  495  513  530  626  655  668  699  750  766  785  791  805  806  816  858  105  114  122  194  196  263  368  431  451  598  630  645  664  679  686  718  743  30  87  158  167  185  211  216  253  336  33  38  73  92  94  148  171  193  223  241  338  363  20  65  209  236  294  316  385  440  442  530  568  589  596  656  664  695  14  75  116  133  185  209  259  277  305  308  310  340  416  465  473  499  504  552  626  633  665  673  742  840  855  859  954  994  1058  1087  1105  1158  1171  1198  1207  1246  1271  1283  1335  1374  1421  1437  17  20  23  43  69  109  151  200  231  233  242  308  356  366  434  442  445  463  472  491  519  527  541  604  620  638  659  756  775  8  71  74  129  153  197  219  277  336  376  393  394  397  407  409  421  434  595  84  149  275  313  21  50  120  127  130  151  180  181  8  40  56  143  144  157  174  207  348  403  31  156  161  174  265  275  318  353  407  59  112  118  121  129  191  192  21  22  84  90  94  4  41  46  82  193  6  70  72  76  121  136  229  276  277  300  328  333  361  405  421  425  455  524  526  574  737  815  874  888  912  926  928  931  41  46  65  66  94  98  152  169  212  255  279  306  318  328  417  443  461  462  480  535  557  564  595  598  677  29  133  228  258  262  266  318  341  360  396  397  401  437  18  34  39  100  116  280  281  291  328  342  363  406  417  423  428  497  518  614  665  682  758  807  820  982  1006  1099  1178  1183  1198  1219  1237  51  77  82  190  326  619  644  661  738  792  841  47  55  75  116  118  134  150  155  217  233  237  309  316  56  63  64  92  99  111  171  172  195  198  271  273  279  290  346  367  401  414  483  485  489  61  69  74  146  154  155  230  287  290  296  302  320  22  29  31  81  82  98  110  143  198  202  221  225  291  295  311  3  32  36  135  167  251  275  290  395  468  479  499  82  83  93  168  178  85  139  146  161  171  221  230  284  300  317  359  394  422  449  519  6  70  72  76  121  136  229  276  277  300  328  333  361  405  421  425  455  524  526  566  713  821  826  894  934  937  2  107  137  149  178  333  336  350  364  16  29  34  111  138  194  248  264  268  25  174  206  228  254  259  332  391  435  479  595  605  694  721  793  850  867  875  887  895  20  20  14  19  33  66  77  164  199  203  204  211  217  233  363  417  112  160  332  495  52  53  156  158  375  574  588  732  733  59  85  138  174  214  302  32  63  225  249  267  280  283  312  40  43  47  92  138  253  274  3  4  7  54  58  126  38  39  55  64  130  136  147  148  234  242  254  262  275  301  348  373  402  404  466  475  495  504  544  577  43  68  185  207  221  223  25  45  48  287  289  300  433  435  446  507  509  520  529  575  611  614  634  644  645  646  669  672  692  702  703  704  754  815  878  985  9  110  30  76  79  155  82  100  125  140  173  174  279  13  46  97  118  134  154  175  181  189  226  255  265  103  109  115  117  128  162  164  194  266  283  287  308  310  350  386  410  422  432  455  501  553  582  588  609  624  715  722  756  833  1308  1347  1375  1460  1488  1852  1878  131  200  214  225  237  244  262  265  314  371  389  397  448  463  60  162  221  261  267  25  44  189  200  236  252  270  280  287  407  41  67  75  95  112  120  127  245  257  263  269  273  283  289  294  19  116  121  138  146  29  46  51  56  63  66  89  94  135  140  145  152  155  175  21  44  85  87  94  119  124  171  179  189  231  456  459  489  500  520  568  578  685  694  40  135  148  159  229  231  308  309  324  382  440  448  502  34  269  284  288  351  393  468  124  132  165  186  220  231  239  241  254  255  261  337  342  348  398  427  439  487  498  579  644  661  666  705  720  724  18  46  47  87  107  147  178  426  460  478  546  554  567  578  608  628  658  690  727  745  746  759  760  795  815  895  907  987  1222  32  71  73  97  113  152  322  344  348  355  43  92  4  39  81  107  179  210  222  238  247  258  274  285  355  129  39  125  144  158  200  219  266  318  376  432  481  522  571  577  579  585  637  650  686  756  765  798  838  884  962  972  1001  1167  1206  1212  1286  1323  1380  1420  1460  1525  1529  1599  1608  1614  1619  1622  1628  1657  1682  1724  1782  1794  1837  1838  1857  1887  1935  1970  1975  1976  1981  2004  2011  2062  2111  2141  2161  2168  2181  2204  2210  2248  2250  2274  2278  2282  2393  2427  2449  2453  2482  2483  2521  2523  2567  2602  2605  2607  2612  2617  2622  2685  2692  2757  2759  2763  2764  2799  2859  2882  2971  3023  3031  3136  3143  3176  3184  3202  3209  3210  3221  3244  3287  3338  3347  3376  3382  3390  3405  3438  3459  3462  3665  3678  3692  3843  3900  3935  3947  4030  4045  4069  4079  4091  4104  4105  4121  4142  4159  4177  4240  4247  4261  4287  4328  4351  4392  4411  4449  4527  4560  4606  4640  53  155  156  166  203  217  242  260  268  309  417  421  425  445  453  462  485  510  552  562  570  582  152  307  317  341  369  382  397  410  425  438  453  466  485  509  537  550  565  593  621  17  65  71  118  132  199  257  304  314  323  386  549  551  557  567  587  633  682  687  728  729  731  745  815  840  872  876  905  985  989  1067  1107  1169  1214  1227  1272  1299  1313  1336  1364  1382  1413  1421  1432  1467  1569  1588  1635  1679  1694  1773  1841  1900  1927  1946  1983  2032  2053  2115  2116  2194  2222  2224  2258  2273  2274  2310  2321  2325  2340  2349  2380  2495  2567  2569  2582  2588  100  150  243  248  292  301  332  353  406  417  471  473  505  526  558  560  594  648  683  690  751  770  772  783  786  805  818  819  829  841  855  861  930  946  959  972  978  995  1000  1004  1053  1062  1121  1126  1158  1169  1173  1189  1212  1221  1236  34  89  95  98  121  168  252  315  571  572  588  907  939  1112  1116  1121  1408  1497  1538  1587  1621  1690  1718  1761  1814  1836  29  40  98  119  169  186  234  268  309  312  326  328  334  340  347  352  382  400  403  410  422  464  480  505  512  546  573  576  579  591  8  59  92  94  147  153  200  208  216  238  260  304  394  399  406  436  441  452  499  527  529  539  546  561  4  22  75  81  198  220  6  25  45  50  77  112  117  183  211  217  258  287  435  509  518  535  543  631  684  685  727  946  1032  1040  1048  1049  1078  1086  1090  1138  1152  1198  1294  1303  1366  1369  1381  1412  1477  1488  1606  1675  1690  1707  1708  1730  1741  1763  1817  1834  1869  1875  1881  1884  1904  1959  75  85  125  162  202  219  279  291  319  358  404  444  482  486  527  550  558  595  624  625  662  670  708  737  775  813  821  850  891  899  967  1017  1020  1040  1043  1051  1081  1130  1137  1141  1163  1251  1398  1412  1430  1499  1532  1535  1619  1620  1667  1706  1728  1928  2064  2106  2135  2199  2313  2354  2465  4  11  13  31  180  212  234  265  298  338  397  441  485  486  533  611  693  700  727  799  878  886  898  906  99  105  125  170  185  189  194  206  212  260  362  364  398  408  439  444  90  91  112  125  209  219  220  249  282  297  454  466  485  499  501  526  537  547  614  621  633  636  66  85  101  161  167  334  360  370  385  398  549  629  631  653  727  743  878  885  905  1028  1095  1147  1194  1211  1227  1236  1263  1265  1267  1312  1313  1404  1490  1563  1567  1579  1607  1623  1701  1724  11  74  82  95  98  118  127  141  154  237  251  264  280  284  347  362  363  379  545  554  627  641  653  684  735  801  813  24  41  48  77  78  94  104  121  186  192  244  316  319  343  366  418  473  487  7  12  131  145  202  283  290  82  112  159  220  221  241  270  285  409  421  424  439  43  100  107  113  121  176  212  229  249  13  14  15  27  35  63  67  74  78  88  89  94  116  124  178  186  7  35  81  109  136  167  173  175  195  34  41  61  79  103  120  156  162  163  220  232  242  64  124  47  50  74  95  97  133  16  90  116  119  155  199  213  223  225  272  300  312  314  318  342  346  373  407  411  4  20  42  45  88  161  181  183  267  299  306  334  390  432  544  649  650  665  715  139  157  206  215  3  71  73  37  40  43  67  123  126  389  407  427  434  762  836  842  867  894  948  1031 | GRQKGQTERYRGSEYTVEF  XXXXAQTVPYGIPLIKADK  QNTIGYPAKYDSVIAVGAV  XXXXXXXXMYKDLEGKVVV  TEKAKVVVNYRSKEDEANS  FLGSREAIKYFVENDIKGT  LMTETLALEYAPKGIRVNN  DVESMIPMGYIGEPEEIAA  AWLASSEASYVTGITLFAD  FEAPGRVTRYLSSQRLIKE  SFSQQAMKGYHAMMVDIAV  DTIGLCGFNYRFNSFYRDQ  GFNYRFNSFYRDQPHPFIT  QRANPDDPAYDENKRQFQE  EPLDDENIRYQIITFLIAG  TSGLLSFALYFLVKNPHVL  RVLVDPVPSYKQVKQLKYV  SYKQVKQLKYVGMVLNEAL  KEDTVLGGEYPLEKGDELM  HFDFEDHTNYELDIKETLT  AHNTPLLVLYGSNMGTAEG  GAVLIVTASYNGHPPDNAK  SADEVKGVRYSVFGCGDKN  CGDKNWATTYQKVPAFIDE  DASDDFEGTYEEWREHMWS  EHMWSDVAAYFNLDIENSE  EIELPKEASYQEGDHLGVI  DHLGVIPRNYEGIVNRVTA  TVSVEELLQYVELQDPVTR  LEALLEKQAYKEQVLAKRL  LTMLELLEKYPACEMKFSE  ALLPSIRPRYYSISSSPRV  LLPSIRPRYYSISSSPRVD  VVSGEAWSGYGEYKGIASN  GEAWSGYGEYKGIASNYLA  GEYKGIASNYLAELQEGDT  GQSLGEAHLYFGCRSPHED  FGCRSPHEDYLYQEELENA  CRSPHEDYLYQEELENAQS  SRMPNQPKTYVQHVMEQDG  ELLDQGAHFYICGDGSQMA  AVEATLMKSYADVHQVSEA  LQQLEEKGRYAKDVWAGXX  VESTTDALGYKHFRYAPVV  DALGYKHFRYAPVVNGVPI  VHVDKSDNVYAVNGELHNQ  KAIETKDGSYRLAYDVTIR  TKDGSYRLAYDVTIRYVEP  RLAYDVTIRYVEPEPANWE  LNISYEGGKYVLRDLSKPT  KPTGTQIITYDLQNRQSRL  SQRAAVDAHYNLGKVYDYF  DAHYNLGKVYDYFYSNFKR  HYNLGKVYDYFYSNFKRNS  NLGKVYDYFYSNFKRNSYD  FYSNFKRNSYDNKGSKIVS  GSKIVSSVHYGTQYNNAAW  VSSVHYGTQYNNAAWTGDQ  AAWTGDQMIYGDGDGSFFS  NESFSDVFGYFNDTEDWDI  LRSLSNPTKYNQPDNYANY  PTKYNQPDNYANYRNLPNT  YNQPDNYANYRNLPNTDEG  NLPNTDEGDYGGVHTNSGI  NSGIPNKAAYNTITKLGVS  LGVSKSQQIYYRALTTYLT  GVSKSQQIYYRALTTYLTP  QIYYRALTTYLTPSSTFKD  ALIQSARDLYGSTDAAKVE  XXXXXXXXMYRYLGEALLL  XXXXXXMYRYLGEALLLSR  GLVPPARRHYSEAAADRED  DGSWEVIEGYRAQHSQHRT  RTPCKGGIRYSTDVSVDEV  VKALASLMTYKCAVVDVPF  AGVKINPKNYTDNELEKIT  REMSWIADTYASTIGHYDI  DTYASTIGHYDINAHACVT  IENFINEASYMSILGMTPG  ILGFPKAKIYEGSILEVDC  RNIMVIPDLYLNAGGVTVS  LNAGGVTVSYFEWLNNLNH  WLNNLNHVSYGRLTFKYER  KDIVHSGLAYTMERSARQI  LGLDLRTAAYVNAIEKVFR  VFLEAHGIRYRIMIEDVQS  RARSTNTFNYATYHTLDEI  STNTFNYATYHTLDEIYDF  ATYHTLDEIYDFMDLLVAE  VSKLQIGRSYEGRPIYVLK  GRSYEGRPIYVLKFSTGGS  WFAKKFTEDYGQDPSFTAI  ASSSPCSETYHGKYANSEV  PCSETYHGKYANSEVEVKS  IHSYSQLLLYPYGYTTQSI  SYSQLLLYPYGYTTQSIPD  SQLLLYPYGYTTQSIPDKT  SAVEALKSLYGTSYKYGSI  ALKSLYGTSYKYGSIITTI  KSLYGTSYKYGSIITTIYQ  ASGGSIDWSYNQGIKYSFT  DWSYNQGIKYSFTFELRDT  TFELRDTGRYGFLLPASQI  IMEHTLNNLYXXXXXXXXX  IAKVFKNSKYNSLTINNDI  TCSTSTPGVYARVTALVNW  SLLAFTSACYFQNCPRGGK  PAEPAQPGVYXXXXXXXXX  EESIISQETYKQEKNMAIN  VVRNANEEEYSIGSSSEES  VKITVDDKHYQKALNEINQ  KALNEINQFYQKFPQYLQY  NQFYQKFPQYLQYLYQGPI  YQKFPQYLQYLYQGPIVLN  KFPQYLQYLYQGPIVLNPW  NFLKKISQRYQKFALPQYL  RYQKFALPQYLKTVYQHQK  IQPKTKVIPYVRYLXXXXX  KTKVIPYVRYLXXXXXXXX  PFAQTQSLVYPFPGPIPNS  KHKEMPFPKYPVEPFTESQ  LPVPQKAVPYPQRDMPIQA  DIQKVAGTWYSLAMAASDI  DAQSAPLRVYVEELKPTPE  LVLDTDYKKYLLFCMENSA  SLLLLFSSAYSRGVFRRDT  IHGWTVTGMYESWVPKLVA  MNWMADEFNYPLGNVHLLG  PLGNVHLLGYSLGAHAAGI  QKPVGHVDIYPNGGTFQPG  LNEENPSKAYRCNSKEAFE  VRAKRSSKMYLKTRSQMPY  HFSGTESNTYTNQAFEISL  TNQAFEISLYGTVAESENI  LPEVSTNKTYSFLLYTEVD  TNKTYSFLLYTEVDIGELL  LKLKWISDSYFSWSNWWSS  HRGTEKLIEYKTYLQALPY  TEKLIEYKTYLQALPYFDR  YKTYLQALPYFDRLDYVSM  ALPYFDRLDYVSMMCNEQA  VSMMCNEQAYSLAVEKLLN  EEREKMFEFYERVSGARMH  VSGARMHAAYVRPGGVHQD  LPLGLMDDIYEFSKNFSLR  IVTAEDALNYGFSGVMLRG  QWDLRKTQPYDVYDQVEFD  LRKTQPYDVYDQVEFDVPI  VPIGSRGDCYDRYLCRVEE  GSRGDCYDRYLCRVEEMRQ  ESLIHHFKLYTEGYQVPPG  HHFKLYTEGYQVPPGATYT  GYQVPPGATYTAIEAPKGE  EAPKGEFGVYLVSDGSSRP  LVSDGSSRPYRCKIKAPGF  HKQLSAFGEYVAEILPKYV  EYVAEILPKYVQQVQVSCF  TRQNRFEIVYNLLSLRFNS  FNSRIRVKTYTDELTPIES  TPIESSVPVYKAANWYERE  VPVYKAANWYEREIWDMFG  PDLRRILTDYGFEGHPFRK  FRKDFPLSGYVELRYDDEV  PLSGYVELRYDDEVKRVVA  EEDNWRWHFYDTVKGSDWL  PASVVELENYGMPFSRTED  FSRTEDGKIYQRAFGGQSL  TGHSLLHTLYGRSLRYDTS  HTLYGRSLRYDTSYFVEYF  GRSLRYDTSYFVEYFALDL  RYDTSYFVEYFALDLLMES  RNTVIATGGYGRTYFSCTS  IATGGYGRTYFSCTSAHTS  FVQFHPTGIYGAGCLITEG  SQGERFMERYAPVAKDLAS  GCGPEKDHVYLQLHHLPPA  PIPVLPTVHYNMGGIPTNY  YNMGGIPTNYKGQVLRHVN  GQDQGVPGLYACGEAACAS  EGCEKISSLYGDLRHLKTF  LMLCALQTIYGAEARKESR  EDFKERVDEYDYSKPIQGQ  FKERVDEYDYSKPIQGQQK  QHWRKHTLSYVDIKTGKVT  IKTGKVTLEYRPVIDRTLN  ATVPPAIGSYXXXXXXXXX  LPTTLDPAEYDISSETRKA  EDPALVRWTYARSANIYPN  WTYARSANIYPNFRPNTKT  FGIGPLVFWYYVFKTDRDR  GIGPLVFWYYVFKTDRDRK  PFVFAPAWSYNLDVRHVQN  FPLAGRHFGYRVLQVGNGV  SEGNSMGNLYQCQPETGDC  CLPVTLSSNYTSKYLGMTL  TLSSNYTSKYLGMTLATDP  LSRTCDQNIYLSGLCYLIH  QNIYLSGLCYLIHENLRGP  GPVLQGHPGYQECIKGNVD  VMKKLSNSSYQFAAVQFST  QFAAVQFSTYFRTEFTFLD  FRTEFTFLDYIRQKDPDAL  LTNTFGAINYVAKEVFRPD  IDAAKDIIRYIIGIGKNFK  LFTELQKKIYVIEGTSKQD  PLTVESRAGYLGYTVTWLP  VESRAGYLGYTVTWLPSRG  SLLATGAPRYQHVGRVLLF  EIDGIQIGSYFGGELCGVD  ELLLIAAPLYYGEQRGGRV  LLLIAAPLYYGEQRGGRVF  EQRGGRVFIYQKIQLEFQM  VSELQGETGYPLGRFGAAI  APLEEQGAVYIFNGQQGGL  IPVHEVECSYSTSNQKKEG  QGHLVANLTYTLQLDGHRT  ISPINVSLSYSLWEEEGTP  TLRNTAEDAYWVQVTLSFP  SATTSIPVMYPINVLTKDQ  TKDQENSTLYISFTPKSPK  PKIHHVKHIYQVRIQPSNY  YQVRIQPSNYDNMPPLEAL  QVVMKVDLVYEKEMLYLYV  DLVYEKEMLYLYVLSGIGG  VYEKEMLYLYVLSGIGGLL  LLFLIFIALYKVGFFKRNL  STSAASSSNYCNQMMKSRN  DCRETGSSKYPNCAYKTTQ  GSSKYPNCAYKTTQANKHI  EAKRRVRELYRAWYREVPN  RVRELYRAWYREVPNTVHL  GSRDFVFDLYRALAAAAPA  AFLGAMRTLYLADTFPTNF  EGAKKKINDYVAKQTKGKI  GTQVMVMVNYIFFKAKWET  LKSTHEQDFYVTPETVVRV  PMMKQQDQFYYLLDRNLSC  MMKQQDQFYYLLDRNLSCK  LSCKVVGVPYQGNATAFFI  LPKFSIEGSYQLEEVLPKL  VTIVCLDLGYTIVCHTTAT  DLLNAAGETYEVALTEAGT  EVALTEAGTYSFYCAPHQG  LFVAICLFVYMGLSFGDFS  SFGDFSIVGYSQNDLTSTE  SWMLKHNKIYKNIDEKIYR  IYKNIDEKIYRFEIFKDNL  FEIFKDNLKYIDETNKKNN  DETNKKNNSYWLGLNVFAD  MSNDEFKEKYTGSIAGNYT  KYTGSIAGNYTTTELSYEE  GNYTTTELSYEEVLNDGDV  KIRTGNLNEYSEQELLDCD  RRSYGCNGGYPWSALQLVA  WSALQLVAQYGIHYRNTYP  QLVAQYGIHYRNTYPYEGV  QYGIHYRNTYPYEGVQRYC  GIHYRNTYPYEGVQRYCRS  TYPYEGVQRYCRSREKGPY  QPYNEGALLYSIANQPVSV  EAAGKDFQLYRGGIFVGPC  VDHAVAAVGYGPNYILIKN  VAAVGYGPNYILIKNSWGT  WGTGWGENGYIRIKRGTGN  RIKRGTGNSYGVCGLYTSS  GNSYGVCGLYTSSFYPVKN  VCGLYTSSFYPVKNXXXXX  XXXXMANLSYWLLALFVAT  GGWNTGGSRYPGQGSPGGN  GQGSPGGNRYPPQGGGTWG  HFGNDWEDRYYRENMNRYP  FGNDWEDRYYRENMNRYPN  RYYRENMNRYPNQVYYRPV  QVYYRPVDQYNNQNNFVHD  PYSLNEQLCYVDLGNEYPV  GQPAYAPGLYTVHLSSFKV  MEQRITLKDYAMRFGQTKT  SSPIAGNIDYTISTPGSNY  YTISTPGSNYAVGDKITVK  AVGDKITVKYVSDDIETEG  KINIPTGKNYAKAKEVGEY  YAKAKEVGEYPTLGSNWTA  VDFQANLKKYGIPGVVALY  EIEIVSKADYAKGASALLP  GYGPQTDSQYAIIVRRNDA  EKDIYDSNIYIDDFFAKGG  DFFAKGGSEYIFATAQNWP  VNWRTAAGSYTDNNFNISS  DNNFNISSTYAAIDGNHKY  AIDGNHKYQYDKYNDVNRW  TRQAQRDRLYQEAINPVTG  QGNKALGGIYEYRVVCDTT  NKALGGIYEYRVVCDTTNN  DRNEFVATFYIQPARSINY  YIQPARSINYITLNFVATA  QRVSGLIINYPLDDQDAIA  RLRLAGWHKYLTRNQIQPI  VGADISVVGYDDTEDSSCY  YDDTEDSSCYIPPLTTIKQ  VGKEFPLPTYATSGSAGLD  XXXXMANPLYQKHIISIND  ADTISVISTYVDAIVMRHP  HVAMVGDLKYGRTVHSLTQ  LAKFDGNRFYFIAPDALAM  APDALAMPQYILDMLDEKG  EVMAEVDILYMTRVQKERL  DVDKTPHAWYFQQAGNGIF  EGAWNSSTQYEGGNKGHRP  TKKADEIQIYKYVVHNVAH  KADEIQIYKYVVHNVAHRF  AGLSEQALYYIGGVIKHAK  ALANPTTNSYKRLVPGYEA  RFPDPAANPYLCFAALLMA  VFTDEAIDAYIALRREEDD  TPHPVEFELYYSVXXXXXX  PHPVEFELYYSVXXXXXXX  MSFELPALPYAKDALAPHI  PHISAETIEYHYGKHHQTY  ISAETIEYHYGKHHQTYVT  AAQVWNHTFYWNCLAPNAG  LTVDVWEHAYYIDYRNARP  TVDVWEHAYYIDYRNARPG  VWEHAYYIDYRNARPGYLE  IDYRNARPGYLEHFWALVN  GVTASSDTFYPGQERYDTY  YPGQERYDTYSGRVVRHFK  EWQAMGVMNYEMESATLLT  VALLLGIEAYAAEETFDTH  DDNQPLPGQYDIDIYVNKQ  LPGQYDIDIYVNKQWRGKY  YVNKQWRGKYEIIVKDNPQ  FEQLVQGGSYTWDIGVFRL  AWVEELESGYVPPENWERG  NWERGINAFYTSYYLSQYY  RGINAFYTSYYLSQYYSDY  GINAFYTSYYLSQYYSDYK  FYTSYYLSQYYSDYKASGN  YTSYYLSQYYSDYKASGNN  YYLSQYYSDYKASGNNKST  KASGNNKSTYVRFNSGLNL  PGVWKSNTLYLERGFAQLL  LGTLRVGDMYTSSDIFDSV  TIEQNGFVVYQKEVPPGPF  KEADGSVTTYLVPYAAVPN  GSVTTYLVPYAAVPNMLQP  NMLQPGVSKYDLAAGRSHI  KQSDFVQAGYQYGFNNLLT  SDFVQAGYQYGFNNLLTLY  YGFNNLLTLYGGSMVANNY  YGGSMVANNYYAFTLGAGW  GGSMVANNYYAFTLGAGWN  NGDVFDGQSYQIAYNKFVS  FDGQSYQIAYNKFVSQTST  TRFGLAAWRYSSRDYRTFN  AAWRYSSRDYRTFNDHVWA  HVWANNKDNYRRDENDVYD  NYRRDENDVYDIADYYQND  ENDVYDIADYYQNDFGRKN  NDVYDIADYYQNDFGRKNS  VSLSTLWRDYWGRSGSSKD  WGRSGSSKDYQLSYSNNLR  GSSKDYQLSYSNNLRRISY  YSNNLRRISYTLAASQAYD  SYTLAASQAYDENHHEEKR  DVSTPRRQIYMSNSTTFDD  TVGSRDQFNYGVNLSHQHQ  APVATVNGSYSQSSTYRQA  NGSYSQSSTYRQAGASVSG  MNAPGIKDAYVNGQKYRTT  KDAYVNGQKYRTTNRNGVV  TTNRNGVVIYDGMTPYREN  VVIYDGMTPYRENHLMLDV  ADGQSLTFGYEVNDIHGHN  GKEIDESRNYICQXXXXXX  IVLQRTRVIYDASRKEAAL  VANKGAETPYLLQSWVDNI  LPENKESLFYINVRAIPAK  KRSDHSLNIYNPTEYYVVF  SLNIYNPTEYYVVFAGLAV  LNIYNPTEYYVVFAGLAVD  KTDLTSKIEYIAPGEHKQL  NVKWAAINDYGGSSGTETR  AGLFVSSLSYAENTEIPSY  YAENTEIPSYEEGISLFDV  PDGDAALLNYHIVGVTRDG  KQRFLPQGDYHYQIHYQVK  RFLPQGDYHYQIHYQVKNA  QGDYHYQIHYQVKNAFLRE  TGNHWPFEIYKTRFSLQFS  LFTGEEGDTYRNGRILEDG  GRIESRDPFYREDFTVLYR  FYREDFTVLYRWPHALLSN  CLFLVCGWLYLWKRRPQFT  IETDVIPPDYTPGMLRLDA  MLRLDAKLVYDDKGFCADI  RAFLRMQKFYLPRKKSSFY  YLPRKKSSFYRSDTFLQWG  GLAILAVILYGNLSPVGWA  PLICSGVAFYSLYINVGDV  CSGVAFYSLYINVGDVFFY  YINVGDVFFYWYMPAGYFT  NVGDVFFYWYMPAGYFTAV  FFYWYMPAGYFTAVCLTGY  YFTAVCLTGYLTGMGYIFL  LTGYLTGMGYIFLPKFTQT  KFTQTGQQRYAHGEAIVNY  YAHGEAIVNYLARKEAATH  RKGETRKLDYALLGWAISA  ANTSLLGRSYSGGGAGGGA  FGQPVNVARYDQQKYDIFE  NVARYDQQKYDIFEKLIEK  KHIFISNLKYQTLLDSIQG  QKRAEGISSYYDELIEMTS  KRAEGISSYYDELIEMTSY  YDELIEMTSYWHLLGEGTH  SLRELKKKLYLCLMSVNAL  VNALEAIRFYVSFACSFAF  IAEECKQECYDLFVQAAQQ  GLNKDILCQYVEYITNIRM  KDILCQYVEYITNIRMQAV  APQEVEVSSYLVGQIDSEV  TVLTKASAEYSTQKDQLSR  SRFQATRPFYREAVRHGLI  WPSNSGGWDYKGADLACVV  TIANNKKDAYQILGEPELA  LEMAMQTKIYTTDVAVECV  AMKAVGMKSYAKDMSFPRL  MQRVMALEDYEPWAATYGS  EDYEPWAATYGSSKVDKSR  VSDPDAKKFYAITTLVPAI  LVPAIAFTMYLSMLLGYGL  PFGGEQNPIYWARYADWLF  EQNPIYWARYADWLFTTPL  WAISTAAMLYILYVLFFGF  STAAMLYILYVLFFGFTSK  NVTVVLWSAYPVVWLIGSE  KFIIPNVVKYSPNCKLLIV  VSNPVDILTYVAWKISGFP  CNLDSARFRYLMGERLGVH  AYEVIKLKGYTSWAIGLSV  PVSTMIKGLYGIKDDVFLS  PPAAGAVASYDYLVIGGGS  AAGAVASYDYLVIGGGSGG  SEFMHDHADYGFPSCEGKF  PTIEVSGKKYTAPHILIAT  GRSVIVGAGYIAVEMAGIL  FQNTNVKGIYAVGDVCGKA  RKLAHRLFEYKEDSKLDYN  EYKEDSKLDYNNIPTVVFS  LTEDEAIHKYGIENVKTYS  KYGIENVKTYSTSFTPMYH  TYSTSFTPMYHAVTKRKTK  DCGPESSKKYAEAVTRAKQ  ICTMFHTSGYDTQAIVENN  IVENNESTEYGLFQISNKL  RECVEETCSYEEAFEALES  TATDVFWAKYTACETARTP  NCAEGLGTNYRGHVNITRS  IECQLWRSRYPHKPEINST  DSSTTGPWCYTTDPTVRRQ  QCVPDRGQQYQGRLAVTTH  DGDEEGVWCYVAGKPGDFG  VAGKPGDFGYCDLNYCEEA  GDFGYCDLNYCEEAVEEET  IEGRTATSEYQTFFNPRTF  KTERELLESYIDGRIVEGS  VLTAAHCLLYPPWDKNFTE  RIGKHSRTRYERNIEKISM  EKISMLEKIYIHPRYNWRE  LEKIYIHPRYNWRENLDRD  LKKPVAFSDYIHPVCLPDR  TAASLLQAGYKGRVTGWGN  MKSPFNNRWYQMGIVSWGE  GEGCDRDGKYGFYTHVFRL  CDRDGKYGFYTHVFRLKKW  SGQGEPLDDYVNTQGASLF  EEFTCRAFQYHSKEQQCVI  DVVLFEKKVYLSECKTGNG  ECKTGNGKNYRGTMSKTKN  HPSEGLEENYCRNPDNDPQ  DNDPQGPWCYTTDPEKRYD  CYTTDPEKRYDYCDILECE  TTDPEKRYDYCDILECEEE  ECMHCSGENYDGKISKTMS  DSQSPHAHGYIPSKFPNKN  FPNKNLKKNYCRNPDRELR  TPPPSSGPTYQCLKGTGEN  QCLKGTGENYRGNVAVTVS  FPCKNLDENYCRNPDGKRA  TTNSQVRWEYCKIPSCDSS  ELTPVVQDCYHGDGQSYRG  DCYHGDGQSYRGTSSTTTT  TTDPSVRWEYCNLKKCSGT  DCMFGNGKGYRGKRATTVT  NPRAGLEKNYCRNPDGDVG  DGDVGGPWCYTTNPRKLYD  CYTTNPRKLYDYCDVPQCA  TTNPRKLYDYCDVPQCAAP  LEKSPRPSSYKVILGAHQE  IPACLPSPNYVVADRTECF  VIENKVCNRYEFLNGRVQS  PLVCFEKDKYILQGVTSWG  CARPNKPGVYVRVSRFVTW  CEIDKSKTCYEGNGHFYRG  TCYEGNGHFYRGKASTDTM  NSATVLQQTYHAHRSDALQ  LQLGLGKHNYCRNPDNRRR  PDNRRRPWCYVQVGLKPLV  ENQPWFAAIYRRHRGGSVT  RRHRGGSVTYVCGGSLISP  ISATHCFIDYPKKEDYIVY  FIDYPKKEDYIVYLGRSRL  YPKKEDYIVYLGRSRLNSN  VENLILHKDYSADTLAHHN  IQTICLPSMYNDPQFGTSC  GFGKENSTDYLYPEQLKMT  GKENSTDYLYPEQLKMTVV  SHRECQQPHYYGSEVTTKM  HRECQQPHYYGSEVTTKML  CALKDKPGVYTRVSHFLPW  LPLALGSPMYSIITPNILR  VVLVSLQSGYLFIQTDKTI  LFIQTDKTIYTPGSTVLYR  IYTPGSTVLYRIFTVNHKL  NMGQWKIRAYYENSPQQVF  MGQWKIRAYYENSPQQVFS  FSTEFEVKEYVLPSFEVIV  VIVEPTEKFYYIYNEKGLE  IVEPTEKFYYIYNEKGLEV  EPTEKFYYIYNEKGLEVTI  EVTITARFLYGKKVEGTAF  AEDLVGKSLYVSATVILHS  SGIPIVTSPYQIHFTKTPK  QIHFTKTPKYFKPGMPFDL  VTNPDGSPAYRVPVAVQGE  ATRTMQALPYSTVGNSNNY  YSTVGNSNNYLHLSVLRTE  DRAHEAKIRYYTYLIMNKG  RAHEAKIRYYTYLIMNKGR  HEAKIRYYTYLIMNKGRLL  FIPSFRLVAYYTLIGASGQ  IPSFRLVAYYTLIGASGQR  GCTPGSGKDYAGVFSDAGL  DFFIDLRLPYSVVRNEQVE  EQVEIRAVLYNYRQNQELK  VEIRAVLYNYRQNQELKVR  PPKSSLSVPYVIVPLKTGL  QEVEVKAAVYHHFISDGVR  MTPTVIAVHYLDETEQWEK  GALELIKKGYTQQLAFRQP  RAPSTWLTAYVVKVFSLAV  KAGDFLEANYMNLQRSYTV  ANYMNLQRSYTVAIAGYAL  RSYTVAIAGYALAQMGRLK  RWEDPGKQLYNVEATSYAL  QLYNVEATSYALLALLQLK  VVRWLNEQRYYGGGYGSTQ  VRWLNEQRYYGGGYGSTQA  NEQRYYGGGYGSTQATFMV  FMVFQALAQYQKDAPDHQE  QGTLSVVTMYHAKAKDQLT  TMILEICTRYRGDQDATMS  KQLANGVDRYISKYELDKA  NGVDRYISKYELDKAFSDR  FSDRNTLIIYLDKVSHSED  DCLAFKVHQYFNVELIQPG  LIQPGAVKVYAYYNLEESC  QPGAVKVYAYYNLEESCTR  PGAVKVYAYYNLEESCTRF  NLEESCTRFYHPEKEDGKL  DKACEPGVDYVYKTRLVKV  ACEPGVDYVYKTRLVKVQL  VQLSNDFDEYIMAIEQTIK  ALKLEEKKHYLMWGLSSDF  FWGEKPNLSYIIGKDTWVE  GKTWGQEQTYVISAPKIFR  ASENIVIQVYGYTEAFDAT  ENIVIQVYGYTEAFDATIS  FDATISIKSYPDKKFSYSS  KSYPDKKFSYSSGHVHLSS  LPGGQNPVSYVYLEVVSKH  GGQNPVSYVYLEVVSKHFS  SKSKRMPITYDNGFLFIHT  LFIHTDKPVYTPDQSVKVR  PDQSVKVRVYSLNDDLKPA  DFKIPSNPRYGMWTIKAKY  YGMWTIKAKYKEDFSTTGT  EDFSTTGTAYFEVKEYVLP  GTAYFEVKEYVLPHFSVSI  HFSVSIEPEYNFIGYKNFK  IEPEYNFIGYKNFKNFEIT  NFEITIKARYFYNKVVTEA  EITIKARYFYNKVVTEADV  NKVVTEADVYITFGIREDL  SETAVKELSYYSLEDLNNK  ETAVKELSYYSLEDLNNKY  YSLEDLNNKYLYIAVTVIE  LEDLNNKYLYIAVTVIEST  EEAEIPGIKYVLSPYKLNL  PGIKYVLSPYKLNLVATPL  PLFLKPGIPYPIKVQVKDS  PEENQAREGYRAIAYSSLS  AREGYRAIAYSSLSQSYLY  IAYSSLSQSYLYIDWTDNH  YSSLSQSYLYIDWTDNHKA  NIIVTPKSPYIDKITHYNY  SPYIDKITHYNYLILSKGK  YIDKITHYNYLILSKGKII  TREKFSDASYQSINIPVTQ  MVPSSRLLVYYIVTGEQTA  VPSSRLLVYYIVTGEQTAE  VHLSPDADAYSPGQTVSLN  ALAAVDSAVYGVQRGAKKP  KKIEEIAAKYKHSVVKKCC  PVSKPEIRSYFPESWLWEV  DVFLEMNIPYSVVRGEQIQ  EQIQLKGTVYNYRTSGMQF  IQLKGTVYNYRTSGMQFCV  VPEGVKRESYSGVTLDPRG  GVTLDPRGIYGTISRRKEF  TISRRKEFPYRIPLDLVPK  ELMSVVPVFYVFHYLETGN  VVPVFYVFHYLETGNHWNI  KEGMLSIMSYRNADYSYSV  SIMSYRNADYSYSVWKGGS  MSYRNADYSYSVWKGGSAS  LRVLGQVNKYVEQNQNSIC  NSLLWLVENYQLDNGSFKE  NGSFKENSQYQPIKLQGTL  PVEARENSLYLTAFTVIGI  STFTLAISAYALSLGDKTH  ALVKGNPPIYRFWKDNLQH  TARMVETTAYALLTSLNLK  TSLNLKDINYVNPVIKWLS  IKWLSEEQRYGGGFYSTQD  EEQRYGGGFYSTQDTINAI  INAIEGLTEYSLLVKQLRL  RLSMDIDVSYKHKGALHNY  YKHKGALHNYKMTDKNFLG  STSEEVCSFYLKIDTQDIE  DTQDIEASHYRGYGNSDYK  DIEASHYRGYGNSDYKRIV  HYRGYGNSDYKRIVACASY  YKRIVACASYKPSREESSS  EGVDQLFTDYQIKDGHVIL  FLSPATFTVYEYHRPDKQC  SPATFTVYEYHRPDKQCTM  RPDKQCTMFYSTSNIKIQK  QTACKPEIAYAYKVSITSI  ACKPEIAYAYKVSITSITV  ITVENVFVKYKATLLDIYK  KYKATLLDIYKTGEAVAEK  NAELVKGRQYLIMGKEALQ  MGKEALQIKYNFSFRYIYP  QIKYNFSFRYIYPLDSLTW  KYNFSFRYIYPLDSLTWIE  PLDSLTWIEYWPRDTTCSS  LIQNHFVDEYDPTIEDSYR  ILDTAGQEEYSAMRDQYMR  EEYSAMRDQYMRTGEGFLC  DLARSYGIPYIETSAKTRQ  AALGCLVKDYFPEPVTVSW  PAVLQSSGLYSLSSVVTVP  PSSSLGTQTYICNVNHKPS  AKTKPREEQYNSTYRVVSV  PREEQYNSTYRVVSVLTVL  KGQPREPQVYTLPPSRDEL  SLTCLVKGFYPSDIAVEWE  ESNGQPENNYKTTPPVLDS  LDSDGSFFLYSKLTVDKSR  VMHEALHNHYTQKSLSLSP  TRGPPGASGYPGNPGLPGI  SPGFPGEPGYPGLIGRQGP  SPGPPGQPGYTNGIVECQP  SCLICDIDGYRGPPGPQGP  DTGPPGPPGYGPAGPIGDK  LKGSPGSVGYPGSPGLPGE  PGPPGPPGPYDIIKGEPGL  QCPSGTKILYHGYSLLYVQ  SGTKILYHGYSLLYVQGNE  ILYHGYSLLYVQGNERAHG  VCNFASRNDYSYWLSTPEP  NFASRNDYSYWLSTPEPMP  SGWSSLWIGYSFVMHTSAG  ECHGRGTCNYYANAYSFWL  CHGRGTCNYYANAYSFWLA  GTCNYYANAYSFWLATIER  EEVKAKVQPYLDDFQKKWQ  KKWQEEMELYRQKVEPLRA  APPPISGGGYRARPAKAAA  SQTSSSSFQYMYLLKDLWQ  TSSSSFQYMYLLKDLWQKR  KDNENVVNEYSSELEKHQL  SSELEKHQLYIDETVNSNI  ESDVSAQMEYCRTPCTVSC  RKGGETSEMYLIQPDSSVK  IQPDSSVKPYRVYCDMNTE  DSSVKPYRVYCDMNTENGG  VDFGRKWDPYKQGFGNVAT  VATNTDGKNYCGLPGEYWL  WKGDKVKAHYGGFTVQNEA  FTVQNEANKYQISVNKYRG  NKYQISVNKYRGTAGNALM  IHNGMFFSTYDRDNDGWLT  SKEDGGGWWYNRCHAANPN  CHAANPNGRYYWGGQYTWD  HAANPNGRYYWGGQYTWDM  VWMNWKGSWYSMRKMSMKI  ATRFKHLRKYTYNYEAESS  RFKHLRKYTYNYEAESSSG  KHLRKYTYNYEAESSSGVP  EEFAAAMSRYELKLAIPEG  LYPEKDEPTYILNIKRGII  KQVLFLDTVYGNCSTHFTV  LISSSQSCQYTLDAKRKHV  EQHLFLPFSYKNKYGMVAQ  RDQRSRATLYALSHAVNNY  YALSHAVNNYHKTNPTGTQ  TQELLDIANYLMEQIQDDC  QDDCTGDEDYTYLILRVIG  DCTGDEDYTYLILRVIGNM  SPGDKRLAAYLMLMRSPSQ  MDFRKFSRNYQLYKSVSLP  RKFSRNYQLYKSVSLPSLD  SKVLVDHFGYTKDDKHEQD  SKEVPEARAYLRILGEELG  GSKNDFFLHYIFMENAFEL  CKQVFPGLNYCTSGAYSNA  GLNYCTSGAYSNASSTDSA  NASSTDSASYYPLTGDTRL  ASSTDSASYYPLTGDTRLE  LRPTGEIEQYSVSATYELQ  IEQYSVSATYELQREDRAL  QTEATMTFKYNRQSMTLSS  DESTEGKTSYRLTLDIQNK  LQMDSSATAYGSTVSKRVA  TVSKRVAWHYDEEKIEFEW  SNFPVDLSDYPKSLHMYAN  SDYPKSLHMYANRLLDHRV  LQKASGSLPYTQTLQDHLN  FLKSDGRVKYTLNKNSLKI  VPTFTIPKLYQLQVPLLGV  GVLDLSTNVYSNLYNWSAS  LSTNVYSNLYNWSASYSGG  SNLYNWSASYSGGNTSTDH  TDHFSLRARYHMKADSVVD  ADSVVDLLSYNVQGSGETT  NVQGSGETTYDHKNTFTLS  DHKNTFTLSYDGSLRHKFL  DGQFRVSSFYAKGTYGLSC  VSSFYAKGTYGLSCQRDPN  ESNLRFNSSYLQGTNQITG  QGTNQITGRYEDGTLSLTS  IIKNTASLKYENYELTLKS  NTASLKYENYELTLKSDTN  TLKSDTNGKYKNFATSNKM  KQNALLRSEYQADYESLRF  LLRSEYQADYESLRFFSLL  LTELSLGSAYQAMILGVDS  KLSNDMMGSYAEMKFDHTN  DFSSKLDNIYSSDKFYKQT  DNIYSSDKFYKQTVNLQLQ  QTVNLQLQPYSLVTTLNSD  VTTLNSDLKYNALDLTNNG  HVAGNLKGAYQNNEIKHIY  YQNNEIKHIYAISSAALSA  ISSAALSASYKADTVAKVQ  ASAIDMSTNYNSDSLHFSN  LWGEHTGQLYSKFLLKAEP  PLAFTFSHDYKGSTSHHLV  LKTQFNNNEYSQDLDAYNT  NEYSQDLDAYNTKDKIGVE  EFTIVAFVKYDKNQDVHSI  LPFFETLQEYFERNRQTII  INIDQFVRKYRAALGKLPQ  GKLPQQANDYLNSFNWERQ  EKLTALTKKYRITENDIQI  NEKLSQLQTYMIQFDQYIK  QTYMIQFDQYIKDSYDLHD  QFDQYIKDSYDLHDLKIAI  EKLKSLDEHYHIRVNLVKT  SWIQNVDTKYQIRIQIQEK  AKVHELIERYEVDQQIQVL  DKLVELAHQYKLKETIQKL  VLQQVKIKDYFEKLVGFID  LIKKLKSFDYHQFVDETND  EETKATVAVYLESLQDTKI  MDIQQELQRYLSLVGQVYS  RYLSLVGQVYSTLVTYISD  GQVYSTLVTYISDWWTLAA  KNLTDFAEQYSIQDWAKRM  ELQWPVPDIYLRDLKVEDI  IEVPTFGKLYSILKIQSPL  KESVKFSSKYLRTEHGSEM  QLTLDSNTKYFHKLNIPKL  HLRVNQNLVYESGSLNFSK  TGKIDFLNNYALFLSPSAQ  WQVSARFNQYKYNQNFSAG  VSARFNQYKYNQNFSAGNN  SFDLSVKAQYKKNKHRHSI  NALDFVTKSYNETKIKFDK  NETKIKFDKYKAEKSHDEL  LPRTFQIPGYTVPVVNVEV  FTIEMSAFGYVFPKAVSMP  FIPAMGNITYDFSFKSSVI  SSSVIDALQYKLEGTTRLT  TVSSSMEFKYDFNSSMLYS  KYDFNSSMLYSTAKGAVDH  VKGSVLSREYSGTIASEAN  GTIASEANTYLNSKSTRSS  AGEATLQRIYSLWEHSTKN  FLKNIILPVYDKSLWDFLK  HLRVSTAFVYTKNPNGYSF  FVYTKNPNGYSFSIPVKVL  FPEVDVLTKYSQPEDSLIP  ARFEVDSPVYNATWSASLK  SASLKNKADYVETVLDSTC  CSSTVQFLEYELNVLGTHK  FAHRDFSAEYEEDGKYEGL  SAEYEEDGKYEGLQEWEGK  PAFTDLHLRYQKDKKGIST  DDDFSKWNFYYSPQSSPDK  DDFSKWNFYYSPQSSPDKK  NVPKATGVLYDYVNKYHWE  PKATGVLYDYVNKYHWEHT  EWKDKAQNLYQELLTQEGQ  EVGTVLSQVYSKVHNGSEI  HNGSEILFSYFQDLVITLP  HKLIDVISMYRELLKDLSK  KQLKEMKFTYLINYIQDEI  EMKFTYLINYIQDEINTIF  DEINTIFSDYIPYVFKLLK  NTIFSDYIPYVFKLLKENL  SQELQQIHQYIMALREEYF  QYIMALREEYFDPSIVGWT  PSIVGWTVKYYELEEKIVS  SIVGWTVKYYELEEKIVSL  VALKDFHSEYIVSASNFTS  QFLHRNIQEYLSILTDPDG  IATKKIISDYHQQFRYKLQ  ISDYHQQFRYKLQDFSDQL  QDFSDQLSDYYEKFIAESK  DFSDQLSDYYEKFIAESKR  RLIDLSIQNYHTFLIYITE  IQNYHTFLIYITELLKKLQ  LQSTTVMNPYMKLAPGELT  SRQLAPALGYLGSRQKHSL  QKHSLPDLPYDYGALEPHI  HSLPDLPYDYGALEPHINA  LGIDVWEHAYYLQYKNVRP  GIDVWEHAYYLQYKNVRPD  VWEHAYYLQYKNVRPDYLK  LQYKNVRPDYLKAIWNVIN  SSSVPSQKTYQGSYGFRLG  PSQKTYQGSYGFRLGFLHS  GTAKSVTCTYSPALNKMFC  GTRVRAMAIYKQSQHMTEV  RVEGNLRVEYLDDRNTFRH  TFRHSVVVPYEPPEVGSDC  VGSDCTTIHYNYMCNSSCM  SDCTTIHYNYMCNSSCMGG  SKQITADKQYKGIIDCVVR  RGNLANVIRYFPTQALNFA  ALNFAFKDKYKQIFLGGVD  VDKRTQFWLYFAGNLASGG  AGATSLCFVYPLDFARTRL  GLGDCLVKIYKSDGIKGLY  YKSDGIKGLYQGFNVSVQG  NVSVQGIIIYRAAYFGIYD  QGIIIYRAAYFGIYDTAKG  VTAVAGLTSYPFDTVRRRM  SGRKGTDIMYTGTLDCWRK  MGGAFVLVLYDEIKKYTXX  LVLYDEIKKYTXXXXXXXX  SQRMVVMHGYKAVKEALLD  KAVKEALLDYKDEFSGRGD  RFSLTTLRNYGMGKQGNES  DILFRKHFDYNDEKFLRLM  NDEKFLRLMYLFNENFHLL  LLSTPWLQLYNNFPSFLHY  YNNFPSFLHYLPGSHRKVI  IKNVAEVKEYVSERVKEHH  KEKHSAERLYTMDGITVTV  TETTSTTLRYGLLILMKYP  AIKDRQEMPYMDAVVHEIQ  VVPTLDSVLYDNQEFPDPE  ENGKFKYSDYFKPFSTGKR  IGFGCIPPRYKLCVIPRSX  EKFNLPPGNYKKPKLLYCS  YIKSTETGQYLAMDTDGLL  LAMDTDGLLYGSQTPNEEC  PTAVHPVRDYAAQTSPSPK  ALTGLTVAEYFRDQEGQDV  GSITSVQAIYVPADDLTDP  RGVQKILQDYKSLQDIIAI  GFQQILAGEYDHLPEQAFY  DMSPLRPQNYLFGCELKAD  GCELKADKDYHFKVDNDEN  PKVEAKFINYVKNCFRMTD  KFIIPQIVKYSPDCIIIVV  VSNPVDILTYVTWKLSGLP  CNLDSARFRYLMAEKLGIH  AYEVIKLKGYTNWAIGLSV  PVSTMVKGMYGIENEVFLS  EGNTLISVDYEIFGKVQGV  LIAKGPVSKYSQAVPAVTE  GPIPEVLKNYMDAQYYGEI  VLKNYMDAQYYGEIGIGTP  LKNYMDAQYYGEIGIGTPP  DIACWIHHKYNSDKSSTYV  KYNSDKSSTYVKNGTSFDI  KNGTSFDIHYGSGSLSGYL  HYGSGSLSGYLSQDTVSVP  KFDGILGMAYPRISVNNVL  LMLGGTDSKYYKGSLSYLN  MLGGTDSKYYKGSLSYLNV  SKYYKGSLSYLNVTRKAYW  SYLNVTRKAYWQVHLDQVE  GAVPLIQGEYMIPCEKVST  ITLKLGGKGYKLSPEDYTL  KGYKLSPEDYTLKVSQAGK  ILGDVFIGRYYTVFDRDNN  LGDVFIGRYYTVFDRDNNR  GDHSTPPSAYGSVKAYTNF  AQRQDIAFAYQRRTKKELA  LGLLKTPAQYDASELKASM  QELQEINRVYKEMYKTDLE  EINRVYKEMYKTDLEKDII  RAEDGSVIDYELIDQDARD  LIDQDARDLYDAGVKRKGT  PHLQKVFDRYKSYSPYDML  QKVFDRYKSYSPYDMLESI  FDRYKSYSPYDMLESIRKE  VQCIQNKPLYFADRLYDSM  KPLYFADRLYDSMKGKGTR  KIRSEFKRKYGKSLYYYIQ  FKRKYGKSLYYYIQQDTKG  KRKYGKSLYYYIQQDTKGD  RKYGKSLYYYIQQDTKGDY  YIQQDTKGDYQKALLYLCG  KGDYQKALLYLCGGDDXXX  XXXMAGWNAYIDNLMADGT  TCQDAAIVGYKDSPSVWAA  MASHLRRSQYXXXXXXXXX  LMSLIINTFYSNKEIFLRE  SSDALDKIRYESLTDPSKL  MIGQFGVGFYSAYLVAEKV  QFGVGFYSAYLVAEKVTVI  ITKHNDDEQYAWESSAGGS  KKKKKIKEKYIDQEELNKT  NPDDITNEEYGEFYKSLTN  RKKKNNIKLYVRRVFIMDN  DNCEELIPEYLNFIRGVVD  TELAEDKENYKKFYEQFSK  EDKENYKKFYEQFSKNIKL  RKKLSELLRYYTSASGDEM  KKLSELLRYYTSASGDEMV  GDEMVSLKDYCTRMKENQK  RMKENQKHIYYITGETKDQ  MKENQKHIYYITGETKDQV  LRKHGLEVIYMIEPIDEYC  IYMIEPIDEYCVQQLKEFE  SPCCIVTSTYGWTANMERI  SVKDLVILLYETALLSSGF  DPQTHANRIYRMIKLGLGI  SDVEAIFSKYGKIVGCSVH  VHKGFAFVQYVNERNARAA  GVKRSAAEMYGSVTEHPSP  LLSSSFDLDYDFQRDYYDR  DLDYDFQRDYYDRMYSYPA  LDYDFQRDYYDRMYSYPAR  FQRDYYDRMYSYPARVPPP  XXXXXXXXMYRALRLLARS  GELKVPNDKYYGAQTVRST  ELKVPNDKYYGAQTVRSTM  RAAAEVNQDYGLDPKIANA  LTLGQEFSGYVQQVKYAMT  FSGYVQQVKYAMTRIKAAM  VTALNPHIGYDKAAKIAKT  LKETAIELGYLTAEQFDEW  MAKPAQGAKYRGSIHDFPG  DPNQDAEALYTAMKGFGSD  RQRQEVCQSYKSLYGKDLI  EVCQSYKSLYGKDLIADLK  GKDLIADLKYELTGKFERL  IVGLMRPPAYCDAKEIKDA  EQMHQLVAAYKDAYERDLE  QLVAAYKDAYERDLEADII  LVQQDVQDLYEAGELKWGT  WGTDEAQFIYILGNRSKQH  VKCIRSTPEYFAERLFKAM  DIREIFRTKYEKSLYSMIK  FRTKYEKSLYSMIKNDTSG  MIKNDTSGEYKKTLLKLSG  FFPEAAQVAYQMWELSAVA  LGLMMPPAHYDAKQLKKAM  AEIRAINEAYKEDYHKSLE  AINEAYKEDYHKSLEDALS  FMTILCTRSYPHLRRVFQE  FQEFIKMTNYDVEHTIKKE  KPLFFADKLYKSMKGAGTD  NIRREFIEKYDKSLHQAIE  RDCSGGCQCYPEKGGRGQP  QPGPVGPQGYNGPPGLQGF  QGGPRGRPGYDGCNGTQGD  GPKGQKGEPYALPKEERDR  ALPKEERDRYRGEPGEPGL  QQGNRGLGFYGVKGEKGDV  TGVTFHPDQYKGEKGSEGE  IMGFPGLRGYPGLSGEKGS  DPGPPGLPAYSPHPSLAKG  IGDPGIPALYGGPPGPDGK  IKGPPGDPGYPGIPGTKGT  PGPSGQPGLYGPPGLHGFP  DEGPMGLKGYLGAKGIQGM  APGIFGLKGYRGPPGPPGS  MPGRSVSIGYLLVKHSQTD  VGMNKLWSGYSLLYFEGQE  KLWSGYSLLYFEGQEKAHN  ARFSTMPFLYCNPGDVCYY  LYCNPGDVCYYASRNDKSY  YCNPGDVCYYASRNDKSYW  YYASRNDKSYWLSTTAPLP  PVAEDEIKPYISRCSVCEA  AGWRSLWIGYSFLMHTAAG  CNGGRGTCHYYANKYSFWL  NGGRGTCHYYANKYSFWLT  GTCHYYANKYSFWLTTIPE  ISKNKDTGTYEDFVEGLRV  QEDSNGCINYEAFVKHIMS  SYVTTSTRTYSLGSALRPS  LRPSTSRSLYASSPGGVYA  LYASSPGGVYATRSSAVRL  QELNDRFANYIDKVRFLEQ  QGKSRLGDLYEEEMRELRR  KNLQEAEEWYKSKFADLSE  RQAKQESTEYRRQVQSLTC  ENFAVEAANYQDTIGRLQD  EEMARHLREYQDLLNVKMA  MALDIEIATYRKLLEGEES  XXXXXXMPPYTVVYFPVRG  XXMPPYTVVYFPVRGRCAA  KFQDGDLTLYQSNTILRHL  RHLGRTLGLYGKDQQEAAL  DGVEDLRCKYISLIYTNYE  LRCKYISLIYTNYEAGKDD  KYISLIYTNYEAGKDDYVK  TNYEAGKDDYVKALPGQLK  VGDQISFADYNLLDLLLIH  LDAFPLLSAYVGRLSARPK  LKAFLASPEYVNLPINGNG  ELQWEQAQDYLKRFYLYDS  QAQDYLKRFYLYDSETKNA  QDYLKRFYLYDSETKNANS  RCGVPDVAEYSLFPNSPKW  PKWTSKVVTYRIVSYTRDL  KVVTYRIVSYTRDLPHITV  GSSLGINFLYAATHELGHS  HSSDPNAVMYPTYGNGDPQ  DPNAVMYPTYGNGDPQNFK  DDIKGIQKLYGKRSNSRKK  KKPRGKMSSYAFFVQTCRE  DMAKADKARYEREMKTYIP  SAFFLFCSEYRPKIKGEHP  NTAADDKQPYEKKAAKLKE  KKAAKLKEKYEKDIAAYRA  EKYEKDIAAYRAKGKPDAA  SPQNISNCRYAQTPANMFY  YAQTPANMFYIVACDNRDQ  RDQRRDPPQYPVVPVHLDR  TPGGGHLNGYPVPPYAFFF  HLNGYPVPPYAFFFPPMLG  LQHQLPVSGYSTPSPATIE  PSPPPLPRIYKPCFVCQDK  FVCQDKSSGYHYGVSACEG  CQDKSSGYHYGVSACEGCK  RRSIQKNMVYTCHRDKNCI  NKVTRNRCQYCRLQKCFEV  VPKPECSESYTLTPEVGEL  LNCKMKFAVYLPPKAETGK  AETGKCPALYWLSGLTCTE  EQNFISKSGYHQSASEHGL  WDFGTGAGFYVDATEDPWK  ATEDPWKTNYRMYSYVTEE  DPWKTNYRMYSYVTEELPQ  WKTNYRMYSYVTEELPQLI  ICALKNPGKYKSVSAFAPI  PWGKKAFSGYLGTDQSKWK  PVVFRLQEGYDHSYYFIAT  RLQEGYDHSYYFIATFITD  LQEGYDHSYYFIATFITDH  TDHIRHHAKYLNAXXXXXX  VLAPHLTRAYAKDVKFGAD  AKSIDLKDKYKNIGAKLVQ  IEGMKFDRGYISPYFINTS  KFDRGYISPYFINTSKGQK  EQLDVTTSEYEKEKLNERL  AVGIDLGTTYSCVGVFQHG  AGRPKVQVEYKGETKSFYP  QASIEIDSLYEGIDFYTSI  DSLYEGIDFYTSITRARFE  TKQTQTFTTYSDNQPGVLI  NQPGVLIQVYEGERAMTKD  ERMVQEAEKYKAEDEKQRD  VSSKNSLESYAFNMKATVE  VCNPIITKLYQSAGGMPGG  SGDLAKKKIYPTIWWLFRD  LPENTFIVGYARSRLTVAD  LEDFFARNSYVAGQYDDAA  ARNSYVAGQYDDAASYQRL  AGQYDDAASYQRLNSHMNA  LGSQANRLFYLALPPTVYE  FYLALPPTVYEAVTKNIHE  SSLFREDQIYRIDHYLGKE  EDQIYRIDHYLGKEMVQNL  PFGTEGRGGYFDEFGIIRD  QANNVVLGQYVGNPDGEGE  DGEGEATKGYLDDPTVPRG  TATFAAVVLYVENERWDGV  PEESELDLTYGNRYKNVKL  ELDLTYGNRYKNVKLPDAY  YKNVKLPDAYERLILDVFC  ELEKPKPIPYIYGSRGPTE  EKPKPIPYIYGSRGPTEAD  ELMKRVGFQYEGTYKWVNP  RVGFQYEGTYKWVNPHKLX  TTTESEVMKYITSLNEDST  AIVIDCGINYVPDDKKPNG  FKPGKWMIQYNNLNLKTPV  GLLSEEVELYGETKAKVLL  RLKHRPDGKYVVVTGITPT  LVQALGAHLYQNVFACVRQ  IKGGAAGGGYSQVIPMEEF  EKFFNIKCRYSGLCPHVVV  TAGLPLPKAYIQENLELVE  QAPSSFQLLYDLKLPVEDK  KIRIIAQKIYGADDIELLP  PEAQHKAEVYTKQGFGNLP  RASVGAGFLYPLVGTMSTM  PGLPTRPCFYDIDLDPETE  EAKLLVDAAYNWTQKSIKQ  SASPMDLLSYFKQPVAATR  TRTVVRAADYMHVALGLLE  DQAERCSDKYRTITGRCNN  ALARWLPAEYEDGLSLPFG  TSFVDASMVYGSEVSLSLR  SLRLRNRTNYLGLLAINQR  NPRWNGDKLYNEARKIMGA  MGAMVQIITYRDFLPLVLG  ARARRTLGHYRGYCSNVDP  RRTLGHYRGYCSNVDPRVA  AFFASWRIVYEGGIDPILR  RSRDHGLPGYNAWRRFCGL  DLARKFLNLYGTPDNIDIW  SRDIFRANIYPRGFVNCSR  APNQEKVSDYEMKLMDLDV  EQLGIPEQEYSCVVKMPSG  PVQLTFALRYLNFFTKATP  SADVPLVVEYKIADMGHLK  KIADMGHLKYYLAPKIEDE  IADMGHLKYYLAPKIEDEE  GAQNISNCTYADRPGRRFY  YADRPGRRFYVVACDNRDP  NRDPRDSPRYPVVPVHLDT  DNPLSGGDQYQNITVHRHL  EGEDGLDEIYSFSESLRKL  SNLSIRIAAYKSILQERVK  KEDIIQGFRYGSDIVPFSK  SKVDEEQMKYKSEGKCFSV  DLDMVAIVRYAYDKRANPQ  DMVAIVRYAYDKRANPQVG  VAFPHIKHNYECLVYVQLP  IKHNYECLVYVQLPFMEDL  LPFMEDLRQYMFSSLKNSK  FSSLKNSKKYAPTEAQLNA  QFLDTNETPYFMKSIDCIR  LSMDEKVELYRIKFKESFA  ALVIMWQKHYVYGPLPQSF  VIMWQKHYVYGPLPQSFDK  IQGLASKWDYEKNEWKKXX  IKSTAISLFYELSENDLNF  ELQLEPEELYQTFQRIVEN  ENVNVIISTYGEGESGPMG  FTLKQFAEMYVAKFAAKGE  MMKKLWGDRYFDPANGKFS  LPSPVTAQKYRCELLYEGP  AQKYRCELLYEGPPDDEAA  CDPKGPLMMYISKMVPTSD  VPTSDKGRFYAFGRVFSGL  QRTILMMGRYVEPIEDVPC  IKKSDPVVSYRETVSEESN  KSPNKHNRLYMKARPFPDG  RQELKQRARYLAEKYEWDV  QRARYLAEKYEWDVAEARK  IIPTARRCLYASVLTAQPR  AQPRLMEPIYLVEIQCPEQ  CPEQVVGGIYGVLNRKRGH  GTPMFVVKAYLPVNESFGF  AKVDTDGNGYISFNELNDL  KAACLPLPGYRVREITENL  QSSVGTQHSYSEEEKYAFV  QHSYSEEEKYAFVNWINKA  AEDLKEGKPYLVLGLLWQV  EELLLRWANYHLENAGCNK  STDIKDSKAYYHLLEQVAP  TDIKDSKAYYHLLEQVAPK  AFIANLFNRYPALHKPENQ  GVNPRVNHLYSDLSDALVI  SDALVIFQLYEKIKVPVDW  DWNRVNKPPYPKLGGNMKK  NMKKLENCNYAVELGKNQA  ALIWQLMRRYTLNILEEIG  DAIQPGSINYDLLKTENLN  DDEKLNNAKYAISMARKIG  LNFSHGTHEYHAETIKNVR  CDENILWLDYKNICKVVEV  KVVEVGSKIYVDDGLISLQ  LSGETAKGDYPLEAVRMQH  IAREAEAAIYHLQLFEELR  GRSAHQVARYRPRAPIIAV  PQTARQAHLYRGIFPVLCK  MMKLIINSLYKNKEIFLRE  LIGQFGVGFYSAFLVADKV  LVLKEEASDYLELDTIKNL  DTIKNLVKKYSQFINFPIY  YSQFINFPIYVWSSKTETV  PSKEVEEDEYKAFYKSFSK  VEEDEYKAFYKSFSKESDD  SKESDDPMAYIHFTAEGEV  SAPRGLFDEYGSKKSDYIK  DEYGSKKSDYIKLYVRRVF  SKKSDYIKLYVRRVFITDD  DDFHDMMPKYLNFVKGVVD  MIKKIADDKYNDTFWKEFG  PTDITSLDQYVERMKEKQD  RMKEKQDKIYFMAGSSRKE  FVERLLKKGYEVIYLTEPV  LLKKGYEVIYLTEPVDEYC  IYLTEPVDEYCIQALPEFD  SPCALVASQYGWSGNMERI  MERIMKAQAYQTGKDISTN  TGKDISTNYYASQKKTFEI  FETATLRSGYLLPDTKAYG  GYLLPDTKAYGDRIERMLR  VAKNRGQGTYEDYLEGFRV  NRGQGTYEDYLEGFRVFDK  HEDSNGCINYEAFLKHILS  NPQENLPDFYNDWMFIAKH  LVLGCITMAYVWGKGHGDV  VLPRNIAVPYCQLSKKLEL  KLELPPILVYADCVLANWK  KKDPNKPLTYENMDVLFSF  KAFFSVLRIYLSGWKGNPQ  NPQLSDGLVYEGFWEDPKE  AQFLQDMRRYMPPAHRNFL  KGDAGLREAYDACVKALVS  KTIGLREVWYFGLHYVDNK  REVWYFGLHYVDNKGFPTW  LQFKFRAKFYPEDVAEELI  KEGILSDEIYCPPETAVLL  PETAVLLGSYAVQAKFGDY  YAVQAKFGDYNKEVHKSGY  YNKEVHKSGYLSSERLIPQ  MLKDNAMLEYLKIAQDLEM  LKIAQDLEMYGINYFEIKN  QDLEMYGINYFEIKNKKGT  GVDALGLNIYEKDDKLTPK  DKKAPDFVFYAPRLRINKR  QLCMGNHELYMRRRKPDTI  EELMLRLQDYEEKTKKAER  APPPPPPPVYEPVSYHVQE  PPPVYEPVSYHVQESLQDE  QDEGAEPTGYSAELSSEGI  ENMRQGRDKYKTLRQIRQG  MKIKSLEEIYLFSLPIKES  FKAFVAIGDYNGHVGLGVK  LSIVPVRRGYWGNKIGKPH  LMMAGIDDCYTSARGCTAT  ATFDAISKTYSYLTPDLWK  FDAISKTYSYLTPDLWKET  VKPQHQMLFYKDDVLFYNI  LFYKDDVLFYNISSMKSTE  ISSMKSTESYFIPEVRIYD  SYFIPEVRIYDSGTYKCTV  EVRIYDSGTYKCTVIVNNK  NNKEKTTAEYQLLVEGVPS  HNRHGNKAVYSVMAMVEHS  MAMVEHSGNYTCKVESSRI  IASKSDSGTYICTAGIDKV  EMLSQPRISYDAQFEVIKG  SISGTLPISYQLLKTSKVL  KDNPTEDVEYQCVADNCHS  VNEGSGPITYKFYREKEGK  GSGPITYKFYREKEGKPFY  YREKEGKPFYQMTSNATQA  KASKEQEGEYYCTAFNRAN  ASKEQEGEYYCTAFNRANH  ALLIIAAKCYFLRKAKAKQ  DPNMEANSHYGHNDDVRNH  KEPLNSDVQYTEVQVSSAE  AVPDAVESRYSRTEGSLDG  XXXXXXMAAYKLVLIRHGE  NLENRFSGWYDADLSPAGH  AQVKIWRRSYDVPPPPMEP  PPMEPDHPFYSNISKDRRY  NLPTGIPIVYELDKNLKPI  RTLLAKNLPYKVTQDELKE  KDGKSKGIAYIEFKTEADA  EIDGRSISLYYTGEKGQNQ  IDGRSISLYYTGEKGQNQD  TGEKGQNQDYRGGKNSTWS  KTLVLSNLSYSATEETLQE  TEDTIFLREYQTRQNQCFY  YQTRQNQCFYNSSYLNVQR  TTKEQLGEFYEALDCLCIP  EVNSLRSREYWDYEAHVPS  SLRSREYWDYEAHVPSWGN  VPSWGNQDDYQLVRKLGRG  LVRKLGRGKYSEVFEAINI  SKTPALVFEYINNTDFKQL  INNTDFKQLYQILTDFDIR  ILTDFDIRFYMYELLKALD  TDFDIRFYMYELLKALDYC  MYELLKALDYCHSKGIMHR  LIDWGLAEFYHPAQEYNVR  AEFYHPAQEYNVRVASRYF  EYNVRVASRYFKGPELLVD  FKGPELLVDYQMYDYSLDM  PELLVDYQMYDYSLDMWSL  LLVDYQMYDYSLDMWSLGC  EPFFHGQDNYDQLVRIAKV  AKVLGTEELYGYLKKYHID  VLGTEELYGYLKKYHIDLD  EELYGYLKKYHIDLDPHFN  LDLLDKLLRYDHQQRLTAK  TAKEAMEHPYFYPVVKEQS  KEAMEHPYFYPVVKEQSQP  XXMLSSTAMYSAPGRDLGM  GPLQLRFSPYVFNGGTILA  IHTRDSPKCYKLTDKTVIG  KIIEARLKMYKHSNNKAMT  IAAMLSTILYSRRFFPYYV  ILYSRRFFPYYVYNIIGGL  LYSRRFFPYYVYNIIGGLD  SRRFFPYYVYNIIGGLDEE  LDEEGKGAVYSFDPVGSYQ  FISAAERDVYTGDALRICI  RELNDRLAVYIDKVRSLET  NGAQIKLREYEAALNSKDA  EDLEFRKSMYEEEINETRR  EVDSGRQIEYEYKLAQALH  DSGRQIEYEYKLAQALHEM  EQHDAQVRLYKEELEQTYH  LYKEELEQTYHAKLENARL  DQMQQQLNDYEQLLDVKLA  LALDMEISAYRKLLEGEEE  IGDTSVSYKYTSRYVLKAG  SVSYKYTSRYVLKAGQTVT  KSFLRFREKYGDVFTVHLG  FELFSGFLKYFPGAHRQVY  TETTSTTLRYGFLLMLKYP  RYGFLLMLKYPHVAERVYR  KYPHVAERVYREIEQVIGP  LSTALHDPHYFEKPDAFNP  GLIWTLILHYSISMPMWDE  NVDEHSVMTYLSQFPKAKL  KLNPKKARAYGPGIEPTGN  SAGQGEVLVYVEDPAGHQE  DKNRTFSVWYVPEVTGTHK  SGNIANKTTYFEIFTAGAG  QLEARGDSTYRCSYQPTME  RGDSTYRCSYQPTMEGVHT  AGVPIPRSPYTVTVGQACN  VKETADFKVYTKGAGSGEL  KQKDLGDGVYGFEYYPMVP  LGDGVYGFEYYPMVPGTYI  GDGVYGFEYYPMVPGTYIV  EYYPMVPGTYIVTITWGGQ  KGDGSCDVRYWPQEAGEYA  RYWPQEAGEYAVHVLCNSE  LVKDNGNGTYSCSYVPRKP  NGNGTYSCSYVPRKPVKHT  GSHPNKVKVYGPGVAKTGL  TGLKAHEPTYFTVDCAEAG  NDNDTFTVKYTPRGAGSYT  KYTPRGAGSYTIMVLFADQ  DIIDHHDNTYTVKYTPVQQ  HHDNTYTVKYTPVQQGPVG  QGPVGVNVTYGGDPIPKSP  RFLPREEGPYEVEVTYDGV  EGPYEVEVTYDGVPVPGSP  NGDGTCSVSYVPTEPGDYN  SYVPTEPGDYNINILFADT  SEAGLPAEVYIQDHGDGTH  HGDGTHTITYIPLCPGAYT  TYIPLCPGAYTVTIKYGGQ  PGAYTVTIKYGGQPVPNFP  AVDTSGVQCYGPGIEGQGV  YVQDRGDGMYKVEYTPYEE  RGDGMYKVEYTPYEEGLHS  GMYKVEYTPYEEGLHSVDV  EGLHSVDVTYDGSPVPSSP  NKDGSCSVEYIPYEAGTYS  GSCSVEYIPYEAGTYSLNV  EYIPYEAGTYSLNVTYGGH  AGTYSLNVTYGGHQVPGSP  NADGTQTVNYVPSREGPYS  NYVPSREGPYSISVLYGDE  EGPYSISVLYGDEEVPRSP  HIQDNHDGTYTVAYVPDVT  NHDGTYTVAYVPDVTGRYT  AYVPDVTGRYTILIKYGGD  TGRYTILIKYGGDEIPFSP  GGDEIPFSPYRVRAVPTGD  NEDGTFDIFYTAPQPGKYV  FYTAPQPGKYVICVRFGGE  LRSQQLAPQYTYAQGGQQT  SQQLAPQYTYAQGGQQTWA  NKDGTVTVRYAPSEAGLHE  AGLHEMDIRYDNMHIPGSP  HIPGSPLQFYVDYVNCGHV  GSPLQFYVDYVNCGHVTAY  YVNCGHVTAYGPGLTHGVV  NQDGTCSVSYLPVLPGDYS  SYLPVLPGDYSILVKYNEQ  PGDYSILVKYNEQHVPGSP  FIIDTRDAGYGGLSLSIEG  LEDGTCRVTYCPTEPGNYI  TYCPTEPGNYIINIKFADQ  EIVEGENHTYCIRFVPAEM  MGTHTVSVKYKGQHVPGSP  RKDGSCGVAYVVQEPGDYE  AYVVQEPGDYEVSVKFNEE  SPSGALEECYVTEIDQDKY  YVTEIDQDKYAVRFIPREN  RFIPRENGVYLIDVKFNGT  GGDPGLVSAYGAGLEGGVT  MDCQECPEGYRVTYTPMAP  ECPEGYRVTYTPMAPGSYL  TYTPMAPGSYLISIKYGGP  PGSYLISIKYGGPYHIGGS  LISIKYGGPYHIGGSPFKA  AKGLGLSKAYVGQKSSFTV  LVKHVGSRLYSVSYLLKDK  VGSRLYSVSYLLKDKGEYT  SYLLKDKGEYTLVVKWGDE  GDEHIPGSPYRVVVPXXXX  GPNTGGMGAYCPAPQVSND  DGMQQEGTPYTGILYAGIM  EGTPYTGILYAGIMLTKNG  ILPLLKSDLYEVIQSTLDG  LTVVMASKGYPGDYTKGVE  MASKGYPGDYTKGVEITGF  FLQQPRSLTYKESGVDIAA  GAEPLFFLDYFSCGKLDLS  GETAEMPDMYPPGEYDLAG  MPDMYPPGEYDLAGFAVGA  KIVAKSSLQYSSPAPDGCG  DLLLTPTRIYSHSLLPVLR  PTRVINHKLYKNRVEFDSA  EVLNIGKKLYEGKTKEVYE  KRNPGVKEGYKFYPPKVEL  PGVKEGYKFYPPKVELFFK  RSQQKDKQSYRDLKEVTPE  DETLRIKAEYEGDGIPTVF  GPVMSGNTAYPVISCPPLT  AAALRTGDDYIAIGADEEE  LGSQIEEAIYQEIRNTDMK  QEIRNTDMKYKNRVRSRIS  RGGRQHHPPYHQQHHQGPP  LLRRPGEKTYTQRCRLFVG  DEFKRLFAKYGEPGEVFIN  ALSVRNLSPYVSNELLEEA  EKLAQKNPMYQKERETPPR  RFAQHGTFEYEYSQRWKSL  AQHGTFEYEYSQRWKSLDE  KLESEMEDAYHEHQANLLR  QMRRQREESYSRMGYMDPR  REESYSRMGYMDPRERDMR  GGAMNMGDPYGSGGQKFPP  PLGGGGGIGYEANPGVPPA  AGYGRGREEYEGPNKKPRF  KMLPDKDCRYALYDATYET  PDKDCRYALYDATYETKES  CRYALYDATYETKESKKED  SAPLKSKMIYASSKDAIKK  IQCQAIREAYLTQLMIIKN  QLMIIKNSTYREIGRRLKF  HMDANKAGRYQCQYRIGHY  NKAGRYQCQYRIGHYRFRY  YQCQYRIGHYRFRYSDTLE  YRIGHYRFRYSDTLELVVT  TLELVVTGLYGKPFLSADR  PVDLNVSGIYRCYGWYNRS  LNVSGIYRCYGWYNRSPYL  SGIYRCYGWYNRSPYLWSF  CYGWYNRSPYLWSFPSNAL  RVDSAATSGYEIGNPPDYR  GYEIGNPPDYRGQSCMKRH  KAKIELLGSYDPQKQLIIE  QKQLIIEDPYYGNDSDFET  KQLIIEDPYYGNDSDFETV  GNDSDFETVYQQCVRCCRA  VELREAQRDYLDFLDDEED  LDDEEDQGIYQSKVRELIS  VRELISDNQYRLIVNVNDL  DFVASIDATYAKQYEEFYV  SIDATYAKQYEEFYVGLEG  TYAKQYEEFYVGLEGSFGS  ATKKTIERRYSDLTTLVAF  LVAFPSSSVYPTKDEENNP  EENNPLETEYGLSVYKDHQ  LETEYGLSVYKDHQTITIQ  GDRVQVVGTYRCLPGKKGG  RCLPGKKGGYTSGTFRTVL  LAPSIHGHDYVKKAILCLL  SVAKSQLLRYVLCTAPRAI  AAANPVYGRYDQYKTPMEN  NPVYGRYDQYKTPMENIGL  SDHVLRMHRYRAPGEQDGD  QEDQQDTQIYEKHDNLLHG  MVSAAFMKKYIHVAKIIKP  PVLTQESATYIAEEYSRLR  ESATYIAEEYSRLRSQDSM  AEEAVELVQYAYFKKVLEK  EAVELVQYAYFKKVLEKEK  QPDAKDGDSYDPYDFSDTE  AKDGDSYDPYDFSDTEEEM  DGSDEKKKLYCIYVAIGQK  DEKKKLYCIYVAIGQKRST  RLTDADAMKYTIVVSATAS  TASDAAPLQYLAPYSGCSM  AAPLQYLAPYSGCSMGEYF  PYSGCSMGEYFRDNGKHAL  DNGKHALIIYDDLSKQAVA  DDLSKQAVAYRQMSLLLRR  REAYPGDVFYLHSRLLERA  ETQAGDVSAYIPTNVISIT  QIFLETELFYKGIRPAINV  LTELLKQGQYSPMAIEEQV  AIEEQVAVIYAGVRGYLDK  AVIYAGVRGYLDKLEPSKI  GLDSMKDEEYEQMVKELQE  CLHLASIHGYLGIVELLVS  CGADVNRVTYQGYSPYQLT  DVNRVTYQGYSPYQLTWGR  RVTYQGYSPYQLTWGRPST  LPESEDEESYDTESEFTEF  REVWFFGLQYQDTKGFSTW  KEGILNDDIYCPPETAVLL  PETAVLLASYAVQSKYGDF  LASYAVQSKYGDFNKEVHK  FNKEVHKSGYLAGDKLLPQ  MLREDAVLEYLKIAQDLEM  LKIAQDLEMYGVNYFSIKN  QDLEMYGVNYFSIKNKKGS  GVDALGLNIYEQNDRLTPK  DKKAPDFVFYAPRLRINKR  ALCMGNHELYMRRRKPDTI  ENMRLGRDKYKTLRQIRQG  EEAANTMVNYYTSVTPVLR  EAANTMVNYYTSVTPVLRG  TPVLRGQPIYIQFSNHKEL  LRIIVENLFYPVTLDVLHQ  NNQFQALLQYADPVSAQHA  SKLTSLNVKYNNDKSRDYT  KYNNDKSRDYTRPDLPSGD  QSLFILFGVYGDVQRVKIL  RAELNPWPEYIYTRLEMYN  ELNPWPEYIYTRLEMYNIL  EYIYTRLEMYNILKAEHDS  DAESWKTTPYQIACGISQG  FEDEEAQAVYWHSSAHIMG  IMGEAMERVYGGCLCYGPP  ERVYGGCLCYGPPIENGFY  YGPPIENGFYYDMYLEEGG  GPPIENGFYYDMYLEEGGV  IENGFYYDMYLEEGGVSSN  KETLLAMFKYNKFKCRILN  LKIHKNSSTYWEGKADMET  RKIGRDQELYFFHELSPGS  SCFFLPKGAYIYNALIEFI  FFLPKGAYIYNALIEFIRS  ALIEFIRSEYRKRGFQEVV  WMTSGHWQHYSENMFSFEV  GCLDFLRTVYSVFGFSFKL  ELNSGDGAFYGPKIDIQIK  IQIKDAIGRYHQCATIQLD  QLPIRFNLTYVSHDGDDKK  RMIAILTENYGGKWPFWLS  VPVGPTCDEYAQKVRQQFH  KIRNAQLAQYNFILVVGEK  GQPFDVGPRYTQLQYIGEG  VGPRYTQLQYIGEGAYGMV  QLQYIGEGAYGMVSSAYDH  GAYGMVSSAYDHVRKTRVA  KISPFEHQTYCQRTLREIQ  STLEAMRDVYIVQDLMETD  VQDLMETDLYKLLKSQQLS  QQLSNDHICYFLYQILRGL  SNDHICYFLYQILRGLKYI  HDHTGFLTEYVATRWYRAP  LTEYVATRWYRAPEIMLNS  PEIMLNSKGYTKSIDIWSV  NRPIFPGKHYLDQLNHILG  CIINMKARNYLQSLPSKTK  TVEEALAHPYLEQYYDPTD  ALAHPYLEQYYDPTDEPVA  LAHPYLEQYYDPTDEPVAE  GLAVAEPAVYFKEQFLDGD  KFVLSSGKFYGDEEKDKGL  LQTSQDARFYALSASFEPF  EQNIDCGGGYVKLFPNSLD  QTDMHGDSEYNIMFGPDIC  TKKVHVIFNYKGKNVLINK  CKDDEFTHLYTLIVRPDNT  TLIVRPDNTYEVKIDNSQV  EPPVIQNPEYKGEWKPRQI  IHPEIDNPEYSPDPSIYAY  PEYSPDPSIYAYDNFGVLG  YSPDPSIYAYDNFGVLGLD  NFLITNDEAYAEEFGNETW  VGETVGKTDYIPLLDVDEK  TEFLEEKMAYQEYPNSQNW  LEEKMAYQEYPNSQNWPED  PIQTDPFKMYHDDDLADLV  GQNDPLKDSYGMSPCNTAV  APPSSPKVTYKAPVPTGEV  DDTDDEIAKYDGKWEVEEM  FDTKPLIVQYEVNFQNGIE  QNGIECGGAYVKLLSKTPE  LDQFHDKTPYTIMFGPDKC  FGPDKCGEDYKLHFIFRHK  RHKNPKTGIYEEKHAKRPD  KRPDADLKTYFTDKKTHLY  YFTDKKTHLYTLILNPDNS  EGWLDDEPEYVPDPDAEKP  QRPVIDNPNYKGKWKPPMI  KPPMIDNPSYQGIWKPRKI  EERPWLWVVYILTVALPVF  GKKQTSGMEYKKTDAPQPD  TQAVADAVTYQLGFHSIEL  AASLFKEMCYRYREDLMAG  SLFKEMCYRYREDLMAGII  WDPQEGGQVYSVPMGGMMV  FAIGGSGSSYIYGYVDATY  IGGSGSSYIYGYVDATYRE  GSGSSYIYGYVDATYREGM  YIYGYVDATYREGMTKEEC  LGPPTTPGPYRSIYFSSPK  TTPGPYRSIYFSSPKGHLT  RGRIVETEAYLGPEDEAAH  GMFMKPGTLYVYIIYGMYF  FMKPGTLYVYIIYGMYFCM  LYVYIIYGMYFCMNISSQG  EWARKPLRFYVRGSPWVSV  SQARDFINQYYSSIKRSGS  QARDFINQYYSSIKRSGSQ  FTYICNHIKYATNRGNLRS  RIWNSQLVRYAGYRQQDGS  NSQLVRYAGYRQQDGSVRG  PVFHQEMVNYFLSPAFRYQ  NYFLSPAFRYQPDPWKGSA  AALMEMSGPYNSSPRPEQH  PKRSWKRQRYRLSAQAEGL  EALSQDPRRYEEWKWFRCP  CRCSQLDHLYRDEVQNAQQ  EKIWFDKFKYDDAERRFYE  QLREERLRQYAEKKAKKPA  GASKLVPVGYGIRKLQIQC  QPQHPLHVTYAGAAVDELG  DAPSRKDPKYREWHHFLVV  PPKGTGLHRYVWLVYEQDR  GLHRYVWLVYEQDRPLKCD  FKVASFRKKYELRAPVAGT  LRAPVAGTCYQAEWDDYVP  TCYQAEWDDYVPKLYEQLS  EWDDYVPKLYEQLSGKXXX  TANTNTCNKYGVSGYPTLK  FRDGEEAGAYDGPRTADGI  KAASNLRDNYRFAHTNVES  TNVESLVNEYDDNGEGIIL  NKFEDKTVAYTEQKMTSGK  IQGKDLLIAYYDVDYEKNA  QGKDLLIAYYDVDYEKNAK  LLIAYYDVDYEKNAKGSNY  YEKNAKGSNYWRNRVMMVA  KALERFLQDYFDGNLKRYL  DYFDGNLKRYLKSEPIPES  ENKDVLIEFYAPWCGHCKN  GHCKNLEPKYKELGEKLSK  ATANDVPSPYEVRGFPTIY  YEVRGFPTIYFSPANKKLN  ANKKLNPKKYEGGRELSDF  GRELSDFISYLQREATNPP  GNIDDALQCYSEAIKLDPH  KLDPHNHVLYSNRSAAYAK  VLYSNRSAAYAKKGDYQKA  SAAYAKKGDYQKAYEDGCK  AKKGDYQKAYEDGCKTVDL  DLKPDWGKGYSRKAAALEF  NRFEEAKRTYEEGLKHEAN  MNPFNMPNLYQKLESDPRT  TRTLLSDPTYRELIEQLRN  KEKELGNDAYKKKDFDTAL  KDFDTALKHYDKAKELDPT  KELDPTNMTYITNQAAVYF  TYITNQAAVYFEKGDYNKC  AAVYFEKGDYNKCRELCEK  EVGRENREDYRQIAKAYAR  EDYRQIAKAYARIGNSYFK  KAYARIGNSYFKEEKYKDA  GNSYFKEEKYKDAIHFYNK  EKYKDAIHFYNKSLAEHRT  GNECFQKGDYPQAMKHYTE  GDYPQAMKHYTEAIKRNPK  KRNPKDAKLYSNRAACYTK  KLYSNRAACYTKLLEFQLA  QLEPTFIKGYTRKAAALEA  AAALEAMKDYTKAMDVYQK  KDYTKAMDVYQKALDLDSS  SSCKEAADGYQRCMMAQYN  GYQRCMMAQYNRHDSPEDV  IKGVTLGFRYKMRSVYAHF  GFRYKMRSVYAHFPINVVI  EIRNFLGEKYIRRVRMRPG  XXXXMALKDYALEKEKVKK  DELGKKQFKYGNQLVRLAH  LAHREQVALYVDLDDVAED  DSICENARRYAKLFADAVQ  DAVQELLPQYKEREVVNKD  VVNKDVLDVYIEHRLMMEQ  GMVRSPQNQYPAELMRRFE  AELMRRFELYFQGPSSNKP  VKPKMVVATYTCDQCGAET  TCDQCGAETYQPIQSPTFM  QTNRSGGRLYLQTRGSRFI  VVQGLLSETYLEAHRIVKM  LRQIAEEDFYEKLAASIAP  LAASIAPEIYGHEDVKKAL  GVAKSQLLSYIDRLAPRSQ  IDRLAPRSQYTTGRGSSGV  SILAAANPAYGRYNPRRSL  AAANPAYGRYNPRRSLEQN  DLRLAQHITYVHQHSRQPP  PLDMKLMRRYIAMCREKQP  PMVPESLADYITAAYVEMR  SLADYITAAYVEMRREAWA  EAWASKDATYTSARTLLAI  AQFQAALDEYEELNVWQVN  GMVKPEPQIYKFLLDTLKA  CNPSDMSHGYVTVKPRVRL  LCHGFPESWYSWRYQIPAL  FPESWYSWRYQIPALAQAG  QIPALAQAGYRVLAMDMKG  RVLAMDMKGYGESSAPPEI  SSAPPEIEEYCMEVLCKEM  HDWGGMLVWYMALFYPERV  MLVWYMALFYPERVRAVAS  ANPVFDYQLYFQEPGVAEA  MVTEEEIQFYVQQFKKSGF  GIDLGFQSCYVAVARAGGI  GGIETIANEYSDRCTPACI  VEAEKSNLAYDIVQLPTGL  TGLTGIKVTYMEEERNFTT  DCVVSVPCFYTDAERRSVM  NETTAVALAYGIYKQDLPA  TAVALAYGIYKQDLPALEE  VFVDMGHSAYQVSVCAFNR  HFCEEFGKKYKLDIKSKIR  EFSITDVVPYPISLRWNSP  APFSKVLTFYRKEPFTLEA  RKEPFTLEAYYSSPQDLPY  KEPFTLEAYYSSPQDLPYP  YYSSPQDLPYPDPAIAQFS  QIDREMLNLYIENEGKMIM  NDAKNAVEEYVYEMRDKLS  AKNAVEEYVYEMRDKLSGE  EMRDKLSGEYEKFVSEDDR  KLEDTENWLYEDGEDQPKQ  DGEDQPKQVYVDKLAELKN  EELGKQIQQYMKIISSFKN  SSFKNKEDQYDHLDAADMT  XXXMAGWQSYVDNLMCDGC  CCQEAAIVGYCDAKYVWAA  AIVGYCDAKYVWAATAGGV  KCSVIRDSLYVDGDCTMDI  HGGGLNKKAYSMAKYLRDS  NKKAYSMAKYLRDSGFXXX  FLFKTKFHQYAMNGEKDIN  SPVTQDDLQYHNLSKQQNE  PQAIEFVNQYYGSFKEAKI  QAIEFVNQYYGSFKEAKIE  TKEIETTGTYQLTGDELIF  FEHICRHVRYSTNNGNIRS  RVWNAQLIRYAGYQMPDGS  NAQLIRYAGYQMPDGSIRG  WFRELELKWYALPAVANML  FPGCPFNGWYMGTEIGVRD  VRDFCDVQRYNILEEVGRR  SFMKYMQNEYRSRGGCPAD  PVFHQEMLNYVLSPFYYYQ  MLNYVLSPFYYYQVEAWKT  LNYVLSPFYYYQVEAWKTH  NYVLSPFYYYQVEAWKTHV  NPKVVCMDKYRLSCLEEER  LKELNNKFRYAVFGLGSSM  AVFGLGSSMYPRFCAFAHD  SNVTWDPHHYRLVQDSQPL  SCEDGQGLNYLPGEHLGVC  LEALDESGSYWVSDKRLPP  PCSLSQALTYFLDITTPPT  LEALCQPSEYSKWKFTNSP  QLPILKPRFYSISSSRDHT  IHLTVAVVTYHTRDGQGPL  CRRPDEDHIYQEEMLEMAQ  GVLHAVHTAYSRLPGKPKV  SRLPGKPKVYVQDILRQQL  VLHKEPGHLYVCGDVRMAR  KLNEEQVEDYFFQLKSQKR  FFQLKSQKRYHEDIFGAVF  EDIFGAVFPYEAKKDRVAV  LVLLTQPLGYLEAEMKTYS  GYLEAEMKTYSHRTMPSAC  SKAPGSAGHYELPWVEKYR  HYELPWVEKYRPVKLNEIV  QALRRTMEIYSKTTRFALA  IQSRCAVLRYTKLTDAQIL  NVIEKERVPYTDDGLEAII  CVNANIDEAYKILAHLWHL  ILAHLWHLGYSPEDIIGNI  VCKTFQMAEYLKLEFIKEI  LALAAAVRGYRCIIVMPEK  RNASNPLAHYDTTADEILQ  ELNQTEQTTYEVEGIGYDF  TTYEVEGIGYDFIPTVLDR  VILPDSVRNYMTKFLSDRW  PSDQVGKVIYKQFKQIRLT  ALVVHEQIQYHSTGKSSQR  TEPWMLASLYNGSVCVWNH  DDMQIRVFNYNTLERVHMF  VHMFEAHSDYIRCIAVHPT  SQVFEGHTHYVMQIVINPK  HEKGVNCIDYYSGGDKPYL  EKGVNCIDYYSGGDKPYLI  DYYSGGDKPYLISGADDRL  DDRLVKIWDYQNKTCVQTL  TVRIWHSSTYRLESTLNYG  TYRLESTLNYGMERVWCVA  RGSNNVALGYDEGSIIVKL  VKDMGSCEIYPQTIQHNPN  FVVVCGDGEYIIYTAMALR  VCGDGEYIIYTAMALRNKS  FAWAHDSSEYAIRESNSIV  KPDFGAESIYGGFLLGVRS  VRSVNGLAFYDWDNTELIR  TEESFFILKYLSEKVLAAQ  GLWVGDCFIYTSSVNRLNY  YTSSVNRLNYYVGGEIVTI  TSSVNRLNYYVGGEIVTIA  TIAHLDRTMYLLGYIPKDN  LDRTMYLLGYIPKDNRLYL  GYIPKDNRLYLGDKELNII  GDKELNIISYSLLVSVLEY  YSLLVSVLEYQTAVMRRDF  LQLGELKIAYQLAVEAESE  QECLHHAQDYGGLLLLATA  GKNNVAFMSYFLQGKVDAC  PEAAFLARTYLPSQVSRVV  HADLWPAKQYPLVTPNEER  XXXXMASNDYTQQATQSYG  DYTQQATQSYGAYPTQPGQ  QQATQSYGAYPTQPGQGYS  AYPTQPGQGYSQQSSQPYG  GYSQQSSQPYGQQSYSGYS  SSQPYGQQSYSGYSQSTDT  PYGQQSYSGYSQSTDTSGY  YSQSTDTSGYGQSSYSSYG  DTSGYGQSSYSSYGQSQNT  GYGQSSYSSYGQSQNTGYG  SYGQSQNTGYGTQSTPQGY  YGTQSTPQGYGSTGGYGSS  PQGYGSTGGYGSSQSSQSS  GSSQSSQSSYGQQSSYPGY  QSSYGQQSSYPGYGQQPAP  YGQQSSYPGYGQQPAPSST  PAPSSTSGSYGSSSQSSSY  YGSSSQSSSYGQPQSGSYS  SYGQPQSGSYSQQPSYGGQ  SGSYSQQPSYGGQQQSYGQ  PSYGGQQQSYGQQQSYNPP  QQSYGQQQSYNPPQGYGQQ  QQSYNPPQGYGQQNQYNSS  PQGYGQQNQYNSSSGGGGG  GGGGGGGGNYGQDQSSMSS  SSGGGSGGGYGNQDQSGGG  QSGGGGSGGYGQQDRGGRG  GGGGGGGGGYNRSSGGYEP  GGYNRSSGGYEPRGRGGGR  NVTIESVADYFKQIGIIKT  KTGQPMINLYTDRETGKLK  RGGPMGRGGYGGGGSGGGG  DDRRGGRGGYDRGGYRGRG  GRGGYDRGGYRGRGGDRGG  HRQDRRERPYXXXXXXXXX  EGDIALLKTYGQSTYSRQI  LLKTYGQSTYSRQIKQVED  INADSEDPKYIINVKQFAK  MRVGVDRNKYQIHIPLPPK  QVEEKPDVTYSDVGGCKEQ  VIGSELVQKYVGEGARMVR  EAVNKVIKSYAKFSATPRY  YAKFSATPRYMTYNXXXXX  FSATPRYMTYNXXXXXXXX  ACARPLISVYSEKGESSGK  NLRKNNRQPYAVSELAGHQ  RRVNTTQKRYAICSALAAS  LVVEDKVEGYKKTKEAVLL  IQRRGPCIIYNEDNGIIKA  SAFRKLDELYGTWRKAASL  LRIMLKLNPYAKTMRRNTI  XXXMAASTGYVRLWGAARC  RRKVRLAQPYVGVFLKRDD  DVVESLDEIYHTGTFAQIH  RDIIALNPLYRESVLQMMQ  GQRVVDNPIYLSDMGAALT  EETNIPKRLYKALSLLKKE  EKIKQTHRKYLLQEQLKII  SSEFNVTRNYLDWLTSIPW  AQAVLEEDHYGMEDVKKRI  STQGKILCFYGPPGVGKTS  SIARALNREYFRFSVGGMT  AEIKGHRRTYVGAMPGKII  DEVDKIGRGYQGDPSSALL  EQNANFLDHYLDVPVDLSK  RMEMINVSGYVAQEKLAIA  QEKLAIAERYLVPQARALC  DVLTLLIKQYCRESGVRNL  VEKVLRKSAYKIVSGEAES  KPVFTVERMYDVTPPGVVM  VMKESARIAYTFARAFLMQ  LMQHAPANDYLVTSHIHLH  LPAENKKDFYDLAAFITEG  GLEVHFVEHYREIFDIAFP  FTFRNSKQTYSGIPIIVAN  SMFTAIHKHYSLDDWKLFA  FICLDVANGYSEHFVEFVK  TTRTKTGVGYPQLSAVIEC  RNGRKLKLFYGMSSDTAMN  NKHAGGVAEYRASEGKTVE  SEGKTVEVPYKGDVENTIL  XXMANRGPAYGLSREVQQK  EVQQKIEKQYDADLEQILI  VLCELINALYPEGQAPVKK  SQFLQAAERYGINTTDIFQ  TGDFHAIDEYKPQDATTNP  ILAAAQMPAYQELVEEAIA  QELVEEAIAYGRKLGGSQE  ARARRLIELYKEAGISKDR  PGVKSVTKIYNYYKKFSYK  VKSVTKIYNYYKKFSYKTI  KSVTKIYNYYKKFSYKTIV  IYNYYKKFSYKTIVMGASF  KALEAVFGKYGRIVEVLLM  RGGHMDDGGYSMNFNMSSS  APVSRGRDSYGGPPRREPL  EPLPSRRDVYLSPRDDGYS  VYLSPRDDGYSTKDSYSSR  DDGYSTKDSYSSRDYPSSR  TKDSYSSRDYPSSRDTRDY  YPSSRDTRDYAPPPRDYTY  RDYAPPPRDYTYRDYGHSS  YAPPPRDYTYRDYGHSSSR  PPRDYTYRDYGHSSSRDDY  YGHSSSRDDYPSRGYSDRD  SRDDYPSRGYSDRDGYGRD  SRGYSDRDGYGRDRDYSDH  RDGYGRDRDYSDHPSGGSY  YSDHPSGGSYRDSYESYGN  PSGGSYRDSYESYGNSRSA  GSYRDSYESYGNSRSAPPT  PPSYGGSSRYDDYSSSRDG  YGGSSRYDDYSSSRDGYGG  DDYSSSRDGYGGSRDSYSS  DGYGGSRDSYSSSRSDLYS  SYSSSRSDLYSSGRDRVGR  GLPPSMERGYPPPRDSYSS  RGYPPPRDSYSSSSRGAPR  SDRGGGRSRYXXXXXXXXX  AFRLVSRRDYASEAIKGAV  AVTNPNNTFYATKRLIGRR  ATKRLIGRRYDDPEVQKDI  AWVEAHGKLYSPSQIGAFV  MKMKETAENYLGHTAKNAV  NEPTAAALAYGLDKSEDKV  KSEDKVIAVYDLGGGTFDI  SVQTDINLPYLTMDSSGPK  ENMVKNAEKYAEEDRRKKE  AGSATLSMAYAGARFVFSL  VKSQETECTYFSTPLLLGK  SKHKPKFTFYDGPPFATGL  PPFATGLPHYGHILAGTIK  GTIKDIVTRYAHQSGFHVD  WDCHGLPVEYEIDKTLGIR  DVAKMGITEYNNQCRAIVM  NQCRAIVMRYSAEWKSTVS  GRWIDFDNDYKTLYPQFME  DFDNDYKTLYPQFMESVWW  SVWWVFKQLYDKGLVYRGV  KQLYDKGLVYRGVKVMPFS  LSNFESHQNYKDVQDPSVF  AVCVNPEMQYVKIKDVARG  LMEARLSALYKLESDYEIL  SALYKLESDYEILERFPGA  EILERFPGAYLKGKKYRPL  PGAYLKGKKYRPLFDYFLK  GKKYRPLFDYFLKCKENGA  GAFTVLVDNYVKEEEGTGV  GTGVVHQAPYFGAEDYRVC  QAPYFGAEDYRVCMDFNII  TEVTDFAGQYVKDADKSII  LVATTFTHSYPFCWRSDTP  QLLRNNDLCYWVPELVREK  RDWTISRNRYWGTPIPLWV  CWFESGSMPYAQVHYPFEN  GSMPYAQVHYPFENKREFE  GIDQTRGWFYTLLVLATAL  QKMSKRKKNYPDPVSIIQK  PDPVSIIQKYGADALRLYL  KYGADALRLYLINSPVVRA  VLKDVLLPWYNAYRFLIQN  DVLLPWYNAYRFLIQNVLR  QKEEEIEFLYNENTVRESP  GFFETEMAAYRLYTVVPRL  ETEMAAYRLYTVVPRLVKF  KFVDILTNWYVRMNRRRLK  LSLCRLMAPYTPFLTELMY  YTPFLTELMYQNLKVLIDP  QDKDTLSIHYLMLPRVREE  RDRKTIPIKYPLKEIVVIH  LKDIKSLEKYIIEELNVRK  VTLSTDKNKYGIRLRAEPD  LHDEDIRLMYTFDQATGGT  LVPTDEITVYYKAKSEGTY  VPTDEITVYYKAKSEGTYL  YYKAKSEGTYLNSVIESHT  TTIKAPLKPYPVSPSDKVL  SSLPGPACAYVNLNICANG  NSSSTLLCQYINLQLLNAK  NGLTHQGLLYEAAKVFGLR  VKTLNMKTVYVSVLPTTAD  GPGRLHAASYLPAARAGPV  GGLLSPARLYAIAAKEKDI  DTLQKLGAVYDVSHYNALL  LGAVYDVSHYNALLKVYLQ  SHYNALLKVYLQNEYKFSP  LLKVYLQNEYKFSPTDFLA  ANIQPNRVTYQRLIASYCN  VTYQRLIASYCNVGDIEGA  AGIEPGPDTYLALLNAYAE  DTYLALLNAYAEKGDIDHV  IIFSFSKAGYPQYVSEILE  SFSKAGYPQYVSEILEKVT  LEKVTCERRYIPDAMNLIL  NTPVEKLTDYCKKLKEVQM  EEGFPIRPHYFWPLLVGRR  LGVHPDQETYTDYVIPCFD  HPDQETYTDYVIPCFDSVN  LWSEITELLYKDGRYCQEP  TELLYKDGRYCQEPRGPTE  PRGPTEAVGYFLYNLIDSM  PTEAVGYFLYNLIDSMSDS  QAKEEHLRQYFHQLEKMNV  MNVKIPENIYRGIRNLLES  RGIRNLLESYHVPELIKDA  QKALELKAKYESDMVTGGY  YESDMVTGGYAALINLCCR  SSAVLDTGKYVGLVRVLAK  TALEVAIDCYEKYKVLPRI  EVAIDCYEKYKVLPRIHDV  QEQGEMVMLYDLFFAFLQT  FFAFLQTGNYKEAKKIIET  LFECDRDQMYYNLLKLYKI  FECDRDQMYYNLLKLYKIN  QMYYNLLKLYKINGDWQRA  VPFDVPELWYEDEKHSLNS  CRLNQKKGAYDIFLNAKEQ  QNIVFNAETYSNLIKLLMS  LIKLLMSEDYFTQAMEVKA  LIITQVRRDYLKEAVTTLK  SENKVIEPQYFGLAYLFRK  IEPQYFGLAYLFRKVIEEQ  ERLANQFAIYKPVTDFFLQ  EAYNSLMKSYVSEKDVTSA  KDVTSAKALYEHLTAKNTK  KLDDLFLKRYASLLKYAGE  LKRYASLLKYAGEPVPFIE  IEPPESFEFYAQQLRKLRE  ARDLDELSRYPEDKITPEN  RTEEGPTLSYGRDGRSATR  GRSATREPPYRVPRDDWEE  HGSRSQESGYYDRMDYEDD  GSRSQESGYYDRMDYEDDR  ESGYYDRMDYEDDRLRDGE  SFFGETSHNYHKFDSEYER  HNYHKFDSEYERMGRGPGP  DFHGLLPKGYPHLCSICDL  RRCQLLLEIYPEWNPDNDT  DFQRGKNLRYQLLQLVEPF  TEDAQAAVDYYTTTPALVF  EDAQAAVDYYTTTPALVFG  PVRVHLSQKYKRIKKPEGK  HLSNLPHSGYSDSAVLKLA  SAVLKLAEPYGKIKNYILM  AEPYGKIKNYILMRMKSQA  CVKVDLSEKYKKLVLRIPN  KKDKSRKRSYSPDGKESPS  GQSDENKDDYTIPDEYRIG  KDDYTIPDEYRIGPYQPNV  IPDEYRIGPYQPNVPVGID  QPNVPVGIDYVIPKTGFYC  DYVIPKTGFYCKLCSLFYT  FYCKLCSLFYTNEEVAKNT  NTHCSSLPHYQKLKKFLNK  EEDLQFAERYLRSYYHPTN  QFAERYLRSYYHPTNLAGI  FAERYLRSYYHPTNLAGIL  RCGVPDVGEYNVFPRTLKW  LKWSKMNLTYRIVNYTPDM  MNLTYRIVNYTPDMTHSEV  FGIKEHGDFYPFDGPSGLL  AHAFPPGPNYGGDAHFDDD  ETWTSSSKGYNLFLVAAHE  DPGALMFPIYTYTGKSHFM  GALMFPIYTYTGKSHFMLP  DDVQGIQSLYGPGDEDPNP  GRKFWALNGYDILEGYPKK  LNGYDILEGYPKKISELGL  LFSGNQVWRYDDTNHIMDK  DTNHIMDKDYPRLIEEDFP  GIGDKVDAVYEKNGYIYFF  VDAVYEKNGYIYFFNGPIQ  AVYEKNGYIYFFNGPIQFE  FFNGPIQFEYSIWSNRIVR  IVRTSPNHRYTFTLKTHPS  IGQEIEVSLYTFDKAKQCI  QKKSIDSNPYDTDKMAAEF  CKHVKGILLYGPPGCGKTL  PAFGTNQEDYASYIMNGII  GTNQEDYASYIMNGIIKWG  AMKKIFDDAYKSQLSCVVV  VDDIERLLDYVPIGPRFSN  EMSLQMDPEYRVRKFLALL  FLIKRNKQTYSTEPNNLKA  NLKARNSFRYNGLIHRKTV  IRHMIRKNKYRPDLRMAAI  LVFKEDGQEYAQVIKMLGN  DIILVGLRDYQDNKADVIL  DNKADVILKYNADEARSLK  XXXMVQRLTYRRRLSYNTA  SRTPGNRIVYLYTKKVGKA  TPGNRIVYLYTKKVGKAPK  KTKKHVSRAYGGSMCAKCV  EAASLLGKKYGETANECGE  NECGEAFFFYGKSLLELAR  AREELREQVYDAMGEKEEA  GEVSVESENYVQAVEEFQS  QSCLNLQEQYLEAHDRLLA  HDRLLAETHYQLGLAYGYN  ETHYQLGLAYGYNSQYDEA  HYQLGLAYGYNSQYDEAVA  GLAYGYNSQYDEAVAQFSK  KEAEGSSAEYKKEIEELKE  MHPTVVISAYRKALDDMIS  GRKEIDIKKYARVEKIPGG  DVTHPRMRRYIKNPRIVLL  TRILQMEEEYIQQLCEDII  KGISDLAQHYLMRANITAI  AMTGVEQWPYRAVAQALEV  EPLAVKLQTYKTAVETAVL  XXXXXXMPQYQTWEEFSRA  EFSRAAEKLYLADPMKARV  PMKARVVLKYRHSDGNLCV  DFFKRNEHTYVHSSATIPL  DLDDVGKDVYHHTFFEMLG  MLGSWSFGDYFKELACKMA  EFGIPIERLYVTYFGGDEA  IPIERLYVTYFGGDEAAGL  PCGPCSEIHYDRIGGRDAA  EIWNLVFIQYNREADGILK  SVLQNKMSNYDTDLFVPYF  NYDTDLFVPYFEAIQKGTG  IQKGTGARPYTGKVGAEDA  EDADGIDMAYRVLADHART  GRPDNTGRGYVLRRILRRA  RRILRRAVRYAHEKLNASR  IPGDTAWLLYDTYGFPVDL  DTAWLLYDTYGFPVDLTGL  GEDLIMLDIYAIEELRARG  LEVTDDSPKYNYHLDSSGS  VTDDSPKYNYHLDSSGSYV  NYHLDSSGSYVFENTVATV  GVVLDKTCFYAEQGGQIYD  FYAEQGGQIYDEGYLVKVD  QGGQIYDEGYLVKVDDSSE  VKNAQVRGGYVLHIGTIYG  GYVLHIGTIYGDLKVGDQV  EMIEAAKAVYTQDCPLAAA  XXXXXMNEEYDVIVLGTGL  KVLHMDRNPYYGGESASIT  VLHMDRNPYYGGESASITP  ASITPLEDLYKRFKIPGSP  NGQLVKMLLYTEVTRYLDF  MLLYTEVTRYLDFKVTEGS  SFVYKGGKIYKVPSTEAEA  KRRFRKFLVYVANFDEKDP  PKKTTMRDVYKKFDLGQDV  DFTGHALALYRTDDYLDQP  ALALYRTDDYLDQPCYETI  TDDYLDQPCYETINRIKLY  YETINRIKLYSESLARYGK  KLYSESLARYGKSPYLYPL  SLARYGKSPYLYPLYGLGE  ARYGKSPYLYPLYGLGELP  QGFARLSAIYGGTYMLNKP  RLSAIYGGTYMLNKPIEEI  CKQLICDPSYVKDRVEKVG  NQVNRKSDIYVCMISFAHN  AHNVAAQGKYIAIVSTTVE  ESQIFISRTYDATTHFETT  TTCDDIKNIYKRMTGSEFD  EMKRKKNDIYGEDXXXXXX  XXXXXMALHYPKAPGFAQM  KHFSGLEEAYYRNIQACKE  YGPNGMNKMYINHLEKLFV  VINHLEKLFYTNDAATILR  AATILRELEYQHPAAKMIV  VQHPAAKMIYMASHMQEQE  MASHMQEQEYGDGTNFVLV  QEVGDGTNFYLVFAGALLE  VGDGTNFVLYFAGALLELA  EELLRIGLSYSEVIEGYEI  LRIGLSVSEYIEGYEIACR  KAHEILPNLYCCSAKNLRD  AKNLRDIDEYSSLLRTSIM  IMSKQYGNEYFLAKLIAQA  LAKLIAQACYSIFPDSGHF  IFPDSGHFNYDNIRVCKIL  GHFNVDNIRYCKILGSGIS  LGSGISSSSYLHGMVFKKE  SSSSVLHGMYFKKETEGDV  VFKKETEGDYTSVKDAKIA  KETEGDVTSYKDAKIAVYS  TSVKDAKIAYYSCPFDGMI  GMITETKGTYLIKTAEELM  GEENLMDAQYKAIADTGAN  KAIADTGANYVVTGGKVAD  AIADTGANVYVTGGKVADM  IADTGANVVYTGGKVADMA  ANVVVTGGKYADMALHYAN  YANKYNIMLYRLNSKWDLR  WDLRRLCKTYGATALPRLT  LEEMGHCDSYYLSEVGDTQ  HCDSVYLSEYGDTQVVVFK  YLSEVGDTQYVVFKHEKED  LSEVGDTQVYVFKHEKEDG  SEVGDTQVVYFKHEKEDGA  KEDGAISTIYLRGSTDNLM  NLMDDIERAYDDGVNTFKV  DIERAVDDGYNTFKVLTRD  VDDGVNTFKYLTRDKRLVP  KVLTRDKRLYPGGGATEIE  PRALAENSGYKANEVISKL  ENSGVKANEYISKLYAVHQ  NEVISKLYAYHQEGNKNVG  AVHQEGNKNYGLDIEAEVP  NVGLDIEAEYPAVKDMLEA  LDIEAEVPAYKDMLEAGIL  AIKLATNAAYTVLRVDQII  KLATNAAVTYLRVDQIIMA  TNAAVTVLRYDQIIMAKPA  AAGGRGKGAYQDRDKPAQI  SLNSKVVSQYSSLLSPMSV  IELALRLTEYSRTLSGMES  SRTLSGMESYCVRAFADAM  YVNKKFSNQYKATIGADFL  RFQSLGVAFYRGADCCVLV  ATKRAQAWCYSKNNIPYFE  WCYSKNNIPYFETSAKEAI  LKQETEVELYNEFPEPIKL  EPYANPTKRYRAFITNIPF  VKEKVGEVTYVELLMDAEG  STVFVANLDYKVGWKKLKE  KFNECGHVLYADIKMENGK  DKDDESLIKYKKTLLGDGP  TIVLKEGSEYRVKIHFKVN  NRDIVSGLKYVQHTYRTGV  SGLKYVQHTYRTGVKVDKA  DKATFMVGSYGPRPEEYEF  GSYGPRPEEYEFLTPVEEA  PKGMLARGTYHNKSFFTDD  EELKTQANDYFKAKDYENA  ANDYFKAKDYENAIKFYSQ  KDYENAIKFYSQAIELNPS  IELNPSNAIYYGNRSLAYL  ELNPSNAIYYGNRSLAYLR  IYYGNRSLAYLRTECYGYA  SLAYLRTECYGYALGDATR  AYLRTECYGYALGDATRAI  TRAIELDKKYIKGYYRRAA  ELDKKYIKGYYRRAASNMA  LDKKYIKGYYRRAASNMAL  GKFRAALRDYETVVKVKPH  PHDKDAKMKYQECNKIVKQ  IESMTIEDEYSGPKLEDGK  SFMKELMQWYKDQKKLHRK  QKKLHRKCAYQILVQVKEV  VCGDTHGQFYDLLNIFELN  NGLPSETNPYIFNGDFVDR  LTLFGFKLLYPDHFHLLRG  HETDNMNQIYGFEGEVKAK  GFEGEVKAKYTAQMYELFS  VKAKYTAQMYELFSEVFEW  AFLEENNLDYIIRSHEVKA  CVTVFSAPNYCDQMGNKAS  CDQMGNKASYIHLQGSDLR  PHPNVKPMAYANTLLQLGM  SEQTGKELLYKFICTTSAI  TSAIQNRFKYARVTPDTDW  VPHSQAEEFYVCIYATREG  QAEEFYVCIYATREGDYVL  CIYATREGDYVLFHHEGGV  SFISGLFNFYEDLYFTYLE  GLFNFYEDLYFTYLEINPL  NFYEDLYFTYLEINPLVVT  FPPPFGREAYPEEAYIADL  GREAYPEEAYIADLDAKSG  VAGGGASVVYSDTICDLGG  LGGVNELANYGEYSGAPSE  VNELANYGEYSGAPSEQQT  SGAPSEQQTYDYAKTILSL  APSEQQTYDYAKTILSLMT  KGIVRAIRDYQGPLKEHEV  AVQGMLDFDYVCSRDEPSV  DEPSVAAMVYPFTGDHKQK  FTGDHKQKFYWGHKEILIP  INFASLRSAYDSTMETMNY  YDSTMETMNYAQIRTIAII  LDNILASKLYRPGSVAYVS  KLYRPGSVAYVSRSGGMSN  IISRTTDGVYEGVAIGGDR  EGVAIGGDRYPGSTFMDHV  STFMDHVLRYQDTPGVKMI  LGEIGGTEEYKICRGIKEG  ELGEIIQSVYEDLVANGVI  VPPPTVPMDYSWARELGLI  CDERGQELIYAGMPITEVF  LWFQKRLPKYSCQFIEMCL  DMRVQILKDYVRQHFPATP  HFPATPLLDYALEVEKITT  SFTREEADEYIDIGALNGI  GRSMGFIGHYLDQKRLKQG  DQKRLKQGLYRHPWDDISY  YRHPWDDISYVLPEHMSMX  SIQPCPDVKYGKRIHVLPI  GITGNLFEVYLKPYFLEAY  NLFEVYLKPYFLEAYRPIR  YLKPYFLEAYRPIRKGDIF  KVVETDPSPYCIVAPDTVI  EEESLNEVGYDDIGGCRKQ  VKPPRGILLYGPPGTGKTL  KRELQELVQYPVEHPDKFL  MTPSKGVLFYGPPGCGKTL  SVSDNDIRKYEMFAQTLQQ  SGGGTGGSVYTEDNDDDLY  YTEDNDDDLYGXXXXXXXX  LDLVQQSCNYKQLRKGANE  LLCEDKNVPYVFVRSKQAL  KFGKITNDFYPEEDGKTKG  PEEDGKTKGYIFLEYASPA  KTKGYIFLEYASPAHAVDA  VDAVKNADGYKLDKQHTFR  VNLFTDFDKYMTISDEWDI  PFKDLGNLRYWLEEAECRD  LEEAECRDQYSVIFESGDR  EERARWTETYVRWSPKGTY  YVRWSPKGTYLATFHQRGI  LIDFSPCERYLVTFSPLMD  KLHWQKNGDYLCVKVDRTP  EAPRISVSFYHVKNNGKIE  CTVMNIAEHYMASDVEWDP  DVEWDPTGRYVVTSVSWWS  WWSHKVDNAYWLWTFQGRL  KQIKKDLKKYSKIFEQKDR  RTMMEDFRKYRKMAQELYM  KYRKMAQELYMEQKNERLE  VFMLHIWGKYTRSXXXXXX  LSESLLRGIYAYGFEKPSA  ESLLRGIYAYGFEKPSAIQ  QKVVMALGDYMGASCHACI  RVFDMLNRRYLSPKYIKMF  LNRRYLSPKYIKMFVLDEA  LTLEGIRQFYINVEREEWK  WKLDTLCDLYETLTITQAV  VQQVSLVINYDLPTNRENY  YDLPTNRENYIHRIGRGGR  RTLRDIETFYNTSIEEMPL  AEPDESNARYFHVVIAGPQ  KLELFLPEEYPMAAPKVRF  ETARAWTRLYAMNNIXXXX  CGTGYTKLGYAGNTEPQFI  GDEAIEKPTYATKWPIRHG  RFMEQVIFKYLRAEPEDHY  YLRAEPEDHYFLLTEPPLN  PLNTPENREYTAEIMFESF  FESFNVPGLYIAVQAVLAL  THVIPVAEGYVIGSCIKHI  IPIAGRDITYFIQQLLRDR  ETAKAVKERYSYVCPDLVK  AKAVKERYSYVCPDLVKEF  PDLVKEFNKYDTDGSKWIK  TDGSKWIKQYTGINAISKK  KKEFSIDVGYERFLGPEIF  CPIDVRRPLYKNIVLSGGS  QVITHHMQRYAVWFGGSML  SMLASTPEFYQVCHTKKDY  YQVCHTKKDYEEIGPSICR  NLEKETTHRYCANAFKLHR  GKQKPNLGKYDDPPDWQEI  PPDWQEILTYFRGSELQNY  DLKAIIKPQYVDQIPKAAK  IFMFDEPSSYLDVKQRLKA  IRSLINPDRYIIVVEHDLS  VEHDLSVLDYLSDFICCLY  YLSDFICCLYGVPSAYGVV  CCLYGVPSAYGVVTMPFSV  EGINIFLDGYVPTENLRFR  EEEVKKMCMYKYPGMKKKM  EVKKMCMYKYPGMKKKMGE  GEVPVLNVSYKPQKISPKS  LLHEKIRDAYTHPQFVTDV  LCLGKPADVYLIDEPSAYL  VYLIDEPSAYLDSEQRLMA  VEHDFIMATYLADRVIVFD  ITFRRDPNNYRPRINKLNS  VDPFSKKDWYDVKAPAMFN  HVDVKTTDGYLLRLFCVGF  DIEKACQSIYPLHDVFVRK  GAKVERADGYEPPVQESVX  VGDKVLLPEYGGTKVVLDD  FRDGDILGKYVDXXXXXXX  QKSTCGKCGYPAKRKRKYN  GYPAKRKRKYNWSAKAKRR  GRMRHLKIVYRRFRHGFRE  ESDAVECLNYQHYKGSDFD  AVECLNYQHYKGSDFDCEL  SPIKGRAQPYDPNFYDETY  RAQPYDPNFYDETYDYGGF  YDPNFYDETYDYGGFTMMF  PNFYDETYDYGGFTMMFDD  RPMPPSRRDYDDMSPRRGP  DRRGRPGDRYDGMVGFSAD  WSPSEWQMAYEPQGGSGYD  AYEPQGGSGYDYSYAGGRG  EPQGGSGYDYSYAGGRGSY  QGGSGYDYSYAGGRGSYGD  YSYAGGRGSYGDLGGPIIT  TQDQIQNAQYLLQNSVKQY  YLLQNSVKQYSGKFFXXXX  KICGDIHGQYYDLLRLFEY  ICGDIHGQYYDLLRLFEYG  YYDLLRLFEYGGFPPESNY  YGGFPPESNYLFLGDYVDR  ESNYLFLGDYVDRGKQSLE  LETICLLLAYKIKYPENFF  CLLLAYKIKYPENFFLLRG  HECASINRIYGFYDECKRR  ASINRIYGFYDECKRRYNI  GFYDECKRRYNIKLWKTFT  LVTLFSAPNYCGEFDNAGA  KPADKNKGKYGQFSGLNPG  GKAGSGLRQYYLSKIEELQ  KAGSGLRQYYLSKIEELQL  LQLLQEQGSYVGEVVRAMD  MVEKVPDSTYEMIGGLDKQ  IAQPKGVLLYGPPGTGKTL  KGVCTEAGMYALRERRVHV  RKTGGKRKPYHKKRKYELG  RKPYHKKRKYELGRPAANT  VRVRGGNKKYRALRLDVGN  CIVLIDSTPYRQWYESHYA  IDSTPYRQWYESHYALPLG  PYRQWYESHYALPLGRKKG  KRSKKIQKKYDERKKNAKI  VLEGKELEFYLRKIKARKG  EMIEPRTLQYKLLEPVLLL  KGGGHVAQIYAIRQSISKA  QSISKALVAYYQKYVDEAS  SISKALVAYYQKYVDEASK  KALVAYYQKYVDEASKKEI  FGGPGARARYQKSYRXXXX  GARARYQKSYRXXXXXXXX  XXXMGHQQLYWSHPRKFGQ  NMCRQCFRQYAKDIGFIKL  MADIQTERAYQKQPTIFQN  ETGKEKLPRYYKNIGLGFK  TGKEKLPRYYKNIGLGFKT  QRTIVIRRDYLHYIRKYNR  IVIRRDYLHYIRKYNRFEK  RDYLHYIRKYNRFEKRHKN  IIFLRNRLKYALTGDEVKK  KTGENFRLIYDTKGRFAVH  HRITPEEAKYKLCKVRKIF  VTHDARTIRYPDPLIKVND  TLRLRRQPKYPRKSAPRRN  APRRNKLDHYAIIKFPLTT  IRPDGEKKAYVRLAPDYDA  ALKRQGRTLYGFGGXXXXX  HLTGEFEKKYVATLGVEVH  EKFGGLRDGYYIQAQCAII  KFGGLRDGYYIQAQCAIIM  MFDVTSRVTYKNVPNWHRD  VFHRKKNLQYYDISAKSNY  YYDISAKSNYNFEKPFLWL  VMDPALAAQYEHDLEVAQT  KFIRHQSDRYVKIKRNWRK  GQILMPNIGYGSNKKTKHM  AVDFAERHGYIKGIVKDII  LAKVVFRDPYRFKKRTELF  EGIHTGQFVYCGKKAQLNI  KPILKAGRAYHKYKAKRNC  LKAGRAYHKYKAKRNCWPR  XXXMEAIAKYDFKATADDE  LNEECDQNWYKAELNGKDG  GKDGFIPKNYIEMKPHPWF  KVLRDGAGKYFLWVVKFNS  FNSLNELVDYHRSTSVSRN  IEQVPQQPTYVQALFDFDP  GRFNGQFKTYAICGAIRRM  LWNTLGVCKYTVQDESHSE  KTNHIGHTGYLNTVTVSPD  WDLNEGKHLYTLDGGDIIN  NALCFSPNRYWLCAATGPS  ADGQTLFAGYTDNLVRVWQ  IGGKSPDTNYLFMGDYVDR  DTNYLFMGDYVDRGYYSVE  FMGDYVDRGYYSVETVTLL  MGDYVDRGYYSVETVTLLV  TLLVALKVRYRERITILRG  HESRQITQVYGFYDECLRK  RQITQVYGFYDECLRKYGN  GFYDECLRKYGNANVWKYF  KYGNANVWKYFTDLFDYLP  WKYFTDLFDYLPLTALVDG  WGISPRGAGYTFGQDISET  RAHQLVMEGYNWCHDRNVV  VVTIFSAPNYCYRCGNQAA  TIFSAPNYCYRCGNQAAIM  PHVTRRTPDYFLXXXXXXX  AEMGKGSFKYAWVLDKLKA  SLWKFETSKYYVTIIDAPG  LWKFETSKYYVTIIDAPGH  QTREHALLAYTLGVKQLIV  NKMDSTEPPYSQKRYEEIV  TEPPYSQKRYEEIVKEVST  EEIVKEVSTYIKKIGYNPD  VSTYIKKIGYNPDTVAFVP  NHPGQISAGYAPVLDCHTA  PMCVESFSDYPPLGRFAVR  EALGRLLVVYPWTQRFFES  GGSRGAPQHYPKTAGNSEF  VDKALEEPKYSSLYAQLCL  LEEPKYSSLYAQLCLRLAE  ENRTRNVDVYDKRENPLLP  ERAKSLMDQYFARMCSLML  MKSQGLSQLYHNQSQGLLS  KLTETVVTEYLNSGNANEA  EVDIPLVKSYLAQFAARAI  NILMTSFLQYISSEVNPPS  HVDLQVSALYALQVHCYNS  ALYALQVHCYNSNFPKGML  MLLRFFVHFYDMEIIEEEA  NCFINRQLIYNYPEQLFGA  FINRQLIYNYPEQLFGAAG  QTVKDSRTVYGGGCSEMLM  GKEAVAMESYAKALRMLPT  PTIIADNAGYDSADLVAQL  PVPEGSIRIYSMRFCPFAE  LENSQGQLIYESAITCEYL  IYESAITCEYLDEAYPGKK  ITCEYLDEAYPGKKLLPDD  FIRSQNKEDYAGLKEEFRK  GGNSISMIDYLIWPWFERL  KDWQGFLELYLQNSPEACD  LQNSPEACDYGLXXXXXXX  LCSFSGYKIYPGHGRRYAR  KIYPGHGRRYARTDGKVFQ  PRQINWTVLYRRKHKKGQS  DSCPLDCKVYVGNLGNNGN  TELERAFGYYGPLRSVWVA  SWGRRPRDDYRRRSPPPRR  LAAAAVEEQYSCDYGSGRF  AVEEQYSCDYGSGRFFILC  CRMQVDPQKYKGIFNGFSV  KGWAPTFLGYSMQGLCKFG  MQGLCKFGFYEVFKVLYSN  GFYEVFKVLYSNMLGEENT  SNMLGEENTYLWRTSLYLA  NTYLWRTSLYLAASASAEF  TLRDAAPKMYKEEGLKAFY  YKEEGLKAFYKGVAPLWMR  APLWMRQIPYTMMKFACFE  CFERTVEALYKFVVPKPRS  QLVVTFVAGYIAGVFCAIV  TLTALQWFIYDSVKVYFRL  WFIYDSVKVYFRLPRPPPP  VALVTDNAVYHWSMEGESQ  SLAGCQIINYRTDAKQKWL  NRVVGAMQLYSVDRKVSQP  HDVVFLITKYGYIHLYDLE  VVFLITKYGYIHLYDLETG  ITKYGYIHLYDLETGTCIY  YDLETGTCIYMNRISGETI  CVEEENIIPYITNVLQNPD  PGQTSPLLQYFGILLDQGQ  LLDQGQLNKYESLELCRPV  VDPTLALSVYLRANVPNKV  TGQVQKIVLYAKKVGYTPD  IVLYAKKVGYTPDWIFLLR  TQIVDVFMEYNLIQQCTAF  ILGNQMFTHYDRAHIAQLC  RALEHFTDLYDIKRAVVHT  LLNPEWLVNYFGSLSVEDS  QICVQVASKYHEQLSTQSL  SFKSFEGLFYFLGSIVNFS  SQDPDVHFKYIQAACKTGQ  ERICRESNCYDPERVKNFL  FDFVHDLVLYLYRNNLQKY  FVHDLVLYLYRNNLQKYIE  YLYRNNLQKYIEIYVQKVN  NNLQKYIEIYVQKVNPSRL  PERFLRENPYYDSRVVGKY  ERFLRENPYYDSRVVGKYC  YYDSRVVGKYCEKRDPHLA  NSLFKSLSRYLVRRKDPEL  GSVLLESNPYRRPLIDQVV  KADRTRVMEYINRLDNYDA  MEYINRLDNYDAPDIANIA  EHIGNLDRAYEFAERCNEP  GMVKEAIDSYIKADDPSSY  YIKADDPSSYMEVVQAANT  SGNWEELVKYLQMARKKAR  MARKKARESYVETELIFAL  HIQQVGDRCYDEKMYDAAK  GDRCYDEKMYDAAKLLYNN  KMYDAAKLLYNNVSNFGRL  ADELEELINYYQDRGYFEE  DELEELINYYQDRGYFEEL  LINYYQDRGYFEELITMLE  GMFTELAILYSKFKPQKMR  HLWAELVFLYDKYEEYDNA  AELVFLYDKYEEYDNAIIT  VFLYDKYEEYDNAIITMMN  ITKVANVELYYRAIQFYLE  TKVANVELYYRAIQFYLEF  ELYYRAIQFYLEFKPLLLN  RLDHTRAVNYFSKVKQLPL  VKQLPLVKPYLRSVQNHNN  NNLFITEEDYQALRTSIDA  QALRTSIDAYDNFDNISLA  LIEFRRIAAYLFKGNNRWK  VELCKKDSLYKDAMQYASE  DSLYKDAMQYASESKDTEL  CFGACLFTCYDLLRPDVVL  HNIMDFAMPYFIQVMKEYL  PYFIQVMKEYLTKVDKLDA  QATETQPIVYGQPQLMLTA  AVPPQAPFGYGYTAPPYGQ  PPQAPFGYGYTAPPYGQPQ  FGYGYTAPPYGQPQPGFGY  YGQPQPGFGYSMXXXXXXX  RPREDHGRGYFEYIEENKY  EDHGRGYFEYIEENKYSRA  YFEYIEENKYSRAKSPQPP  DDTVVCLDTYNCDLHFKIS  FLWAGGRASYGVSKGKVCF  TEKIPVRHLYTKDIDIHEV  MLLGEEEFSYGYSLKGIKT  LGEEEFSYGYSLKGIKTCN  KTCNCETEDYGEKFDENDV  FESDEVELSYAKNGQDLGV  FNFGQKEKPYFPIPEEYTF  KPYFPIPEEYTFIQNVPLE  VVVCPKDEDYKQRTQKKAE  VAECFDEITYVELQKEEAQ  EEAQKLLEQYKEESKKALP  GGAPGNRGGYNRRGNMPQR  GGGGSGGIGYPYPRAPVFP  GGSGGIGYPYPRAPVFPGR  APVFPGRGSYSNRGNYNRG  RGSYSNRGNYNRGGMPNRG  RGGMPNRGNYNQNFRGRGN  FRGRGNNRGYKNQSQGYNQ  RGYKNQSQGYNQWQQGQFW  WGQKPWSQHYHQGYYXXXX  PWSQHYHQGYYXXXXXXXX  WSQHYHQGYYXXXXXXXXX  TSLKVDNLTYRTSPDTLRR  DTLRRVFEKYGRVGDVYIP  EKYGRVGDVYIPRDRYTKE  GDVYIPRDRYTKESRGFAF  RELRVQMARYGRPPDSHHS  SRRGPPPRRYGGGGYGRRS  PPRRYGGGGYGRRSRSPRR  RSRSRSRSRYSRSKSRSRT  SHTSDMHRGYADSPSKAGA  SPSKAGAAPYVQAFDSLLA  SLLAGPVAEYLKISKEIGG  VAMAPKPGPYVKEMNDAAM  KEMNDAAMFYTNRVLKEYK  FYTNRVLKEYKDVDKKHVD  KKHVDWVKAYLSIWTELQA  LSIWTELQAYIKEFHTTGL  PPVSTISCSYESASRSSLF  EDTELKQVAYIYKCVNTTL  TELKQVAYIYKCVNTTLQI  MKASGTLREYKVVGRCLPT  TPKCHTPPLYRMRIFAPNH  HVVAKSRFWYFVSQLKKMK  MKKSSGEIVYCGQVFEKSP  VKNFGIWLRYDSRSGTHNM  DSRSGTHNMYREYRDLTTA  SGTHNMYREYRDLTTAGAV  MIGDRVFVHYTGWLLDGTK  VCHITCKPEYAYGSAGSPP  HITCKPEYAYGSAGSPPKI  RRIQTRGEGYAKPNEGAIV  AIVEVALEGYYKDKLFDQR  IVEVALEGYYKDKLFDQRE  MEKGEHSIVYLKPSYAFGS  HSIVYLKPSYAFGSVGKEK  QIPPNAELKYELHLKSFEK  TIVKERGTVYFKEGKYKQA  GTVYFKEGKYKQALLQYKK  GKYKQALLQYKKIVSWLEY  YKKIVSWLEYESSFSNEEA  ADFQKVLQLYPNNKAAKTQ  RQLAREKKLYANMFERLAE  PVLVRGIGRYSRSAMYSRK  IGRYSRSAMYSRKAMYKRK  SAMYSRKAMYKRKYSAAKS  SRKAMYKRKYSAAKSKVEK  VVKLRKMPRYYPTEDVPRK  VKLRKMPRYYPTEDVPRKL  KIPKHLTDAYFKKKKLRKP  IKAIPQLQGYLRSVFALTN  SVFALTNGIYPHKLVFXXX  CNHPDLDVQYRSGAAPQPG  AEPAQASGPYVEIIEQPKQ  PKQRGMRFRYKCEGRSAGS  VGKDCRDGFYEAELCPDRC  KVQKEDIEVYFTGPGWEAR  VAIVFRTPPYADPSLQAPV  ELSEPMEFQYLPDTDDRHR  RIEEKRKRTYETFKSIMKK  SVPKPAPQPYPFTSSLSTI  FTSSLSTINYDEFPTMVFP  HTTEPMLMEYPEAITRLVT  FRPGLRDVAYQYVKKGSRI  PGLRDVAYQYVKKGSRIYL  QYVKKGSRIYLEGKIDYGE  RIYLEGKIDYGEYMDKNNV  LEGKIDYGEYMDKNNVRRQ  TSPLHLAAQYGHYSTTEVL  LHLAAQYGHYSTTEVLLRA  DEANREAQKYRQQLLKKEQ  LKKEQEAEAYRQKLEAMTR  QFKDISLSDYKGKYVVFFF  ISLSDYKGKYVVFFFYPLD  KGKYVVFFFYPLDFTFVCP  DPKRTIAQDYGVLKADEGI  PAGNNDCRIYVGNLPPDIR  KDIEDVFYKYGAIRDIDLK  DPRDAEDAVYGRDGYDYDG  EDAVYGRDGYDYDGYRLRV  AVYGRDGYDYDGYRLRVEF  GRDGYDYDGYRLRVEFPRS  GGGGAPRGRYGPPSRRSEN  HMREAGDVCYADVYRDGTG  AGDVCYADVYRDGTGVVEF  KVDGPRSPSYGRSRSRSRS  SRSNSRSRSYSPRRSRGSP  PRRSRGSPRYSPRHSRSRS  XXXXXMPRVYIGRLSYQAR  PRVYIGRLSYQARERDVER  RDVERFFKGYGKILEVDLK  ILEVDLKNGYGFVEFDDLR  DLRDADDAVYELNGKDLCG  ARGPRRDGSYGSGRSGYGY  GSYGSGRSGYGYRRSGRDK  YGSGRSGYGYRRSGRDKYG  GYRRSGRDKYGPPTRTEYR  KYGPPTRTEYRLIVENLSS  RCSWQDLKDYMRQAGEVTY  NEGVIEFVSYSDMKRALEK  KPGSRRRRSYSRSRSHSRS  XMGDVKNFLYAWCGKRKMT  CGKRKMTPSYEIRAVGNKN  FMCEVQVEGYNYTGMGNST  CEVQVEGYNYTGMGNSTNK  SNAARDFVNYLVRINEIKS  NNSEVGASGYGVPGPTWDR  WDRGANLKDYYSRKEEQEV  DRGANLKDYYSRKEEQEVQ  ENAKARLNQYFQKEKIQGE  FQKEKIQGEYKYTQVGPDH  RSFIAEMTIYIKQLGRRIF  CALSLVRQLYHLGVVEAYS  LYHLGVVEAYSGLTKKKEG  KKEGETVEPYKVNLSQDLE  SMDLKNELMYQLEQDHDLQ  GEEPGKSCGYSVRFESILP  VVLRDVVQAYPEVRIVLMS  TIDTSMFCEYFFNCPIIEV  FFNCPIIEVYGRTYPVQEY  PIIEVYGRTYPVQEYFLED  YGRTYPVQEYFLEDCIQMT  NCNLICGDEYGPETRLSMS  FELIEALLKYIETLNVPGA  VFLPGWNLIYTMQKHLEMN  MNPHFGSHRYQILPLHSQI  TSITINDVVYVIDSCKQKV  FTAHNNMTNYATVWASKTN  KMMIMGCIFYVGDAICTIA  FINEGKRLGYIHRNFAGNR  VISLLAFGVYPNVCYHKEK  AFGVYPNVCYHKEKRKILT  CPFSSQDMKYPSPFFVFGE  INLMIGSTRYGDGPRPPKM  GPRPPKMARYDNGSGYRRG  MARYDNGSGYRRGGSSYSG  SGYRRGGSSYSGGGYGGGY  GGSSYSGGGYGGGYSSGGY  YSGGGYGGGYSSGGYGSGG  YGGGYSSGGYGSGGYGGSA  SSGGYGSGGYGGSANSFRA  GSANSFRAGYGAGVGGGYR  GYGAGVGGGYRGVSRGGFR  GFRGNSGGDYRGPSGGYRG  GDYRGPSGGYRGSGGFQRG  FQRGGGRGAYGTGYFGQGR  GGRGAYGTGYFGQGRGGGG  FGQGRGGGGYXXXXXXXXX  RGEAGWEGGYPEIVKENKL  VKENKLFEHYYQELKIVPE  KENKLFEHYYQELKIVPEG  LPATLRITGYKSHAKEILH  EILHCLKNKYFKELEDLEV  VEVPQPLSWYPEELAWHTN  ANDVDNKRCYLLVHQAKRL  VDGRKEILFYDRILCDVPC  QLAEGGRMVYSTCSLNPIE  PLFPPIEKFYALDPSFPRM  TTEGKKRQLYMVSKELRNV  AFRLAQEGIYTLYPFINSR  LAQEGIYTLYPFINSRIIT  LAKGSIVLKYEPDSANPDA  FVPKNERLHYLRMMGLEVL  VEERVINEEYKIWKKNTPF  IWKKNTPFLYDLVMTHALE  DDAQFDASHYDSEKGEFGG  HEGEVNRARYMPQNPCIIA  RLRGHQKEGYGLSWNPNLS  EVNCLSFNPYSEFILATGS  QVWQMAENIYNDEDPEGSV  FVNDDRHVMYKHSSVYPTQ  VYPTQEELEYVQNMVSHTE  QNMVSHTERYLKAVSDWID  VSHTERALKYVSDWIDEQE  EQEKGSSEQYESDNMDVPP  PPEDDSKEGYGEQKTEHMT  RGVMRVGLVYKGLLLKGDL  LLCKEKPTTYLLDKVADNL  PTTALLDKVYDNLAIQLAA  LLDKVADNLYIQLAAVTED  VADNLAIQLYAVTEDKYEI  ADNLAIQLAYVTEDKYEIL  YEILQSVDDYAIVIKNTKE  EILQSVDDAYIVIKNTKEP  VREEMEKVLYGETLSVNDP  DVLDRQKCLYALASLRHAK  VLDRQKCLAYLASLRHAKW  DRQKCLAALYSLRHAKWFQ  LAALASLRHYKWFQARANG  SLRHAKWFQYRANGLKSCV  RHAKWFQARYNGLKSCVIV  LLCEKSIGTYNRPMGAGEA  IGTANRPMGYGEALRRVLE  ANRPMGAGEYLRRVLECLA  ALRRVLECLYSGIVMPDGS  GIYDPCEKEYTDAIGHLDR  DPCEKEATDYIGHLDRQQR  QQREDITQSYQHALRLAAF  EDITQSAQHYLRLAAFGQL  QSAQHALRLYAFGQLHKVL  SAQHALRLAYFGQLHKVLG  VQIPPSTTYYITPMKRPME  KKIQKKEEKYEPPQAMNAL  KEEKAEPPQYMNALMRLNQ  KAEPPQAMNYLMRLNQLKP  LVSQTGPVHYPIFTMSVEV  VEVDGNSFEYSGPSKKTAK  EASGPSKKTYKLHVAVKVL  SKKTAKLHVYVKVLQDMGL  LQDMGLPTGYEGRDSSKGE  RDSSKGEDSYEETEAKPAV  GEDSAEETEYKPAVVAPAP  SAEETEAKPYVVAPAPVVE  ETEAKPAVVYPAPVVEAVS  EAKPAVVAPYPVVEAVSTP  VVAPAPVVEYVSTPSAAFP  VVEAVSTPSYAFPSDATAE  VEAVSTPSAYFPSDATAEQ  TPSAAFPSDYTAEQGPILT  SAAFPSDATYEQGPILTKH  EVDGQKFQGYGSNKKVAKA  QGAGSNKKVYKAYAALAAL  SNKKVAKAYYALAALEKLF  NKKVAKAYAYLAALEKLFP  KVAKAYAALYALEKLFPDT  VAKAYAALAYLEKLFPDTP  EKLFPDTPLYLDANKKKRA  FPDTPLALDYNKKKRAPVP  ALDANKKKRYPVPVRGGPK  VPVRGGPKFYAKPHNPGFG  PVRGGPKFAYKPHNPGFGM  RGRGRGFGGYNHGGYMNAG  GANHGGYMNYGAGYGSYGY  NHGGYMNAGYGYGSYGYGG  GSYGYGGNSYTAGYSQFYS  YGYGGNSATYGYSQFYSNG  YSNGGHSGNYSGGGGGGGG  YNSPVPPKHYGKKQPHGGQ  GGNSYGSGGYSYNPGSHGG  SSQGGYGRNYDHSMNYQYR  AEKKLEENPYDLDAWSILI  QPIDKARKTYERLVAQFPS  PSSGRFWKLYIEAEIKAKN  IEAEIKAKNYDKVEKLFQR  VLHIDLWKCYLSYVRETKG  IDLWKCYLSYVRETKGKLP  RETKGKLPSYKEKMAQAYD  SYKEKMAQAYDFALDKIGM  DKIGMEIMSYQIWVDYINF  IMSYQIWVDYINFLKGVEA  QRITAVRRVYQRGCVNPMI  INIEQLWRDYNKYEEGINI  EQLWRDYNKYEEGINIHLA  KMIEDRSRDYMNARRVAKE  MNARRVAKEYETVMKGLDR  AQQVDMWKKYIQWEKSNPL  LITKRVMFAYEQCLLVLGH  VLGHHPDIWYEAAQYLEQS  PDIWYEAAQYLEQSSKLLA  LFSDEAANIYERAISTLLK  TLLKKNMLLYFAYADYEES  KKNMLLYFAYADYEESRMK  MLLYFAYADYEESRMKYEK  ADYEESRMKYEKVHSIYNR  MKYEKVHSIYNRLLAIEDI  IEDIDPTLVYIQYMKFARR  IDPTLVYIQYMKFARRAEG  EDTRTRHHVYVTAALMEYY  VYVTAALMEYYCSKDKSVA  YVTAALMEYYCSKDKSVAF  KIFELGLKKYGDIPEYVLA  LKKYGDIPEYVLAYIDYLS  GDIPEYVLAYIDYLSHLNE  PEYVLAYIDYLSHLNEDNN  RRFTAFKEEYEGKETALLV  KETALLVDRYKFMDLYPCS  VDRYKFMDLYPCSASELKA  SASELKALGYKDVSRAKLA  KDEVDRKPEYPKPDTQQMI  AVVPPVHDIYRARQQKRIR  XXXXXXMSEYIRVTEDEND  QFPGACGLRYRNPVSQCMR  PDAGWGNLVYVVNYPKDNK  WGNLVYVVNYPKDNKRKMD  KTTEQDLKEYFSTFGEVLM  DELREFFSQYGDVMDVFIP  QAFGSGNNSYSGSNSGAAI  KDAEIQEREYHLKELREQL  VSAEPTTRTYDLNKPPEFS  QIKDMVECMYGRNYQQGSY  MVECMYGRNYQQGSYIIKQ  YGRNYQQGSYIIKQGEPGN  TTFGELAILYNCTRTASVK  RTAQARDEQYRNFLRSVSL  KIIDCLEVEYYDKGDYIIR  IIDCLEVEYYDKGDYIIRE  EVEYYDKGDYIIREGEEGS  VGTFEELQKYLEGYVANLN  EELQKYLEGYVANLNRDDE  VDTKQQEHVYSEKRILEEL  LCSPFIVKLYRTFKDNKYV  LYRTFKDNKYVYMLLEACL  RTFKDNKYVYMLLEACLGG  VACVTEAFDYLHRLGIIYR  DYLHRLGIIYRDLKPENLI  ENLILDAEGYLKLVDFGFA  TWTFCGTPEYVAPEVILNK  DFWSLGILVYELLTGNPPF  FSGVDQMMTYNLILKGIEK  ELKGPIDHSYFDKYPPEKG  PIDHSYFDKYPPEKGMPPD  FSFGINSILYQRGIYPSET  NSILYQRGIYPSETFTRVQ  SETFTRVQKYGLTLLVTTD  VTTDLELIKYLNNVVEQLK  VVEQLKDWLYKCSVQKLVV  VSCSFDLLIYTDKDLVVPE  CFSKDIVENYFMRDSGSKA  CEDNAPATSYCVECSEPLC  CVEAHQRVKYTKDHTVRST  KSRDGERTVYCNVHKHEPL  ALLLSKKLIYFQLHRALKM  SQPMEVQEGYGFGSGDDPY  YGFGSGDDPYSSAEPHVSG  RLQEKLSPPYSSPQEFAQD  HERSITQIKYNREGDLLFT  AKDPIVNVWYSVNGERLGT  SVNGERLGTYMGHTGAVWC  MFSTDKQMGYQCFVSFFDL  PSQIDNNEPYMKIPCNDSK  GHESGELNQYSAKSGEVLV  VNSAALSPNYDHVVLGGGQ  VAFHPDGKSYSSGGEDGYV  GEDGYVRIHYFDPQYFEFE  VRIHYFDPQYFEFEFEAXX  ETPMIGDKVYVHYKGKLSN  MIGDKVYVHYKGKLSNGKK  ICHLLCKPEYAYGSAGSLP  HLLCKPEYAYGSAGSLPKI  RRTKRKGEGYSNPNEGATV  QCILYLGPRYGFGEAGKPK  GIEPNAELIYEVTLKSFEK  AIVKEKGTVYFKGGKYMQA  GTVYFKGGKYMQAVIQYGK  GKYMQAVIQYGKIVSWLEM  KIVSWLEMEYGLSEKESKA  AAFLNLAMCYLKLREYTKA  AMCYLKLREYTKAVECCDK  LDSANEKGLYRRGEAQLLM  EHNERDRRIYANMFKKFAE  TQGTRRKVCYYYDGDVGNY  QGTRRKVCYYYDGDVGNYY  GTRRKVCYYYDGDVGNYYY  YYYDGDVGNYYYGQGHPMK  YYDGDVGNYYYGQGHPMKP  YDGDVGNYYYGQGHPMKPH  RMTHNLLLNYGLYRKMEIY  HNLLLNYGLYRKMEIYRPH  YGLYRKMEIYRPHKANAEE  KANAEEMTKYHSDDYIKFL  EMTKYHSDDYIKFLRSIRP  KKSEASGFCYVNDIVLAIL  VLAILELLKYHQRVLYIDI  LLKYHQRVLYIDIDIHHGD  HGDGVEEAFYTTDRVMTVS  RVMTVSFHKYGEYFPGTGD  TVSFHKYGEYFPGTGDLRD  RDIGAGKGKYYAVNYPLRD  DIGAGKGKYYAVNYPLRDG  GKGKYYAVNYPLRDGIDDE  LRDGIDDESYEAIFKPVMS  PMLMLGGGGYTIRNVARCW  IRNVARCWTYETAVALDTE  DTEIPNELPYNDYFEYFGP  IPNELPYNDYFEYFGPDFK  ELPYNDYFEYFGPDFKLHI  NMTNQNTNEYLEKIKQRLF  VSSRREPPPYGYRKGWIPR  SRREPPPYGYRKGWIPRLL  AFPEIHVAQYPLDMGRKKK  QVDSEGKIKYDAIARQGQS  QGQSKDKVIYSKYTDLVPK  SKDKVIYSKYTDLVPKEVM  AADKLAPAQYIRYTPSQQG  KLAPAQYIRYTPSQQGVAF  ISNWKNAKGYTIPLDKRLA  NFAKLAEALYIADRKAREA  NPRTSNEVQYDQRLFNQSK  GFAGGEDEIYNVYDQAWRG  GGEDEIYNVYDQAWRGGKD  PSKNLDKDMYGDDLEARIK  LFVITKPDVYKSPASDTYI  ERRQQVLDRYHRFKELSTL  LRRQKLEDSYRFQFFQRDA  LLQAQKLVQYLRECEDVMD  ERHARLNDSYRLQRFLADF  GQALLAAGHYASDEVREKL  ELWELRRQQYEQCMDLQLF  EQCMDLQLFYRDTEQVDNW  ATKLIQNNHYAMEDVATRR  KMKTATDEAYKDPSNLQGK  GQKLIDVNHYAKDEVAARM  NVEDIELWLYEVEGHLASD  VEGHLASDDYGKDLTNVQN  KKQEALVARYEALKEPMVA  LEDSLQAQQYFADANEAES  KEPIVGSTDYGKDEDSAEA  KELVLALYDYQEKSPREVT  DRQGFVPAAYVKKLDPAQS  LRMKQVEELYHSLLELGEK  VQAVQQQEVYGMMPRDETD  GQQLLAHGHYASPEIKQKL  KLQTASDESYKDPTNIQSK  VEALLASEDYGKDLASVNN  KKLLVGSEDYGRDLTGVQN  GQRLEESLEYQQFVANVEE  KMTLVASEDYGDTLAAIQG  KENSLKTDDYGRDLSSVQT  KSFRVASNPYTWFTMEALE  HQWIQETRTYLLDGSCMVE  EEALILDNKYTEHSTVGLA  FKSCLRSLGYDLPMVEEGE  RDGHVSLQEYMAFMISRET  KPYVTKEELYQNLTREQAD  QNLTREQADYCVSHMKPYV  DYCVSHMKPYVDGKGRELP  GRELPTAFDYVEFTRSLFV  XMVNENTRMYIPEENHQGS  PEENHQGSNYGSPRPAHAN  SSTQRKRQQYGKPKKQGST  FANCVALAIYIPFPEDDSN  TNSNLERVEYLFLIIFTVE  VEAFLKVIAYGLLFHPNAY  YGLLFHPNAYLRNGWNLLD  LLVLFVIIIYAIIGLELFM  FMGKMHKTCYNQEGIADVP  TMEGWTDVLYWVNDAVGRD  AVGRDWPWIYFVTLIIIGS  QQLEEDLKGYLDWITQAED  RISKSKFSRYWRRWNRFCR  RAAVKSNVFYWLVIFLVFL  NTLTIASEHYNQPNWLTEV  FTAEMLLKMYSLGLQAYFV  KMYSLGLQAYFVSLFNRFD  LLRIFKITRYWNSLSNLVA  TGEDWNSVMYDGIMAYGGP  SVMYDGIMAYGGPSFPGML  SFPGMLVCIYFIILFICGN  FIILFICGNYILLNVFLAI  PNENEDKSPYPNPETTGEE  TSFRNHILFYFDIVFTTIF  ALKILGNADYVFTSIFTLE  LEIILKMTAYGAFLHKGSF  LHKGSFCRNYFNILDLLVV  GVQLFKGKLYTCSDSSKQT  QTEAECKGNYITYKDGEVD  AECKGNYITYKDGEVDHPI  TFEGWPELLYRSIDSHTED  SHTEDKGPIYNYRVEISIF  TEDKGPIYNYRVEISIFFI  VEISIFFIIYIIIIAFFMM  TFQEQGEQEYKNCELDKNQ  DKNQRQCVEYALKARPLRR  ALKARPLRRYIPKNQHQYK  RYIPKNQHQYKVWYVVNST  KNQHQYKVWYVVNSTYFEY  KVWYVVNSTYFEYLMFVLI  YVVNSTYFEYLMFVLILLN  NTICLAMQHYGQSCLFKIA  KLIAFKPKGYFSDPWNVFD  DVILSETNHYFCDAWNTFD  FIKSFQALPYVALLIVMLF  LLIVMLFFIYAVIGMQVFG  PCGSSFAVFYFISFYMLCA  FAVFYFISFYMLCAFLIIN  VAVIMDNFDYLTRDWSILG  DEFKRIWAEYDPEAKGRIK  DDEVTVGKFYATFLIQEYF  FYATFLIQEYFRKFKKRKE  GGLFGNHVSYYQSDGRSAF  GLFGNHVSYYQSDGRSAFP  VDSTFTPSSYSSTGSNANI  LGRLPRPAGYPSTVSTVEG  QEETSQDETYEVKMNHDTE  SLLSTEMLSYQDDENRQLT  QGISAEPQTYRSFHDLRQA  QIQEIVDCMYPVEYGKDSC  IVDCMYPVEYGKDSCIIKE  KEGDVGSLVYVMEDGKVEV  KVFGELAILYNCTRTATVK  RTGLIKHTEYMEFLKSVPT  LADVLEETHYENGEYIIRQ  EETHYENGEYIIRQGARGD  GLDDVSNKAYEDAEAKAKY  AHSDFIVRLYRTFKDSKYL  LYRTFKDSKYLYMLMEACL  RTFKDSKYLYMLMEACLGG  SFEDSTTRFYTACVVEAFA  TACVVEAFAYLHSKGIIYR  AYLHSKGIIYRDLKPENLI  ENLILDHRGYAKLVDFGFA  TWTFCGTPEYVAPEIILNK  NKGHDISADYWSLGILMYE  FSGPDPMKTYNIILRGIDM  GEEEKEFISYNINIDIHYG  SYNINIDIHYGVKSNSLAF  VLTLSEDSPYETLHSFISN  NAVAPFFKSYIRESGKADR  MITNVAKQCYERGEKPKVT  FWLNLERALYRIQEKRESP  KQALETVNDYNPLMKDFPL  HLRKIRNTKYPIQRALRLV  GTRKLMHVAYEEFEKVMVA  EVFQTWDDEYEKLQVLLRD  NAIEEVNLAYENVKEVDGL  EAWEAAMKRYDERIDRVET  PHIRGAIREYQTQLIQRVK  KQIDRQLTAYMKRVEDVLG  NKAHQANQLYPFAISLIES  ISLIESVRTYERTCEKVEE  EGIALVWESYKLDPYVQRL  VWESYKLDPYVQRLAETVF  EVRSLETCMYDHKTFSEIL  AVDDLNLHSYSNLPIWVNK  ELRITNQVIYLNPPIEECR  LNPPIEECRYKLYQEMFAW  PIEECRYKLYQEMFAWKMV  SLPRIQSQRYQVGVHYELT  SQRYQVGVHYELTEEEKFY  YELTEEEKFYRNALTRMPD  DGPVALEESYSAVMGIVSE  MGIVSEVEQYVKVWLQYQC  EQYVKVWLQYQCLWDMQAE  LWDMQAENIYNRLGEDLNK  KEFGPVVIDYGKVQSKVNL  KVQSKVNLKYDSWHKEVLS  TSDAVTFITYVQSLKRKIK  KQFEKQVELYRNGQRLLEK  RFQFPPSWLYIDNIEGEWG  EEALQALTIYEGKFGRLKD  KSFPARLRQYASYEFVQRL  PARLRQYASYEFVQRLLKG  EFVQRLLKGYMKINMLVIE  KQIREVWNTYELDLVNYQN  NTYELDLVNYQNKCRLIRG  SVSAMKLSPYYKVFEEDAL  VSAMKLSPYYKVFEEDALS  WIDVQRRWVYLEGIFTGSA  GKIQKALGEYLERERSSFP  RERSSFPRFYFVGDEDLLE  KATSIDPNTYITWIDKYQA  NTYITWIDKYQAQLVVLSA  FEWLSQMRFYFDPKQTDVL  IQMANAKFNYGFEYLGVQD  NAKFNYGFEYLGVQDKLVQ  VQTPLTDRCYLTMTQALEA  ALREHSNPNYDKTSAPITC  AIFITMNPGYAGRSNLPDN  RQLIAQVMLYSQGFRTAEV  DEQLSSQSHYDFGLRALKS  LSDVFPGVQYHRGEMTALR  ELKKVCQEMYLTYGDGEEV  KVCQEMYLTYGDGEEVGGM  MWVEKVLQLYQITQINHGL  PKAISKDHLYGTLDPNTRE  IMFEVQDLKYATLATVSRC  RDAATIMQPYFTSNGLVTK  HQACRNVAQYNANHPDFPM  PMQIEQLERYIQRYLVYAI  EQLERYIQRYLVYAILWSL  ERYIQRYLVYAILWSLSGD  LKMRAELGEYIRRITTVPL  TAPNIPIIDYEVSISGEWS  DTVRHEALLYTWLAEHKPL  ELLLKTFDHYCEYRRTPNG  LKTFDHYCEYRRTPNGVVL  EINLPDMDKYGTQRVISFI  RQMVEHGGFYRTSDQTWVK  RFLRHVPVVYVDYPGPASL  RHVPVVYVDYPGPASLTQI  PGPASLTQIYGTFNRAMLR  LRLIPSLRTYAEPLTAAMV  PLTAAMVEFYTMSQERFTQ  RFTQDTQPHYIYSPREMTR  TQDTQPHYIYSPREMTRWV  EKAMSRPILYSNWLSKDYI  LYSNWLSKDYIPVDQEELR  PVDQEELRDYVKARLKVFY  YVKARLKVFYEEELDVPLV  VAWMNGLSVYQIKVHRKYT  VYQIKVHRKYTGEDFDEDL  VPGLFEGDEYATLMTQCKE  LMLDSHEELYKWFTSQVIR  FGDWSTEALYQVGKEFTSK  SKMDLEKPNYIVPDYMPVV  EKPNYIVPDYMPVVYDKLP  IVPDYMPVVYDKLPQPPSH  RTMAITPRHYLDFINHYAN  RHYLDFINHYANLFHEKRS  AIREKMKKNYMSNPSYNYE  KKNYMSNPSYNYEIVNRAS  NYMSNPSYNYEIVNRASLA  VKWAIAQLNYADMLKRVEP  RDLEASIARYKEEYAVLIS  ASIARYKEEYAVLISEAQA  CLLSAAFIAYAGYFDQQMR  SAAFIAYAGYFDQQMRQNL  FRTDIARTEYLSNADERLR  AIMLKRFNRYPLIIDPSGQ  QATEFIMNEYKDRKITRTS  QEVETVSQQYLPLSTACSS  PLSTACSSIYFTMESLKQI  ESLKQIHFLYQYSLQFFLD  LKQIHFLYQYSLQFFLDIY  YSLQFFLDIYHNVLYENPN  FLDIYHNVLYENPNLKGVT  LKGTVGEPTYDAEFQHFLR  SSSPEQTVPYLWSEETPAT  PVLMCSVPGYDASGHVEDL  KSPNERARLYFLLAWFHAI  HAIIQERLRYAPLGWSKKY  YAPLGWSKKYEFGESDLRS  LKTLMAQSIYGGRVDNEFD  MLEDEDDLAYAETEKKTRT  CEGKKKQTNYLRTLINELV  GILPRSWSHYTVPAGMTVI  LGGLFVPEAYITATRQYVA  EAYITATRQYVAQANSWSL  KASVVTLPVYLNFTRADLI  ATKEDPRSFYERGVAVLCT  FQSSDGEIKYLQLAEELIR  LSTTIQEEFYRVYPYLCRA  TIQEEFYRVYPYLCRALKT  QEEFYRVYPYLCRALKTFV  KEIPLAKDFYVAFQDLPTR  IRDVEQQFKYTQPNICRNP  NSRVSGVDGYETEGIRGLR  RALGVRDLSYRLVFLACCV  FEMSQDKNLYHNLCTSLFP  VEEFSPRAVYTSGKASSAA  AAANPISGHYDRSKSLKQN  VDECNEVTDYAIARRIVDL  RIEESIDRVYSLDDIRRYL  VYSLDDIRRYLLFARQFKP  PAPVNGINGYNEDINQESA  ASLRLGFSEYCRISNLIVL  LKRSELVNWYLKEIESEID  EKVIHRLTHYDHVLIELTQ  KGSTEGSESYEEDPYLVVN  GSESYEEDPYLVVNPNYLL  DPYLVVNPNYLLEDXXXXX  LIEIENFKSYKGRQIIGPF  ANRAFVSMVYSEEGAEDRT  NNKVVQLHEYSEELEKLGI  SRSGELAQEYDKRKKEMVK  KAEEDTQFNYHRKKNIAAE  KQEKEEADRYQRLKDEVVR  QVQLQLFKLYHNEVEIEKL  SELNQKRPQYIKAKENTSH  KSLQNAQKHYKKRKGDMDE  TLEENQVKKYHRLKEEASK  QKRIEKLEEYITTSKQSLE  EIMESIKRLYPGSVYGRLI  IKRLYPGSVYGRLIDLCQP  DLCQPTQKKYQIAVTKVLG  EKTGRDCIQYIKEQRGEPE  AKLVIDVIRYEPPHIKKAL  PPHIKKALQYACGNALVCD  AHGLQMRLKYSQSDLEQTK  SGSQRISSIYAREALIEID  AREALIEIDYGDLCEDLKD  SVATNIDEIYKALSRNSSA  LGPENPEEPYLDGINYNCV  EEPYLDGINYNCVAPGKRF  LALLFAIHSYKPAPFFVLD  NTNIGKVANYIKEQSTCNF  IVISLKEEFYTKAESLIGV  TKAESLIGVYPEQGDCVIS  KVLTFDLTKYPDANPNPNE  XXMALHSPQYIFGDFSPDE  PRSSVELPPYSGTVLCGTQ  VDKLPDGQEYQRIEFGVDE  SDTLPRTPSYSISSTLNPQ  PDGITKEASYGSIDCQYPG  ASYGSIDCQYPGSALALDG  KKKKKRPPGYYSYLKDGGD  KKKKRPPGYYSYLKDGGDD  KKRPPGYYSYLKDGGDDSI  KPSSSSPVAYVETKYSPPA  SPVAYVETKYSPPAISPLV  GLINKGNWCYINATLQALV  QALVACPPMYHLMKFIPLY  YHLMKFIPLYSKVQRPCTS  RPGAAFEPTYIYRLLTVNK  GAAFEPTYIYRLLTVNKSS  KGRQEDAEEYLGFILNGLH  LVARESVQGYTTKTKQEVE  LVLHLKRFVYEKTGGCQKL  CQKLIKNIEYPVDLEISKE  NKNFKCHRTYRLFAVVYHH  RTYRLFAVVYHHGNSATGG  HGNSATGGHYTTDVFQIGL  DQTVKVINQYQVVKPTAER  VKPTAERTAYLLYYRRVDL  TAERTAYLLYYRRVDLLXX  AERTAYLLYYRRVDLLXXX  NCARCGKIVYPTEKVNCLD  CKMTLNMKNYKGYEKKPYC  TLNMKNYKGYEKKPYCNAH  NYKGYEKKPYCNAHYPKQS  EKKPYCNAHYPKQSFTMVA  QSELQSQVRYKEEFEKNKG  TQDQISNIKYHEEFEKSRM  RRDSQDGSSYRRPLEQQQP  HHIPTSAPVYQQPQQQPVA  PQQQPVAQSYGGYKEPAAP  QPVAQSYGGYKEPAAPVSI  SAPGGGGKRYRAVYDYSAA  GGGKRYRAVYDYSAADEDE  GKRYRAVYDYSAADEDEVS  VQQIDDGWMYGTVERTGDT  QFINPETPGYVGFANLPNQ  INSLFLTDLYPERVIPGAA  RLTVVDTPGYGDAINCRDC  RDCFKTIISYIDEQFERYL  IDNRVHCCFYFISPFGHGL  EIEEHNIKIYHLPDAESDE  KGKKVRGRLYPWGVVEVEN  LQEVTQDLHYENFRSERLK  XXXMADFDTYDDRAYSSFG  QKELPTEPPYTAYVGNLPF  LPTEPPYTAYVGNLPFNTV  VFLKNEKGQYISPFHDIPI  ISPFHDIPIYADKDVFHMV  QDVKKGKLRYVANLFPYKG  LRYVANLFPYKGYIWNYGA  VANLFPYKGYIWNYGAIPQ  FPYKGYIWNYGAIPQTWED  NDVKRLKPGYLEATVDWFR  EATVDWFRRYKVPDGKPEN  QDRCSDAAGYPHATHDLEG  DLEGPPLDAYSIQGQHTIS  DSSSPEVKGYWASLDASTQ  SAASISLAQYLINARLSSE  XXXXXXMFLYNLTLQRATG  FRLTGGTKDYIVVGSDSGR  DSGRIVILEYQPSKNMFEK  SAIEKQKLVYILNRDAAAR  EAHKANTLVYHVVGVDVGF  PMFACLEMDYEEADNDPTG  ANTQQTLTFYELDLGLNHV  LGLNHVVRKYSEPLEEHGN  SGVLICSENYITYKNFGDQ  LICSENYITYKNFGDQPDI  DMVTEIRLKYFDTVPVAAA  FVASEFGNHYLYQIAHLGD  ASEFGNHYLYQIAHLGDDD  LANEDTPQLYVACGRGPRS  RHIEDEFDAYIIVSFVNAT  LGDDALVQVYPDGIRHIRA  IALTGGELVYFEMDPSGQL  MDPSGQLNEYTERKEMSAD  GERGSIGFLYLNIGLQNGV  GDLSDTRTRYLGSRPVKLF  AMSSRSWLSYSYQSRFHLT  SSRSWLSYSYQSRFHLTPL  SRFHLTPLSYETLEFASGF  FNQVAFPLQYTPRKFVIHP  IIIETDHNAYTEATKAQRK  RFSNTGEDWYVLVGVAKDL  PRSVAGGFVYTYKLVNNGE  SVAGGFVYTYKLVNNGEKL  IGVGKLLRVYDLGKKKLLR  KCENKHIANYISGIQTIGH  VQESFIWVRYKRNENQLII  WVTTASLLDYDTVAGADKF  SQKAEVIMNYHVGETVLSL  IPGGSESLVYTTLSGGIGI  GRDHLSFRSYYFPVKNVID  RDHLSFRSYYFPVKNVIDG  KKLEDIRTRYAFXXXXXXX  DRVTGQHQGYGFVEFLSEE  EFLSEEDADYAIKIMNMIK  IKIMNMIKLYGKPIRVNKA  DPEIDEKLLYDTFSAFGVI  DPDTGNSKGYAFINFASFD  AAIEAMNGQYLCNRPITVS  LCNRPITVSYAFKKDSKGE  PPPLMPPHGYTGPPRPPPY  YTGPPRPPPYGYQRGPLPP  GPPRPPPYGYQRGPLPPPR  ETIDINKDPYFMKNHLGSY  LTLHNNEGSYLAHTQGKKH  KFVKIGRPGYKVTKQRDSE  QQSLLFQIDYPEIAEGIMP  IEPPDRRWQYLLMAAEPYE  QYLLMAAEPYETIAFKVPS  FNFLNPNDPYHAYYRHKVS  LNPNDPYHAYYRHKVSEFK  NPNDPYHAYYRHKVSEFKE  QLMQKEQRNYQFDFLRPQH  LRPQHSLFNYFTKLVEQYT  NYFTKLVEQYTKILIPPKG  PREVLDQVCYRVEWAKFQE  EEKEKERVAYAQIDWHDFV  GARILIQERYEKFGESEEV  PDQVIVRKDYDPKASKPLP  LPPAPAPDEYLVSPITGEK  MPAGKQKLQYEGIFIKDSN  FIKDSNSLAYYNMANGAVI  IKDSNSLAYYNMANGAVIH  IWNVKKQSVYLMNLRKSGT  HPGSLDETTYERLAEETLD  FFEDLADKPYTFEDYDVSF  ADKPYTFEDYDVSFGSGVL  SSPSSGPKRYDWTGKNWVY  XXXXXXMSRYGRYGGETKV  XXXMSRYGRYGGETKVYVG  KGELERAFSYYGPLRTVWI  GELERAFSYYGPLRTVWIA  RPFDPNDRCYECGEKGHYA  CYECGEKGHYAYDCHRYSR  ECGEKGHYAYDCHRYSRRR  GHYAYDCHRYSRRRRSRSR  SHSRSRGRRYSRSRSRSRG  RSGSIKGSRYFQSPSRSRS  DLGSIPWLGYALDFGKDAA  IFTILVGGRYVTVLLDPHS  VTVLLDPHSYDAVVWEPRT  PRTRLDFHAYAIFLMERIF  RIFDVQLPHYSPSDEKARM  ELQALTEAMYTNLHAVLLG  HEMGLLDFSYSFLLRAGYL  SYSFLLRAGYLTLYGIEAL  LLRAGYLTLYGIEALPRTH  AHRSKWLESYLLHLEEMGV  LSPQRDPEIYTDPEVFKYN  IYTDPEVFKYNRFLNPDGS  PDGSEKKDFYKDGKRLKNY  GHNHCLGRSYAVNSIKQFV  EIPEFDLSRYGFGLMQPEH  QPEHDVPVRYRIRPXXXXX  TVALREIRRYQKSTELLIR  MALQEACESYLVGLFEDTN  GGGIGGTILYAKWDSHFRE  RESVEKTIPYSDKLFEMVL  FEMVLGPAAYNVPLPKKSI  QSEAKVVSQYHELVVQARD  NFTLDINTAYARLRGIEQA  LWLSVEALKYSMKTSSAET  PPESLTRGVYSEETLRARF  SEETLRARFYAVQKLARRV  MIDETRNSLYQYFLSYLQS  DETRNSLYQYFLSYLQSLL  NSLYQYFLSYLQSLLLFPP  DINTFKLLSYASYCIEHGD  TFKLLSYASYCIEHGDLEL  KQIVEILTAYASAVGIGTT  LQGEVVAFDYQSKMLALKC  ADILLINLQYVSEVEIIND  EKEEKLSQAYAISAGVSLE  MEEVVITPPYQVENCKGKE  GFLKEMACPYSVLISGDIK  SQLDKNSEVYQEVQAMFDT  RINDALSCEYECRRRMLMK  VKTDDIARIYQPKRYALSP  IARIYQPKRYALSPKTTIT  TVENELMISYLMFLQILVY  YLMFLQILVYFSFMSWXXX  LMSLIINTFYSNKEIFLWE  IAKFQDQTEYLEEMQVKEV  VEKHSQFLGYPITLYLEKE  QFLGYPITLYLEKEREKEI  NTEDITQEEYGEFYKSLTN  ITQEEYGEFYKSLTNDWKD  DWKDHLAVRYFSVEEYVSR  AVRYFSVEEYVSRMKEIQK  MKEIQKSIYYITGESKEQV  VWKRDSRVVYMTEPIDGYQ  VYMTEPIDGYQLKEFDGKS  SSCCIVTSTYSWTANMEQI  EAVREGWGVYVTPRAPIRE  NGGSSDAPAYRTPPSRQGR  RFSDEPPEVYGDFEPLVAK  AKEEVRESAYYLRSRQRRQ  KEEVRESAYYLRSRQRRQP  IRLRRPPLRYPRYEATSVQ  DKTTRSSSQYIESFWQSSQ  KQPSVLSSGYQKTPQEWAP  NQMNQLKNKYQGQDEKLWK  CLSEQIADAYSSFRSVRAI  FPAGSTLIFYKYCDHENAA  AGSTLIFYKYCDHENAAFK  FTNSNTPNSYNHMDPDKLN  XXXMAGVFPYRGPGNPVPG  PGPLAPLPDYMSEEKLQEK  KWQQLQAKRYAEKRKFGFV  RKFRHDKRVYLGALKYMPH  KRVYLGALKYMPHAVLKLL  EQIRDVPVLYHITGAISFV  EIPWVIEPVYISQWGSMWI  FDDEEPPLDYADNILDVEP  EDAPVLDWFYDHQPLRDSR  HQPLRDSRKYVNGSTYQRW  SRKYVNGSTYQRWQFTLPM  FTLPMMSTLYRLANQLLTD  LLTDLVDDNYFYLFDLKAF  TDLVDDNYFYLFDLKAFFT  IIRQPIRTEYKIAFPYLYN  RTEYKIAFPYLYNNLPHHV  EYKIAFPYLYNNLPHHVHL  LPHHVHLTWYHTPNVVFIK  TEDPDLPAFYFDPLINPIS  EPFLKDTPLYTDNTANGIA  LDIPLVKNWYREHCPAGQP  GQPVKVRVSYQKLLKYYVL  RVSYQKLLKYYVLNALKHR  VSYQKLLKYYVLNALKHRP  RPPKAQKKRYLFRSFKATK  VGLQVCRQGYNMLNLLIHR  LLIHRKNLNYLHLDYNFNL  KNLNYLHLDYNFNLKPVKT  KLVVDSHVQYRLGNVDAFQ  AFQLADGLQYIFAHVGQLT  AHVGQLTGMYRYKYKLMRQ  VGQLTGMYRYKYKLMRQIR  QLTGMYRYKYKLMRQIRMC  MCKDLKHLIYYRFNTGPVG  CKDLKHLIYYRFNTGPVGK  TPIENMILRYVKAKADWWT  ADWWTNTAHYNRERIRRGA  KNLGRLTRLYLKAEQERQH  KAEQERQHNYLKDGPYITA  QHNYLKDGPYITAEEAVAV  ITAEEAVAVYTTTVHWLES  SPIPFPPLSYKHDTKLLIL  LALERLKEAYSVKSRLNQS  EELGLIEQAYDNPHEALSR  EVGIEFMDLYSHLVPVYDV  DLYSHLVPVYDVEPLEKIT  EPLEKITDAYLDQYLWYEA  KITDAYLDQYLWYEADKRR  DAYLDQYLWYEADKRRLFP  DTEPPPLLVYKWCQGINNL  MLESRFEKMYEKIDLTLLN  LIVDHNIADYMTAKNNVVI  TAKNNVVINYKDMNHTNSY  YKDMNHTNSYGIIRGLQFA  LQFASFIVQYYGLVMDLLV  QFASFIVQYYGLVMDLLVL  AHPIRLFCRYIDRIHIFFR  DEARDLIQRYLTEHPDPNN  DPNNENIVGYNNKKCWPRD  QWENSFVSVYSKDNPNLLF  NTALIGLMTYFREAVVNTQ  PSRFPPVVFYTPKELGGLG  EEDQLIPNLYRYIQPWESE  DQLIPNLYRYIQPWESEFI  IDSQRVWAEYALKRQEAIA  FQKDRHTLAYDKGWRVRTD  WRVRTDFKQYQVLKQNPFW  DGKLWNLNNYRTDMIQALG  LEHTLFKGTYFPTWEGLFW  SPTINRANVYVGFQVQLDL  KETIHPRKSYKMNSSCADI  CADILLFASYKWNVSRPSL  VMDSTTTQKYWIDIQLRWG  IDIQLRWGDYDSHDIERYA  DYDSHDIERYARAKFLDYT  RYARAKFLDYTTDNMSIYP  DYTTDNMSIYPSPTGVLIA  GVLIAIDLAYNLHSAYGNW  DLAYNLHSAYGNWFPGSKP  KIMKANPALYVLRERIRKG  ERIRKGLQLYSSEPTEPYL  LYSSEPTEPYLSSQNYGEL  TEPYLSSQNYGELFSNQII  IWFVDDTNVYRVTIHKTFE  EPQMVLFNLYDDWLKTISS  DDWLKTISSYTAFSRLILI  QLKDLILADYGKKNNVNVA  EIITSTTSNYETQTFSSKT  NLHLRTNHIYVSSDDIKET  SSDDIKETGYTYILPKNVL  DDIKETGYTYILPKNVLKK  SDLRAQIAGYLYGVSPPDN  LRAQIAGYLYGVSPPDNPQ  LPGQLPQHEYLKEMEPLGW  TPGSCTLTAYKLTPSGYEW  TAYKLTPSGYEWGRQNTDK  TDKGNNPKGYLPSHYERVQ  NPKGYLPSHYERVQMLLSD  MVPAQSSWNYNFMGVRHDP  GVRHDPNMKYELQLANPKE  LQLANPKEFYHEVHRPSHF  FALLQEGEVYSADREDLYA  VYSADREDLYAXXXXXXXX  QIGNACWELYCLEHGIQPD  KKSKLEFSIYPAPQVSTAV  QVSTAVVEPYNSILTTHTT  AFMVDNEAIYDICRRNLDI  VKCDPRHGKYMACCLLYRG  GKYMACCLLYRGDVVPKDV  RLDHKFDLMYAKRAFVHWY  YAKRAFVHWYVGEGMEEGE  EDMAALEKDYEEVGVDSVE  FMDVKCPGCYKITTVFSHA  ENKTPQGREYGMIYLGKDT  PQGREYGMIYLGKDTNGEN  GSHTIRDLKYTIENPRHFV  VVRALLLPDYYLVTVMLSG  VRALLLPDYYLVTVMLSGI  RCVDWSIAVYTRGAEKLRA  IVVKLNSGDYKTIHLSSIR  DKNKKLRPLYDIPYMFEAR  KLRPLYDIPYMFEAREFLR  GKKVNVTVDYIRPASPATE  SKGLATVIRYRQDDDQRSS  QDDDQRSSHYDELLAAEAR  AGRSEAVVEYVFSGSRLKL  VFSGSRLKLYLPKETCLIT  VHFTAERSSYYKSLLSAEE  HFTAERSSYYKSLLSAEEA  QKKEKVWAHYEEQPVEEVM  LEEKERSASYKPVFVTEIT  TEITDDLHFYVQDVETGTQ  ASHPPVEGSYAPRRGEFCI  IAKFVDGEWYRARVEKVES  ESPAKIHVFYIDYGNREVL  AKIHVFYIDYGNREVLPST  RVLPAQATEYAFAFIQVPQ  KQFQKVITEYLNAQESAKS  KSARLNLWRYGDFRADDAD  RADDADEFGYSRXXXXXXX  XXMGRESRHYRKRSASRGR  GEELDPLDAYMEEVKEEVK  MENDQDAMEYSSEEEEVDL  VDLQTALTGYQTKQRKLLE  EPVDHGKIEYEPFRKNFYV  EYEPFRKNFYVEVPELAKM  ILNSLKKHGYEKPTPIQTQ  TLGLRVVCVYGGTGISEQI  RVTNLRRVTYVVLDEADRM  LKLLELLGHYQESGSVIIF  LLKDLMRASYPCMSLHGGI  MSLHGGIDQYDRDSIINDF  VKHLILVVNYSCPNHYEDY  VVNYSCPNHYEDYVHRAGR  YSCPNHYEDYVHRAGRTGR  RTGRAGNKGYAYTFITEDQ  GRAGNKGYAYTFITEDQAR  TFITEDQARYAGDIIKALE  AEKINAKLNYVPLEKQEEE  GGQNESFKRYEEELEINDF  KEALQRISEYSEAAITIRG  EAAITIRGTYFPPGKEPKE  EPKEGERKIYLAIESANEL  SYQPTNKGRYKVLXXXXXX  XXXXMASASYHISNLLEKM  GPLVSKVKEYQVETIVDTL  NDSMSTTRTYIQCIAAISR  RQAGHRIGEYLEKIIPLVV  NVDDDELREYCIQAFESFV  FVRRCPKEVYPHVSTIINI  STIINICLKYLTYDPNYNY  INICLKYLTYDPNYNYDDE  LKYLTYDPNYNYDDEDEDE  YLTYDPNYNYDDEDEDENA  DDDQGSDDEYSDDDDMSWK  TRHEMLPEFYKTVSPALIS  NVKADVFHAYLSLLKQTRP  LKIDALSCLYVILCNHSPQ  VVACVGDPFYKITSEALLV  QPSSFDATPYIKDLFTCTI  SALDILIKNYSDSLTAAMI  VVTGTNNLGYMDLLRMLTG  LLRMLTGPVYSQSTALTHK  STALTHKQSYYSIAKCVAA  TALTHKQSYYSIAKCVAAL  SEEVKSAASYALGSISVGN  SISVGNLPEYLPFVLQEIT  EITSQPKRQYLLLHSLKEI  SASVVGLKPYVENIWALLL  ETLLPRLKGYLISGSSYAR  KGYLISGSSYARSSVVTAV  LLDTVLPHLYNETKVRKEL  IRKAAFECMYTLLDSCLDR  HVEDGLKDHYDIKMLTFLM  EGPSPGDFRYSDGTPVNYT  RYSDGTPVNYTNWYRGEPA  GTPVNYTNWYRGEPAGRGK  RGKEQCVEMYTDGQWNDRN  VPGAIMDEDYYGSAAEWGD  PGAIMDEDYYGSAAEWGDE  PSIFNTLKRYFQAGGSPEN  NVIQLLSENYTAVAQTVNL  AHTTWRDLFYKLAEAHPDC  TVKLISDAGYQGEITSVST  KMVCHGEHTYLFAQAMMSV  TLALGTAASYPRACQALGA  RINQDHKHKYIHILAYAAS  KHKYIHILAYAASVVETWK  ELVAELSTLYQCIRFPVVA  VDWTVSEPRYFQLQTDHTP  RMVHLLSRGYVLPVVSYIR  RGYVLPVVSYIRKCLEKLD  LDTDISLIRYFVTEVLDVI  EVLDVIAPPYTSDFVQLFL  SEGVDLIDIYADEEFNQDP  FNNTDQIDLYDDVLTATSQ  PNNKTPAILYTYSGLRNRR  NKTPAILYTYSGLRNRRAA  GLRNRRAAVYVGSFSWWTT  IQVIRSIGVYDVVELKFAE  NRANGQSKGYAEVVVASEN  VDKPPSVLPYFNRPPSALP  VPPPPPGIHYQHLMPPPPR  ATVGPPPDTYMKASAPYNH  DTYMKASAPYNHHGSRDSG  AVSGASAGDYSDAIETLLT  LHNEDRHDDYFQERNREHE  GQETQEYRLYREKKTALWI  SITWEHAGRYRCYYGSDTA  PVSPSRRWWYRCYAYDSNS  PSRRWWYRCYAYDSNSPYE  RRWWYRCYAYDSNSPYEWS  CYAYDSNSPYEWSLPSDLL  DAGYNRFVLYKDGERDFLQ  FTLGPVSRSYGGQYRCYGA  PVSRSYGGQYRCYGAHNLS  RSYGGQYRCYGAHNLSSEW  LDILIAGQFYDRVSLSVQP  DDPWRLRSTYQSQKYQAEF  PVTSAHAGTYRCYGSQSSK  SAHAGTYRCYGSQSSKPYL  AADAQEENLYAAVKHTQPE  HDEDPQAVTYAEVKHSRPR  ASEAPQDVTYAQLHSLTLR  GPSPAVPSIYATLAIHXXX  GELLKQADLYISEGLHPRI  SVLAVRRPGYPIDLFMVEI  ILICNVSLEYEKTEVNSGF  DCLGHAGLVYEYTLGEEKF  LGHAGLVYEYTLGEEKFTF  VAMAEALVTYKNSIKGRAR  PKVLAQNAGYDPQETLVKV  AADAGVWDNYCVKKQLLHS  XXXXMSVEAYGPSSQTLTF  NFEEDEEDTYYTKDLPIHA  FEEDEEDTYYTKDLPIHAC  KDLPIHACSYCGIHDPACV  GIHDPACVVYCNTSKKWFC  PLGETVLECYNCGCRNVFL  EEPQHVLLRYEDAYQYQNI  HVLLRYEDAYQYQNIFGPL  LLRYEDAYQYQNIFGPLVK  GPLVKLEADYDKKLKESQT  LGLNKKRIAYFTLPKTDSG  MQGDEICLRYKGDLAPLWK  GHVIKVPDNYGDEIAIELR  AVDETSVSGYIYHKLLGHE  DETSVSGYIYHKLLGHEVE  LPDLNHSQVYAVKTVLQRP  KTVTSATIVYHLARQGNGP  ELSSADEKRYRALKRTAER  IRPIRLQVQYRMHPALSAF  LSAFPSNIFYEGSLQNGVT  PQPDKPMFFYVTQGQEEIA  EEIASSGTSYLNRTEAANV  PDQIGIITPYEGQRSYLVQ  ITPYEGQRSYLVQYMQFSG  EGQRSYLVQYMQFSGSLHT  FSGSLHTKLYQEVEIASVD  LNVALTRARYGVIIVGNPK  QPLWNHLLNYYKEQKVLVE  PLWNHLLNYYKEQKVLVEG  REAIIPGSVYDRSSQGRPS  SSQGRPSSMYFQTHDQIGM  VMPPMPPPGYFGQANGPAA  FSQGALTQGYISMSQPSQM  SQPELSQDSYLGDEFKSQI  DVALSQDSTYQGERAYQHG  DSTYQGERAYQHGGVTGLS  HGGVTGLSQYXXXXXXXXX  AAAAAGLAVYRRKDGGPAT  SVRVWLGKHYKKYVHADAP  ALCHILGAAYKYKNEQGWR  CHILGAAYKYKNEQGWRRF  NNCLTRPNIYLIPDIDLKL  EKSKASHHIYPYSSSQDDE  SKASHHIYPYSSSQDDEEW  KQVLVHWGFYPDSYDTWVH  VHWGFYPDSYDTWVHSNDV  FNEWMNEEDYEVDENRKPV  SGKKGQASLYGKRRSQKEE  QTNHIIIPSYASWFDYNCI  IPSYASWFDYNCIHVIERR  KNKSKTPEIYLAYRNFMID  SKTPEIYLAYRNFMIDTYR  AYRNFMIDTYRLNPQEYLT  DTYRLNPQEYLTSTACRRN  FLEQWGLVNYQVDPESRPM  QNFGLRTDIYSKKTLAKSK  LLLLEALEMYKDDWNKVSE  FLRLPIEDPYLENSDASLG  SDASLGPLAYQPVPFSQSG  RASGKVDPTYGLESSCIAG  QNFHMEQLKYAELRARQQM  MMPHQQPPPYPLMHHQMPP  VPLTAPNGMYPPPPQQQPP  XXXXXXMADYSTVPPPSSG  AGTSLNSNDYGYGGQKRPL  TSLNSNDYGYGGQKRPLED  QSRSVMTEEYKVPDGMVGF  KPLRITGDPYKVQQAKEMV  GGFREVRNEYGSRIGGNEG  PGPGTPMGPYNPAPYNPGP  PMGPYNPAPYNPGPPGPAP  PAPHGPPAPYAPQGWGNAY  YAPQGWGNAYPHWQQQAPP  DPNSAAWAAYYAHYYQQQA  PNSAAWAAYYAHYYQQQAQ  AAWAAYYAHYYQQQAQPPP  AWAAYYAHYYQQQAQPPPA  NPAPAGQVDYTKAWEEYYK  VDYTKAWEEYYKKMGQAVP  DYTKAWEEYYKKMGQAVPA  GAPPGGQPDYSAAWAEYYR  PDYSAAWAEYYRQQAAYYA  DYSAAWAEYYRQQAAYYAQ  AEYYRQQAAYYAQTSPQGM  EYYRQQAAYYAQTSPQGMP  QYQFTGIKKYFNSYTLTGR  TGIKKYFNSYTLTGRMNCV  GRMNCVLATYGSIALIVLY  YGSIALIVLYFKLRSKKTP  EELAALFAPYGTVMSCAVM  VIECDVVKDYAFVHMEKEA  TGGFSATFDYQQAFGNSTG  LRRSPPRASYVAPLTAQPA  APLTAQPATYRAQPSVSLG  QPSVSLGAAYRAQPSASLG  QPSASLGVGYRTQPMTAQA  QPSVSLGAPYRGQLASPSS  SAAASSLGPYGGAQPSASA  QPSASALSSYGGQAAAASS  AAAASSLNSYGAQGSSLAS  GAQGSSLASYGNQPSSYGA  ASYGNQPSSYGAQAASSYG  SYGAQAASSYGVRAAASSY  YGVRAAASSYNTQGAASSL  QGAASSLGSYGAQAASYGA  GSYGAQAASYGAQSAASSL  AQSAASSLAYGAQAASYNA  LAYGAQAASYNAQPSASYN  SYNAQPSASYNAQSAPYAA  ASYNAQSAPYAAQQAASYS  PYAAQQAASYSSQPAAYVA  ASYSSQPAAYVAQPATAAA  VAQPATAAAYASQPAAYAA  AAYASQPAAYAAQATTPMA  QATTPMAGSYGAQPVVQTQ  PVVQTQLNSYGAQASMGLS  QASMGLSGSYGAQSAAAAT  QSAAAATGSYGAAAAYGAQ  TGSYGAAAAYGAQPSATLA  QPSATLAAPYRTQSSASLA  QSSASLAASYAAQQHPQAA  QQHPQAAASYRGQPGNAYD  SYRGQPGNAYDGAGQPSAA  DGAGQPSAAYLSMSQGAVA  ANANSTPPPYERTRLSPPR  TRLSPPRASYDDPYKKAVA  PPRASYDDPYKKAVAMSKR  KKAVAMSKRYGSDRRLAEL  DRRLAELSDYRRLSESQLS  RSPTKSSLDYRRLPDAHSD  RRLPDAHSDYARYSGSYND  PDAHSDYARYSGSYNDYLR  SDYARYSGSYNDYLRAAQM  ARYSGSYNDYLRAAQMHSG  LRAAQMHSGYQRRMXXXXX  RLDIQNKRFYLDVKQNAKG  DSLGDFIEHYAQLGPSSPE  EFLVRENRKYYLDLKENQR  FLVRENRKYYLDLKENQRG  DALAKLIDDYGGEDDELAG  GAGGPGGGLYGELPEGTSI  FFFDVGCNKYGVFLRVSEV  GKFGGAFCRYADEMKEIQE  IQERQRDKLYERRGGGSGG  VNSSELDEEYLIELEKNMS  GNEQFKKGDYIEAESSYSR  GDYIEAESSYSRALEMCPS  RAILRRAELYEKTDKLDEA  DKLDEALEDYKSILEKDPS  IKQDSSTGSYSINFVQNPN  PADTEKMLRYEIKQIKMFK  GFEKVKDVQYIYTPFDSSL  EKVKDVQYIYTPFDSSLCG  GKVFIHLCNYIEPWEDLSL  VQRESLNHHYHLNCGCQIT  NCGCQITTCYTVPCTISAP  KHVDGTCSWYRGHLPLRKE  AEFLKQVKPYVEEGLHPQI  AGFEMQPKKYHNPKIALLN  EIRVHTVEDYQAIVDAEWN  IVDAEWNILYDKLEKIHHS  LPIGDVATQYFADRDMFCA  EETQIGGERYNFFTGCPKA  GAIEMELSKYLRDYSRTIP  MELSKYLRDYSRTIPGKQQ  GKQQLLIGAYAKALEIIPR  ARHAQGGTWYGVDINNEDI  LQPHAEPGGYSGPDGHYAM  GGYSGPDGHYAMDNITRQN  DNITRQNQFYDTQVIKQEN  VIKQENESGYERRPLEMEQ  PLEMEQQQAYRPEMKTEMK  QQFQSRKRPYEENRGRGYF  PYEENRGRGYFEHREDRRG  DDTLVAIDTYNCDLHFKVA  KVARDRSSGYPLTIEGFAY  YPLTIEGFAYLWSGARASY  YLWSGARASYGVRRGRVCF  TQLGEEPFSYGYGGTGKKS  LGEEPFSYGYGGTGKKSTN  KSTNSRFENYGDKFAENDV  KEALGGQALYPHVLVKNCA  FNFGQRAEPYCSVLPGFTF  HAASNPSKKYNILGTNAIM  VMGLRRQRNYAGRWDVLIQ  QIAARKKRNYILDQTNVYG  NYILDQTNVYGSAQRRKMR  EEADKLVRQYNEEGRKAGP  RGGGGGFQRYENRGPPGGN  GGGSGGGGNYRGGFNRSGG  GFNRSGGGGYSQNRWGNNN  NNNSNNRGSYNRAPQQQPP  PQQPPPPPSYSPARNPPGA  NTSTPTVSSYSPPQPSYSQ  SSYSPPQPSYSQPPYNQGG  PQPSYSQPPYNQGGYSQGY  SQPPYNQGGYSQGYTAPPP  YNQGGYSQGYTAPPPPPPP  PPPPPPPPAYNYGSYGGYN  PPPPPPAYNYGSYGGYNPA  PPPAYNYGSYGGYNPAPYT  AYNYGSYGGYNPAPYTPPP  SYGGYNPAPYTPPPPPTAQ  PPPPPTAQTYPQPSYNQYQ  TAQTYPQPSYNQYQQYAQQ  TYPQPSYNQYQQYAQQWNQ  QPSYNQYQQYAQQWNQYYQ  QQYAQQWNQYYQNQGQWPP  QYAQQWNQYYQNQGQWPPY  YQNQGQWPPYYGNYDYGSY  QNQGQWPPYYGNYDYGSYS  GQWPPYYGNYDYGSYSGNT  WPPYYGNYDYGSYSGNTQG  YYGNYDYGSYSGNTQGGTS  XXXXXMTDRYTIHSQLEHL  WLVNQHRDSYCSYMGHFDL  NQHRDSYCSYMGHFDLLNY  YMGHFDLLNYFAIAENESK  DEVNFLVYRYLQESGFSHS  ISIIQKGLQYVEAEVSINE  DVVQTRQQAYRDKLAQQQA  EGTLLATGSYDGFARIWTK  DLQAHNKEIYTIKWSPTGP  TLTKHQEPVYSVAFSPDGR  SVAFSPDGRYLASGSFDKC  TQTGALVHSYRGTGGIFEV  KLSVNAPEFYPSGYSSSYT  NAPEFYPSGYSSSYTESYE  FYPSGYSSSYTESYEDGCE  GYSSSYTESYEDGCEDYPT  ESYEDGCEDYPTLSEYVQD  CEDYPTLSEYVQDFLNHLT  ALQELVELIYQQATSIPNF  QATSIPNFSYMGARLCNYL  SYMGARLCNYLSHHLTISP  LLLQRCRTEYEVKDQAAKG  AFVLFLGELYLNLEIKGTN  EATPENDPNYFMNEPTFYT  NYFMNEPTFYTSDGVPFTA  VPFTAADPDYQEKYQELLE  AADPDYQEKYQELLEREDF  LEREDFFPDYEENGTDLSG  TDLSGAGDPYLDDIDDEMD  EMDPEIEEAYEKFCLESER  AFFRWLSRKYPSIIVNCVE  PNDVEFDNLYLDMNGIIHP  DEMMVAIFEYIDRLFSIVR  SIVRPRRLLYMAIDGVAPR  MDNLAKCLRYYIADRLNND  DNLAKCLRYYIADRLNNDP  GEGEHKIMDYIRRQRAQPN  FLRLNVLREYLERELTMAS  NAIDRLVNIYKNVVHKTGG  KNVVHKTGGYLTESGYVNL  TGGYLTESGYVNLQRVQMI  VASNPRQAAYEMRMQNNSS  LWEAGWKQRYYKNKFDVDA  WEAGWKQRYYKNKFDVDAA  KFRRKVVQSYVEGLCWVLR  VEGLCWVLRYYYQGCASWK  EGLCWVLRYYYQGCASWKW  GLCWVLRYYYQGCASWKWY  YQGCASWKWYYPFHYAPFA  QGCASWKWYYPFHYAPFAS  SWKWYYPFHYAPFASDFEG  DPDSSIIDFYPEDFAIDLN  FAIDLNGKKYAWQGVALLP  RLRAALEEVYPDLTPEETR  PLHDFILELYQTGSTEPVE  MPRGSGTGIYSNAAPPPVT  SNAAPPPVTYQGNLYRPLL  PPVTYQGNLYRPLLRGQAQ  QNAAFQPNQYQMLAGPGGY  YQMLAGPGGYPPRRDDRGG  RDDRGGRQGYPREGRKYPL  QGYPREGRKYPLPPPSGRY  YPLPPPSGRYNWNXXXXXX  QWLLTPSREYATKTRIGIR  AAVGLGALCYYGLGLSNEI  AVGLGALCYYGLGLSNEIG  IEKAVIWPQYVKDRIHSTY  YVKDRIHSTYMYLAGSIGL  KDRIHSTYMYLAGSIGLTA  AGMLVRSIPYDQSPGPKHL  GPLLIRAAWYTAGIVGGLS  PTTVAGATLYSVAMYGGLV  GATLYSVAMYGGLVLFSMF  LVLFSMFLLYDTQKVIKRA  VSPMYGVQKYDPINSMLSI  DPINSMLSIYMDTLNIFMR  VFAQADGSAYIEQGNTKAL  GNTKALAVVYGPHEIRGSR  PDRALVNCQYSSATFSTGE  LHPRSQIDIYVQVLQADGG  QVLQADGGTYAACVNAATL  IQAKTFHHVYSGKDLIAQA  TKKLSVACFYGGTPYGGQF  VACFYGGTPYGGQFERMRN  QVEEILSVAYKKDSEDNPQ  HWVFNVAKKYMKSTYEQVD  VAKKYMKSTYEQVDLIGKK  SSPPKDVESYIHRSGRTGR  GRTGVCICFYQHKEEYQLV  ICFYQHKEEYQLVQVEQKA  CSIEMPNISYAWKELKEQL  PELEGPREGYGGFRGQREG  TSKHSWGKTYGEHKRHLEF  RHLEFSHDQYRELQRYAEE  HDQYRELQRYAEEVGIFFT  GSGDTNNFPYLEKTAKKGR  QSMDTMKQVYQIVKPLNPN  FCFLQCTSAYPLQPEDVNL  DVNLRVISEYQKLFPDIPI  KLFPDIPIGYSGHETGIAI  TVKVGEPKGYPPEDIFNLV  XXXXXMAAQYGSMSFNPST  FNPSTPGASYGPGRQEPRN  LLLVVPLGRYTEEEHKATE  DLGDTNLHDYLREAPEDIQ  DLMDIFGDRYCALNNKATG  VVRENKEGCYTNRMYQRAE  KEGCYTNRMYQRAEEEIQK  REKARIREEYEEKIRKLED  SRSHSRKKRYSSRSRSRTY  YSSRSRSRTYSRSRSRDRM  SRSRSRDRMYSRDYRRDYR  SRDRMYSRDYRRDYRNNRG  MYSRDYRRDYRNNRGMRRP  RNNRGMRRPYGYRGRGRGY  NRGMRRPYGYRGRGRGYYQ  YGYRGRGRGYYQGGGGRYH  GYRGRGRGYYQGGGGRYHR  GYYQGGGGRYHRGGYRPVW  GGGRYHRGGYRPVWNRRHS  RSRSRSRRSYRSSRSPRSS  SSSSRSSSPYSKSPVSKRR  GDIWPGLSAYDNSPRSPHS  GSVGNGSSRYSPSQNSPIH  EWEDQEALDYFSDKESGKQ  GDDTEETEDYRQFRKSVLA  RNTEEEGLKYKSKVSLKGN  SDGFREEKNYKLKETGYVV  KNYKLKETGYVVERPSTTK  SEKLKDLFDYSPPLHKNLD  SLVHHVKEQYFKSAAMTLN  MTLNERFTSYQKATEEHST  RKQEKTPKDYKEYKSYKDD  EKTPKDYKEYKSYKDDSKH  PKDYKEYKSYKDDSKHKRE  FKTHHEMKEYSGFAGVSRP  PKEEEWDPEYTPKSKKYFL  PEYTPKSKKYFLHDDRDDG  HDDRDDGVDYWAKRGRGRG  SSPKWTHDKYQGDGIVEDE  MPKPVRVVQYNINTEELYS  QYNINTEELYSYLKEFIHI  NINTEELYSYLKEFIHILY  YLKEFIHILYFRHLLVNPR  ETLTRVLFKYFEVPSVLLA  NSAMVLDCGYRESLVLPIY  YRESLVLPIYEGIPVLNCW  RPSPPPNVDYPLDGEKILH  LHRLLAEIRYLVEKPKYKK  IRYLVEKPKYKKALGTKTF  LGSRSVSKEYYNQTGRIPD  GSRSVSKEYYNQTGRIPDW  LEKQTSKGKYFVTFPYPYM  KGKYFVTFPYPYMNGRLHL  KYFVTFPYPYMNGRLHLGH  LSKCEFAVGYQRLKGKCCL  DKLKREIELYGCPPDFPDE  AAAKAGSSKYQWGIMKSLG  FSEAEHWLDYFPPLAIQDL  SFITTDVNPYYDSFVRWQF  FITTDVNPYYDSFVRWQFL  RNKIKFGKRYTIYSPKDGQ  IKFGKRYTIYSPKDGQPCM  TGEGVGPQEYTLLKLKVLE  LLKLKVLEPYPSKLSGLKG  NCWVRPDMKYIGFETVNGD  ASLSAPLTSYKVIYVLPML  APLTSYKVIYVLPMLTIKE  KKKQALRAKYGIRDDMVLP  KLAEAKEKIYLKGFYEGIM  KEKIYLKGFYEGIMLVDGF  MIDAGDALIYMEPEKQVMS  CVVALCDQWYLDYGEENWK  ALCDQWYLDYGEENWKKQT  LQEHACSRTYGLGTHLPWD  IESLSDSTIYMAFYTVAHL  SDSTIYMAFYTVAHLLQGG  QQMTKEVWDYVFFKEAPFP  QLKQEFEFWYPVDLRVSGK  KDLVPNHLSYYLYNHVAMW  DLVPNHLSYYLYNHVAMWP  VPNHLSYYLYNHVAMWPEQ  MADAGILRLYTWVEWVKEM  AGIIKTDQNYEKMMFKEAL  FEFQAAKDKYRELAVEGMH  NEVLIHSSQYLMEVTHDLR  HDLRLRLKNYMMPAKGKKT  LQKPSHCTIYVAKNYPPWQ  HCTIYVAKNYPPWQHTTLS  LGSMPELKKYMKKVMPFVA  KAVLMENIVYLTNSLELEH  RRVPVLGKEYTEKTPISEH  RVDIGDTIIYLVHXXXXXX  GLRHGDFQRYRGYCSRRQR  HGDFQRYRGYCSRRQRRLR  TEELLTDNRYLLLVLMDAE  LMDAERAWSYAMQLKQEAN  AKTKLEAQAYTAYLSGMLR  KLEAQAYTAYLSGMLRFEH  EAFNKCKTIYEKLASAFTE  AFTEEQAVLYNQRVEEISP  VEEISPNIRYCAYNIGDQS  ISPNIRYCAYNIGDQSAIN  EPGKVSNLQYLHSYLTYIK  VSNLQYLHSYLTYIKLSTA  LQYLHSYLTYIKLSTAIKR  PRPQDLIRLYDIILQNLVE  GLKTLVFKAYRCFFIAQSY  YRCFFIAQSYVLVKKWSEA  KKWSEALVLYDRVLKYANE  LVLYDRVLKYANEVNSDAG  QKTKSGLTGYIKGIFGFRS  XXXXMALLCYNRGCGQRFD  ETNSDDACTYHPGVPVFHD  CKNGGCSKTYQGLESLEEV  LESLEEVCVYHSGVPIFHE  VPIFHEGMKYWSCCRRKTS  TGGEVTISVYAKNSLPELS  RQQVPTRKDYGSKVSLFSH  VSLFSHLPQYSRQNSLTQF  PAMVRLGLQYSQGLVSGSN  RALQQVIQDYTTPPNEELS  RDLVNKLKPYMSFLTQCRP  SELRAAIDRYVQEKIVLAA  AAQAISRFAYQKISNGDVI  ISNGDVILVYGCSSLVSRI  LVHAGVPASYLLIPAASYV  SYLLIPAASYVLPEVSKVL  VPVLVCCETYKFCERVQTD  ASLRLLNLVYDVTPPELVD  TESSQNGKQYIISEELISE  IPVLQRTLHYECIVLVKQF  KQFRPPMGGYCIEFPAGLI  LRELEEETGYKGDIAECSP  EHLTVDARVYSYALALKHA  LTVDARVYSYALALKHANA  PEPAMTSGVYRPPGARLTT  KTPQGPPEIYSDTQFPSLQ  ALKLQLDNQYAVLENQKSS  ENQKSSHSQYNXXXXXXXX  LFQEAEEFLYRFLPQKIIY  YRFLPQKIIYLNQLLQEDS  EAFQTTISKYFSERGDAVA  LVHERDEAAYGELRAMVLD  AMVLDLRAFYAELYHIISS  DLRAFYAELYHIISSNLEK  PKGEEKPSMYXXXXXXXXX  LVGREFVRQYYTLLNKAPE  VGREFVRQYYTLLNKAPEY  KAPEYLHRFYGRNSSYVHG  HRFYGRNSSYVHGGVDASG  ASGKPQEAVYGQNDIHHKV  PEGSVPNKFYVHNDMFRYE  FYVHNDMFRYEDEVFGDSE  PVQENANSGYYEAHPVTNG  VQENANSGYYEAHPVTNGI  DSDNRRIIRYPDSHQLFVG  DLQIALASFYEDGGDEDIV  EEEEEGQRFYAGGSERSGQ  KPRPFAGGGYRLGAAPEEE  SLDNGELRSYQDPSNAQFL  LKTTEMETIYDLGTKMIES  GRSFTRARDYDAMGSQTKF  GITRIRGTSYQSPHGIPID  RLLIVSTTPYSEKDTKQIL  EDVEMSEDAYTVLTRIGLE  RIGLETSLRYAIQLITAAS  FLDESRSTQYMKEYQDAFL  SRSTQYMKEYQDAFLFNEL  LLAEVTLKTYWVSKGSAFS  SWRDRPFKPYNFLAHGVLP  VKRTHSQGGYGSQGYKYNW  SQGGYGSQGYKYNWKLDEA  GGYGSQGYKYNWKLDEARK  TTSASARALYRLAQKKPFT  QKKPFTPVKYFSIDRVFRN  ITQLRFKPAYNPYTEPSME  LRFKPAYNPYTEPSMEVFS  TEPSMEVFSYHQGLKKWVE  SLERPTMIKYGINNIRELV  GHKVNLQMVYDSPLCRLDA  LSSRSRSRSYSPAHNRERN  NRERNHPRVYQNRDFRGHN  RDFRGHNRGYRRPYYFRGR  GHNRGYRRPYYFRGRNRGF  HNRGYRRPYYFRGRNRGFY  YFRGRNRGFYPWGQYNRGG  NRGFYPWGQYNRGGYGNYR  PWGQYNRGGYGNYRSNWQN  QYNRGGYGNYRSNWQNYRQ  GNYRSNWQNYRQAYSPRRG  SNWQNYRQAYSPRRGRSRS  SSKPWPDATYGTGSASRAS  GSTLPSGAGYQSGTHQGQF  KSPPSTGSTYGSSQKEESA  ESAASGGAAYTKRYLEEQK  SGGAAYTKRYLEEQKTENG  FRGSQSPKRYKLRDDFEKK  KSGKWEGLVYAPPGKEKQR  PEKNFRVTAYKAVQEKSSS  MTLHERFTKYLKRGTEQEA  EMKSPREPGYKAEGKYKDD  SSSSQSSHSYKAEEYTEET  SSHSYKAEEYTEETEEREE  RGRGWGRGNYSGNNNNNSN  NREEEWDPEYTPKSKKYYL  PEYTPKSKKYYLHDDREGE  EYTPKSKKYYLHDDREGEG  ETFERILRLYEKENPDIYK  LYEKENPDIYKELRKLEVE  KELRKLEVEYEQKRAQLSQ  PPPPQVVQMYGRKVGFALD  PRRRDEDMLYSPELAQRGH  VSSTSEDDGYPEDMDQDKH  VSVQTKDDVYEAFMKEMEG  GKNCTAGAVYTYHEKKKDT  NCTAGAVYTYHEKKKDTAA  HDPVVTPDGYLYEREAILE  PVVTPDGYLYEREAILEYI  LYEREAILEYILHQKKEIA  KEIARQMKAYEKQRGTRRE  VGLITRSERYVCAVTRDSL  DLAFSGITPYGYFLISACK  AFSGITPYGYFLISACKDG  GGQDKLLRIYDLNKPEAEP  FNMSVSSMEYIPEGEILVI  PEGEILVITYGRSIAFHSA  VAGGEDFKLYKYDYNSGEE  GGEDFKLYKYDYNSGEELE  EDFKLYKYDYNSGEELESY  YNSGEELESYKGHFGPIHC  VRFSPDGELYASGSEDGTL  LWQTVVGKTYGLWKCVLPE  IREVCGFAPYERRAMELLK  FQSGFLSILYSIGSKPLQI  IEGTNVSTTYITCPADPKK  VMIIKNLKKYFTFEVQVLD  VRRRFRASNYQSTTRVKPF  NLLDFTRRAYGTNYIETLR  FTRRAYGTNYIETLRVQIH  HANCRIRRVYFSDRLYSED  RRVYFSDRLYSEDELPAEF  LLGPFQKDLYRDVMLEIYS  IYSNLLSMGYQVSKPDALS  QRMLKSVEQYHEHNAFGNT  RENHDTFELYIKTLKSNLS  DGKSFLHGNYEELYSAAKF  FLHGNYEELYSAAKFSVST  ERVHTGEKPYGCTLCAKVF  QRTHTGEKPYICNECGKGF  QRNHTGEKSYICSECGKGF  QRTHTGEKPYICSECGKGF  CGKGFTTKHYVIIHQRNHT  QRNHTGEKPYICNECGKGF  QRTHTGEKPYICSECGKGF  QRNHTVEKSYLCSECGKGF  QRTHTGEKPYTCSECGKGF  QRTHTGEKPYRCSECGKGF  QRTHTGEKPYICNECGKGF  CGKGFTMKRYLIVHQQIHT  KQVHTGEKPYGCNECGKGF  LLMLKAFEPYLEILEVYST  EPYLEILEVYSTKAKNYVN  EVYSTKAKNYVNGHCTKYE  NYVNGHCTKYEPWQLIAWS  VWTLLIVWGYEFVFQPESL  MSFLKVDKEYVKALPSQGL  SAVLEKLKEYSSMDAFWQE  QEGRASGTVYSGEEKLTEL  KLTELLVKAYGDFAWSNPL  ESILMACKAYRDLAFEKGI  HAAFNKAASYFGMKIVRVP  PEVAKLAVKYKIPLHVDAC  LIVFMEKAGYPLEHPFDFR  TSISADTHKYGYAPKGSSL  SLVLYSDKKYRNYQFFVDT  LYSDKKYRNYQFFVDTDWQ  VDTDWQGGIYASPTIAGSR  ALMHFGENGYVEATKQIIK  ALGSRDFDIYRLSNLMTAK  AKTTGMGAIYGMAQTTVDR  LSSVFLDSLYSTDTVTQGS  XXXXMAERGYSFSLTTFSP  PSGKLVQIEYALAAVAGGA  TEKKQKSILYDERSVHKVE  PITKHIGLVYSGMGPDYRV  LVYSGMGPDYRVLVHRARK  HRARKLAQQYYLVYQEPIP  RARKLAQQYYLVYQEPIPT  KLAQQYYLVYQEPIPTAQL  QRVASVMQEYTQSGGVRPF  ICGWNEGRPYLFQSDPSGA  LFQSDPSGAYFAWKATAMG  WKATAMGKNYVNGKTFLEK  NGKTFLEKRYNEDLELEDA  RRGGVSAEVYTEEDAVSYV  VYTEEDAVSYVRKVIPKDY  YVRKVIPKDYKTMTALAKA  QQGNEGDNFYVVDQGEVDV  VVDQGEVDVYVNGEWVTNI  GSFGELALIYGTPRAATVK  KLWGIDRDSYRRILMGSTL  GSTLRKRKMYEEFLSKVSI  VQGEPGDDFYIITEGTASV  LQRRSPNEEYVEVGRLGPS  EVGRLGPSDYFGEIALLLN  EILKRNIQRYNSFISLTVX  KLNPRLAILYAKRASVFVK  QKIAEHRRKYERKREEREI  EARRQSGAQYGSFPGGFPG  AQNPANMSKYQSNPKVMNL  TKKVGIVGKYGTRYGASLR  GIVGKYGTRYGASLRKMVK  KTVAGGAWTYNTTSAVTVK  KNKEPRPPSYTISIVGLSG  RFVRSKADEYYPEHTSVLS  FVRSKADEYYPEHTSVLST  RVVNNDHFLYWGDIIQNSE  PHRSTNLQPYIKRAAASKL  KLQSAEKLMYICTDQLGLE  TKCDECVDHYLREVQAFAS  TRSKPKIIPYLDAYKTQRQ  PKIIPYLDAYKTQRQLVVT  EKLVQTVRDYHATWKTVSN  SNKLKNHPDYEEYINLEGT  LKNHPDYEEYINLEGTRKA  EHIRKRREEYINTLPRAFN  LSTLEAEKVYQNHVQHLIS  MCFVMEDEAYKYITEADSK  FVMEDEAYKYITEADSKEV  ITEADSKEVYGRHQREIVE  MLFEHSELFYDLDLNATPS  HTVLSEEPRYKALQKLAPD  QLDHGRLRLYHDSTNIDKV  IRTQSTDDEYALDGKIYEL  DEYALDGKIYELDLRPVDA  LRPVDAKSPYFLSQLWTAA  EASQIRKDKYMANLPFTLI  PFVDVPAGTYPRKFNETQI  RRIQITILSYHSSIGVRKD  VRKDELVHGYILVYSAKRK  ELVHGYILVYSAKRKASMG  EITAKFTALYSLSQYHRQT  FTALYSLSQYHRQTEVFTL  EKKNMIENSYLSDNTREST  LPSPRDCFPYNNYPDSDDD  PRDCFPYNNYPDSDDDTEA  DDDTEAPPPYSPIGDDVQL  LPTPSDRSRYRLDLEGNEY  YRLDLEGNEYPIHSTPNCH  PEDMDPSDNYAEPIDTIFK  IDTIFKQKGYSDEIYVVPD  KQKGYSDEIYVVPDDSQNR  SHGERRPSKYKYKSKTLFS  GERRPSKYKYKSKTLFSKA  KTLFSKAKSYYRRTHSDAS  TLFSKAKSYYRRTHSDASD  PTRRNWESNYFGMPLQDLV  DTGLCTEGLYRVSGNKTDQ  ADLPDPLIPYSLHPELLEA  IVKKFHPVNYDVFRYVITH  HPVNYDVFRYVITHLNRVS  FIQQCQFFFYNGEIVETTN  TGLLESGRHYLAASRAFVV  AALRTARAGYRGRALDYAL  AGYRGRALDYALQINVIED  HEELSRLSQYRKELGAQLH  FTIQSNQLVYQKKYKDPVT  SNQLVYQKKYKDPVTVVVD  EKEAWIHAKYVEKKFLTKL  AQGQAGDETYLDIFRDFSL  FLEPTSEAKYIPPDLCVCN  QGGGHRTLLYGHAVLLRHS  LLRHSFSGMYLTCLTTSRS  ILVSVSSERYLHLSVSNGN  CSGSSIEEGYLLGGHVVRL  NDSQHRRIFYEAGGAGTRA  RLRHLTTGHYLALTEDQGL  EGMGVPEIKYGDSVCFVQH  HIASGLWVTYKAQDAKTSR  LQTLQDLIAYFQPPEEEMR  LNCIDRLNVYNSVAHFAGI  AWKEILNLLYKLLAALIRG  LGVAEGSAQYKKWYFELII  EGSAQYKKWYFELIIDQVD  RVGWASSSGYAPYPGGGEG  WASSSGYAPYPGGGEGWGG  GGNGVGDDLYSYGFDGLHL  NGVGDDLYSYGFDGLHLWS  EFKFLPPSGYAPCYEALLP  LPPSGYAPCYEALLPKEKM  KMRLEPVKEYKRDADGIRD  SKLPETEKNYNLQMSTETL  LKKVKLPKNYMMSNGYKPA  PKNYMMSNGYKPAPLDLSD  KDRIKQGWTYGIQQDLKNK  NKRNPRLVPYALLDERTKK  REAVRTFVGYGYNIEPSDQ  AVRTFVGYGYNIEPSDQEL  IRFFRVERSYAVRSGKWYF  SYAVRSGKWYFEFEVVTGG  GQRWHQGSGYFGRTWQPGD  KGSELAFADYEIENGFVPI  GTDASTFKFYTMCGLQEGF  TQNSNADMIYCRLSMPVEC  ILSHTTTQCYYAIRIFAGQ  LSHTTTQCYYAIRIFAGQD  VWVGWVTPDYHLYSEKFDL  GWVTPDYHLYSEKFDLNKN  HESVKRSNCYMVWGGDIVA  ANGKELGTCYQVEPNTKVF  EQEDLMRFHYHTLRLYSAV  RFHYHTLRLYSAVCALGNS  CALGNSRVAYALCSHVDLS  SHVDLSQLFYAIDNKYLPG  QLFYAIDNKYLPGLLRSGF  LPGLLRSGFYDLLISIHLA  ERKLMMKNEYIIPITSTTR  KLQMCELLSYLCDCELQHR  EAIVAFGDIYVSKLQANQK  KLQANQKFRYNELMQALNM  CPEEIREELYDFHEDLLLH  WTGKLCALVYKIKGPPKPE  MMFNLLRRQYDSIGELLQA  ELLQALRKTYTISHTSVSD  GDIMNNKVFYQHPNLMRVL  VASCCRFLCYFCRISRQNQ  QKAMFEHLSYLLENSSVGL  EPDLEKVVTYLAGCGLQSC  SCPMLLAKGYPDVGWNPIE  GWNPIEGERYLSFLRFAVF  PALDLPSQGYKREVSTGDD  HMGNAIMSFYSALIDLLGR  PMVLFLDRVYGIKDQTFLL  TEAALALNRYICSAVLPLL  LIDSTLQTIYRLSKGRSLT  VFDVPQLNEYCKMPLKLLT  MPLKLLTNHYEQCWKYYCL  TNHYEQCWKYYCLPSGWGS  NHYEQCWKYYCLPSGWGSY  YCLPSGWGSYGLAVEEELH  IFDSLSHKKYDPDLFRMAL  NFSLPEKLEYIVTKYAEHS  EKLEYIVTKYAEHSHDKWA  CDKSQSGWKYGISLDENVK  TLTEKEKEIYRWPARESLK  VSQANQGNSYSPAPLDLSN  GMVEVVAENYHNIWAKKKK  GGSHPLLVPYDTLTAKEKF  ASSMEKRFAYKFLKKILKY  YKFLKKILKYVDSAQEFIA  KVLLPLVDQYFTSHCLYFL  DQYFTSHCLYFLSSPLKPL  PLKPLSSSGYASHKEKEMV  IKGVSQNINYTTVALLPIL  LLGDVQISCYHILCSLYSL  SCYHILCSLYSLGTGKNIY  YSLGTGKNIYVERQRPALG  AFLEPTLNRYNPLSVFNTK  NDLAESGARYTEMPHVIEV  EVILPMLCNYLSYWWERGP  LPMLCNYLSYWWERGPENL  ASWMKRIAVYAQPIISKAR  EFAVLCRDLYAFYPMLIRY  VLCRDLYAFYPMLIRYVDN  YAFYPMLIRYVDNNRSNWL  KKTKRRGDLYSIQTSLIVA  ELISLAKSRYSHRDTDEEV  PAVKWQLNLYKDVLKSEEP  VACFRMAPLYNLPRHRSIN  RSINLFLHGYQRFWIETEE  QRFWIETEEYSFEEKLVQD  PDPLHQIILYFSRNALTER  RSKLEDDPLYTSYSSMMAK  LEDDPLYTSYSSMMAKSCQ  KEMEKQKTLYQQARLHERG  AGVQQKMLDYLKEKKDAGF  VNVIISTVDYLLRLQESIS  RLQESISDFYWYYSGKDII  QESISDFYWYYSGKDIIDE  ESISDFYWYYSGKDIIDES  KQIFNSLTEYIQGPCIGNQ  LTSSDTFKEYDPDGKGIIS  QKAMEGQKQYTQSEIDFLL  EADENDMFNYVDFVDRFHE  LDPAESVLNYFEPYLGRIE  ESVLNYFEPYLGRIEIMGG  GGAKKIERVYFEISESSRT  EEEEDEDSSYVLEIAGEEE  ATLKNLRKQYRNVKKMTAK  KDKEEEQAEYLWTEVTKKK  ANFFKGLEIYQTKLLHYLA  EIYQTKLLHYLARNFYNLR  LLHYLARNFYNLRFLALFV  FAINFILLFYKVTEEPLEE  FFVLQESTGYMAPTLRALA  IISLVCVVGYYCLKVPLVV  ISLVCVVGYYCLKVPLVVF  ARKLEFDGLYITEQPSEDD  INTPSFPNNYWDKFVKRKV  FVKRKVINKYGDLYGAERI  KVINKYGDLYGAERIAELL  SWLSSIDMKYHIWKLGVVF  VVFTDNSFLYLAWYTTMSV  DNSFLYLAWYTTMSVLGHY  YTTMSVLGHYNNFFFAAHL  TVGLLAVVVYLYTVVAFNF  GLLAVVVYLYTVVAFNFFR  VAFNFFRKFYNKSEDDDEP  MKCDDMMTCYLFHMYVGVR  MMTCYLFHMYVGVRAGGGI  EIEDPAGDPYEMYRIVFDI  DPAGDPYEMYRIVFDITFF  CFICGIGNDYFDTTPHGFE  TLQEHNLANYLFFLMYLIN  LANYLFFLMYLINKDETEH  ETEHTGQESYVWKMYQERC  GQESYVWKMYQERCWDFFP  PAGDCFRKQYEDQLGXXXX  QPLEKENDGYFRKGCNPLA  EGLNISVGVYQNTEEAFTI  VLKDFILEHYSEDGYLYED  ILEHYSEDGYLYEDEIADL  EHYSEDGYLYEDEIADLMD  EAGVELLMTYFIQLGFVES  RQMGLLFTWYDSLTGVPVS  SVLFNTGALYTQIGTRCDR  KDTFTHTPSYDMSPAMLSV  QEAAKVGEVYQQLHAAMSQ  QAPVKENIPYSWASLACVK  SLACVKAHHYAALAHYFTA  AHHYAALAHYFTAILLIDH  HQEKCLSQLYDHMPEGLTP  AAQERSRLTYAQHQEEDDL  PVQVHFLDPYCSASVAGAR  SVAGAREGDYIVSIQLVDC  SSMHNKSATYSVGMQKTYS  TYSVGMQKTYSMICLAIDD  SLLNSDSSWYXXXXXXXXX  LEKDRGNPIYLGLNGLNLC  PEPVKSFLFYHSQSGRNST  VTEEGKRHPYKMNLASEPQ  TTARVITNQYNNPAGLYSS  NQYNNPAGLYSSENISNFN  LVIDKESEVYKMLQEKQEL  RDRHRHPECYVCTDCGTNL  GHFFVEDQIYCEKHARERV  PVLQPQPKSYFKSISVTKI  GRIVKMEVDYSATVDQRLP  ILVAVVKMCYEAKEWDLLN  AKMVQQCCTYVEEITDLPI  LRMVTEGKIYVEIERARLT  MRLCLAVKDYIRTQIISKK  ENTEKLKLKYYNLMIQLDQ  NTEKLKLKYYNLMIQLDQH  IQLDQHEGSYLSICKHYRA  GSYLSICKHYRAIYDTPCI  SICKHYRAIYDTPCIQAES  QQALKSVVLYVILAPFDNE  DKKLEEIPKYKDLLKLFTT  MRWSTLVEDYGMELRKGSL  EHNIRIMAKYYTRITMKRM  HNIRIMAKYYTRITMKRMA  GTGATGATLYLLRLALFNP  LGPNDQYKFYSVNVDYSKL  YKFYSVNVDYSKLKKERPD  ENYLIQMARYGQLSEKVSE  VMDSDEDDDYXXXXXXXXX  MLQELNFGAYLGLPAFLLP  WHNFRTLCDYSKRIAVALE  HHSEKEFCSYLQYLEYLSQ  EKEFCSYLQYLEYLSQNRP  FCSYLQYLEYLSQNRPPPN  SQNRPPPNAYELFAKGYED  NAYELFAKGYEDYLQSPLQ  ELFAKGYEDYLQSPLQPLM  LMDNLESQTYEVFEKDPIK  EVFEKDPIKYSQYQQAIYK  EKDPIKYSQYQQAIYKCLL  KYSQYQQAIYKCLLDRVPE  KQADRRIKLYAVEKNPNAV  DDGVSIPGEYTSFLAPISS  LAPISSSKLYNEVRACREK  DPEAQFEMPYVVRLHNFHQ  RDPMIDNNRYCTLEFPVEV  NTVLHGFAGYFETVLYQDI  FAGYFETVLYQDITLSIRP  RCSNSKKVWYEWAVTAPVC  AIHNPTGRSYTIGLXXXXX  LAEGGFAFVYEAQDVGSGR  AQDVGSGREYALKRLLSNE  SCDTVLKIFYQTCRAVQHM  FGSATTISHYPDYSWSAQR  ATTISHYPDYSWSAQRRAL  EITRNTTPMYRTPEIIDLY  YRTPEIIDLYSNFPIGEKQ  DIWALGCILYLLCFRQHPF  AKLRIVNGKYSIPPHDTQY  YSIPPHDTQYTVFHSLIRA  TELLEQNGGYGSATLSRGP  PPVGPAGSGYSGGLALAEY  YSGGLALAEYDQPYGGFLD  LALAEYDQPYGGFLDILRG  SKVIQSVANYAKGDLDISY  YAKGDLDISYITSRIAVMS  FLDSKHPGHYAVYNLSPRT  SKHPGHYAVYNLSPRTYRP  AVYNLSPRTYRPSRFHNRV  RRAPHLHTLYNICRNMHAW  LFSTAEAAVYMFSMKRCPP  PGIWPSHKRYIEYMCDMVA  WPSHKRYIEYMCDMVAEEP  SGCRPFCEVYVGDERVAST  ERVASTSQEYDKMRDFKIE  VQGDVLIVIYHARSTLGGR  NATTVKFAKYDLDACDIQE  LDACDIQEKYPDLFQVNLE  PRQPGSTAQYDAGAGSPEA  KACTQPRPNYASNFSVIGA  VAPEQVKKHYRRAVLAVHP  SVTMEDMNEYSNIEEFAEG  DTSKKDLTEYLSRFGEVVD  DTSEEQIKEYFGAFGEIEN  ERRGFCFITYTDEEPVKKL  IKVAQPKEVYRQQQQQQKG  QNWNQGFNNYYDQGYGNYN  NWNQGFNNYYDQGYGNYNS  GFNNYYDQGYGNYNSAYGG  NYYDQGYGNYNSAYGGDQN  QGYGNYNSAYGGDQNYSGY  NSAYGGDQNYSGYGGYDYT  YGGDQNYSGYGGYDYTGYN  DQNYSGYGGYDYTGYNYGN  NYSGYGGYDYTGYNYGNYG  GYGGYDYTGYNYGNYGYGQ  GGYDYTGYNYGNYGYGQGY  DYTGYNYGNYGYGQGYADY  TGYNYGNYGYGQGYADYSG  YGNYGYGQGYADYSGQQST  YGYGQGYADYSGQQSTYGK  ADYSGQQSTYGKASRGGGN  RGGGNHQNNYQPYXXXXXX  GNHQNNYQPYXXXXXXXXX  RHRLDLGEDYPSGKKRAGT  FTNLPHTPRYYDILKKRLQ  TNLPHTPRYYDILKKRLQL  KRLQLPVWEYKDRFTDILV  TQIPQWCVEYMRSLPGPKR  DVMLGQEVGYSIRFEDCSS  CSSAKTILKYMTDGMLLRE  AMNDPLLERYGVIILDEAH  TLDAGKFQIYFDNCPLLTI  FYTPEPERDYLEAAIRTVI  VGDIKIIPLYSTLPPQQQQ  DPGFAKQKVYNPRIRVESL  TRPGKCFRLYTEKAYKTEM  CFRLYTEKAYKTEMQDNTY  YKTEMQDNTYPEILRSNLG  LMRALELLNYLAALNDDGD  AKMVIASCDYNCSNEVLSI  GDHLTLLNVYHAFKQNHES  QNHESVQWCYDNFINYRSL  QWCYDNFINYRSLMSADNV  RSTDFTSRDYYINIRKALV  STDFTSRDYYINIRKALVT  NIRKALVTGYFMQVAHLER  VAHLERTGHYLTVKDNQVV  LDHKPEWVLYNEFVLTTKN  NEFVLTTKNYIRTCTDIKP  EWLVKIAPQYYDMSNFPQC  WLVKIAPQYYDMSNFPQCE  IIAKLQSKEYSQYXXXXXX  KLQSKEYSQYXXXXXXXXX  GMQRATNVTYQAHHVSRNK  TTVSMALEEYLVCHGIPCY  YLVCHGIPCYTLDGDNIRQ  VCITSFISPYTQDRNNARQ  CEQRDVKGLYKKARAGEIK  KGFTGIDSEYEKPEAPELV  RDIVPVDASYEVKELYVPE  DASYEVKELYVPENKLHLA  LNGFMREREYLQCLHFDCL  DGCTAFALMYEGRRVAILR  WGTTCKNHPYIKMVMEQGD  GDLQVLDRVYWNDGLDQYR  VYWNDGLDQYRLTPTELKQ  THKQLLERGYRRPVLLLHP  VAIFPSPMMYAGPTEVQWH  ARMVAGANFYIVGRDPAGM  EIVPFRVAAYNKKKKRMDY  YNKKKKRMDYYDSEHHEDF  NKKKKRMDYYDSEHHEDFE  PKAWTVLTEYYKSLEKAXX  KAWTVLTEYYKSLEKAXXX  VKREKRDDGYEAAASSKTS  EFGQRRQDLYSARDLQGLT  VELRKKKPDYLPYAEDESV  RKKKPDYLPYAEDESVDDL  QKPRSILSKYDEELEGERP  CRTLGEIPTYGLAGNREEQ  PNKSLPSAVYCIEDKMAID  EDKMAIDDKYSRREEYRGF  DDKYSRREEYRGFTQDFKE  TQDFKEKDGYKPDVKIEYV  GYKPDVKIEYVDETGRKLT  EKQKAQKTPYIVLSGSGKS  SHNLPHGSTYQMRRPGGEL  GGELPLSKSYSSGNRKGFL  DVLQEARRKYKTIESETVR  VVLHKDSKWYQQWKDFKEN  NRFFEMKMKYDESDNAFIR  ELDILKDWCYEATYSQLAH  LKDWCYEATYSQLAHPIQQ  DPDKVLRMLYVWALCRDQD  CRDQDELNPYAAWRLLDIS  KMAIKLRNEYGPVLHMPTS  ATDSYVHKQYPANQGQEVE  PANQGQEVEYFVAGTHPYP  EYFVAGTHPYPPGPGVALT  NRTAPSGSEYRHPGASDRP  PQWHPPWKLYRVISGHLGW  GVIVSTRSPYLFSCGEDKQ  KQVKCWDLEYNKVIRHYHG  LEYNKVIRHYHGHLSAVYG  HYHGHLSAVYGLDLHPTID  RAVVLHPRHYTFASGSPDN  MHLWDWRTGYNFQRVHAAV  AEADKTIKVYREDDTATEE  SSWDTSVRLYDVPANSMRL  VPANSMRLKYQHTGAVLDC  TGAVLDCAFYDPTHAWSGG  HDAPIRCVEYCPEVNVMVT  GTFSQPEKVYTLSVSGDRL  QQRRESSLKYQTRCIRAFP  IRAFPNKQGYVLSSIEGRV  PSPEVQKKKYAFKCHRLKE  LKENNIEQIYPVNAISFHN  KKRLCQFHRYPTSIASLAF  GTTLAIASSYMYEMDDTEH  TLAIASSYMYEMDDTEHPE  EHGGPPELFYDETEARKYV  FYDETEARKYVRNSRMIDI  MAGRALELLYLPENKPCYL  LYLPENKPCYLLDIGCGTG  GCGTGLSGSYLSDEGHYWV  GSYLSDEGHYWVGLDISPA  KSENPAKRLYCFFASLFSV  RGSRAVLQLYPENSEQLEL  GFSGGMVVDYPNSAKAKKF  PNSAKAKKFYLCLFSGPST  DEQLRMQELYGDGKDGDTQ  LGQQPTDTPYEWDLDKKAW  KITEDFIATYQANYGFSND  DFIATYQANYGFSNDGASS  VEEDRNTNVYVSGLPPDIT  QTEEFKVKLYKDNQGNLKG  NLKGDGLCCYLKRESVELA  LLDEDEIRGYKLHVEVAKF  VAKFQLKGEYDASKKKKKC  SKKKKKCKDYKKKLSMQQK  AQAWDGTTDYQVEETSRER  DEKEEEEDTYEKVFDDESD  ESDEKEDEEYADEKGLEAA  XMDNKKRLAYAIIQFLHDQ  ENFEAAVHFYGKAIELNPA  IELNPANAVYFCNRAAAYS  VYFCNRAAAYSKLGNYAGA  AAAYSKLGNYAGAVQDCER  ERAICIDPAYSKAYGRMGL  CIDPAYSKAYGRMGLALSS  LNKHVEAVAYYKKALELDP  NKHVEAVAYYKKALELDPD  XXXMVLAELYVSDREGSDA  CVKIGALEGYRGQRVKVFG  FLVLRDGTGYLQCVLADEL  VLADELCQCYNGVLLSTES  LSTESSVAVYGMLNLTPKG  FRDHFFDRGYYEVTPPTLV  RDHFFDRGYYEVTPPTLVQ  GGATLFKLDYFGEEAFLTQ  AFLTQSSQLYLETCLPALG  GDVFCIAQSYRAEQSRTRR  SRTRRHLAEYTHVEAECPF  PKRPFKRMNYSDAIVWLKE  DVKKEDGTFYEFGEDIPEA  RFPVEIKSFYMQRCPEDSR  KREGIDPTPYYWYTDQRKY  REGIDPTPYYWYTDQRKYG  GIDPTPYYWYTDQRKYGTC  YYWYTDQRKYGTCPHGGYG  KYGTCPHGGYGLGLERFLT  RFLTWILNRYHIRDVCLYP  RYHIRDVCLYPRFVQRCTP  VHNDAQSFDYDHDAFLGAE  IKFAQKRWIYEDVERQWKG  EDGLVSWEEYKNATYGYVL  SWEEYKNATYGYVLDDPDP  EEYKNATYGYVLDDPDPDD  DPDPDDGFNYKQMMVRDER  FTAFLHPEEYDYMKDIVVQ  AFLHPEEYDYMKDIVVQET  ADGFIDLEEYIGDMYSHDG  DLEEYIGDMYSHDGNTDEP  TKDWILPSDYDHAEAEARH  LTKEEIVDKYDLFVGSQAT  TIMELLIMVYACKTSCAKS  AKSIIGVIPYFPYSKQCKM  IIGVIPYFPYSKQCKMRKR  LRASPFLLQYIQEEIPDYR  QYIQEEIPDYRNAVIVAKS  AETLKERGAYKIFVMATHG  RIHNGESMSYLFRNIGLDD  KVAEKLDEIYVAGLVAHSD  AFLCGVMKTYRQREKQGTK  IKALLERTGYTLDVTTGQR  LDVTTGQRKYGGPPPDSVY  YGGPPPDSVYSGQQPSVGT  DPLTGLNRGYAFVTFCTKE  EAAQEAVKLYNNHEIRSGK  TEGLTDVILYHQPDDKKKN  KNRGFCFLEYEDHKTAAQA  QRQAAKNQMYDDYYYYGPP  AAKNQMYDDYYYYGPPHMP  AKNQMYDDYYYYGPPHMPP  KNQMYDDYYYYGPPHMPPP  NQMYDDYYYYGPPHMPPPT  GRGRGGRGGYGYPPDYYGY  GRGGRGGYGYPPDYYGYED  RGGYGYPPDYYGYEDYYDY  GGYGYPPDYYGYEDYYDYY  YGYPPDYYGYEDYYDYYGY  PPDYYGYEDYYDYYGYDYH  PDYYGYEDYYDYYGYDYHN  YYGYEDYYDYYGYDYHNYR  YGYEDYYDYYGYDYHNYRG  YEDYYDYYGYDYHNYRGGY  DYYDYYGYDYHNYRGGYED  DYYGYDYHNYRGGYEDPYY  YDYHNYRGGYEDPYYGYED  NYRGGYEDPYYGYEDFQVG  YRGGYEDPYYGYEDFQVGA  GGYEDPYYGYEDFQVGARG  AAPPRGRAGYSQRGGPGSA  VGGKRKADGYNQPDSKRRQ  LQGGDHSGNYGYKSENQEF  GGDHSGNYGYKSENQEFYQ  GYKSENQEFYQDTFGQQWK  LTDRIAAAQYSVTGSAVAR  MGPKKKHLDYLIQATNETN  VHGNERFIQYLASRNTLFN  FLDKSGSHGYDMSTFIRRY  YDMSTFIRRYSRYLNEKAF  STFIRRYSRYLNEKAFSYR  RYLNEKAFSYRQMAFDFAR  KDLIKLFACYNDGVINLLE  MANVADTKLYDILGVPPGA  ASENELKKAYRKLAKEYHP  KAYRKLAKEYHPDKNPNAG  LSNPEKRELYDRYGEQGLR  PEKRELYDRYGEQGLREGS  PLKVSLEDLYNGKTTKLQL  RDGNDLHMTYKIGLVEALC  LDGRQIVVKYPPGKVIEPG  VVRGEGMPQYRNPFEKGDL  RNPFEKGDLYIKFDVQFPE  SGGGQRREAYNDSSDEESS  XXXXXXXMPYEIKKVFASL  GDPKGNNFLYTNGKCVILR  IDNPALADIYTEHAHQVVV  HAHQVVVAKYAPSGFYIAS  VAKYAPSGFYIASGDVSGK  TTQKEHLLKYEYQPFAGKI  QKEHLLKYEYQPFAGKIKD  SVDIKQSRPYRLATGSDDN  ATASADGQIYIYDGKTGEK  ASADGQIYIYDGKTGEKVC  GSKAHDGGIYAISWSPDST  HLLSVSLSGYINYLDRNNP  SVSLSGYINYLDRNNPSKP  TVHKNGGKSYIYSGSHDGH  HKNGGKSYIYSGSHDGHIN  SGSHDGHINYWDSETGEND  SCSMDDTVRYTSLMLRDYS  KCVAVGPGGYAVVVCIGQI  KCFSIDNPGYEPEVVAVHP  GGVDGNVRLYSILGTTLKD  AKGPVTDVAYSHDGAFLAV  VTVFSVADGYSENNVFYGH  DGYSENNVFYGHHAKIVCL  ASGGMDMMVYVWTLSDPET  ASVKEWTITYXXXXXXXXX  KRLVKEKVMYEKEAKQQEE  KMRAEDGENYDIKKQAEIL  DCQRRLEAAYLDLQRILEN  VGQITVDMMYGGMRGMKGL  GMRGMKGLVYETSVLDPDE  YAQGISRTKYWELIYEDSM  SRTKYWELIYEDSMDLIAK  KLPCVAAKIYRNLYREGSG  VAAKIYRNLYREGSGIGAI  SHNFTNMLGYTDHQFTELT  HQFTELTRLYLTIHSDHEG  LVGSALSDPYLSFAAAMNG  DVSDEKLRDYIWNTLNSGR  AVLRKTDPRYTCQREFALK  PMFKLVAQLYKIVPNVLLE  DAHSGVLLQYYGMTEMNYY  AHSGVLLQYYGMTEMNYYT  QYYGMTEMNYYTVLFGVSR  YYGMTEMNYYTVLFGVSRA  LEAGAMTFHYIIEQGVCDL  AFPKTLAFAYLEDLHSEFD  KKVPTVSRPYSFIEFDTFI  TFIQKTKKLYIDSCARRNL  NNLSSLSKKYRQDAKYLNM  SKKYRQDAKYLNMHSTYAK  AKYLNMHSTYAKLAAVAVF  AVFFIMLIVYVRFWWLXXX  RGPVLGPMVYAICYCPLPR  LGPMVYAICYCPLPRLADL  STSMLGRVKYNLNSLSHDT  HDTATGLIQYALDQGVNVT  VDTVGMPETYQARLQQSFP  EKLQDLDTDYGSGYPNDPK  DLDTDYGSGYPNDPKTKAW  QEGLRKITSYFLNEGSQAR  QARPRSSHRYFLERGLESA  SNLMVCNLAYSGKLEELKE  NQNGCTPLHYAASKNRHEI  NLKMIHILLYYKASTNIQD  LKMIHILLYYKASTNIQDT  LLVSQGASIYIENKEEKTP  ENPPAIDWAYYKANVAKAG  NPPAIDWAYYKANVAKAGL  PETKLDKKKYPYWPHQPIE  TKLDKKKYPYWPHQPIENL  KPADMVIEAYGHGQRTFGE  GSTIFGERDYSKKPTPDKC  PDKVQLLSQYDNEKKWELI  FQVKNPPAAYIQKLKSYVD  NRGLDVLLEYLAFAQCSVT  LAFAQCSVTYDMESTDNGA  IMCLRAIMNYQSGFSLVMN  QHRFEKLMEYFRNEDSNID  NMNFRVFLQYEFTHLGLDL  EFTHLGLDLYLERLRLTES  DKLQVQIQAYLDNIFDVGA  ERICQAIEAYDLQALGLDF  LLMRFLPTEYERSLITRFE  LEIVLAFGNYMNSSKRGAA  MNSSKRGAAYGFRLQSLDA  TDRKQTLLHYLVKVIAEKY  YLVKVIAEKYPQLTGFHSD  QEAFESVVEYFGENPKTTS  SLFSRFIKAYKKAEQEVEQ  RRQQKEPLIYESDRDGAIE  CRASESVDNYGISFMNWFQ  PGQPPKLLIYAASNQGSGV  XAKTTAPSVYPLVPVCGGT  VTLGCLVKGYFPEPVTLTW  FPALLQSGLYTLSSSVTVT  AQTQTHREDYNSTLRVVSA  RGPVRAPQVYVLPPPAEEM  TSNGRTEQNYKNTATVLDS  LDSDGSYFMYSKLRVQKST  ENTEENRRFYRQLLLTADD  GVILFHETLYQKADDGRPF  DGLSERCAQYKKDGADFAK  GDHDLKRCQYVTEKVLAAV  VTEKVLAAVYKALSDHHVY  YKALSDHHVYLEGTLLKPN  LKPWALTFSYGRALQASAL  ENLKAAQEEYIKRALANSL  ANSLACQGKYTPSGQSGAA  ESLFISNHAYXXXXXXXXX  RKVNLGVGAYRTDESQPWV  IGADFLGRWYNGTDNKNTP  GTDNKNTPIYVSSPTWENH  AAGFKDIRPYCYWDAEKRG  GFKDIRPYCYWDAEKRGLD  FLFPFFDSAYQGFASGDLE  LEKDAWAIRYFVSEGFELF  QSFSKNFGLYNERVGNLTV  TGLNPKQVEYLVNEKHIYL  EYLVNEKHIYLLPSGRINM  CGLTTKNLDYVATSIHEAV  AYRDDNGKPYVLPSVRKAE  RDAGMQLQGYRYYDPKTCG  AGMQLQGYRYYDPKTCGFD  GMQLQGYRYYDPKTCGFDF  NLFAFFDMAYQGFASGDGD  GINVCLCQSYAKNMGLYGE  QSYAKNMGLYGERVGAFTV  ERLTKEFSVYMTKDGRISV  AGVTSGNVGYLAHAIHQVT  AHKSEIAHRYNDLGEQHFK  GLVLIAFSQYLQKCSYDEH  CAIPNLRENYGELADCCTK  ENPTTFMGHYLHEVARRHP  LHEVARRHPYFYAPELLYY  EVARRHPYFYAPELLYYAE  PYFYAPELLYYAEQYNEIL  YFYAPELLYYAEQYNEILT  PELLYYAEQYNEILTQCCA  ADDRAELAKYMCENQATIS  VEDQEVCKNYAEAKDVFLG  KDVFLGTFLYEYSRRHPDY  VFLGTFLYEYSRRHPDYSV  SLLLRLAKKYEATLEKCCA  CAEANPPACYGTVLAEFQP  NLVKTNCDLYEKLGEYGFQ  GFQNAILVRYTQKAPQVST  DQRLPCVEDYLSAILNRVC  FSALTVDETYVPKEFKAET  XXXXMSSFGYDPYFSTSYK  XMSSFGYDPYFSTSYKRRY  GYDPYFSTSYKRRYVETPR  VHISSVRSGYSTARSAYSS  SGYSTARSAYSSYSAPVSS  STARSAYSSYSAPVSSSLS  SSSLSVRRSYSSSSGSLMP  IAELQAQIQYAQISVEMDV  SEMARYLKEYQDLLNVKMA  MALDIEIAAYRKLLEGEET  TSVGSITSGYSQSSQVFGR  SSQVFGRSAYSGLQSSSYL  AYSGLQSSSYLMSARSFPA  LMSARSFPAYYTSHVQEEQ  MSARSFPAYYTSHVQEEQT  XXXXXXXMSYTLDSLGNPS  LDSLGNPSAYRRVTETRSS  GSPSTVSSSYKRSALAPRL  RSALAPRLAYSSAMLSSAE  LLNGGSGGDYKLSRSNEKE  QGLNDRFAGYIEKVHYLEQ  FAGYIEKVHYLEQQNKEIE  SHITVERKDYLKTDISTAL  QAEEWFKCRYAKLTEAAEQ  RSAKEEIAEYRRQLQSKSI  ERHNHDLSSYQDTIQQLEN  WEMARHLREYQDLLNVKMA  MALDIEIAAYRKLLEGEET  FSGSITGPLYTHRQPSVTI  SEGGDGATKYITKSVTVTQ  TRMQLIMLCYNPDFEKQKP  PWFAGDKVTYVDFLAYDIL  FLAYDILDQYRMFEPKCLD  FEGLKKISAYMKSSRYIAT  ISAYMKSSRYIATPIFSKM  ASEASRLAHYNKRSTITSR  SEGTKAVTKYTSSKXXXXX  GSWFKILLFYVIFYGCLAG  KILLFYVIFYGCLAGIFIG  LTISELKPTYQDRVAPPGL  SFRPNDPKSYEAYVLNIIR  PNDPKSYEAYVLNIIRFLE  LNIIRFLEKYKDSAQKDDM  NCSGLNDDSYGYREGKPCI  SGLNDDSYGYREGKPCIII  PPKNESLETYPLMMKYNPN  DKDKVGNIEYFGMGGYYGF  NIEYFGMGGYYGFPLQYYP  IEYFGMGGYYGFPLQYYPY  GGYYGFPLQYYPYYGKLLQ  GYYGFPLQYYPYYGKLLQP  YGFPLQYYPYYGKLLQPKY  GFPLQYYPYYGKLLQPKYL  YYGKLLQPKYLQPLLAVQF  TEIRVECKAYGENIGYSEK  CKAYGENIGYSEKDRFQGR  TNLDFQMEQYIYKRKSDGI  LDFQMEQYIYKRKSDGIYI  IYKRKSDGIYIINLKRTWE  LCNTDSPLRYVDIAIPCNN  HPWEVMPDLYFYRDPEEIE  WEVMPDLYFYRDPEEIEKE  HLNKGIKQMYMSLPQGEKV  PQGEKVQAMYIWVDGTGEG  QSEGSNSDMYLHPVAMFRD  KLVLCEVFKYNRKPAETNL  HPWFGMEQEYTLMGTDGHP  NGFPGPQGPYYCGVGADKA  GFPGPQGPYYCGVGADKAY  YCGVGADKAYGRDIVEAHY  YGRDIVEAHYRACLYAGVK  VEAHYRACLYAGVKITGTN  IDKLSKRHQYHIRAYDPKG  KRHQYHIRAYDPKGGLDNA  INDPFIDLNYMVYMFQYDS  PFIDLNYMVYMFQYDSTHG  LNYMVYMFQYDSTHGKFNG  FVMGVNHEKYDNSLKIVSN  EGPLKGILGYTEDQVVSCD  DNFVKLISWYDNEYGYSNR  ISWYDNEYGYSNRVVDLMA  SNRVVDLMAYMASKEXXXX  GNPTVEVDLYTAKGLFRAA  ELRDGDKQRYLGKGVLKAV  GAAERDLPLYRHIAQLAGN  DAMRLGAEVYHTLKGVIKD  TLKGVIKDKYGKDATNVGD  VKEAIDKAGYTEKMVIGMD  ASEFYRDGKYDLDFKSPAD  ITGDQLGALYQDFVRNYPV  ALYQDFVRNYPVVSIEDPF  PCRSERLAKYNQLMRIEEE  XXMKVLSLLYLLTAIPGIL  LSLTCSVTGYSITSGYYWN  PGNKLEWMGYISYDGSNNY  KLEWMGYISYDGSNNYNPS  YISYDGSNNYNPSLKNRIS  SVTTEDTATYYCARXXXXX  VTTEDTATYYCARXXXXXX  DVKQYKPVVYSNTIQSLAA  RAMDTLGVEYGDKERKTDS  QECFNRSREYQLNDSAKYY  EYQLNDSAKYYLDSLDRIG  YQLNDSAKYYLDSLDRIGA  SLDRIGAGDYQPTEQDILR  IIFCVALSGYDQVLHEDET  SPLTICFPEYPGSNTYEDA  FPEYPGSNTYEDAAAYIQT  SNTYEDAAAYIQTQFESKN  KNRSPNKEIYCHMTCATDT  ANNLRGCGLYXXXXXXXXX  PLHGGGSLHYSLSRKAGPG  QALNDRFAGYIDKVRQLEA  AGRAAMGELYEREVREMRG  AQALQEECGYLRRHHQEEV  RSAQEEITEYRRQLQARTT  WEMAAQLREYQDLLNVKMA  MALDIEIAAYRKLLEGEEC  HGSVVKSEDYAFPTYADRR  KSEDYAFPTYADRRDYPLP  LSRDEKVQLYRIQFNESFA  ALVLIWEKSYVYGPIPHTF  VLIWEKSYVYGPIPHTFDR  IQGFSAKWDYDKNEWKKXX  LEPEEPPTKYQISQPEAYV  KYQISQPEAYVVAPGESLE  NNRTVLIGEYLQIKGATPR  GATPRDSGLYACTAARTVD  ENRSNQRAPYWTNTEKMEK  FKQEHRIGGYKVRNQHWSL  SVVPSDKGNYTCLVENEYG  NYTCLVENEYGSINHTYHL  NEYGSINHTYHLDVVERSP  GDVEFVCKVYSDAQPHIQW  SKNGPDGLPYLKVLKAAGV  TTDKEIEVLYIRNVTFEDA  NVTFEDAGEYTCLAGNSIG  EKEITASPDYLEIAIYCIG  SPDYLEIAIYCIGVFLIAC  TPMLAGVSEYELPEDPKWE  GACTQDGPLYVIVEYASKG  DGPLYVIVEYASKGNLREY  YASKGNLREYLRARRPPGM  RARRPPGMEYSYDINRVPE  RRPPGMEYSYDINRVPEEQ  TFKDLVSCTYQLARGMEYL  TYQLARGMEYLASQKCIHR  ARDINNIDYYKKTTNGRLP  APEALFDRVYTHQSDVWSF  EIFTLGGSPYPGIPVEELF  KPTNCTNELYMMMRDCWHA  ILTLTTNEEYLDLTQPLEQ  LDLTQPLEQYSPSYPDTSS  QPLEQYSPSYPDTSSSCSS  SVFSPDPMPYEPCLPQYPH  MPYEPCLPQYPHINGSVKT  AAAAERRRLYPPSAEYPDL  RRLYPPSAEYPDLRKHNNC  MASHLTPAVYARLCDKTTP  PVIQERHNGYDPRTMKHTT  LDASKIRSGYFDERYVLSS  IRSGYFDERYVLSSRVRTG  GLKGDLAGRYYRLSEMTEA  LKGDLAGRYYRLSEMTEAE  VQLVIDGVNYLIDCERRLE  FVQEDRAQLYIDCDKMESA  GSSPAIRTNYIGHKTKDLQ  PLCFHNGVQYKNNEEWTVD  CFAGAKCTSYPDGSWKCGA  WKCGACPPGYSGNGIQCKD  HRCKNTDPGYNCLPCPPRF  DCNKNAKCNYLGHYSDPMY  NAKCNYLGHYSDPMYRCEC  YLGHYSDPMYRCECKPGYA  MYRCECKPGYAGNGIICGE  NLVCVANATYHCKKDNCPN  NLPNSGQEDYDKDGIGDAC  DDRDNCPFHYNPAQYDYDR  CPFHYNPAQYDYDRDDVGD  FHYNPAQYDYDRDDVGDRC  VGDRCDNCPYNHNPDQADT  ILNERDNCQYVYNVDQRDT  NERDNCQYVYNVDQRDTDM  HQNNLDNCPYVPNANQADH  NCDPGLAVGYDEFNAVDFS  FINTERDDDYAGFVFGYQS  DDYAGFVFGYQSSSRFYVV  FGYQSSSRFYVVMWKQVTQ  VMWKQVTQSYWDTNPTRAQ  DTNPTRAQGYSGLSVKVVN  HIGWKDFTAYRWRLSHRPK  KIMADSGPIYDKTYAGGRL  DSGPIYDKTYAGGRLGLFV  EMVFFSDMKYECRDSXXXX  LQLVNSTTEYAEFLHCKGK  NKLLPLRRGYIGVVNRSQK  HLADRMGTPYLQKVLNQQL  LSIEKEVDEYKNFRPDDPA  EKELRREISYAIKNIHGIR  RQCTKKLQQYPRLREEMER  MLLIDIELAYMNTNHEDFI  GIMKGGSKEYWFVLTAENL  VLTAENLSWYKDDEEKEKK  KDDEEKEKKYMLSVDNLKL  LFNTEQRNVYKDYRQLELA  TEQRNVYKDYRQLELACET  KASFLRAGVYPERVGDKEK  ETIRNLVDSYMAIVNKTVR  IFSELLANLYSCGDQNTLM  QRRDEMLRMYHALKEALSI  XXXXXXMEGYHKPDQQKLQ  AVLFFHTMRYKALDPRNPH  SKGHAAPILYAVWAEAGFL  GLGAACGMAYTGKYFDKAS  ACGMAYTGKYFDKASYRVY  TGKYFDKASYRVYCMLGDG  YFDKASYRVYCMLGDGEVS  WEAMAFAGIYKLDNLVAIF  APLQHQVDIYQKRCEAFGW  NIRMPTPPSYKVGDKIATR  GDKIATRKAYGLALAKLGH  EHPDRFIECYIAEQNMVSI  RSVPMSTVFYPSDGVATEK  TSRPENAIIYSNNEDFQVG  GRILTVEDHYYEGGIGEAV  RILTVEDHYYEGGIGEAVS  MRASVVLTCYCWLLVRVSS  PCPKVFSNFYSRPGNISKN  FPDFIDACGYNDPEDESKI  PEDESKISFYILVKAIYTL  SFYILVKAIYTLGYSVSLM  LVKAIYTLGYSVSLMSLTT  SVLVKDSVLYSSSGLLRCH  CKLSLVFFQYCIMANFYWL  FQYCIMANFYWLLVEGLYL  FYWLLVEGLYLHTLLVAIL  LPPSRCFLAYLLIGWGIPS  DVGGNDQSQYKRLAKSTLL  LLIPLFGVHYMVFAAFPIG  AFPIGISSTYQILFELCVG  FQGLVVAVLYCFLNSEVQC  CLTQAGSRDYRLHSWSMSR  DEDKESTFGYVHGVSGPVV  ACDMAGAAMYELVRVGHSE  EGDMATIQVYEETSGVSVG  DISSQTQSIYIPRGVNVSA  GSHITGGDIYGIVNENSLI  PPRNRGSVTYIAPPGNYDA  VTYIAPPGNYDASDVVLEL  TVISQSLSKYSNSDVIIYV  KYSNSDVIIYVGCGERGNE  PVAAREASIYTGITLSEYF  IYTGITLSEYFRDMGYHVS  LSEYFRDMGYHVSMMADST  LAEMPADSGYPAYLGARLA  MPADSGYPAYLGARLASFY  YLGARLASFYERAGRVKCL  FPSVNWLISYSKYMRALDE  VNWLISYSKYMRALDEYYD  SKYMRALDEYYDKHFTEFV  KYMRALDEYYDKHFTEFVP  KDDFLQQNGYTPYDRFCPF  FLQQNGYTPYDRFCPFYKT  TPYDRFCPFYKTVGMLSNM  GMLSNMISFYDMARRAVET  IREHMGEILYKLSSMKFKD  VDGVLGGNDYNENNINQWT  THLVKLGKAYKYIVTCAVV  LVKLGKAYKYIVTCAVVQR  TCAVVQRSPYGFHTASSCF  KLHLVSRSIYRSHHPALKL  HKLKLSPIKYGYQPRRNFW  LKLSPIKYGYQPRRNFWPA  LAARLLKLRYIILGSAVGG  ILGSAVGGGYTAKKTFDEW  KDMIPDLSDYKWIVPDFIW  PDFIWEIDEYIDLEKIRKA  DHGSESDKHYRKVSDKEKI  EELLHTQLKYQRILERLEK  KLKKSLIDMYSEVLDVLSD  SEVLDVLSDYDASYNTQDH  DVLSDYDASYNTQDHLPRV  ETIFSISKAYMQNPNAIIL  KLFPMKALGYFAVVTGKGN  SESIEAIREYEEEFFQNSK  NLETEWKNNYPRLRELDRN  VSTHVIENIYLPAAQTMNS  DKQQWDAAIYFMEEALQGR  GPDWKKRWMYWKNRTQEQC  LIKDTWHQVYRRHFLKTAL  HCNLCRRGFYYYQRHFIDS  CNLCRRGFYYYQRHFIDSE  NLCRRGFYYYQRHFIDSEL  TGTEKLIETYFSKNYQDYE  LIETYFSKNYQDYEYLINV  TYFSKNYQDYEYLINVIHA  FSKNYQDYEYLINVIHAFQ  LINVIHAFQYVIYGTASFF  VIHAFQYVIYGTASFFFLY  YGTASFFFLYGALLLAEGF  GALLLAEGFYTTGAVRQIF  GAVRQIFGDYKTTICGKGL  HPDKFVGITYALTVVWLLV  VFACSAVPVYIYFNTWTTC  ACSAVPVYIYFNTWTTCQS  LLTFMIAATYNFAVLKLMG  FETFNTPAMYVAIQAVLSL  VAIQAVLSLYASGRTTGIV  DGVTHTVPIYEGYALPHAI  TVLSGGTTMYPGIADRMQK  KIIAPPERKYSVWIGGSIL  AKLAEQAERYDEMVESMKK  ANTGESKVFYYKMKGDYHR  NTGESKVFYYKMKGDYHRY  VFYYKMKGDYHRYLAEFAT  EAAENSLVAYKAASDIAMT  GLALNFSVFYYEILNSPDR  LALNFSVFYYEILNSPDRA  EQALAVSRNYLSQPRLTYK  NYLSQPRLTYKTVSGVNGP  QPINPQCRIYPEEMIQTGI  VKKSKDVVDYSEENFAIVF  ALTTAEFLAYQCEKHVLVI  LVILTDMSSYAEALREVSA  VPGRRGFPGYMYTDLATIY  GRRGFPGYMYTDLATIYER  YMYTDLATIYERAGRVEGR  THPIPDLTGYITEGQIYVD  TGYITEGQIYVDRQLHNRQ  DRQLHNRQIYPPINVLPSL  DHADVSNQLYACYAIGKDV  DVSNQLYACYAIGKDVQAM  EALTSDDLLYLEFLQKFEK  EKNFITQGPYENRTVYETL  QGPYENRTVYETLDIGWQL  ECATQALEKYNIEKDIAAH  CIVGRNFGSYVTHETKHFI  VTHETKHFIYFYLGQVAIL  HETKHFIYFYLGQVAILLF  VGGSPANTRYLFLGDYVDR  NTRYLFLGDYVDRGYFSIE  FLGDYVDRGYFSIECVLYL  GYFSIECVLYLWALKILYP  LYLWALKILYPKTLFLLRG  NHECRHLTEYFTFKQECKI  TFKQECKIKYSERVYDACM  CKIKYSERVYDACMDAFDC  THNTVRGCSYFYSYPAVCD  NTVRGCSYFYSYPAVCDFL  VRGCSYFYSYPAVCDFLQH  RAHEAQDAGYRMYRKSQTT  EAQDAGYRMYRKSQTTGFP  LITIFSAPNYLDVYNNKAA  FSAPNYLDVYNNKAAVLKY  YNNKAAVLKYENNVMNIRQ  RQFNCSPHPYWLPNFMDVF  AGGVVNSALYNVDAGHRAV  PVASQLPRIYTSIGEDYDE  RIYTSIGEDYDERVLPSIT  QLSRSRNITYLPAGQSVLL  LVKSKKAHSYHQPRSPGPD  HQPRSPGPDYSLRLETVPA  CRSLDEAQPYTLPFKPYAW  QPYTLPFKPYAWSGLAGSD  SDLRHLVQSYRQCSALERS  SEPGRPAARYGPEQAAGGA  ATSAAGLGLYGDFAPAAAG  DFAPAAAGLYERPSTAAGR  RPSTAAGRLYQDHGHELHA  LLIHSDTRPYPCQYCGKRF  SDTRPYPCQYCGKRFHQKS  QEKFRPSIAYFPQIVSVAA  RIINDDQSFYADVYLEDGL  DDQSFYADVYLEDGLIKQI  IPGGIDVNTYLQKPSQGMT  AADTKSCCDYSLHVDITSW  SLHVDITSWYDGVREELEV  DKGVNSFQVYMAYKDLYQM  VNSFQVYMAYKDLYQMSDS  QVYMAYKDLYQMSDSQLYE  LYQMSDSQLYEAFTFLKGL  IAGRINCPVYITKVMSKSA  QVTGSGHCPYSTAQKAVGK  TNAAKIFNLYPRKGRIAVG  AKSHKSTVEYNIFEGMECH  PRKPFPEHLYQRVRIRSKV  GLHSVSRGMYDGPVYEVPA  SRGMYDGPVYEVPATPKHA  AGRPLDLADYTYDLGEEDA  RPLDLADYTYDLGEEDAPE  EEDAPELLNYKDPCKAAAF  FLERTDEDSYIVFTYRPCG  DEDSYIVFTYRPCGCCSYV  TYRPCGCCSYVGRRGGGPQ  RENIQPGQEYNFLKMEVQE  QEVESLGETYDFDSIMHYA  TYDFDSIMHYARNTFSRGI  IFLDTIVPKYEVNGVKPSI  GDIAQARKLYKCPACGETL  STGNFSSPEYPNGYSAHMH  FSSPEYPNGYSAHMHCVWR  ILNFTSMDLYRSRLCWYDY  DLYRSRLCWYDYVEVRDGF  YRSRLCWYDYVEVRDGFWR  WVGKGFFAVYEAICGGDVK  DNGHIQSPNYPDDYRPSKV  IQSPNYPDDYRPSKVCIWR  EIERHDSCAYDYLEVRDGH  ERHDSCAYDYLEVRDGHSE  SESSNLIGRYCGYEKPDDI  SNLIGRYCGYEKPDDIKST  QRCLNTLGSYKCSCDPGYE  SYKCSCDPGYELAPDKRRC  IWQLVAPTQYRISLQFDFF  ETEGNDVCKYDFVEVRSGL  EKPEVITSQYNNMRVEFKS  QDCVNTFGSYECQCRSGFV  ITSPNWPDKYPSKKECTWA  DIESQPECAYDHLEVFDGR  TGNRMFLRFYSDNSVQRKG  RADVKTKDLYSHAQFGDNN  SHAQFGDNNYPGGVDCEWV  EWVIVAEEGYGVELVFQTF  EVEEETDCGYDYIELFDGY  EEETDCGYDYIELFDGYDS  YDYIELFDGYDSTAPRLGR  DSTAPRLGRYCGSGPPEEV  CGSGPPEEVYSAGDSVLVK  ISKKGFHLRYTSTKFQDTL  TGVDNPGHPYIMTVGAVAG  GAVAGDEESYDVFKDLFDP  PIIEERHGGYQPSDEHKTD  QGGDDLDPNYVLSSRVRTG  SLDGDLSGRYYALKSMTEA  LDGDLSGRYYALKSMTEAE  EFMWNPHLGYILTCPSNLG  XXXXXXXXMYPQGRHPAPH  AQYHSLKVEYDKLANEKTE  NEKTEMQRHYVMYYEMSYG  TEMQRHYVMYYEMSYGLNI  EMQRHYVMYYEMSYGLNIE  HYVMYYEMSYGLNIEMHKQ  SEKHRGSADYSMEAKKRKA  AEEKDSLSRYDSDGDKSDD  RTPITLTSSYPAPFAMMSH  NGSLTSPSAYAGLHNIPSQ  AAAAAAAAAYGRSPMVSFG  ASIPGGKPAYSFHVSADGQ  TISNPTRHVYTGGKGCVKI  QLDCLNRDNYIRSCKLLPD  ELTSSAPACYALAISPDAK  FTSQIFSLGYCPTGEWLAV  VLHHTKPDKYQLHLHESCV  SCVLSLKFAYCGKWFVSTG  NLLNAWRTPYGASIFQSKE  SCDISADDKYIVTGSGDKK  GSGDKKATVYEVIYXXXXX  KKATVYEVIYXXXXXXXXX  LPAEVSPINYSLCLKPDLL  ADIDIITASYAPEGDEEIH  EEIHATGFNYQNEDEKVTL  ELNDKMKGFYRSRYTTPAG  KMKGFYRSRYTTPAGEVRY  YTTPAGEVRYAAVTQFEAT  NMNVIDRKPYPDDENLVEV  FARTPVMSTYLVAFVVGEY  YLVAFVVGEYDFVETRSKD  SKDGVCVRVYTPVGKAEQG  EVAAKTLPFYKDYFNVPYP  AKTLPFYKDYFNVPYPLPK  FYKDYFNVPYPLPKIDLIA  AMENWGLVTYRETALLIDP  NEGFASWIEYLCVDHCFPE  LCVDHCFPEYDIWTQFVSA  IWTQFVSADYTRAQELDAL  VDEIFDAISYSKGASVIRM  ASVIRMLHDYIGDKDFKKG  TKQMGFPLIYVEAEQVEDD  QKKFCASGPYGGEDCPQWM  KLNLGTVGFYRTQYSSAML  GTVGFYRTQYSSAMLESLL  MEAFVNEPNYTVWSDLSCN  STLLSHTDFYEEIQEFVKD  LSADLRSPVYLTVLKHGDG  DNWEELHNRYQGGFLISRL  KRDADSIHQYLLQRKTSPP  SSGAGARRPYAARGTPVGP  LWPLSGSTLYFNTSRMKQK  RRKEREDQMYRERLRTLFI  VPESDVVEVYLHPGAVVFG  FGRPRLALMYRMQVANIDK  ESKDRIPVSYKRTGFFGNA  RTGFFGNALYALGMTAVGL  TAVGLAILWYVFRLAGMTG  KLEVREFVDYLKSPERFLQ  ARARAPCIVYIDEIDAVGK  TSVHTFNFEYAVERVIAGT  FSQMLPRDQYLFTKEQLFE  DLRKVTRIAYSMVKQFGMA  EAKLLVAKAYRHTEKVLLD  ALLEKEVINYEDIEALIGP  GSDLPLRFPYGRPEFLGLS  TRRLPWATGYAEVINAGKS  SESEGISCHYWSLFDGHAG  IVVCLLGKLYVANAGDSRA  PETERQRLQYLAFMQPHLL  RKELGKKMLYRDFNMTGWA  RDFNMTGWAYKTIEDDDLK  DDDLKFPLIYGEGKKARVM  DLKVHDSNIYIKPFLSSAP  LSSAPEVRVYDLSRYEHGA  EVRVYDLSRYEHGADDVLI  NCDPDDPHRYTLAAQDLVM  LGSGDDISVYVIPLIHGNK  EKKKGKLDDYQERMNKGER  QDQLDAVSKYQEVTNNLEF  EELSLLDEFYKLVDPERDM  DMSLRLNEQYEHASIHLWD  KEKPVCGTTYKALKEIVER  IVERVFQSNYFDSTHNHQN  EAEPEPAEEYTEQSEVEST  EQSEVESTEYVNRQFMAET  DLMAQMQGPYNFIQDSMLD  PLVSSTSEGYTASQPLYQP  EGYTASQPLYQPSHATEQR  PETLKQQSQYQATYNQSFS  KQQSQYQATYNQSFSSQPH  DQLQTVVGTYHGSQDQPHQ  TGFPRSSQPYYNSRGVSRG  GFPRSSQPYYNSRGVSRGG  RGARGLMNGYRGPANGFRG  GPANGFRGGYDGYRPSFSN  NGFRGGYDGYRPSFSNTPN  SFSNTPNSGYSQSQFTAPR  QSQFTAPRDYSGYQRDGYQ  FTAPRDYSGYQRDGYQQNF  DYSGYQRDGYQQNFKRGSG  XXXXXMSGSYDEASEEITD  TDSFWEVGNYKRTVKRIDD  QERAKIEKAYAQQLTDWAK  RQLIEKGPQYGSLERAWGA  KVKNWQKDAYHKQIMGGFK  KELEAAKKAYHLACKEERL  QDVQKTQEKYEKVLEDVGK  EDVGKTTPQYMEGMEQVFE  HLNLAENSSYMHVYRELEQ  AENSSYMHVYRELEQAIRG  AGDRGSVSSYDRGQTYATE  VSSYDRGQTYATEWSDDES  AKGVRVRALYDYDGQEQDE  GVRVRALYDYDGQEQDELS  RLDSGQLGLYPANYVEAIX  XXXXXXMSSYSSDRDRGRD  ELPKFEKNFYQEHPDLARR  RRTAQEVDTYRRSKEITVR  QTGSGKTLSYLLPAIVHIN  QQVQQVAAEYCRACRLKST  ACRLKSTCIYGGAPKGPQI  GKTNLRRTTYLVLDEADRM  QLAEDFLKDYIHINIGALE  VEDVKFVINYDYPNSSEDY  DVKFVINYDYPNSSEDYIH  YDYPNSSEDYIHRIGRTAR  ARSTKTGTAYTFFTPNNIK  GMKDDRRDRYSAGKRGGFN  FNTFRDRENYDRGYSNLLK  RDRENYDRGYSNLLKRDFG  FGAKTQNGVYSAANYTNGS  QNGVYSAANYTNGSFGSNF  FRTGNPTGTYQNGYDSTQQ  NPTGTYQNGYDSTQQYGSN  QNGYDSTQQYGSNVANMHN  MHNGMNQQAYAYPLPQAAP  NGMNQQAYAYPLPQAAPMI  LPQAAPMIGYPMPTGYSQX  MIGYPMPTGYSQXXXXXXX  VRRRNPQMRYTLSPENLQS  SRSSLTASMYEYTLGQAQN  SSLTASMYEYTLGQAQNLI  VDPSDWIGLYHIDENSPAN  IVWRIEPGPYFMEPEIKIC  EPEIKICFKYYHGISGALR  PEIKICFKYYHGISGALRA  TNPIWHREKYSFFALLTDV  RQAGDQMLSYNLGRRLPAD  RLPADHVSGYLQFKVEVTS  RGRQDSLNDYLDAIEHNGP  DHEFQQDLGYPSSLEEEGG  PSVRQDVSRYQRVDEALPP  RIDSHGRIFYVDHVNRTTT  QQMEQLNRRYQSIRRTMTN  TVLHSNPSAYRMFTNNTCL  RRDTHHFERYQHNRDLVGF  TFNTVSRPQYQDMVPVAYN  QYQDMVPVAYNDKIVAFLR  SLFEEEIMSYVPPHALLHP  PPHALLHPSYCQSPRGSPV  RANARAPAPYKRDFEAKLR  DFEAKLRNFYRKLETKGYG  FYRKLETKGYGQGPGKLKL  EDAFNQIMGYSRKDLQRNK  RKDLQRNKLYVTFVGEEGL  TFVGEEGLDYSGPSREFFF  LVSRELFNPYYGLFEYSAN  VSRELFNPYYGLFEYSAND  FNPYYGLFEYSANDTYTVQ  LFEYSANDTYTVQISPMSA  ILGLALIHQYLLDAFFTRP  LDAFFTRPFYKALLRILCD  ILCDLSDLEYLDEEFHQSL  PVTEKNKKEYIERMVKWRI  QTESLVRGFYEVVDARLVS  LNDWRNNTEYRGGYHDNHI  RNNTEYRGGYHDNHIVIRW  FVTGTSSIPYEGFASLRGS  CFNRLDLPPYPSFSMLYEK  PPYPSFSMLYEKLLTAVEE  IGAILCFLAYGIQAGTEDD  EDDPSGDNLYLGIVLAAVV  VVIITGCFSYYQEAKSSKI  VIITGCFSYYQEAKSSKIM  FFILSLILGYTWLEAVIFL  EIPFNSTNKYQLSIHETED  ETEDPNDNRYLLVMKGAPE  GERVLGFCHYYLPEEQFPK  ERVLGFCHYYLPEEQFPKG  FDNLKKSIAYTLTSNIPEI  DMVPAISLAYEAAESDIMK  VNERLISMAYGQIGMIQAL  IQALGGFFSYFVILAENGF  RTVNDLEDSYGQQWTYEQR  EDSYGQQWTYEQRKVVEFT  ETALAAFLSYCPGMDVALR  PGMDVALRMYPLKPSWWFC  PSWWFCAFPYSFLIFVYDE  FPYSFLIFVYDEIRKLILR  PGGWVEKETYYXXXXXXXX  GGWVEKETYYXXXXXXXXX  KEHTLKIFAYINSWTQRQC  RQCLCCFKEYKHLEIFNQV  KIFQIQFPLYTAYKHNTHP  QIQFPLYTAYKHNTHPTIE  VEVPLHLLRYVCLFCGKNG  LSLMKDCFEYGTPETLPFL  MQHIIPFRTYVIRYLCKLS  IPFRTYVIRYLCKLSDQEL  KESLDLAFKYFMSPTLTMR  AAQLKHCSRYIHDLFPSLI  PGVHTEQTLYLASMLIKAL  QHLPNLASIYHEHLSQGPA  GTFQQFGSSYDTHWITMWA  MKLFFDNLVYYIQGIREGR  KLFFDNLVYYIQGIREGRQ  REGRQKHALYSHSAEVQVR  HCLVEDSECYDDALHWFLN  DQHAMGMETYKHLFLEKMP  NLARLATSAYDGGSNSELC  GDVSRAAIQYINSYYINGK  RAAIQYINSYYINGKTGLE  AAIQYINSYYINGKTGLEK  LEAFRRRFAYHLRQWQIEG  LPDKMTIEMYPSDQVADLR  DLRAEVTHWYENLQKEQIN  SSGHELTTDYDEKALHELG  KIKSAHKLLYALEIIEALG  RIRRESTGSYSDLYPDSDD  ESTGSYSDLYPDSDDSSED  VDPSDLDLAYHDVFAWSGI  LIQRLMSVAYTYDNLAPRV  QRLMSVAYTYDNLAPRVLK  HRSRHEVSHYSMWLLVSWA  VRHESCNGLYKLSLSGLDG  ALKPPRIDDYEEEPLLKPG  PLLKPGCKEYFWLLCKLVD  KSHSCRAAAYDLLVEMVKG  EMVKGSVENYRLIHNWVMA  AQHMQSHAPYKWDYWPHED  QSHAPYKWDYWPHEDVRAE  GLTNLGATCYLASTIQQLY  YLASTIQQLYMIPEARQAV  ARQAVFTAKYSEDMKHKTT  LLELQKMFTYLMESECKAY  YLMESECKAYNPRPFCKTY  YNPRPFCKTYTMDKQPLNT  HVSQTAEEFYTVRCQVADM  CQVADMKNIYESLDEVTIK  KDTLEGDNMYTCSHCGKKV  RILSFNTMRYTFNMVTMMK  FPLRLDMTPYTEDFLMGKS  GDRSKDTESYEYDLIGVTV  RSKDTESYEYDLIGVTVHT  HTGTADGGHYYSFIRDIVN  TGTADGGHYYSFIRDIVNP  IRDIVNPHAYKNNKWYLFN  PHAYKNNKWYLFNDAEVKP  FGGEMTTKTYDSVTDKFMD  FSFEKTHSAYMLFYKRMEP  KTHSAYMLFYKRMEPEEEN  QDKNIFEHTYFGFMWQLCS  RLRPVHAHLYLQPGMEDGS  KPHSKHLTEYFAFLYEFAK  HLTEYFAFLYEFAKMGEEE  QAISTMVHFYMGTKGPENP  DILSLAEEKYRPAALEKMI  CNLIFSLCRYNNRLAEHIV  PPGMPPFASYILQRIWEVI  LQRIWEVIEYNPSQCLDWL  VERFLLAHNYPRVRTSAAY  YPRVRTSAAYLLVSLIPSN  DTTVVLHQVYNVLLGLLSR  LGLLSRAKLYVDAAVHGTT  VHGTTKLVPYLSFMTYCLI  LVPYLSFMTYCLISKTEKL  KTEKLMFSTYFMDLWNLFQ  NKQALLSFWYNVCADCPEN  VVTKNIAFNYILADHDDQD  LFNRGMLPAYYGILRLCCE  FNRGMLPAYYGILRLCCEQ  KNLTPHASQYPGAVEELFN  QFKKTTISCYLRCLDGRSC  ESFNTLHMMYHEATACHVT  LSVLKSTRPYLQRKDVKQA  HKLLTLLNSYSPPELRNAC  PFLQHNHCTYHHSNIPMSL  SNIPMSLGPYFPCRENIKL  ETSKGKDDVYDRMLLDYFF  DVYDRMLLDYFFSYHQFIH  RMLLDYFFSYHQFIHLLCR  LVKLSVLVAYEGLPLHLAL  LCEDPVFAEYIKCILMDER  RTFLNNNIVYTFMTHFLLK  TLITNLINQYQNLQSDFTN  SPENSSVKEYRMEGPSSFS  DLQLERINVYYNEAAGNKY  YYNEAAGNKYVPRAILVDL  LLISKIREEYPDRIMNTFS  KVSDTVVEPYNATLSVHQL  QLVENTDETYSIDNEALYD  TYSIDNEALYDICFRTLKL  PLTSRGSQQYRALTVPELT  AACDPRHGRYLTVAAIFRG  LNVQNKNSSYFVEWIPNNV  FRRKAFLHWYTGEGMDEME  SNMNDLVSEYQQYQDATAD  NDLVSEYQQYQDATADEQG  SLLVTGIPEYCSHAEIEET  NGVLLPLGTYRVLNKIFLR  LGAVVQVIFYMDAAIRSRD  SAFKMEDRNYWKNTEDHGD  CWRKSQGSNYNSNYNKVNL  SQGSNYNSNYNKVNLEAGE  IGGSLTKLAYYSTVQHKVA  GGSLTKLAYYSTVQHKVAK  DVEQDHEPPYEISVQEEIT  LHFIKFENTYMEACLDFIR  VIQATGGGAYKFKDLIEEK  KNIPHEAFMYQKDSDPEFR  QTNHPNIFPYLLVNIGSGV  NVDMLVQDIYGGAHQTLGL  NDIGQLACLYAKLHGLDRV  HPVTMRTITYSINFFSKGE  QALFLRHEGYLGAIGAFLK  GAEQDNPNQYSWGENYAAS  PNQYSWGENYAASSGLMST  LPLLLDPSSYVPDTVDLTD  LTDDALARQYWLTCFEEAL  ERAEKFRQKYWGKLQTLRH  QTLRHQPFAYGTLTVRSLL  LNEFNFPDPYSKVKQKENG  QERPWLVDSYTKWLQRLKG  SGPALNDVTYSESLIVAER  GMGRAIHTNYHALLRCESL  GQLFSVIFKYEVPTKXXXX  HELRNIFLQYASTEVDGEH  ASTEVDGEHYMTPEDFVQR  MTPEDFVQRYLGLYNDPNS  DFVQRYLGLYNDPNSNPKI  DQTKDGLISYQEFLAFESV  GHNRKKHLNYVEFTQFLQE  GTSHQVSFSYFNAFNSLLN  NNMELVRKIYSTLAGTRKD  EEFAQSAIRYGQVTPLEID  VTPLEIDILYQLADLYNAS  DILYQLADLYNASGRLTLA  IWLQIAESAYRFTLGSVAG  AGAVGATAVYPIDLVKTRM  TGSVVGELMYKNSFDCFKK  FDCFKKVLRYEGFFGLYRG  LRYEGFFGLYRGLIPQLIG  LQDLGLFGLYKGAKACFLR  LRDIPFSAIYFPVYAHCKL  PFSAIYFPVYAHCKLLLAD  VAARAGQTTYSGVVDCFRK  PQFGVTLVTYELLQRWFYI  TYELLQRWFYIDFGGLKPS  PANPDHIGGYRLATATFAG  AGIENKFGLYLPKFKSPSV  KLISATDIQYSASLLNSLN  RNILSASSTYFHQLFSVAG  AEIFAEILNYIYSSKVVRV  IFAEILNYIYSSKVVRVRA  EGLSTGCKVYANIGEDTYD  VYANIGEDTYDIVIPVKDD  RMKVKHDDHYELIVDGRVY  YICIVCKRSYVCLTSLRRH  FNIHSWEKKYQCRYCDKVF  SWEKKYQCRYCDKVFPLAE  CDKVFPLAEYRTKHEIHHT  EIHHTGERRYQCLTCGKSF  LTCGKSFINYQFMSSHIKS  QDPSGDSKLYRLHPCKSLQ  PCKSLQIRQYAYLSNKSSA  KSLQIRQYAYLSNKSSAMP  PVMKDDAVGYKVDAGKEPP  EFEFIIPESYXXXXXXXXX  GFAKKTFISYSATFKDNFR  SCQQAAIQAYEDMVKQIQR  EMAAEIKTEYPEKEVTLIH  NLEELPRNEYREYIKVETD  ELPRNEYREYIKVETDKGT  VNEFLQVEGYSNIYAIGDC  LQVEGYSNIYAIGDCADTK  ADTKEPKMAYHAGLHANVA  SMKQRPLKAYKPGALTFLL  DGVGQISGFYVGRLMVRLA  RANKVNWQSYLQGQMISAE  HISKEQTVQYILTMVDDML  HQRVSIFFDYAKRSKSTAW  KRSKSTAWPYFLPMLNRQD  ELMEGSDLNYYFNWIKTQL  LMEGSDLNYYFNWIKTQLS  TISSSDSSQYVQCVAGCLQ  LQLMLRVNEYRFAWVEADG  SNKCGFQLQYQMIFSIWLL  PQMCEHLRRYNIIPVLSDI  STERETRQEYALAMIQCKV  QLENLEQQKYDDEDISEDI  VQDLSSFDEYSSELKSGRL  NAVRLNEKNYELLKILTKL  HDVGEYVRHYPRGKRVIEQ  MHHEDQQVRYNALLAVQKL  QKLMVHNWEYLGKQLQSEQ  APTTSPIPGYAFKPPPRPD  EMDIPKIDIYHYELDIKPE  DIPKIDIYHYELDIKPEKC  PVFDGRKNLYTAMPLPIGR  VMRHLPSMRYTPVGRSFFT  NIDVSATAFYKAQPVIEFV  THCGQMKRKYRVCNVTRRP  QTVECTVAQYFKDRHKLVL  KDRHKLVLRYPHLPCLQVG  QVGQEQKHTYLPLEVCNIV  RSASFNTDPYVREFGIMVK  RVLQPPSILYGGRNKAIAT  IQGQPCFCKYAQGADSVEP  PMFRHLKNTYAGLQLVVVI  VILPGKTPVYAEVKRVGDT  GSMDAHPNRYCATVRVQQH  MVRELLIQFYKSTRFKPTR  RFKPTRIIFYRDGVSEGQF  EACIKLEKDYQPGITFIVV  ITHPTEFDFYLCSHAGIQG  IQGTSRPSHYHVLWDDNRF  SSDELQILTYQLCHTYVRC  ILTYQLCHTYVRCTRSVSI  RSVSIPAPAYYAHLVAFRA  SVSIPAPAYYAHLVAFRAR  AHLVAFRARYHLVDKEHDS  VHQDTLRTMYFAXXXXXXX  TLKDGEGACYTSLISDVCY  YTSLISDVCYPPREDSAYF  CYPPREDSAYFTGILQKEN  PLDQMKAEAYKYIDITRPQ  DQMKAEAYKYIDITRPQEA  LFEESTFAPYIDELSDEQH  AIKEAKGLSYETTESPRPV  ASEDALPSGYVSFGHVSGP  PSPASPSIQYSILREEREA  NKQKAIDLLYWRDIKQTGI  QFSVVSVVAYLALAALSAT  LSATISFRIYKSVLQAVQK  TDEGHPFKAYLELEITLSQ  TLSQEQIQKYTDCLQLYVN  QKYTDCLQLYVNSTLKELR  FAVLMWLLTYVGALFNGLT  VSMFTLPVVYVKHQAQVDQ  QEVRCLERSYASKPTLNEV  IPKEEVKEVYMGNVIQGGE  GMESMSNVPYVMSRGATPY  YVMSRGATPYGGVKLEDLI  IVKDGLTDVYNKIHMGNCA  NISRQEQDTYALSSYTRSK  EQDTYALSSYTRSKEAWDA  DVVVKEDEEYKRVDFSKVP  AYAVPKVLKYAGLKKEDIA  GFAGDQIPKYCFPNYVGRP  QIPKYCFPNYVGRPKHMRV  EHRGLLTIRYPMEHGVVRD  WNDMERIWQYVYSKDQLQT  DMERIWQYVYSKDQLQTFS  ISMQAVLSLYATGRTTGVV  DGVTHAVPIYEGFAMPHSI  DIAGRDVSRYLRLLLRKEG  VRTIKERACYLSINPQKDE  EALETEKVQYTLPDGSTLD  KISAPQERLYSTWIGGSIL  KKMWVSKKEYEEDGSRAIH  IGLSNSPKGYVRTTGTAAS  VGMQIPRFCYHERLSVAGN  RGNDMQVGTYIEKMFMSEL  PVGALTSKPYAFTARPWET  WISDKTRFAYDGLKRQRLT  VRNEKGLLTYTSWEDALSR  GAGTDLRSNYLLNTTIAGV  LIGSPVDLTYRYDHLGDSP  GSPVDLTYRYDHLGDSPKI  SQVAALDLGYKPGVEAIRK  DLPKDCFIVYQGHHGDVGA  ADVILPGAAYTEKSATYVN  AAYTEKSATYVNTEGRAQQ  EEVSPNLVRYDDIEETNYF  RYDDIEETNYFQQASELAK  PPQLTIKDFYMTDSISRAS  RKQDVRIPTYNISVVGLSG  RVVNNDHFLYWGEVSRSLE  PHRSTALQPYIKRAAATKL  KLASAEKLMYFCTDQLGLE  DQLKFVSNLYNQLAKTKKP  TKCDEGVERYIRDAHTFAL  SRGKTKIIPYFEALKQQSQ  QQIATAKDKYEWLVSRIVK  SRKMQASPEYQDYVYLEGT  MQASPEYQDYVYLEGTQKA  ASPEYQDYVYLEGTQKAKK  EHIERRRKLYLAALPLAFE  MDTVPAEQLYETHLEKLRN  RSFIMNEDFYQWLEESVYM  FYQWLEESVYMDIYGKHQK  EEFQELLLEYSELFYELEL  LLLEYSELFYELELDAKPS  LILKHIHFVYHPTKETCPS  RALCTNDDKYVIDGKMYEL  DKYVIDGKMYELSLRPIEG  FQPHGCLCLYNSKESLSYV  LYNSKESLSYVVESIEKSR  LDPASAGIGYGRNINEKQI  KRIELSVLSYHSSFSIRKS  IRKSRLVHGYIVFYSAKRK  RLVHGYIVFYSAKRKASLA  KNIIEATHMYDNVAEACST  SEEDVEPPSYHLFREDATL  HFDITKDLSYLDQGHREGQ  PQDGFDPSDYAEPMDAVVK  KPRNEEENIYSVPHDSTQG  VSAVSKPVLYRTRCTRLGR  CTRLGRFASYRTSFSVGSD  KEEDQASQGYKGDNAVIPY  YKGDNAVIPYETDEDPRRR  ITKATWESNYFGVPLTTVV  PIFIERCIEYIEATGLSTE  ATGLSTEGIYRVSGNKSEM  SELPDPLVPYSMQIDLVEA  PKENHEVFKYVISHLNKVS  MDALTATRSYQTIIELFIQ  FIQQCPFFFYNRPISEPPG  WNPLGDSAFYRKYELYSMD  LGDSAFYRKYELYSMDWDL  SAFYRKYELYSMDWDLKEE  KDCLVAAAPYGGPIALLRN  ASVRPVLEIYSASGLPLAS  VQEDGAVLVYGLHGDFRRH  SGVAILTGAYRFTLSANVG  ILLAVGPDLYLLDHATCSA  FLQMAVSFTYRYLALFTDT  QMAVSFTYRYLALFTDTGY  YLALFTDTGYIWMGTASLK  IQFVLDEDSYLVPELDGVR  ALLLEAQKEYEKESQKADE  EKESQKADEYLREIQELGQ  LRVLNAIRDYHIGIPLTYT  DYHIGIPLTYTQYKQLTIQ  IGIPLTYTQYKQLTIQVLL  LDRLVLRRLYPLAIQICEY  YPLAIQICEYLRLPEVQGV  SRILAHWACYKARAWDMRD  KLGDTPGVSYSDIAARAYG  SYSDIAARAYGCGRTELAI  TELAIKLLEYEPRSGEQVP  RNQPMALSLYRQFCKHQEL  QELDTLKDLYNQDDNHQEL  LGSFHIRASYAAEERIEGR  ALQTAADAFYKAKNEFAAK  SKSKKSPIGYLPFVEICMK  QHNKHEAKKYASRVGPEQK  XXMADEGKSYNEHDDRVSF  DPQDGPRVRYNPYTNRPNR  DGPRVRYNPYTNRPNRRGD  KITIPYGRKYDKTWLLSMI  VPFNPIEFHYENTRAHFFV  IIINASAPPYTVQNELKPE  QLKLIMSKRYDGNQQALDL  SLNLSSNRLYKLDDMSSIV  CDNFGDQSSYISAIRERFP  TMLPPCKGSYFGTENLKSL  SLVLRFLQQYYVIYDSGDR  LVLRFLQQYYVIYDSGDRQ  RFLQQYYVIYDSGDRQGLL  DSGDRQGLLYAYHDGACCS  GDRQGLLYAYHDGACCSLS  GACCSLSIPYNPQNPVRKN  NPVRKNLAEYVKDSRNVKK  KCLQDNNWDYTRSAQAFTL  KVLVAQHDAYKGLLPEELT  SPDTFVRTIYAGNALCTVK  TGKIVAPELYIAVGISGAI  EAPIFQVADYGIVADLFKV  AKLAEQAERYDDMAAAMKA  EERNLLSVAYKNVVGARRS  ATQAESKVFYLKMKGDYFR  VFYLKMKGDYFRYLSEVAS  LKMKGDYFRYLSEVASGEN  TTVSNSQQAYQEAFEISKK  GLALNFSVFYYEILNSPEK  LALNFSVFYYEILNSPEKA  XXMSGAPPSYSFVALPPRA  PRDEVQEVVYFPAVDHDAE  DAESKVECTYISIDQVPRT  LDEHGQGGNYYEDAHSCHS  DEHGQGGNYYEDAHSCHSF  HSCHSFQSAYLLVDRDEAW  AWVLETVGKYWAAERITEG  DEEHPELRTYAQSQGWWTG  EKPDRRHELYKAHEWARAV  DPAEVGDLFYDCVDTEMKF  GALHKGCVCYFSVSTRHHT  AGGAGVPAFYTSTGYGTLV  VPAFYTSTGYGTLVQEGGS  VQEGGSPIKYNKDGSVAIA  PEDIHIPKIYVHRLIKGEK  VHRLIKGEKYEKRIERLSL  AALEFEDGMYANLGIGIPL  ENGVLGLGPYPLKDEADAD  MLGAMQVSKYGDLANWMIP  TPFDFTPENYKRIEAIVKN  VLQVPPMRVYEVATFYTMY  MRVYEVATFYTMYNRKPVG  YEVATFYTMYNRKPVGKYH  MYNRKPVGKYHIQVCTTTP  APMVQINDNYYEDLTPKDI  PMVQINDNYYEDLTPKDIE  KWLDGFRKWYYNAAGFNKL  WLDGFRKWYYNAAGFNKLG  ILPKDQWTKYEEDKFYLEP  WTKYEEDKFYLEPYLKEVI  EEDKFYLEPYLKEVIRERK  XXXXXXMAPYQFQNAKDLQ  VQVYGIEGRYATALYSAAS  IEGRYATALYSAASKEKKL  KVSLAVLNPYIKRTVKVKS  GMIVRIGEKYVDMSAKSKI  XXXXMTDSKYFTTNKKGEI  DNLELKKLVYLYLMNYAKS  LELKKLVYLYLMNYAKSQP  KLVYLYLMNYAKSQPDMAI  CIRVDKITEYLCEPLRKCL  RKCLKDEDPYVRKTAAVCV  IFILDCLSNYNPKDDREAQ  LELLPKDSDYYNMLLKKLA  ELLPKDSDYYNMLLKKLAP  LLSGEPEVQYVALRNINLI  QEIKVFFVKYNDPIYVKLE  FFVKYNDPIYVKLEKLDIM  AQVLAELKEYATEVDVDFV  LDLIQTKVNYVVQEAIVVI  VVIRDIFRKYPNKYESIIA  DIFRKYPNKYESIIATLCE  AAMIWIVGEYAERIDNADE  DNPDLRDRGYIYWRLLSTD  PDLRDRGYIYWRLLSTDPV  CHIGSLASVYHKPPNAFVE  TFTHRQGHIYMEMNFTNKA  AVKNNIDVFYFSCLIPLNV  SSKLQNNNVYTIAKRNVEG  RNVEGQDMLYQSLKLTNGI  LRIQPGNPNYTLSLKCRAP  KCRAPEVSQYIYQVYDSIL  RAPEVSQYIYQVYDSILKN  EVSQYIYQVYDSILKNXXX  KGLDRVQDEYSRRSYSRFE  VQDEYSRRSYSRFEEEDDD  DDFPAPADGYYRGEGAQDE  DFPAPADGYYRGEGAQDEE  EGHDEDDEIYEGEYQGIPR  EDDEIYEGEYQGIPRAESG  KDREELAQQYETILRECGH  GHGRFQWTLYFVLGLALMA  NKGMLGLIVYLGMMVGAFL  AFFSSFVQGYGTFLFCRLL  GGSIPIVFSYFSEFLAQEK  CMFWMIGGVYAAAMAWAII  AMAWAIIPHYGWSFQMGSA  GWSFQMGSAYQFHSWRVFV  EIQSDTGTWYQRWGVRALS  NFLSCFSPEYRRITLMMMG  GVWFTMSFSYYGLTVWFPD  VWFTMSFSYYGLTVWFPDM  MIRHLQAVDYAARTKVFPG  FEDSLFEECYFEDVTSSNT  NCTFINTVFYNTDLFEYKF  VFYNTDLFEYKFVNSRLVN  DVTGTGEGAYMVYFVSFLG  GTGEGAYMVYFVSFLGTLA  ALDVLTVELYPSDKRTTAF  SCSTVGGVGYGDSKDCILE  TAPFEEQENYFLLCDVLPE  KKLDNLQCLYCEKTFRDKN  RKINPKNREYDRFYVINYL  PKNREYDRFYVINYLELGK  EYDRFYVINYLELGKSWEE  KQAETIEKLYVHMEDAHEF  IKSELGLNFYQQVKLVNFI  RRQVHQCRCYGCHVKFKSK  DRKTWDQLEYYFPTYENDT  RKTWDQLEYYFPTYENDTL  DQLEYYFPTYENDTLLCTL  IISEDTSKLYALKQSSILN  HRGSIPAMSYAPFRDVRGP  RGPPMHRTQYVHSPYDRPG  HRTQYVHSPYDRPGWNPRF  EGRPHGEHEYHLGRSRRKS  RKSVPGGKQYSMEAAPAAP  ARELPPKKRYYCELCLDDM  RELPPKKRYYCELCLDDML  CELCLDDMLYARTTSKPRS  AVRALRLHLYRDSDKKRKK  KKRKKDKAGYVGLVTVPVA  AGRHFTEQWYPVTLPTGSG  CPAVRLKARYQTMSILPME  TMSILPMELYKEFAEYVTN  MELYKEFAEYVTNHYRMLC  EFAEYVTNHYRMLCAVLEP  TLATKAIEEYMRLIGQKYL  AIGEFIRALYESEENCEVD  PSLFGLMQEYPDEQTSRTL  EWGSMQQFLYEISNLDTLT  LTNSSSFEGYIDLGRELST  RGPSAEMQGYMMRDLNSSI  PPGGGKDLFYVSRPPLARS  PPLARSSPAYCTSSSDITE  GDTFAPFHGYSKSEDLSTG  AASILHSHSYSDEFGPSGT  NLLQSPEPSYGPARPRQQS  ESAHIEREEYKLKEYSKSM  EREEYKLKEYSKSMDESRL  ESRLDRVKEYEEEIHSLKE  HMSNRKLEEYERRLLSQEE  EQTSKILMQYQARLEQSEK  RPTAPQISVYSGIPDRQTV  HRQPSTAAQYLQQMYAAQQ  TAAQYLQQMYAAQQQHLML  ALTASQAQMYLRAQMLIFT  ATHPLIAPAYAHLQSHQLL  TTDSEMEEPYLQESKEEGT  ELCGRVDFAYKFKRSKRFC  FCSMACAKRYNVGCTKRVG  SSRCSDNSSYEEPLSPISA  PTKWNVEDVYEFIRSLPGC  IKLGPALKIYARISMLKDS  GQTHSVETPYGSVTFTVYG  PYGSVTFTVYGTPKPKRPA  FTYHDVGLNYKSCFQPLFR  EGAPVFPLGYQYPSLDQLA  APVFPLGYQYPSLDQLADM  ADMIPCILQYLNFSTIIGV  IGVGVGAGAYILSRYALNH  GAGAYILSRYALNHPDTVE  SGNSELIQKYRGIIQHAPN  APNLENIELYWNSYNNRRD  ENIELYWNSYNNRRDLNFE  PGKLTEAFKYFLQGMGYMA  FKYFLQGMGYMASSCMTRL  KLSVDLIKTYKHINEVYYA  KTYKHINEVYYAKKKRRAQ  TYKHINEVYYAKKKRRAQQ  KEKKVLNHGYDDDNHDYIV  HGYDDDNHDYIVRSGERWL  RSGERWLERYEIDSLIGKG  MNQHDTEMKYYIVHLKRHF  NQHDTEMKYYIVHLKRHFM  LCLVFELLSYNLYDLLRNT  VFELLSYNLYDLLRNTHFR  SSCQLGQRIYQYIQSRFYR  CQLGQRIYQYIQSRFYRSP  IYQYIQSRFYRSPEVLLGT  SPEVLLGTPYDLAIDMWSL  LEQAPKARKYFERLPGGGW  RRTKELRKDYQGPGTRRLQ  GEPGHSPADYLRFQDLVLR  QDLVLRMLEYEPAARISPL  SGSSNDNRAYRYSNRYCGG  SSNDNRAYRYSNRYCGGPG  NRAYRYSNRYCGGPGPPIT  QMKIIHEAGYSEEECKQYK  GYSEEECKQYKAVVYSNTI  ECKQYKAVVYSNTIQSIIA  QACFNRSREYQLNDSAAYY  EYQLNDSAAYYLNDLDRIA  YQLNDSAAYYLNDLDRIAQ  IIFCVALSDYDLVLAEDEE  IKKSPLTICYPEYAGSNTY  SPLTICYPEYAGSNTYEEA  YPEYAGSNTYEEAAAYIQC  SNTYEEAAAYIQCQFEDLN  NKRKDTKEIYTHFTCATDT  RSSVSDYGNYDIIVRHYNY  GNYDIIVRHYNYTGKLNIG  YDIIVRHYNYTGKLNIGAE  KTKKFHRPMYYFIGNLALS  TKKFHRPMYYFIGNLALSD  LSDLLAGVAYTANLLLSGA  NLLLSGATTYKLTPAQWFL  SLLAIAIERYITMLKMKLH  SSCSTVLPLYHKHYILFCT  TVLPLYHKHYILFCTTVFT  LLLLSIVILYCRIYSLVRT  SIVILYCRIYSLVRTRSRR  CKAKTCDILYKAEYFLVLA  TCDILYKAEYFLVLAVLNS  LNSGTNPIIYTLTNKEMRR  XXXXXXXMSYQGKKNIPRI  KIVNDDQSFYADIYMEDGL  DDQSFYADIYMEDGLIKQI  WADSKSCCDYSLHVDITEW  DHGVNSFLVYMAFKDRFQL  IANQTNCPLYVTKVMSKSA  QARKKGTVVYGEPITASLG  ASLGTDGSHYWSKNWAKAA  TNAAKVFNLYPRKGRISVG  LHVTEGSGRYIPRKPFPDF  PRKPFPDFVYKRIKARSRL  ELRGVPRGLYDGPVCEVSV  AVDGVMNGEYYQESNGPTD  VDGVMNGEYYQESNGPTDS  QESNGPTDSYAAISEVDRL  NNRVADEAFYKQPFADLIG  KQPFADLIGYVAAEEAFVN  EAPVEGDIHYQRGGAPLPP  QGMFVLGLPYAILHGGYLG  LPYAILHGGYLGLFLIIFA  IIFAAVVCCYTGKILIACL  TGKILIACLYEENEDGEVV  IELVMTCILYVVVSGNLMY  YVVVSGNLMYNSFPGLPVS  FVINILVIAYCLSRARDWA  DWAWEKVKFYIDVKKFPIS  ISIGIIVFSYTSQIFLPSL  LKGLFALVAYLTWADETKE  FLVAKALLSYPLPFFAAVE  GSRAFFPACYGGDGRLKSW  VVFTLLMAIYVPHFALLMG  HSLEGLIEAYRTNAEDXXX  XXXXMTDSKYFTTTKKGEI  DNLELKKLVYLYLMNYAKS  LELKKLVYLYLMNYAKSQP  KLVYLYLMNYAKSQPDMAI  CIRVDKITEYLCEPLRKCL  RKCLKDEDPYVRKTAAVCV  IFILDCLANYMPKDDREAQ  MEMLSKDLDYYATLLKKLA  EMLSKDLDYYATLLKKLAP  LLSAEPELQYVALRNINLI  HEMKVFFVKYNDPIYVKLE  FFVKYNDPIYVKLEKLDIM  AQVLAELKEYATEVDVDFV  LDLIQTKVNYVVQEAIVVI  VVIKDIFRKYPNKYESVIA  DIFRKYPNKYESVIATLCE  AAMIWIVGEYAERIDNADE  DNPDLRDRGYIYWRLLSTD  PDLRDRGYIYWRLLSTDPV  PTLLDELICYIGTLASVYH  SGVGTLSGSYVAPKAVWLP  AVKNNIDVFYFSTLYPLHV  IDVFYFSTLYPLHVLFVED  RNVEGQDMLYQSLKLTNGI  RAPEVSQHVYQAYETILKN  EVSQHVYQAYETILKNXXX  XXXXXXXXMYWKSDQMFVC  SCGLEIVDKYLLKVNDLCW  TSLGRHTSCYIKDKDIFCK  DKDIFCKLDYFRRYGTRCS  WVRRAKGNVYHLACFACFS  SPPMLEEMAYSAYVPQDGT  MLEEMAYSAYVPQDGTMLT  GTMLTALHSYMDAHQQLLD  QQLLDSSPCYPIQXXXXXX  PRRTPQNVPYQDLPHLVNA  PHLVNADGQYLFCRYWKPS  ADGQYLFCRYWKPSGTPKA  QHVDTIQKDYPDVPIFLLG  ILVAAERPTYFSGMVLISP  SRNKSEVDLYNSDPLVCRA  DRLCDSKGAYLLMESSRSQ  RSQDKTLKMYEGAYHVLHR  KTLKMYEGAYHVLHRELPE  PFSGGGGGEYMEQEEDWDR  LWCQRKTAPYRNVNVQNFH  HRHRPDLIDYAKLRKDDPI  NTAFEVAEKYLDIPKMLDA  KPDEKAIMTYVSCFYHAFA  AIMTYVSCFYHAFAGAEQA  QRKLEDFRDYRRLHKPPRV  RGLEQVEKGYEDWLLSEIR  KEEMLNQHDYESASLQEVR  LAQELNELDYHEAASVNSR  GEIQKICQTYGLRPKSGNP  GLRPKSGNPYITLSSQDIN  RQQEQNIINYKSNIDRLEG  LVFDNKHTVYSMEHIRVGW  FRACLISMGYDLGEVEFAR  FKILAGDKNYITPEELRRE  RELPAEQAEYCIRRMAPYK  EYCIRRMAPYKGSGAPSGA  SGAPSGALDYVAFSSALYG  DYVAFSSALYGESDLXXXX  VTIVCLDLGYTLKCNKLVP  VTIVCLDLGYTLECHNQQS  PSEPEFEQAYKELAYTLEN  FEQAYKELAYTLENSSLFQ  SSLFQKHPEYRTALTVASI  NGNVQVNRGYRVQFNSALG  VQFNSALGPYKGGLRLHPS  IGVGGREIGYMFGAYRKAA  SLIRPEATGYGLVYYVGHM  PEATGYGLVYYVGHMLEYS  EATGYGLVYYVGHMLEYSG  VYYVGHMLEYSGAGSYAGK  MLEYSGAGSYAGKRVALSG  LSGSGNVAQYAALKLIELG  KEKKGEAVWYAPGKAANCG  FNGLNTAKTYVEAAEGELP  DDFKGRPDLYSSSFITEGQ  SNPASSSSCYLEEHVSQEA  TTALSWSLMYLVTNPRRQR  GVPVDLTPIYGLTMKHPRC  DRNVATPRDYYFALAHTVR  RNVATPRDYYFALAHTVRD  SMATLGLAAYGYGIRYEFG  ATLGLAAYGYGIRYEFGIF  WEVTVKTCAYTNHTVLPEA  DVQVKRIHEYKRQLLNCLH  LNCLHVITLYNRIKKEPNK  DQRGYNAQEYYDRIPELRQ  QRGYNAQEYYDRIPELRQI  ENTEENRRFYRQLLLTADD  GVILFHETLYQKADDGRPF  DGLSERCAQYKKDGADFAK  MENANVLARYASICQQNGI  VTEKVLAAVYKALSDHHIY  LKPWALTFSYGRALQASAL  VTLGCLVKGYLPEPVTVTW  PSVRQSSGLYSLSSVVSVT  RGQPLEPKVYTMGPPREEL  SLTCMINGFYPSDISVEWE  EKNGKAEDNYKTTPAVLDS  PAVLDSDGSYFLYNKLSVP  LDSDGSYFLYNKLSVPTSE  VMHEALHNHYTQKSISRSP  INDPFIDLHYMVYMFQYDS  PFIDLHYMVYMFQYDSTHG  LHYMVYMFQYDSTHGKFHG  IKWGDAGAEYVVESTGVFT  FVMGVNHEKYDNSLKIVSN  TCRLEKAAKYDDIKKVVKQ  EGPLKGILGYTEDQVVSCD  XXXXXXXMDYYRKYAAAIL  XXXXXXMDYYRKYAAAILA  XXXMDYYRKYAAAILAILS  YFSKPDAPIYQCMGCCFSR  FDRAVMVSHYIHNLSSEMF  LLRSWNDPLYHLVTEVRGM  APAPVNPCCYYPCQHQGIC  PAPVNPCCYYPCQHQGICV  ICVRFGLDRYQCDCTRTGY  YQCDCTRTGYSGPNCTIPE  SNLIPSPPTYNIAHDYISW  PPTYNIAHDYISWESFSNV  SWESFSNVSYYTRILPSVP  WESFSNVSYYTRILPSVPR  GHGVDLGHIYGDNLERQYQ  IYGDNLERQYQLRLFKDGK  RLFKDGKLKYQMLNGEVYP  KYQMLNGEVYPPSVEEAPV  VEEAPVLMHYPRGIPPQSQ  FGLLPGLMLYATIWLREHN  ETIKIVIEEYVQQLSGYFL  ELLFGAQFQYRNRIAMEFN  DSFRVGPQDYSYEQFLFNT  FRVGPQDYSYEQFLFNTSM  VLRLQPFNEYRKRFGMKPY  YRKRFGMKPYTSFQELTGE  EMAAELEELYGDIDALEFY  YGDIDALEFYPGLLLEKCH  LGNPICSPEYWKASTFGGE  VCLNTKTCPYVSFHVPDPR  CQGQAQHPPYCRNQPGKCQ  FDRATTVANYNSKLAGEMV  IHPGEKNEPYPVWSEQSSL  DENVRRVAFYRLFHCLHRD  CLHRDSSKIYTYLRILKCR  HRDSSKIYTYLRILKCRLT  IVVGGGANAYAAEEDNTDN  LSMDEISDAYFDYHGDVSD  DEISDAYFDYHGDVSDSVD  TFAKAVSDAYAYADALKKD  AKAVSDAYAYADALKKDNG  DALKKDNGEYTVDVADKGL  TFEEATAKAYAYANLLAKE  EEATAKAYAYANLLAKENG  NLLAKENGEYTADLEDGGN  TFEEATAEAYRYADLLAKV  EEATAEAYRYADLLAKVNG  DLLAKVNGEYTADLEDGGY  YTADLEDGGYTINIKFAGK  KEAGITSDLYFSLINKAKT  EETPELKDGYATYEEAEAA  PELKDGYATYEEAEAAAKE  LKNDDVNNAYEIVQGADGR  EIVQGADGRYYYVLKIEVA  IVQGADGRYYYVLKIEVAD  VQGADGRYYYVLKIEVADE  EDTPEVQEGYATYEEAEAA  PEVQEGYATYEEAEAAAKE  LKEDKVNNAYEVVQGADGR  EVVQGADGRYYYVLKIEDK  VVQGADGRYYYVLKIEDKE  VQGADGRYYYVLKIEDKED  KEAGISSDIYFDAINKAKT  LKEAGITAEYLFNLINKAK  KEAGITSDIYFDAINKAKT  AALSTAAGAYVSLKKRKXX  XMGFPIPDPYVWDPSFRTF  NHIKTIDFKYKGKIXXXXX  EHLVFDFDMYNPSNLSAGV  TRGQLIREAYEDYRHFSSE  QLIREAYEDYRHFSSECPF  QGQCNFTEDYAEPFPIRIF  GCVAGDEESYTVFKDLFDP  PIIQDRHGGYKPTDKHKTD  KGGDDLDPNYVLSSRVRTG  VRTGRSIKGYTLPPHCSRG  SLTGEFKGKYYPLKSMTEQ  LTGEFKGKYYPLKSMTEQE  PFMWNEHLGYVLTCPSNLG  FLVFASLVLYGHSTQDFPE  SWPSQISLQYLSGGSWYHT  LSQNDGTEQYVSVQKIMVH  WNSNNVAAGYDIALLRLAQ  LAQSVTLNNYVQLAVLPQE  TILANNNPCYITGWGRTRT  QLSQTLQQAYLPSVDYSIC  QQAYLPSVDYSICSSSSYW  DYSICSSSSYWGSTVKTTM  PLHCLVNGQYSVHGVTSFV  PTVFTRVSAYISWMNNVIA  ISWMNNVIAYTXXXXXXXX  TFLHEPAVLYNLKERYASW  AVLYNLKERYASWMIYTYS  KERYASWMIYTYSGLFCVT  RYASWMIYTYSGLFCVTVN  GLFCVTVNPYKWLPVYNAQ  HIFSISDNAYQYMLTDREN  FSISDNAYQYMLTDRENQS  TVNTKRVIQYFAVIAAIGD  KLASADIETYLLEKSRVIF  IFQLKAERDYHIFYQILSN  KAERDYHIFYQILSNKKPE  DMLLITNNPYDYAFFSQGE  LLITNNPYDYAFFSQGETT  FTPEEKNSIYKLTGAIMHF  GTEEADKSAYLMGLNSADL  HPRVKVGNEYVTKGQNVQQ  KGQNVQQVAYAIGALAKSV  AIGALAKSVYEKMFNWMVT  TLETKQPRQYFIGVLDIAG  HMFVLEQEEYKKEGIEWTF  TDMTFKAKLYDNHLGKSNN  QEAHFSLIHYAGTVDYNIL  LIHYAGTVDYNILGWLQKN  PLNETVVGLYQKSSLKLLS  KLLSNLFANYAGADAPVDK  RKGFPNRILYGDFRQRYRI  ILYGDFRQRYRILNPAAIP  GSLDIDHNQYKFGHTKVFF  VKNWPWMKLYFKIKPLLKS  SQLTRGKLTYTQQLEDLKR  HDCDLLREQYEEETEAKAE  SEVAQWRTKYETDAIQRTE  KILVEWKQKYEESQSELES  TELFKLKNAYEESLEHLET  SERRIKELTYQTEEDRKNL  DKLQLKVKAYKRQAEEAEE  PLAGSPHLEYFVRFEVPSG  DHPGFSDQVYRQRRKLIAE  KLIAEIAFQYKHGEPIPHV  HGEPIPHVEYTAEEIATWK  EEIATWKEVYVTLKGLYAT  EVYVTLKGLYATHACREHL  LEGFQLLERYCGYREDSIP  FQLLERYCGYREDSIPQLE  AFRVFQCTQYIRHASSPMH  EEIEKLSTVYWFTVEFGLC  CKQNGELKAYGAGLLSSYG  AYGAGLLSSYGELLHSLSE  NDAKDKLRNYASRIQRPFS  RPFSVKFDPYTLAIDVLDS  KGTEDELDKYSEALKDAQE  HIAEDADRKYEEVARKLVI  EKYSQKEDKYEEEIKVLSD  SIDDLEDELYAQKLKYKAI  DELYAQKLKYKAISEELDH  GNPTVEVDLYTAKGLFRAA  VPSGASTGIYEALELRDND  EAMRIGAEVYHNLKNVIKE  NLKNVIKEKYGKDATNVGD  LKSAIAKAGYTDQVVIGMD  GMDVAASEFYRAGKYDLDF  FKSPDDASRYITPDQLADL  ITPDQLADLYKSFIKDYPV  DLYKSFIKDYPVVSIEDPF  PCRSERLAKYNQILRIEEE  IFAFATCGSYTGELRLSVE  ALNIEVEFEYPFRLHQVYF  EYPFRLHQVYFDAPSCVKG  TTKIFLVGDYSSSAEFFVT  VTVAVFAFLYSMGALATYI  LYSMGALATYIFLQNKYRE  ATYIFLQNKYRENNKGPMM  EKQPAPGDAYGDAGYGQGP  AGYGQGPGGYGPQDSYGPQ  PGGYGPQDSYGPQGGYQPD  QDSYGPQGGYQPDYGQPAS  GPQGGYQPDYGQPASGGGG  GQPASGGGGYGPQGDYGQQ  GGGYGPQGDYGQQGYGQQG  PQGDYGQQGYGQQGAPTSF  NEVLMAFTSYATIILAKMM  FRIFVGARIYHTIAYLTPL  GARIYHTIAYLTPLPQPNR  NRGLAFFVGYGVTLSMAYR  GYGVTLSMAYRLLRSRLYL  SSDCPNLQTYFSRCNSVRV  RVDSGCWMLYERPNYQGHQ  CWMLYERPNYQGHQYFLRR  ERPNYQGHQYFLRRGDYPD  HQYFLRRGDYPDYQQWMGF  FLRRGDYPDYQQWMGFSDS  RLIPQHSGTYRMRIYERDD  HSGTYRMRIYERDDFRGQM  NVMEGCWVLYEMPSYRGRQ  CWVLYEMPSYRGRQYLLRP  EMPSYRGRQYLLRPGEYRR  RQYLLRPGEYRRYLDWGAA  LLRPGEYRRYLDWGAANAK  GSFRRVMDFYXXXXXXXXX  MPELIDIRQYVRTLPTNTL  AFAALTTFWYATRPKALKP  VLEDDKLLLYYYDDVRTMY  EDDKLLLYYYDDVRTMYDG  YYYDDVRTMYDGFQRGIQV  LGSRKPNQPYEWISYKQVA  PNQPYEWISYKQVAEMAEC  VTIEQGCFTYSMVVVPLYD  TYSMVVVPLYDTLGTDAIT  DTLGTDAITYIVNKAELSV  LKIIVIMDSYDNDLVERGQ  LRAALGCQFYEGYGQTECT  ALGCQFYEGYGQTECTAGC  HVGAPMPCNYIKLVDVEDM  KLVDVEDMNYQAAKGEGEV  VKGANVFKGYLKDPARTAE  HIFKLAQGEYIAPEKIENI  IAPEKIENIYLRSEAVAQV  KAKRPELRNYFRSQIDELY  YFRSQIDELYSTIKIXXXX  AVLGSLQFGYNIGVINAPQ  MGLANAAASYEILILGRFL  ILGRFLIGAYSGLTSGLVP  GLTSGLVPMYVGEIAPTHL  LPFCPESPRYLYIIRNLEG  FCPESPRYLYIIRNLEGPA  QLSGINAVFYYSTSIFELA  LSGINAVFYYSTSIFELAG  ELAGVEQPAYATIGAGVVN  LLERVPSMSYVSIVAIFGF  NFIVGMGFQYVADAMGPYV  QYVADAMGPYVFLLFAVLL  EVKPSTELEYLGPDENDXX  PSLSPGAFSYSSSSRFSSS  AALRDIRAQYENIAAKNLQ  KNLQEAEEWYKSKYADLSD  EAEEWYKSKYADLSDAANR  EQFALEAGGYQAGAARLEE  MALDIEIATYRKLLEGEES  ELDKSSIHSYXXXXXXXXX  WVAVIMTMRYSLKVLLSYH  RYSLKVLLSYHGWMFAEHG  VLSGRKPMLYSFQTSLPRL  PAVKDTVSRYLESVRPLMK  VNLGPKLQWYLKLKSWWAT  KLKSWWATNYVSDWWEEYI  NYVSDWWEEYIYLRGRGPL  VSDWWEEYIYLRGRGPLMV  RGPLMVNSNYYAMEMLYIT  GPLMVNSNYYAMEMLYITP  SNYYAMEMLYITPTHIQAA  IKDSRHIVVYHRGRYFKVW  HIVVYHRGRYFKVWLYHDG  RGRYFKVWLYHDGRLLRPR  VPWAKCRQTYFARGKNKQS  VTLDESEQGYREEDPEASI  EDPEASIDSYAKSLLHGRC  APVVGHLWEYVMATDVFQL  MATDVFQLGYSEDGHCKGD  IQLALQLAHYKDMGKFCLT  IACEKHQHLYRLAMTGAGI  GIDRHLFCLYVVSKYLAVD  LFCLYVVSKYLAVDSPFLK  LFDFEKNPDYVSCGGGFGP  GFGPVADDGYGVSYIIVGE  VADDGYGVSYIIVGENFIH  GFSDVRKVGYLRKPKSMHK  EAGGPARLEYYENEKKWRH  AGGPARLEYYENEKKWRHK  SKNKHLVALYTRDEHFAIA  DSEAEQDSWYQALLQLHNR  VGEAGEDLSYDTGPGPAFK  GQTKNLIGIYRLCLTSKTI  DGGFISSDEYGSSPCDFRS  ARGEEELSNYICMGGKGAS  STLTAPNGHYILSRGGNGH  QSSVVSIEEYTEMMPAAYP  EYTEMMPAAYPPGGGSGGR  GGSGGRLPGYRHSAFVPTH  HSAFVPTHSYPEEGLEMHH  SSNLHTDDGYMPMSPGVAP  PSNRKGNGDYMPMSPKSVS  HPQRVDPNGYMMMSPSGSC  ISAAPSGSSYGKPWTNGVG  GKLLPCTGDYMNMSPVGDS  SNTSSPSECYYGPEDPQHK  NTSSPSECYYGPEDPQHKP  DPQHKPVLSYYSLPRSFKH  PQHKPVLSYYSLPRSFKHT  LSSSSGRLRYTATAEDSSS  TSSDSLGGGYCGARPESSV  PPEPKSPGEYVNIEFGSGQ  IEFGSGQPGYLAGPATSRS  RSVPNSRGDYMTMQIGCPR  RRSSEDLSTYASINFQKQP  KKIPKQARDYIPIATDRTR  VHHGNVQETYRYLSDLFTT  HGNVQETYRYLSDLFTTLV  FNLLVFTMVYTITWLFFGF  FGFIWWLIAYVRGDLDHVG  SIETETTIGYGFRVITEKC  GMTCQARSSYMDTEVLWGH  PVLTLEKGFYEVDYNTFHD  LEKGFYEVDYNTFHDTYET  VDYNTFHDTYETNTPSCCA  TGQVSDGKDYRGIRFVDRQ  GGALGHPKVYINLDKETKT  XXXXXXMAGYLRAVSSLCR  SWPEQPRRHYAEKRIKVEK  LPHVDVQLKYFDLGLPNRD  IDSALATQKYSVAVKCATI  IGRHAHGDQYKATDFVVDR  GSGAKEWEVYNFPAGGVGM  PAGGVGMGMYNTDESISGF  SGFAHSCFQYSIQKKWPLY  YSIQKKWPLYLSTKNTIMK  STKNTIMKAYDGRFKDIFQ  IFQEIFDKHYKTDFDKNKI  TDFDKNKIWYEHRLIDDMV  AAHGTVTRHYREHQKGRPT  MKGVTSTRVYERAXXXXXX  SGNFRCDNGYCIPASWRCD  SNGQCIPSEYRCDHVSDCP  DGSDERNCHYPTCDQLTCA  QLTCANGACYNTSQRCDQK  GSGECILRAYVCDHDNDCE  DNSDERNCNYDTCGGHQFT  ESNQSHHRCYPREWACPGS  GVCSVLNCEYQCHQTPFGG  RHQCLCEEGYILERGQHCK  GMVMGVDFHYQKHRVFWTD  AVDWINNKLYLVETKVNRI  GIALDPTVGYLFFSDWGSL  TLDLVSKRVYWVDSRYDYI  KRVYWVDSRYDYIETVTYD  VYWVDSRYDYIETVTYDGI  RYDYIETVTYDGIQRKTVA  KFTETNPQVYHQSSLRPHG  SLRPHGVTVYHALRQPNAT  HRTDNGGLGYRCKCEFGFE  FDAQHSTVFYSDLSKDIIY  YSDLSKDIIYKQKIDGTGK  TFDWISRNLYWTDGGLKSV  SIVVHPTAGYMFLSDWFRP  AIDWSASRLYWVDAFFDKI  ISSVMHVKAYDADLQTGSN  DADLQTGSNYCSQTTHANG  NFQRVCGCPYGMKLQRDQM  PDHRCISPLYVCDGDKDCA  DGSSCINSRYRCDGVYDCR  NSRYRCDGVYDCRDNSDEA  FLCDNGNCIYKAWICDGDN  PSTQWQCPGYSTCINLSAL  GATCLCPLGYQLANDTKTC  SFRCACDPEYTLESDGRTC  NITAHTHNLYSLVQDVSFV  AVDWIGRNLYWTDYALETI  IGRNLYWTDYALETIEVSK  RTVIVQEKIYWPCGLSIDY  YWPCGLSIDYPNRLIYFMD  SIDYPNRLIYFMDAYLDYI  NRLIYFMDAYLDYIEFCDY  IYFMDAYLDYIEFCDYDGH  YLDYIEFCDYDGHNRRQVI  ALTLFEDFVYWTDRGTRQV  HGGNQSVVMYSVHQPLGIT  LLSAQAPRHYSCACPSGWN  VPISGIQHGYDVEFDDSEQ  EFDDSEQFIYWVENPGEIH  ALDWVSRNIYYTTPASRSI  LDWVSRNIYYTTPASRSIE  VLTLKGDTRYGKTLIANDG  AVDPARGKLYWSDHGTDSG  TLDIQEQKLYWAVTSRGVI  LAHPWGLVVYGSFLYYSDE  GLVVYGSFLYYSDEQYEVI  LVVYGSFLYYSDEQYEVIE  SFLYYSDEQYEVIERVDKS  KVVLRDNVPYLRGLRVYHR  VPYLRGLRVYHRRNAADSS  SPDGRSCSPYNSFMVVSML  DVDVANGFIYWCDFSSSVR  GSNFTNVVTYGIGANGIRG  ALDWAAGNLYFTNAFVYET  NLYFTNAFVYETLIEVLRI  IEVLRINTTYRRVLLKVSV  HIIVDPKHRYLFWADYGQK  KHRYLFWADYGQKPKIERS  GLAMDHDTGYIYWVDDSLD  AMDHDTGYIYWVDDSLDLI  DGGESQVVRYGSRYPTPYG  SQVVRYGSRYPTPYGITVF  RYGSRYPTPYGITVFGESI  ATSQEDFLIYSLNNSLRSL  VAGTAIALDYDRRNNRIFF  LNSLRGQISYVSLYSGSSS  RGQISYVSLYSGSSSPTVL  AFDWINRRIYYSDFSNQTI  FDWINRRIYYSDFSNQTIN  AIVLDPCRGYMYWTDWGTN  VLDPCRGYMYWTDWGTNAK  ALDLETDLLYWADASLQKI  AFHSFGLTVYGQYIYWTDL  SFGLTVYGQYIYWTDLYTR  GLTVYGQYIYWTDLYTRKI  GQYIYWTDLYTRKIYRANK  WTDLYTRKIYRANKYDGSD  TRKIYRANKYDGSDLVAMT  CQCPHEGNWYLANDNKYCV  NWYLANDNKYCVVDTGTRC  TCGNGRCVPYHYRCDYYND  GNGRCVPYHYRCDYYNDCG  CVPYHYRCDYYNDCGDNSD  VPYHYRCDYYNDCGDNSDE  SNGRCIPLSYVCNGINNCH  GDGSDENPIYCASHTCRSN  LSPQRCIPSYWFCDGEADC  PPNRRCIPQYWVCDGDADC  CDRRNDCGDYSDERGCSYP  DYSDERGCSYPPCHANQFT  NCTDTITSFYCSCLPGYKL  SFYCSCLPGYKLMSDKRSC  QKCENVVGSYICKCAPGYI  SYICKCAPGYIREPDGKSC  CRQNSNIEPYLIFSNRYYI  EPYLIFSNRYYIRNLTTDG  PYLIFSNRYYIRNLTTDGS  RNLTTDGSSYSLILQGLGN  DFDRVEKRLYWIDAEKQII  AVDWVSRKLYWLDAILDCL  VLHPQRGHVYWADWGVHAY  YWADWGVHAYIGRIGMDGT  EWPNAITIDYTNDLLYWAD  TIDYTNDLLYWADAHLGYI  LYWADAHLGYIEFSDLEGH  LEGHHRHTVYDGSLPHPFA  TRTVEKGNKYDGSGRVVLV  THKPFDIHVYHPYRQPIMS  PFDIHVYHPYRQPIMSNPC  CDVDNDCGDYSDEPIDECT  PIDECTTAAYNCDNHTEFS  HTEFSCKTNYRCIPQWAVC  CPTRFPNGTYCPAAMFECK  FRCDNSRCVYGHQLCNGVD  THKPCTDTEYKCSNGNCIS  SNGNCISQHYVCDNVNDCG  QSCRNSKGSYECFCVDGFK  DGFKSMSTHYGERCAADGS  LPENVRIRKYNTSSEKFSE  NTSSEKFSEYLEEEEHIQT  EEEHIQTIDYDWDPEHIGL  PEHIGLSVVYYTVLAQGSQ  EHIGLSVVYYTVLAQGSQF  SQFGAIKRAYIPNFESGSN  IREVDLGLKYLMQPDGLAV  AVDWVGRHIYWSDAKSQRI  IEVATLDGRYRKWLITTQL  GWPNGLSIDYLNDDRVYWS  IDYLNDDRVYWSDSKEDVI  KEDVIEAIKYDGTDRRLII  SLDIFEDKLYWVAKEKGEV  QVRIFHQLRYNQSVSNPCK  HLCLLRPGGYSCACPQGSD  CRCMHGGNCYFDENELPKC  KCSSGYSGEYCEVGLSRGI  ALVLVGLFHYRKTGSLLPT  QPVIFENPMYAAKDNTSKV  VPENVENQNYGRPIDPSEI  QTTNFENPIYAEMDSEVKD  ASKRNLTPGYTATEDTFKD  LMDPRSRRQYVRGRFLGKG  KALTEPEARYYLRQIVLGC  ALTEPEARYYLRQIVLGCQ  LRQIVLGCQYLHRNQVIHR  DFGLATKVEYEGERKKTLC  KKTLCGTPNYIAPEVLSKK  DVWSIGCIMYTLLVGKPPF  FETSCLKETYLRIKKNEYS  TYLRIKKNEYSIPKHINPV  LNDEFFTSGYIPARLPITC  IFWVSKWVDYSDKYGLGYQ  SKWVDYSDKYGLGYQLCDN  DYSDKYGLGYQLCDNSVGV  FNDSTRLILYNDGDSLQYI  LYNDGDSLQYIERDGTESY  YIERDGTESYLTVSSHPNS  ITLLNYFRNYMSEHLLKAG  EGDELARLPYLRTWFRTRS  LCPLMAAVTYINEKRDFRT  INEKRDFRTYRLSLLEEYG  TYRLSLLEEYGCCKELASR  CKELASRLRYARTMVDKLL  GSAGPPETVYGDPGFDRSC  PSLPQNLRNYSEEKRFECK  SEEKRFECKYCGKSFSWSS  QRTHTGEKPYKCNLCGKFF  QRIHTGEKPYRCNLCGKSF  LCGKSFTQRYVLVVHQRTH  QRTHTGERPYECNQCGKSF  QCGKSFRQSYKLIAHQRTH  QRTHTGEKPYECTQCGKSF  QCGKSFIQSYKLIAHQKIH  QKIHSGEKPYECSHCGKSF  HCGKSFSQSYKLVAHQRTH  TGEKPFECNYCGKSFSWSS  QRTHTGEKPYECNECGKSF  QRTHTGEKPYQCKQCGKSF  QCGKSFSQSYVLVVHQRTH  QRTHTGEKPYECGQCGKTF  QRTHTGEKPYECNQCGKTF  QRTHTGEKPYKCSQCGKAF  LHIGDIVSLYAEGSVNGFI  LFKVCPMNRYSAQKQYWKA  MNRYSAQKQYWKAKQTKQD  KKVHGDVVKYGSVIQLLHM  QLLHMKSNKYLTVNKRLPA  AGQPLHASNYELSDNVGCK  QEKFLTCDEYRGKLQVFLR  GGAGHWNGLYRFKHLATGN  RFKHLATGNYLAAEENPSY  YLAAEENPSYKGDVSDPKA  TDSFVPRNSYVRLRHLCTN  ELSDQKNAPYQYMFRLCYR  SDQKNAPYQYMFRLCYRVL  PYQYMFRLCYRVLRHSQED  RVLRHSQEDYRKNQEHIAK  FGMMQSQIGYDILAEDTIT  KNREPRFLDYLSDLCVSNR  VKEMAQSHEYLSIEYSEEE  QSHEYLSIEYSEEEVWLTW  NAHDENVLSYYRYQLKLFA  AHDENVLSYYRYQLKLFAR  DENVLSYYRYQLKLFARMC  FARMCLDRQYLAIDEISKQ  IPTAITIKDYDSNLNASRD  ASTMEFVEDYLNNVVGEAV  VVSLAHNLIYFGFYSFSEL  AHNLIYFGFYSFSELLRLT  QAPAAVLQAYEEPGGKNVR  QFILNVRLDYRISYLLSVF  NVRLDYRISYLLSVFKKEF  VLLHLTMHDYPPLVSGALQ  LISAQDVENYKVIKSELDR  PHGEKSSENYQIVKGILER  VMLDLLQIPYDKNDNKMME  DNKMMEILRYTHQFLQKFC  TMQHIFLNNYQLCSEISEP  WPTHGRHVQYLDFLHTVIK  HTVIKAEGKYVKKCQDMIM  NAGDDVVVFYNDKASLAHL  GVEDHSPLMYHISLVDLLA  AACAEGKNVYTEIKCTSLL  DCITEVKMAYVNFVNHCYV  AYVNFVNHCYVDTEVEMKE  DTEVEMKEIYTSNHIWTLF  RLSDPTLEKYVLTVVLDTI  AAAQRSAANYKTATRTFPR  VIPTANQWDYKNIIEKLQD  LLFLEGSEAYQRCESGGFL  QQMLQKKSKYGDRGNQLRK  QLRKMLLQNYLQNRKSGPR  GNTEIQKSFYNLMTSDKKS  SFSMPSSSRYSLGPGLHRG  LRCQNNKTNYNLVCETLQF  TGGLGLLGLYINEDNVGLV  IQTLETLTEYCQGPCHENQ  LNDISPLCKYRMDLVLQLK  ELVDVIKKAYLQEEERENS  SPREVGHNIYILALQLSRH  QEEEEDPLAYYENHTSQIE  EEEEDPLAYYENHTSQIEI  RRLRSMPLIYWFSRRMTLW  FINIIIAFFYPYVEGASTG  NIIIAFFYPYVEGASTGVL  SIAALFTKHYSVRPLIVAL  IVALVLRSIYYLGIGPTLN  VALVLRSIYYLGIGPTLNI  GNRGTFIRGYKAMVMDMEF  AMVMDMEFLYHVGYILTSV  DMEFLYHVGYILTSVLGLF  LGLFAHELFYSILLFDLIY  YSILLFDLIYREETLFNVI  TALLALILVYLFSIVGFLF  ESLFPARVVYDLLFFFIVI  IKLEHNMWNYLYFIVLVRV  LEHNMWNYLYFIVLVRVKN  LVRVKNKTDYTGPESYVAQ  KTDYTGPESYVAQMIKNKN  ITGLSPGTSYTFSIISVTT  WSNANGTASYRMLIEELTT  LTDLKPDTQYNATIYSQAA  PDTQYNATIYSQAANGTEG  SMTLTWKSNYDGSRTSIVY  YDGSRTSIVYKIHVAGGTH  ILGLSSSTLYNITVHPFLG  EGTPGFLQVYTSPDQVSDF  RNASTGNQSYMVEDLKPGT  VEDLKPGTSYHFEIIPRGP  WQNTDDASGYTYHLVLESK  NTDDASGYTYHLVLESKSG  VGSLTPGTLYNVTIFPEVD  QGISNSITQYTRPSSVSHI  KNEDAASASYAYSVLILKT  EDAASASYAYSVLILKTGD  IPELIPGVSYTVKILTQVG  LKWACPFGMYTGFELGVRS  DTECRTEVAYLNFSTSYNI  VAYLNFSTSYNISIATLSC  EASHGPIKAYAVILTTGEA  AQPSADVLKYTYEDFKRGA  PSADVLKYTYEDFKRGASD  DFKRGASDTYVTYLIRIEE  RGASDTYVTYLIRIEEKGQ  SQGLSEVLNYEIDVGNQST  VGNQSTTLGYYNGRLEPLG  GNQSTTLGYYNGRLEPLGS  NGRLEPLGSYRACVAGFTN  CVAGFTNITYNLQNDGLIN  DGLINGDESYVSFSPYSEA  DESYVSFSPYSEAVFLPQD  LIRVENFEAYFKKQQADSN  DSNCGFAEEYEDLKLIGIS  KLIGISLPKYTAEIAENRG  IAENRGKNRYNNVLPYDIS  KNRYNNVLPYDISRVKLSV  SVQTHSTDDYINANYMPGY  STDDYINANYMPGYHSKKD  YINANYMPGYHSKKDFIAT  EQGRTKCEEYWPSKQAQDY  YWPSKQAQDYGDITVAMTS  TTDLLINFRYLVRDYMKQI  INFRYLVRDYMKQIPPESP  TFIAIDRLIYQIENENTVD  IENENTVDVYGIVYDLRMH  NTVDVYGIVYDLRMHRPLM  PLMVQTEDQYVFLNQCVLD  QKDSKVDLIYQNTTAMTIY  YQNTTAMTIYENLEPVSMF  VSMFGKTNGYIAXXXXXXX  PSPSSGEGLYTNGSPMNFP  SHLHHPNVAYDCLWNYSQY  NVAYDCLWNYSQYPSANPG  YDCLWNYSQYPSANPGNNL  LLSQFPGGQYPLNGILGGN  GLNFDSQELYDSFPDQNFE  EQPELKMCGYNGSVSSVES  AAEPVSGSLYGIDDAELMG  SHRWQGETWYYGPCGKRMK  HRWQGETWYYGPCGKRMKQ  MKQFPEVIKYLSRNVVHSV  ALHDPGLPPYCQSLKILGE  EILRCFLMAYRVEPPFCDS  RAVSLGQDRYRRHYWVLPY  LGQDRYRRHYWVLPYLAGI  YRRHYWVLPYLAGIFVEGS  KFFKQVEQHYLTQLTAQPI  MSWSPKEKTYETDLAVLQW  PDSTREDLTYCEHLPDSPE  VLEQNVERRYLREPLWAAH  PDGTSTEISYEITPRVRVW  DGCDRGCHIYCHRPKMEAV  CLSQQVEEEYTQRPGFPKR  SRDSPAVPRYPEDGLSPPK  PVNPRLVSGYRRVIKNPMD  MRERLLRGGYTSSEEFAAD  LRALVQRTGYSLVQENGQR  LVQENGQRKYGGPPPGWDT  MDFNGNNRGYAFVTFSNKQ  KNAIKQLNNYEIRNGRLLG  TEGVVDVIVYPSAADKTKN  KNRGFAFVEYESHRAAAMA  DTMSSVKILYVRNLMLSTS  VERVKKIRDYAFVHFSNRE  LAKPVDKDSYVRYTRGTGG  PVDKDSYVRYTRGTGGRNT  GGRNTMLQEYTYPLSHVYD  RNTMLQEYTYPLSHVYDPT  EYTYPLSHVYDPTTTYLGA  SHVYDPTTTYLGAPVFYTP  TTYLGAPVFYTPQAYAAIP  APVFYTPQAYAAIPSLHFP  IRTPSVREIYMNVPVGAAG  GVRGLGGRGYLAYTGLGRG  GLGGRGYLAYTGLGRGYQV  LAYTGLGRGYQVKGDKRQD  KGDKRQDKLYDLLPGMELT  QKNNWGQPVYQLHSAIGQD  GQDQRQLFLYKVTIPALAS  PFTPPKLSAYVDEAKRYAA  SAYVDEAKRYAAEHTLQTL  ATSATVFPGYAVPSATAPV  VTLGQDLAAYTTYEVYPTF  GQDLAAYTTYEVYPTFAVT  LAAYTTYEVYPTFAVTTRG  FAVTTRGDGYGTFXXXXXX  XXMDHTAPTYMLANLTHLH  KVVLASISPYFKAMFTGNL  EAALQAIVEYAYTGTVFIS  ALQAIVEYAYTGTVFISQD  IGISRFAETYGCHDLYLAA  AETYGCHDLYLAATKFICQ  NVATEETVFYALESWIKYD  FYALESWIKYDVQERQKYL  KYDVQERQKYLAQLLNSVR  LSVKFLTRLYEANHLIRDD  KHLLNEALKYHFMPEHRLS  FACLDSVEMYFPQNDSWIG  AGEVFALGGYDGQSYLQSV  ALGGYDGQSYLQSVEKYIP  QSYLQSVEKYIPKIRQWQP  AAAVLDGMLYAIGGYGPAH  DGMLYAIGGYGPAHMNSVE  PAHMNSVERYDPSKDSWEM  VSHLSSIERYDPHQNQWTV  GVGAAVIDNYLYVVGGHSG  GAAVIDNYLYVVGGHSGSS  VVGGHSGSSYLNTVQKYDP  SSYLNTVQKYDPISDTWLD  TWLDSAGMIYCRCNFGLTA  XXXXXXMLLYLPQIFSSRA  ASSLLFLVPYLLFWENVAS  KLFTEFNNQYAQGKRYNDR  NNQYAQGKRYNDRIPGTCH  NEDVRCFSFYTLIRCLLRD  VNTYLEVIKYQIFNQNNCX  XXXXEDQVSYTLAITARDN  GIPQKSDTTYLEILVNDVN  NAPQFLRDSYQGSVYEDVP  LRDSYQGSVYEDVPPFTSV  DSGLNGRVFYTFQGGDDGD  RLDRENVAQYILRAYAVDK  NVAQYILRAYAVDKGMPPA  DEGTNAQIMYQIVEGNIPE  ELTALVDLDYEDRPEYILV  DLDYEDRPEYILVIQATSA  GNFEILFNNYVTNRSSSFP  DPDISDSLTYSFERGNELS  PSEDLQERLYLNRSLLTAI  RCPPGFTGDYCETEVDLCY  YCETEVDLCYSRPCGPHGH  GHCRSREGGYTCLCRDGYT  GYTCLCRDGYTGEHCEVSA  TKERDGLLLYNGRFNEKHD  GQWHTVQLKYYNKPLLGQT  QWHTVQLKYYNKPLLGQTG  LRFGAMLGNYSCAAQGTQG  PGHAILSFDYGQQKAEGNL  DSNPCPTNSYCSNDWDSYS  SYCSNDWDSYSCSCDPGYY  SYSCSCDPGYYGDNCTNVC  YSCSCDPGYYGDNCTNVCD  TRKPSAPHGYICECLPNYL  GYICECLPNYLGPYCETRI  ECLPNYLGPYCETRIDQPC  GECHCKENHYRPPSSPTCL  SPTCLLCDCYPTGSLSRVC  VTTNGCEVNYDSCPRAIEA  RNATQHTSGYFGSDVKVAY  YFGSDVKVAYQLATRLLAH  GGTAWLLQHYEAYASALAQ  AWLLQHYEAYASALAQNMR  ALAQNMRHTYLSPFTIVTP  NFAGTKLPRYEALRGERPP  GEAVASVIIYHTLAGLLPH  TLAGLLPHNYDPDKRSLRV  EILPLKTLTYVALGVTLAA  VIAILLHFLYLCTFSWALL  WALLEALHLYRALTEVRDV  DVNASPMRFYYMLGWGVPA  VNASPMRFYYMLGWGVPAF  LAVGLDPEGYGNPDFCWLS  NPDFCWLSIYDTLIWSFAG  FAVSMSVFLYILSARASCA  VNSDTLLFHYLFAACNCVQ  VQGPFIFLSYVVLSKEVRK  TTKSTLTSSYNCPSPYADG  TSSYNCPSPYADGRLYQPY  PSPYADGRLYQPYGDSAGS  YADGRLYQPYGDSAGSLHS  SRSGKSQPSYIPFLLREES  SLEDDQSGSYASTHSSDSE  TPCKNAGTCYVVDHGGIVD  VVDHGGIVDYACSCPLGFS  TCDLLTLTEYKCRCPPGWS  GQCLPFESSYICGCPPGFH  GTCHNEIGSYRCACRATHT  HTGPHCELPYVPCSPSPCQ  GACVDGVNTYNCRCPPEWT  RCPPEWTGQYCTEDVDECQ  GTCHNSHGGYNCVCVNGWT  TCHDRVASFYCECPHGRTG  KAICTCPRGYTGPACSQDV  SFECQCLQGYTGPRCEIDV  EFQCICMPGYEGVYCEINT  ICMPGYEGVYCEINTDECA  KGFSGHLCQYDVDECASTP  AKCLDGPNTYTCVCTEGYT  TYTCVCTEGYTGTHCEVDI  TFTCLCQPGYTGHHCETNI  GGTCQDRDNYYLCLCLKGT  GTCQDRDNYYLCLCLKGTT  GTCLDKIDGYECACEPGYT  GYECACEPGYTGSMCNVNI  GFTCRCPEGYHDPTCLSEV  GACRDGLNGYKCDCAPGWS  GTCKDMTSGYVCTCREGFS  GTCIDDVAGYKCNCPLPYT  GYKCNCPLPYTGATCEVVL  SGVCKESEDYESFSCVCPT  ASCQNTNGSYRCLCQAGYT  SYRCLCQAGYTGRNCESDI  ANCTDCVDSYTCTCPTGFN  LCPPGFTGSYCQYDVNECD  PGFTGSYCQYDVNECDSRP  LHGGTCQDSYGTYKCTCPQ  GTCQDSYGTYKCTCPQGYT  TYKCTCPQGYTGLNCQNLV  GKCWQTNTQYHCECRSGWT  LCVDEEDKHYCHCQAGYTG  KHYCHCQAGYTGSYCEDEV  HCQAGYTGSYCEDEVDECS  CQNGATCTDYLGGFSCKCV  GTCVDQVGGYTCTCPPGFV  SPCVGSNPCYNQGTCEPTS  CEPTSESPFYRCLCPAKFN  NGLLCHILDYSFTGAAGRD  CTQSLQCWKYFSDGHCDSQ  LTEGQCNPLYDQYCKDHFS  GQCNPLYDQYCKDHFSDGH  AQGQQMIFPYYGREEELRK  QGQQMIFPYYGREEELRKH  PMDIHGSIVYLEIDNRQCV  ASLGSLNIPYKIEAVKSET  PLPSQLHLMYVAAAAFVLL  ETALHLAARYSRSDAAKRL  LFLAAREGSYETAKVLLDH  HDIVRLLDEYNLVRSPQLH  SPTLCSPNGYLGNLKSATQ  VDSLESPHGYLSDVASPPL  LQNGMVPSQYNPLRPGVTP  STNTLSPIIYQGLPNTRLA  FLTPPSQHSYSSSPVDNTP  XXXXXXMVDYHAANQAYQY  VDYHAANQAYQYGPSSGGN  YHAANQAYQYGPSSGGNGT  TGGGGGMGDYMAQEDDWDR  LWCQRKTAPYKNVNVQNFH  HRHRPELIEYDKLRKDDPV  NNAFEVAEKYLDIPKMLDA  AIMTYVSSFYHAFSGAQKA  QENEHLMEDYERLASDLLE  QQKLEDFRDYRRVHKPPKV  QHLEQAEKGYEEWLLNEIR  KEAMLKHRDYETATLSDIK  IAQELNELDYYDSHNVNTR  AQELNELDYYDSHNVNTRC  ETIDQLHLEYAKRAAPFNN  HIKLSGSNPYTSVTPQIIN  EDQLSHLKQYERSIVDYKP  KQYERSIVDYKPNLDLLEQ  LIFDNKHTNYTMEHLRVGW  FKACLISLGYDVENDRQGD  RELPPDQAEYCIARMAPYQ  EYCIARMAPYQGPDAAPGA  PDAAPGALDYKSFSTALYG  DYKSFSTALYGESDLXXXX  LSGKKSAIPYGTKSQYILS  AIPYGTKSQYILSVSRENL  KDLLTAAEKYPNVKMHFNH  DLIVGCDGAYSTVRSHLMK  HLMKKPRFDYSQQYIPHGY  KPRFDYSQQYIPHGYMELT  YSQQYIPHGYMELTIPPKN  LTIPPKNGDYAMEPNYLHI  NGDYAMEPNYLHIWPRNTF  NDVVDFFQKYFPDAIPLIG  DHAISDLSMYNYIEMRAHV  AISDLSMYNYIEMRAHVNS  IMPSTFIPLYTMVTFSRIR  TMVTFSRIRYHEAVQRWHW  GSLIAISSTYLLIHYMSPR  ISSTYLLIHYMSPRSFLRL  LSCLDHVISYYHVASDTEK  SCLDHVISYYHVASDTEKI  EGPTGRLEEYLGSMAKIQK  MAKIQKAVEYFQDNSPDSP  IRISRWLVEYGRNQDFMNV  GRNQDFMNVYYQIRSSQLD  RNQDFMNVYYQIRSSQLDR  KSSSSSGVPYSPAIPNKRK  DMLDVETDAYIHCVSAFVR  AFVRLAQSEYQLLMGIIPE  QETSSSATSYNSEFSKRLL  EFSKRLLSTYICKVLGNLQ  LNLLSKSKVYEDPALSAIF  LSAIFLHNNYNYILKSLEK  AIFLHNNYNYILKSLEKSE  VTQKTAERSYREHIEQQIQ  EHIEQQIQTYQRSWLKVTD  QRSWLKVTDYIAEKNLPVF  AQKSIVKETYGAFLHRYSS  ETYGAFLHRYSSVPFTKNP  VPFTKNPEKYIKYRVEQVG  TKNPEKYIKYRVEQVGDMI  KASRKLPKLYGDIPPELVA  KPLEDLDPFYKDHKTFMVL  MVLNKKRTIYRFSAKRALF  RSFDNDIPEYVFIGIYILE  IPEYVFIGIYILEAVIKIL  KTTLNPDNNYTKFDNFGWS  MTQDSWERLYRQILRTSGI  RQILRTSGIYFVFFFVVVI  VVVIFLGSFYLLNLTLAVV  LTLAVVTMAYEEQNRNVAA  PCGKNLASKYLVWDCSPQW  CLKIIALDPYHYFRHGWNV  KIIALDPYHYFRHGWNVFD  ALLSLADVLYNTLSDNNRS  FGTKFNKTAYATQERPRRR  RRRWHMDNFYHSFLVVFRI  LPDARPWKEYDTDMALYTG  KEYDTDMALYTGQAGAPLA  LAEVEDDVEYCGEGGALPT  LWWNIRKTCYQIVKHSWFE  KWVAFGFRRYFTSAWCWLD  QFEGMKVVVYALISAIPAI  CINGTDINMYLDFTEVPNR  NRSQCNISNYSWKVPQVNF  VNFDNVGNAYLALLQVATY  YLALLQVATYKGWLEIMNA  EQPDFEANLYAYLYFVVFI  PDFEANLYAYLYFVVFIIF  FEANLYAYLYFVVFIIFGS  IFMTEEQKKYYNAMKKLGT  FMTEEQKKYYNAMKKLGTK  IKVFALRQHYFTNGWNLFD  LLLFLVMFIYAIFGMSWFS  CQQPQIAVVYFVSYIIISF  QIAVVYFVSYIIISFLIVV  ISFLIVVNMYIAVILENFN  LGEDDFEIFYEVWEKFDPE  DPEASQFIQYSALSDFADA  MEANPFKKLYEPIVTTTKR  QGAAVIQRAYRKHMEKMVK  VQKSRNGGVYPGTSGEKKL  GKPPKRNAFYRKLQNFLYN  FYRKLQNFLYNVLERPRGW  ERPRGWAFIYHAYVFLLVF  RGWAFIYHAYVFLLVFSCL  LSVFSTIKEYEKSSEGALY  YEKSSEGALYILEIVTIVV  VTIVVFGVEYFVRIWAAGC  IWAAGCCCRYRGWRGRLKF  HSKELVTAWYIGFLCLILA  CLILASFLVYLAEKGENDH  KGENDHFDTYADALWWGLI  GLITLTTIGYGDKYPQTWN  LTTIGYGDKYPQTWNGRLL  GLIQSAWRFYATNLSRTDL  RTDLHSTWQYYERTVTVPM  TDLHSTWQYYERTVTVPMI  PMISSQTQTYGASRLIPPL  RKFKESLRPYDVMDVIEQY  YDVMDVIEQYSAGHLDMLS  KKLDFLVSIYTQRMGIPPA  GIPPAETEAYFGAKEPEPA  AKEPEPAPPYHSPEDSRDH  STSSTGQRKYAAPPVMPPA  PAHERSLSAYSGGNRASTE  ENLNALASCYAAVAPCAKV  VAPCAKVRPYIAEGESDTD  SGAGGCCGAYTPAREGLSV  SVILVEASEYFGGTTAYSG  SEYFGGTTAYSGGGGVWFP  DDTIEDALTYYPRVVGDRT  DTIEDALTYYPRVVGDRTP  RTPHELQEAYVRGGAPLID  VRGGAPLIDYLESDDDLEF  EFMVYPWPDYFGKAPKARA  GRFLIALRKYPNVDLYRNT  LRKYPNVDLYRNTPLEELI  DQNDEMRGKYGVPGAARDS  QDGARFTNEYAPYDRLGRD  ARFTNEYAPYDRLGRDVIA  EMTLPFWMIYDDRNGEAPP  NVPLVETEKYVDAGLWKTA  EDFGRGDEPYDLAFTGGGS  SEGAPIPGLYAAGNTMAAP  TMAAPSGTVYPGGGNPIGA  XXXMAVSKVYARSVYDSRG  VSKVYARSVYDSRGNPTVE  AAAEKNVPLYKHLADLSKS  DLSKSKTSPYVLPVPFLNV  NLKSLTKKRYGASAGNVGD  LTGPQLADLYHSLMKRYPI  DLYHSLMKRYPIVSIEDPF  FPTGKESGNYLAIDLGGTN  HTFDTTQSKYKLPHDMRTT  TLPLGFTFSYPASQNKINE  DTVGTLIASYYTDPETKMG  TVGTLIASYYTDPETKMGV  FGTGVNGAFYDVVSDIEKL  NSPMAINCEYGSFDNEHLV  EHLVLPRTKYDVAVDEQSP  IAAICQKRGYKTGHIAADG  GHIAADGSVYNKYPGFKEA  AADGSVYNKYPGFKEAAAK  AAAKGLRDIYGWTGDASKD  SHFATCPSVYEANHPGLEI  QKSFLPSINYPQILAPYAP  INYPQILAPYAPEVNYNDK  LAPYAPEVNYNDKIKKVYI  NYNDKIKKVYIEKIIPLIS  AKFQSDIPLYTKLIKSKDV  ERLTKKAEVYGVDPTNESG  SGERRITPEYLVKIYKEIV  PITKEVEVEYLLRRLEEXX  PSSTGTDGGYYYSWWTDGA  SSTGTDGGYYYSWWTDGAG  STGTDGGYYYSWWTDGAGD  WTDGAGDATYQNNGGGSYT  TYQNNGGGSYTLTWSGNNG  PGAASRSISYSGTYQPNGN  SRSISYSGTYQPNGNSYLS  GTYQPNGNSYLSVYGWTRS  PNGNSYLSVYGWTRSSLIE  GWTRSSLIEYYIVESYGSY  WTRSSLIEYYIVESYGSYD  LIEYYIVESYGSYDPSSAA  GSVTCNGATYDILSTWRYN  TYDILSTWRYNAPSIDGTQ  GMNLGSEHNYQIVATEGYQ  NYQIVATEGYQSSGTATIT  XXXMLVMTEYLLSAGICMA  AISNVSKGQYAKRFFFFAT  RSGSEDPTVYSATSTKKLH  IDGDTVKLMYKGQPMTFRL  KHPKKGVEKYGPEASAFTK  FDKGQRTDKYGRGLAYIYA  TDKYGRGLAYIYADGKMVN  KYGRGLAYIYADGKMVNEA  VRQGLAKVAYVYKPNNTHE  KELNDLEKKYNAHIGVYAL  KKYNAHIGVYALDTKSGKE  KFNSDKRFAYASTSKAINS  SAILLEQVPYNKLNKKVHI  VAYSPILEKYVGKDITLKA  KALIEASMTYSDNTANNKI  GDKVTNPVRYEIELNYYSP  PVRYEIELNYYSPKSKKDT  VRYEIELNYYSPKSKKDTS  LIKDGVPKDYKVADKSGQA  ADKSGQAITYASRNDVAFV  ASRNDVAFVYPKGQSEPIV  VGKDAEITIYADGSDESDA  GVWQGKRVSYERVFSNECE  SHPLMDFNNYGCYCGLGGS  LMDFNNYGCYCGLGGSGTP  RCCETHDNCYRDAKNLDSC  LVDNPYTESYSYSCSNTEI  DNPYTESYSYSCSNTEITC  AAICFSKAPYNKEHKNLDT  LLLVTLTVAYAVPDPRGII  DFLEQNELQYEVLINNLRS  SRVRTTGHSYEKYNNWETI  RTTGHSYEKYNNWETIEAW  GTTFLGNNIYLLKVGKPGP  VREAVLTYGYESHMTEFLN  TEFLNKLDFYVLPVLNIDG  VLPVLNIDGYIYTWTKNRM  PVLNIDGYIYTWTKNRMWR  ASTDPCDETYCGSAAESEK  RNNLSSIKAYLTIHSYSQM  IHSYSQMILYPYSYDYKLP  SYSQMILYPYSYDYKLPEN  MILYPYSYDYKLPENNAEL  AAVKELATLYGTKYTYGPG  ELATLYGTKYTYGPGATTI  DWAYDQGIKYSFTFELRDK  CEETMLAIKYVTNYVLGHL  MLAIKYVTNYVLGHLXXXX  XXXXXXMAPYSLLVTRLQK  LQKALGVRQYHVASVLCQR  AMSHFEPHEYIRYDLLEKN  HFEPHEYIRYDLLEKNIDI  NQEIERGKTYLRLRPDRVA  NFLATAGAKYGVGFWRPGS  IIHQIILENYAYPGVLLIG  HQIILENYAYPGVLLIGTD  KGGTGAIVEYHGPGVDSIS  IGATTSVFPYNHRMKKYLS  FPYNHRMKKYLSKTGRADI  HLVPDPGCHYDQVIEINLS  LIGSCTNSSYEDMGRSAAV  IRATIERDGYAQVLRDVGG  AEFDPGQDTYQHPPKDSSG  FGPVPDTARYYKQHGIRWV  GPVPDTARYYKQHGIRWVV  RWVVIGDENYGEGSSREHR  PLTFADPADYNKIHPVDKL  IVRELTGGIYFGEPRGMSE  EAEAWNTERYSKPEVERVA  KTVEEVGRGYPDVALEHQY  YPDVALEHQYVDAMAMHLV  XXXXXXXMSYSITTPSQFV  RFPDSDFKVYRYNAVLDPL  PDSDFKVYRYNAVLDPLVT  VVKKSGILSYSAYGCYCGW  KSGILSYSAYGCYCGWGGR  ILSYSAYGCYCGWGGRGKP  RCCFVHDCCYGKVTGCNPK  NLDTYDRNKYWRYPASNCQ  TYDRNKYWRYPASNCQEDS  NEFTITGSTYAPIGEVQKD  DDKPVKCHQYDGLVELATI  ALCNDSALDYNEAKGVYEK  LDYNEAKGVYEKVGEATET  YNNMKQFIRYLISSNVGEV  LISGWLFFRYLAIGCYVGA  FFRYLAIGCYVGAATVGAA  ADGGPRVSFYQLSHFLQCK  VDCAIFESPYPMTMALSVL  SMSLHFLILYVEPLPLIFQ  LIMPLVVWVYSTDTNFSDM |
